# A Deep Learning Model for Chili Pepper Fruit Shape Classification Using DenseNet-121 and CBAM

**DOI:** 10.3390/plants15132103

**Published:** 2026-07-07

**Authors:** Zongjun Li, Yinghua Li, Hu Zhao, Liping Huang, Zengjing Zhao, Jianjie Liao, Meng Wang, Xing Wu, Mingxia Gong, Zhi He, Liyan Liu, Risheng Wang

**Affiliations:** 1 Guangxi Academy of Agricultural Sciences, Nanning 530007, China; lizongjun.gxaas@gmail.com (Z.L.);; 2 College of Agriculture and Biology, Guangxi Minzu Normal University, Chongzuo 532200, China

**Keywords:** chili pepper, fruit shape, deep learning, DenseNet-121, CBAM

## Abstract

Traditional manual grading of fresh chili peppers suffers from inconsistent quality control and low efficiency. To meet the demand for accurate fruit shape recognition during the post-harvest stage, this study proposes an intelligent recognition method based on an improved DenseNet-121 network. This approach facilitates the application of machine vision in agricultural sorting equipment. DenseNet-121 serves as the backbone network. The Convolutional Block Attention Module (CBAM) is introduced to enhance feature focus on fruit shapes. A regularization strategy (Dropout = 0.3, weight decay = 1 × 10^−4^) and a cross-entropy loss function with label smoothing (LS = 0.1) are integrated to optimize decision boundaries. These configurations prevent the model from overfitting to hard training labels and yield a robust classification architecture. Experimental results demonstrate that the proposed model achieves a precision of 90.09%, a recall of 89.60%, an F1-score (the harmonic mean of precision and recall) of 89.53%, and an overall accuracy of 89.74%. The model contains 7.09 M parameters and requires a single-frame inference time of 7.35 ms. Comprehensive evaluations indicate that the proposed model achieves an optimal balance among environmental noise robustness, prediction accuracy, and computational efficiency. Consequently, by maintaining high fine-grained classification accuracy alongside a low memory footprint and rapid inference speed, the model demonstrates strong potential for real-time deployment on resource-constrained edge devices within actual agricultural optical sorting equipment.

## 1. Introduction

Chili peppers (*Capsicum* spp.) are cultivated globally as vegetable and spice crops. In China, they rank first among vegetable crops in cultivation area and production value. To date, approximately 42 species within the genus Capsicum have been described. This includes five major cultivated species: *Capsicum annuum* L., *Capsicum frutescens* L., *Capsicum chinense* Jacq., *Capsicum baccatum* L., and *Capsicum pubescens* Ruiz & Pav [1,2]. Through long-term domestication and modern breeding, pepper fruits exhibit diverse morphologies. Fruit shape is a primary agronomic trait for distinguishing cultivars and a determining factor for commercial value [3,4]. In the global pepper market, consistent fruit morphology affects market premiums and consumer preferences [5,6,7]. It is also a critical parameter for evaluating mechanized harvesting adaptability, processing quality control, and brand identity [8,9,10]. As multimodal perception, deep learning, and intelligent control technologies are integrated into agricultural machinery, developing efficient and accurate methods for pepper fruit shape recognition is necessary [11,12].

Traditionally, pepper quality grading has relied on manual visual screening based on human experience. However, rising labor costs and limited mechanization in key processing stages restrict industrial efficiency [13,14,15]. The advancement of machine learning has introduced new approaches to modern agricultural intelligence [16,17,18]. Driven by their performance in image classification, deep convolutional neural networks (CNNs) have become a primary focus of research. For instance, Xiang developed a residual attention neural network (RAN-11) based on Attention-56 and Attention-92 architectures to improve phytoplankton recognition accuracy in Lake Taihu under complex backgrounds [19]. Shang proposed a plant disease recognition method using a deep residual network with a hybrid attention mechanism [20]. Joshi applied a YOLOv8-based deep learning method with k-fold cross-validation for cotton leaf disease classification. Similarly [21], Bouhouch combined Cascade R-CNN and U-Net architectures to develop a quantitative model for monitoring symptom progression in barley seedlings infected with net blotch [22].

Deep neural networks are routinely applied to standard agricultural computer vision tasks, including crop disease identification [23,24], fruit maturity assessment [25,26], and quality grading [27,28]. However, automated chili pepper shape recognition is inherently a fine-grained visual classification task [29,30]. Unlike general image recognition [31,32], fine-grained classification deals with subtle inter-class variations. Several pepper shape categories exhibit similar global contours and can only be distinguished by localized morphological differences, complicating feature extraction and classification [33,34]. For instance, Barbosa [12] demonstrated that when utilizing YOLOv8m for pepper variety classification, visually similar cultivars—such as Fidalga, Habanero, and Scotch Bonnet—yielded significantly lower detection accuracies compared to morphologically distinct varieties. This performance degradation was attributed to the high visual resemblance among these rounded pepper types. Furthermore, this issue was exacerbated by standard data augmentation techniques that introduced unrealistic spatial variations, ultimately impairing the model’s ability to discern subtle inter-class differences. Similarly, in general agricultural object detection, models frequently misclassify visually analogous items, such as erroneously identifying red bell peppers as tomatoes due to shared shape and color profiles. These failure modes underscore the inherent complexity of fine-grained morphological classification in *Capsicum* species, where capturing subtle geometric variations is critical. Additionally, deploying models on mobile and embedded agricultural devices is constrained by limited storage and computational resources. This necessitates reducing model complexity while maintaining classification accuracy. Therefore, developing an end-to-end network architecture that balances precise feature extraction with computational efficiency for pepper shape classification is a critical technical requirement for intelligent agricultural equipment.

In formulating the proposed framework, DenseNet-121 was strategically selected as the backbone network over other ultra-lightweight models (such as the MobileNet series). While models like MobileNet are highly optimized for mobile devices, they frequently employ depthwise separable convolutions and aggressive spatial downsampling, which can inadvertently discard the subtle, low-level geometric features required for fine-grained morphological classification. In contrast, the dense connectivity pattern of DenseNet-121 ensures continuous feature reuse, explicitly preserving critical low-level morphological details—such as localized tip curvatures and basal contours—throughout the network depth while maintaining high parameter efficiency.

This study represents the first attempt to integrate DenseNet-121 with the Convolutional Block Attention Module (CBAM) [35,36], coupled with a specifically optimized regularization strategy, specifically for the fine-grained morphological classification of pepper fruits. While existing studies have extensively employed standard deep learning architectures for general agricultural tasks—such as disease detection or macroscopic yield estimation—they typically utilize out-of-the-box models that struggle with the subtle inter-class geometric similarities inherent to pepper phenotypes. The primary innovation of this work lies in formulating a specialized framework that differs from existing generalized studies in two key aspects: (1) it adaptively forces the network to focus on crucial localized structural variations via dual-channel attention, overcoming the limitations of models that rely solely on global contours; and (2) it rigorously mitigates the risk of overfitting against environmental noise and data augmentation artifacts through empirically validated joint regularization. Consequently, this provides a highly robust and specialized algorithmic foundation for agricultural post-harvest sorting equipment.

## 2. Dataset Construction and Data Augmentation

### 2.1. Dataset Construction

**Figure 1:**
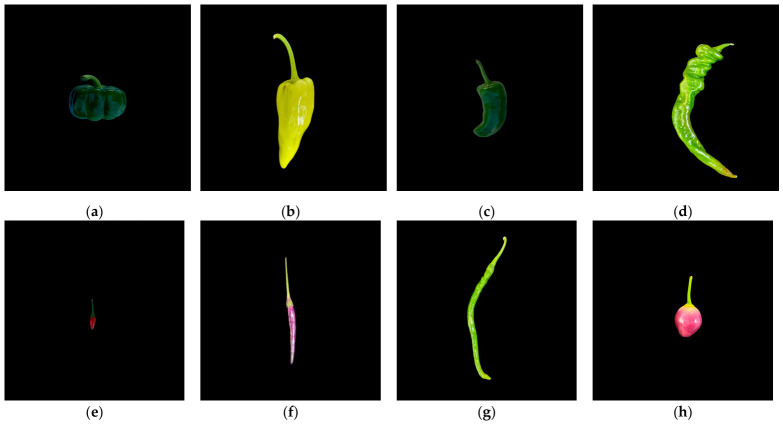
Representative images of the eight distinct chili pepper fruit shape categories in the dataset: (**a**) Lantern; (**b**) Cone; (**c**) Horn; (**d**) Goat-horn; (**e**) Short-finger; (**f**) Long-finger; (**g**) Linear; (**h**) Round.

The chili pepper image dataset was collected at the Lijian Research Base of the Guangxi Academy of Agricultural Sciences, China (23.24° N, 108.06° E). Fruit samples were harvested during the commercial maturity stage, and their shapes were classified according to the Descriptors and Data Standard for Pepper [37]. As illustrated in Figure 1, the dataset encompasses eight distinct fruit shape categories: lantern (152 images), cone (130 images), horn (272 images), goat-horn (254 images), short-finger (159 images), long-finger (373 images), linear (217 images), and round (75 images). The original image dataset is provided as Supplementary Materials.

### 2.2. Data Augmentation

Data augmentation is driven by three primary considerations: enhancing the robustness of the neural network in continuous phenotypic feature extraction, mitigating overfitting during the early stages of training, and simulating varying fruit orientations on sorting pipelines in realistic environments.

The originally collected images underwent preprocessing and targeted data augmentation (Table 1). Utilizing the Albumentations computer vision library, all images were first processed to remove background scales and standardized to a resolution of 512 × 512 pixels to eliminate background interference. The training dataset was then augmented using rotation, flipping, scaling, high-frequency noise injection, and blurring. To address the class imbalance inherent in the naturally collected data, a dynamic class-specific data augmentation strategy was implemented, yielding a balanced dataset containing a total of 3200 images with a uniform class distribution. To prevent data leakage during the subsequent dataset partitioning process, a strict homologous isolation strategy was implemented. The dataset split was executed based exclusively on the unique original source images rather than the entire augmented image pool. Consequently, all augmented derivatives generated from a specific source image were strictly grouped into the identical subset as their origin.

To address the limitations of the initial dataset, the samples within each phenotypic category were partitioned into training, validation, and test sets at a ratio of 7:1.5:1.5 to enhance the volume and diversity of the training data. Table 2 and Table 3 list the parameters required to ensure the reproducibility of the training process, establishing a standard training protocol that enables model replication under similar computational conditions.

## 3. Model Development

### 3.1. Network Framework

**Figure 2:**
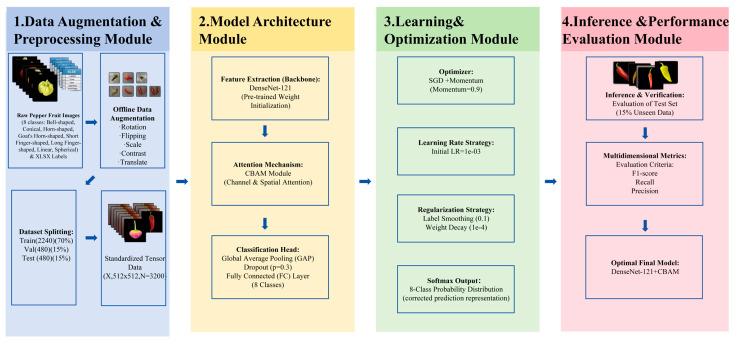
Overall workflow of the chili pepper fruit shape classification framework.

This study proposes an end-to-end image classification architecture that integrates CBAM into a DenseNet-121 backbone. By leveraging the advantages of dense connections in feature reuse and gradient propagation, alongside the capability of the dual spatial and channel attention mechanisms within CBAM to focus on critical localized morphological variations, the network enables fine-grained classification and automated assessment of chili pepper fruit shapes. The overall framework of the proposed network is illustrated in Figure 2.

The overall network framework comprises four primary modules. Compared with standard deep learning models designed for general image classification, the proposed framework differs by incorporating a targeted data augmentation strategy to enhance sample diversity and mitigate class imbalance, integrating CBAM to accentuate critical localized morphological features, and applying dropout, label smoothing (LS), and weight decay (WD) to mitigate overfitting in deep architectures. The specific structure and function of each module are detailed below:

1. Data Augmentation and Preprocessing Module: This module utilizes an offline targeted data augmentation strategy via the Albumentations computer vision library to construct a class-balanced dataset, thereby enhancing model robustness directly at the data source level.
2. Model Architecture Module: This module forms the core of the chili pepper fruit shape classification process. It utilizes DenseNet-121 as the backbone feature extraction network, leveraging dense connections to promote feature reuse and optimize gradient propagation. Subsequently, CBAM is integrated to adaptively recalibrate feature maps by sequentially applying channel and spatial attention mechanisms, thereby directing the model’s focus toward critical regions of fruit shape heterogeneity. Finally, the refined features are processed through a global average pooling (GAP) layer, a dropout layer, and a fully connected (FC) layer to map the outputs into the eight distinct fruit shape categories.
3. Learning and Optimization Module: This module governs model weight iteration and parameter optimization. Training is driven by a Stochastic Gradient Descent (SGD) optimizer with an initial learning rate of 1 × 10^−3^, which is dynamically adjusted via a cosine annealing schedule. Concurrently, a cross-entropy loss function with LS is employed alongside an L2 WD of 1 × 10^−4^. This configuration mitigates overfitting, facilitating feature learning for highly similar chili pepper fruit shapes.
4. Inference and Performance Evaluation Module: This module verifies model generalization capabilities. Once training convergence is achieved, forward inference is executed on an independently reserved test set, where discriminative and classification performance is assessed across multiple dimensions using precision, recall, F1-score, and a confusion matrix.

### 3.2. Model Construction and Optimization Strategies

**Figure 3:**
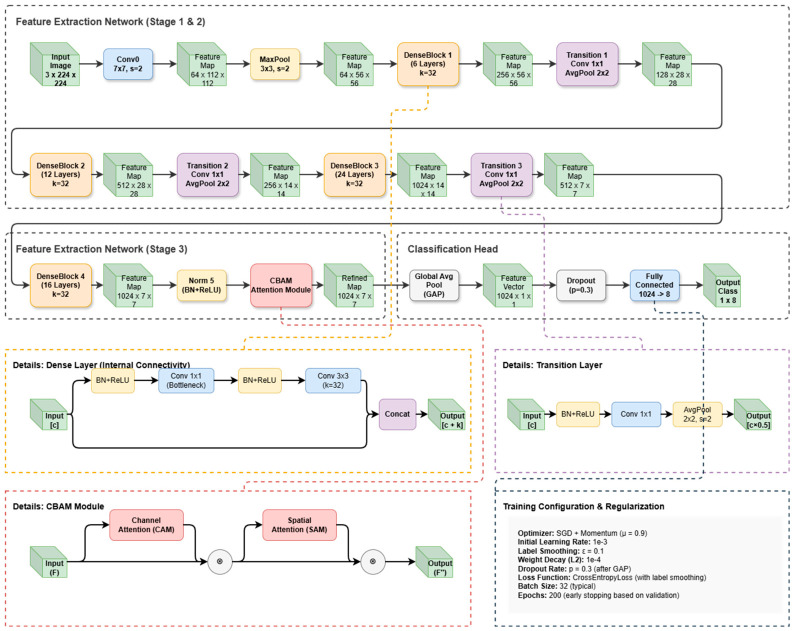
Overall architecture of the proposed modified DenseNet-121 model integrated with the CBAM for chili pepper fruit shape classification. The dashed boxes detail the internal connectivity of the Dense Layer, Transition Layer, CBAM, and the specific training configurations, the dashed arrows are color-coded to match their corresponding functional modules, indicating the direction of data flow through the framework.

To address the high inter-class morphological similarity and complex localized morphological heterogeneity in chili pepper fruit shape classification, this study integrates the CBAM into a DenseNet-121 backbone. The overall network architecture, illustrated in Figure 3, further incorporates dropout regularization [38] after the GAP layer [39], WD during [40] the optimization phase, and a cross-entropy loss function with LS [41].

#### 3.2.1. DenseNet-121 Backbone

To meet the dual requirements of high accuracy and real-time processing for chili pepper fruit shape classification, DenseNet-121 was selected as the backbone network. Through dense connectivity, this architecture mitigates the vanishing gradient and feature degradation issues commonly encountered in deep convolutional networks during backpropagation, thereby improving convergence efficiency. Specifically, during the data loading phase, the stored 512 × 512 pixel images are dynamically resized to 224 × 224 pixels. The DenseNet-121 backbone processes a 224 × 224 three-channel RGB input image through an initial convolutional layer and a max-pooling layer, sequentially followed by four dense blocks and corresponding transition layers. Each transition layer comprises a 1 × 1 convolution and a 2 × 2 average pooling operation, progressively downsampling the feature maps to a dimension of 512 × 7 × 7. Following the fourth dense block and a batch normalization-ReLU (BN-ReLU) sequence, a final 1024 × 7 × 7 feature map is generated. This feature map is subsequently fed into CBAM and the classification head. The non-linear feature propagation process within DenseNet is expressed as Equation (1):

(1) $$X_{l}=H_{l}\left(\left[X_{0},X_{1},\ldots ,X_{l-1}\right]\right),$$

where l denotes the index of the current layer; $X_{l}$ represents the output feature map of the l th layer; $\left[X_{0},X_{1},\ldots ,X_{l-1}\right]$ denotes the concatenation of the feature maps from layers 0 to l−1 along the channel dimension; and $H_{l}\left(\left[.\right]\right)$ defines a non-linear composite function comprising batch normalization (BN), a rectified linear unit (ReLU), and a 3 × 3 convolution.

#### 3.2.2. Convolutional Block Attention Module

CBAM is a lightweight feature enhancement mechanism designed for feed-forward convolutional neural networks. By sequentially recalibrating feature weights across the channel and spatial dimensions, it enhances the network’s representational capacity for critical features while suppressing redundant noise.

In this study, this module serves as the central hub for feature refinement, capturing and enhancing localized morphological variations in chili peppers. To address the high inter-class similarity among different fruit shapes and the susceptibility of images to background and illumination variations during acquisition, CBAM is integrated into the DenseNet-121 backbone. This architecture strengthens the feature responses of target fruit boundaries, contours, and geometric deformations while attenuating the weights of non-critical background regions, thereby improving fine-grained classification accuracy and robustness against interference.

CBAM operates through two sequential sub-modules: the Channel Attention Module (CAM) and the Spatial Attention Module (SAM). Given an input feature map, the CAM first generates a 1D channel attention map, which is multiplied element-wise with the input features. The intermediate output is then processed by the SAM to produce a 2D spatial attention map, which is subsequently multiplied by the intermediate features to yield the final refined feature map. The overall composite process of feature refinement can be formulated as follows:

(2) $$F^{\prime }=M_{c}(F)⊗F,$$

(3) $$F^{\prime \prime }=M_{s}\left(F^{\prime }\right)⊗F^{\prime },$$

(4) $$M_{c}(F)=\sigma \left(MLP\left(AvgPool(F)\right)+MLP\left(MaxPool(F)\right)\right),$$

(5) $$M_{s}(F)=\sigma \left(f^{7\times 7}\left(\left[AvgPool\left(F^{\prime }\right);MaxPool\left(F^{\prime }\right)\right]\right)\right),$$

where ⊗ denotes element-wise multiplication; $F^{\prime }$ represents the intermediate feature map weighted by channel attention; $F^{\prime \prime }$ denotes the final refined output feature map; σ represents the sigmoid activation function; $f^{7\times 7}$ denotes a convolution operation with a 7 × 7 filter kernel; and $\left[.;.\right]$ indicates feature concatenation along the channel dimension.

#### 3.2.3. Global Average Pooling

GAP is a downsampling technique that reduces spatial dimensions by computing the mean of all spatial pixels within each feature map. It is designed to replace the redundant FC layers traditionally located at the end of CNNs, thereby reducing the overall number of model parameters and mitigating overfitting.

In this study, to avoid the parameter explosion and overfitting risks associated with feeding high-dimensional feature maps directly into FC layers, while preserving the discriminative features enhanced by CBAM, a GAP layer is inserted between the CBAM and the dropout layer. This operation compresses the spatial resolution from *7 × 7* to 1 × 1 and flattens the feature maps into a 1024-dimensional vector, thereby reducing computational complexity without compromising classification accuracy.

Operationally, GAP applies global spatial pooling to the *C × H × W* feature map output by the final residual stage. By averaging all pixel values within each individual channel, it ultimately yields a C-dimensional feature vector. Let $x\in R^{C\times H\times W}$ denote the feature map output from the final residual block. For the *c*-th feature channel, its output value $y_{c}$ after applying GAP is calculated as shown in Equation (6):

(6) $$y_{c}=\frac{1}{H\times W}\sum_{i=1}^{H}\sum_{j=1}^{W}x_{c,i,j},$$

where *C* denotes the number of channels; *H* and *W* represent the height and width of the feature map, respectively; and $x_{c,i,j}$ indicates the pixel value at spatial coordinates (*i,j*) within the *c*-th feature channel. Following this operation, the 3D feature tensor is flattened into a 1D global feature vector $Y=[y_{1},y_{2},\ldots ,y_{512}]$.

#### 3.2.4. Dropout

Dropout is a regularization technique employed during the training of deep neural networks that randomly sets the activations of a fraction of neurons to zero based on a specified probability. This mechanism is designed to break complex co-adaptations among neurons, thereby forcing the network to learn more independent and robust representations.

In this study, given the limited number of training samples for the chili pepper fruit shape classification task and the susceptibility of DenseNet-121 to overfitting on fine-grained features, a dropout layer is inserted between the GAP layer and the FC classification layer. By randomly dropping a fraction of the neuronal outputs, this configuration forces individual neurons to learn more independent and complementary contour phenotypes, thereby mitigating the risk of overfitting and ensuring the stability of the classification system.

Given the 1D feature vector output by the GAP layer $Y=[y_{1},y_{2},\ldots ,y_{512}]$, the forward propagation computation within this module is formulated as follows:

(7) r∼Bernoulli(1−p),

where p denotes the specified dropout probability; and r represents a binary mask vector with the same dimensions as the feature vector.

(8) $$\tilde{Y}=\frac{1}{1-p}(r*Y),$$

where $\tilde{Y}$ denotes the new output activation values following the dropout operation; * represents element-wise multiplication; and $\frac{1}{1-p}$ serves as a dynamic scaling factor utilized during training to scale up the weights of the retained neurons, thereby ensuring that the expected value of the entire feature vector remains constant before and after dropout.

#### 3.2.5. Classification Head and Output Layer

The classification head serves as the output module bridging the feature extraction network and the final class predictions. Typically comprising an FC layer, it maps high-dimensional feature vectors into the category space. Specifically, it projects the 1024-dimensional feature vector that was previously compressed via GAP into logits for the eight classes, and subsequently converts these logits into a normalized probability distribution using the softmax function.

In this study, a single-layer FC classification head is introduced following the dropout layer to serve as the final classifier for the end-to-end model. It integrates features across all channels to output confidence scores for each class, thereby mapping the semantic features to specific fruit shape categories.

The workflow of the classification head and output layer comprises two primary stages: linear feature projection and probability normalization. Initially, the FC layer processes the refined 1D feature vector from the dropout layer by applying a linear transformation via its internal weight matrix. This operation projects the feature dimensions into logits corresponding to the number of chili pepper classes, as formulated in Equation (9):

(9) $$z=W\hat{x}+b,$$

where $\hat{x}$ denotes the input feature vector regularized by the dropout layer; W represents the learnable weight matrix of the FC layer; b indicates the bias vector of the FC layer; and z denotes the output unnormalized class logits.

Subsequently, to transform the logits into a statistically valid probability distribution, a softmax activation function is applied at the network output, as formulated in Equation (10):

(10) $$P_{i}=\frac{exp\left(z_{i}\right)}{\sum_{j=1}^{K}exp\left(z_{j}\right)},$$

where K denotes the total number of chili pepper fruit shape categories; $z_{i}$ represents the unnormalized score corresponding to the *i*-th category within the linear transformation output vector; exp(.) denotes the exponential function with the natural base *e*; and $P_{i}$ indicates the final predicted probability output by the network.

#### 3.2.6. Loss Function: Cross-Entropy with Label Smoothing

Cross-entropy loss with LS is a modified supervised learning objective function that incorporates a regularization penalty. By applying uniform probability softening to traditional one-hot encoded ground truth labels, this mechanism mitigates the over-penalization of incorrect classes and prevents the model from developing overconfidence in the correct class during the later stages of training.

In this study, the loss function quantifies the discrepancy between the predicted fruit shapes and the ground truth phenotypes, thereby guiding the iterative update of model weights toward optimal generalization performance. The resulting cross-entropy loss function with LS is formulated as follows:

(11) $$L_{LS-CE}=-\left(0.9+\frac{0.1}{8}\right)\log\left(P_{target}\right)-\sum_{i\neq target}^{8}\left(\frac{0.1}{8}\right)\log\left(P_{i}\right),$$

where $L_{LS-CE}$ denotes the final label-smoothing cross-entropy loss value utilized for neural network backpropagation computation; target represents the ground-truth fruit shape category index corresponding to the current input chili pepper image; $P_{target}$ indicates the predicted probability of the current chili pepper image belonging to its ground-truth fruit shape category; and $P_{i}$ denotes the predicted probability of the current chili pepper image belonging to the *i*-th non-ground-truth fruit shape category.

#### 3.2.7. Weight Decay

WD, mathematically referred to as L2 regularization, is a global regularization technique applied directly to the network parameters. By constraining the magnitude of the weights, this mechanism prevents the model from overfitting to specific noise within the training set, thereby enhancing overall generalization performance.

In this study, this mechanism is implemented as a core constraint operator within the SGD optimizer. Operating in conjunction with dropout and LS, it enhances the model’s robustness against unseen fruit shape samples and maintains smooth decision boundaries. The total objective loss function incorporating WD is formulated in Equation (12):

(12) $$L_{total}=L_{LS-CE}+\frac{\lambda }{2}\sum_{l}{\left\|w_{l}\right\|}_{2}^{2},$$

where $L_{total}$ denotes the total objective loss value employed for backpropagation optimization during the training phase; $L_{LS-CE}$ represents the cross-entropy loss with LS derived in the preceding section; λ indicates the WD coefficient; l denotes the *l*-th layer within the deep neural network; $w_{l}$ represents the weight matrix of the *l*-th layer; and ${\left\|w_{l}\right\|}_{2}^{2}$ indicates the squared L2 norm of the weights.

Integrating the SGD optimizer, the corresponding weight update rule is formulated as follows:

(13) $$w_{t+1}=\left(1-\eta \lambda \right)w_{t}-\eta \nabla_{w}L_{LS-CE}\left(w_{t}\right),$$

where $w_{t}$ denotes the current network weights prior to the *t*-th optimization iteration; $w_{t+1}$ represents the updated network weights following the *t*+1-th iteration; η indicates the learning rate of the model; $\nabla_{w}L_{LS-CE}\left(w_{t}\right)$ denotes the original partial derivative of the cross-entropy loss with LS with respect to the current weights; and $\left(1-\eta \lambda \right)$ represents the WD factor.

### 3.3. Comprehensive Evaluation Methods for Model Performance

When developing a chili pepper fruit shape classification system for real-world agricultural scenarios, a single accuracy metric is insufficient to comprehensively evaluate the applicability of a model. Furthermore, hardware resource constraints associated with deployment on mobile or edge devices need to be considered. In this study, to determine the optimal model, a weighted scoring method based on Min-Max normalization is employed to comprehensively evaluate mainstream deep learning architectures, including ResNet-50 [42], VGG-16 [43], EfficientNet-B0 [44], and DenseNet-121, across multiple metrics: test set accuracy, F1 score, parameter count, model size, inference time, and training duration, as formulated in Equation (14):

(14) $$S_{comp}=W_{acc}\cdot \left(\frac{Acc_{norm}+F1_{norm}}{2}\right)+W_{eff}\cdot \left(\frac{Param_{norm}+Size_{norm}+InfTime_{norm}+TrainTime_{norm}}{4}\right),$$

where $S_{comp}$ denotes the final comprehensive evaluation score of the model; $Acc_{norm}$ and $F1_{norm}$ represent the normalized test set accuracy and F1-score, respectively; $Param_{norm}$, $Size_{norm}$, $InfTime_{norm}$, and $TrainTime_{norm}$ indicate the normalized scores for parameter count, model size, single-image inference time, and training duration, respectively; $W_{acc}$ denotes the weight coefficient for the accuracy dimension; and $W_{eff}$ represents the weight coefficient for the efficiency dimension.

### 3.4. Comparative Experimental Design and Baseline Models

To evaluate the comprehensive performance of the proposed DenseNet-121 model incorporating the CBAM in the chili pepper fruit shape classification task, this study selected four representative baseline models from the fields of agricultural computer vision and image regression for comparative experiments. The specific models are as follows: MobileNetV3 [45], a representative lightweight CNN that combines hardware-aware network architecture search with the Squeeze-and-Excitation (SE) attention mechanism; ShuffleNetV2 [46], an efficient lightweight CNN incorporating the channel shuffle mechanism; Swin Transformer Tiny (Swin-Tiny) [47], a hierarchical pure vision architecture based on the shifted window mechanism; MobileViT [48], a lightweight hybrid architecture integrating CNN and Transformer paradigms.

During training, all baseline models employed data augmentation strategies, input resolutions, and hardware configurations identical to those of the proposed model.

## 4. Results and Analysis

### 4.1. Evaluation Results of Baseline Models

To evaluate the comprehensive performance of model architectures in the chili pepper fruit shape classification task, this study conducted a comparative analysis of four deep learning architectures: DenseNet-121, EfficientNet-B0, ResNet-50, and VGG-16. The corresponding experimental results are presented in Table 4.

As demonstrated in Table 4, DenseNet-121 yields the highest classification accuracy metrics on the test set, recording a test accuracy of 86.90% and an F1-score of 86.74%, which surpass those of the second-best model, EfficientNet-B0, by 3.71% and 3.66%, respectively.

Regarding hardware parameters, DenseNet-121 has 6.96 M parameters and a storage requirement of 27.15 MB, the second lowest among the four evaluated models. Although its parameter count slightly exceeds that of EfficientNet-B0 (4.02 M, 15.62 MB), which is optimized for mobile deployment, it remains substantially lower than those of the conventional deep network ResNet-50 (23.52 M, 90.05 MB) and the earlier heavyweight architecture VGG-16 (134.29 M, 512.30 MB), indicating efficient parameter usage.

Regarding time parameters, DenseNet-121 has a total training time of 5.1 min and a single-image forward inference time of 7.92 ms, both of which are the highest among the four models. This is primarily attributed to the frequent cross-layer concatenation operations within the dense blocks, which increase the GPU memory bandwidth overhead during underlying tensor interactions. Nevertheless, an inference time of 7.92 ms theoretically meets the sorting requirements of most agricultural scenarios.

Based on the comprehensive evaluation method described in Section 3.3, DenseNet-121 achieved the highest final comprehensive score of 0.847. This quantitative score demonstrates that DenseNet-121 achieves an optimal trade-off between classification accuracy and model size, at the cost of a limited increase in time overhead. Consequently, DenseNet-121 was selected as the foundational architecture for all subsequent experiments in this study.

### 4.2. Effects of Optimizers on Model Performance

To evaluate the impact of different optimizers on the classification performance of the proposed method, a comparative analysis was conducted by optimizing the network parameters using three distinct optimizers: SGD with momentum [49], Adam [50], and AdamW [51]. The corresponding experimental results are presented in Table 5.

As demonstrated in Table 5, the SGD with momentum optimizer achieves the highest performance across three evaluation metrics: recall, F1-score, and accuracy, recording 89.40%, 89.34%, and 89.52%, respectively. Compared to the adaptive learning rate optimizers AdamW (87.77% accuracy) and Adam (85.37% accuracy), it yields accuracy improvements of 1.75% and 4.15%, respectively. Furthermore, it requires the shortest training time of 3.59 min among the evaluated optimizers. Consequently, SGD with momentum is selected as the optimizer for this study.

### 4.3. Effects of Initial Learning Rate Configurations on Model Performance

**Figure 4:**
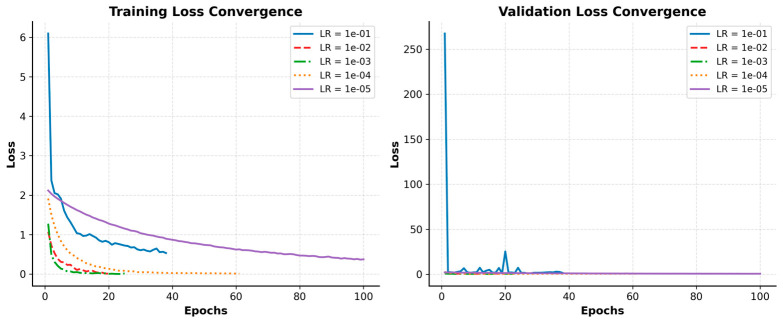
Training and validation loss convergence curves under different initial learning rates (LR).

To evaluate the impact of various initial learning rates on the classification performance of the proposed method, the initial learning rate was configured to five distinct values: 1 × 10^−1^, 1 × 10^−2^, 1 × 10^−3^, 1 × 10^−4^, 1 × 10^−5^. The corresponding loss curves for these configurations are presented in Figure 4.

As illustrated in Figure 4, at an initial learning rate of 1 × 10^−5^, the loss curve exhibits a gradual and prolonged decline, indicating the slowest convergence rate, with the loss value remaining at a relatively high level. Conversely, while initial learning rates of 1 × 10^−1^ and 1 × 10^−2^ induce more rapid convergence, pronounced oscillations occur during the process, preventing effective feature learning and parameter stabilization. An initial learning rate of 1 × 10^−3^, however, facilitates both rapid and smooth convergence. The corresponding experimental results are presented in Table 6.

As demonstrated in Table 6, at an initial learning rate of 1 × 10^−3^, the proposed method achieves the highest test performance, recording a recall of 88.72%, an F1-score of 88.64%, and an accuracy of 88.86%. Compared to the second-best learning rate configuration (1 × 10^−4^), it yields improvements of 3.47%, 3.54%, and 3.49% across these metrics, respectively. These results indicate that 1 × 10^−3^ serves as the optimal initial learning rate for this dataset. Consequently, the initial learning rate is fixed at 1 × 10^−3^ for all subsequent experiments in this study.

### 4.4. Effects of Regularization on Model Performance

To evaluate the impact of various regularization strategies on the classification performance of the proposed method, nine configurations, designated as Exp0 through Exp8, were designed for comparison. These configurations are outlined in Table 7. These regularization strategies were evaluated on the baseline DenseNet-121 architecture prior to the integration of the attention module.

As demonstrated in Table 7, among the nine regularization configurations, Exp8 (LS = 0.1, Dropout = 0.3, WD = 1 × 10^−4^) achieves an optimal balance between classification accuracy and robustness in complex environments. Specifically, on the standard clean test set, Exp8 yields an accuracy of 88.43% and an F1-score of 88.11%, representing improvements of 1.53% and 1.41%, respectively, over the unregularized baseline model (Exp0). Additionally, its overfit gap is narrowed to 12.63%, suppressing the deep network’s overfitting to background noise in the training set.

Exp4, which employs an aggressive structural deactivation strategy (Dropout = 0.5, WD = 1 × 10^−4^), achieves the highest accuracy (89.74%) and F1-score (89.67%) among all configurations. However, during the robustness generalization evaluation, the F1-score of Exp4 degrades substantially to 76.92% under conditions of severe visual perturbation (Severe Robust). In contrast, Exp8 constructs a multidimensional joint regularization framework comprising “softened classification boundaries (LS) + mild structural deactivation to prevent feature co-adaptation (Dropout) + moderate parameter shrinkage (WD).” This mechanism not only yields the highest score of 89.53% on the mild perturbation set (Mild Robust) but also maintains a performance of 81.29% under severe perturbation. In summary, to balance classification accuracy and robustness in agricultural deployments, the composite regularization strategy of Exp8 is selected for all subsequent experiments.

### 4.5. Effects of Attention Mechanisms on Model Performance

To evaluate the impact of various attention modules on the classification performance of the proposed model, six distinct attention modules, namely CBAM, Squeeze-and-Excitation Module (SE) [52], CAM, SAM, Efficient Channel Attention Module (ECA) [53], and Coordinate Attention Module (CA) [54], were systematically integrated into the DenseNet-121 backbone network. The corresponding experimental results are presented in Table 8.

Table 8 shows that among the six attention modules, CBAM achieves the best performance on the test set. The CBAM-equipped model reached an accuracy of 89.74%, a precision of 90.09%, a recall of 89.60%, and an F1-score of 89.53%. These results represent improvements of 3.37%, 3.28%, 3.44%, and 3.28% over the baseline without attention, and outpace the second-best module (ECA) by 0.47%, 0.17%, 0.16%, and 0.22%, respectively. Unlike the other five modules that process only a single feature dimension, CBAM extracts both spatial and channel features from the chili pepper images. Therefore, CBAM was selected as the attention module for the DenseNet-121 backbone.

### 4.6. Ablation Study

To evaluate the effectiveness of the attention module and the composite regularization strategy for chili pepper fruit shape classification, four network variants were designed. The specific configurations and experimental results are summarized in Table 9.

Table 9 shows that the proposed method achieved a precision of 90.09%, a recall of 89.60%, an F1-score of 89.53%, and an accuracy of 89.74% on the test set. These results represent improvements of 1.63%, 1.75%, 1.68%, and 1.75%, respectively, over the baseline model (Exp1_Baseline), which obtained 88.46%, 87.85%, 87.85%, and 87.99% across the corresponding metrics. These findings demonstrate that the proposed method achieves better classification performance on this dataset compared to the baseline.

### 4.7. Confusion Matrix and Fine-Grained Feature Analysis of Fruit Shape Classes

**Figure 5:**
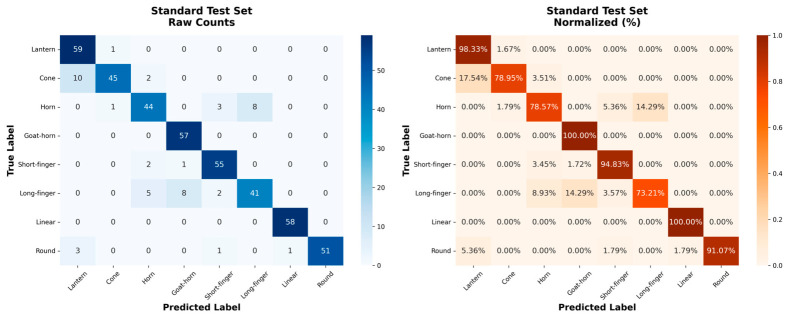
Confusion matrices of the proposed model evaluated on the standard test set. The **left panel** displays the raw classification counts, while the **right panel** illustrates the normalized values representing the recall rate (%) for each morphological category.

To evaluate the fine-grained discriminative and classification capabilities of the proposed model, a confusion matrix and detailed classification metrics were computed on the test set (Figure 5 and Table 10). The results show that the model reliably distinguishes morphologically distinct classes. The Linear class achieved the best overall performance (100% recall, 98.31% precision), and the Goat-horn class also reached 100% recall. Notably, the Round class was the only category to achieve 100% precision. These results suggest that the CBAM module effectively extracts features from both highly elongated shapes and smooth, curved boundaries.

However, confusion persists among certain morphologically similar classes. A total of 48 misclassifications occurred in the test set, primarily involving the following cases: Long-finger vs. Goat-horn and Horn. The Long-finger class had the lowest recall (73.21%). Out of 15 misclassified Long-finger samples, 8 were predicted as Goat-horn and 5 as Horn. Cone to Lantern (Unidirectional). Ten of the 12 misclassified Cone samples were predicted as Lantern, dropping the recall for the Cone class to 78.95%. Horn to Long-finger. Eight Horn samples were misclassified as Long-finger, resulting in a lower precision (83.02%) for the Horn class.

These misclassifications predominantly occur at the fuzzy boundaries between fruit shapes. Overall, the observed confusion aligns with the inherent principles of plant morphology.

### 4.8. Comparison with State-of-the-Art Models

To evaluate the classification performance of the proposed model, it was compared against four mainstream benchmark networks: ShuffleNetV2, MobileNetV3, Swin-Tiny, and MobileViT-S. The results are summarized in Table 11.

Table 10 shows that the proposed model achieved a precision of 90.09%, a recall of 89.60%, an F1-score of 89.53%, and an accuracy of 89.74%, outperforming the four mainstream benchmark networks in feature recognition capabilities. Specifically, compared to the ultra-lightweight classic architecture ShuffleNetV2t, the proposed method yields improvements of 26.56%, 30.38%, 32.09%, and 30.35% in precision, recall, F1-score, and accuracy, respectively. when evaluated against the mobile-optimized MobileNetV3, it achieves increases of 9.80%, 9.15%, 9.57%, and 9.17% across the same metrics. Similarly, compared to the pure vision Transformer architecture Swin-Tiny, the proposed method provides gains of 5.34%, 5.45%, 5.56%, and 5.46% in precision, recall, F1-score, and accuracy, respectively. Furthermore, against the lightweight CNN-Transformer hybrid MobileViT-S, it secures performance margins of 7.44%, 7.22%, 7.29%, and 7.21% across the same metrics. These results indicate that the proposed method offers a distinct advantage over current mainstream benchmark networks in feature extraction and target discrimination for chili pepper fruit shape classification.

### 4.9. Visualization of Feature Maps

**Figure 6:**
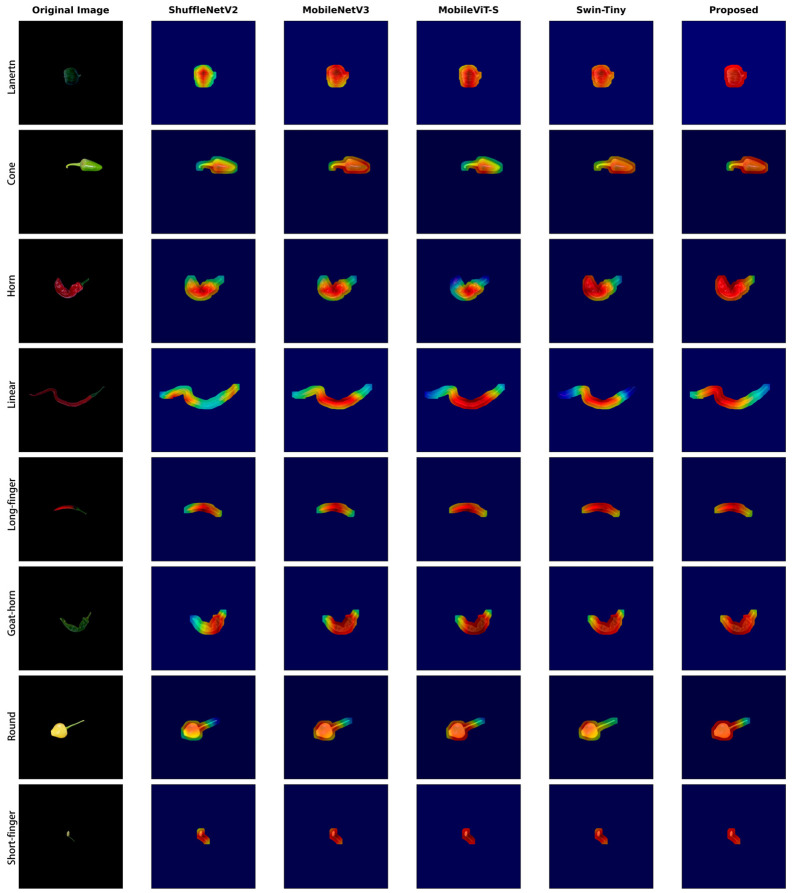
Grad-CAM heatmaps of the five evaluated models across the eight shape classes. Regions with deeper red hues indicate a higher positive contribution of those pixels to the final classification decision.

Currently, the automated classification of chili pepper fruit shapes lacks sufficient interpretability. To investigate the key features learned by the model, Gradient-weighted Class Activation Mapping (Grad-CAM) [55] was employed. The generated heatmaps were superimposed onto the original images for feature visualization. During forward propagation, the network outputs feature maps and prediction scores at the deep layers. Backpropagation is then utilized to obtain the gradients corresponding to the target class, which are used to compute the global importance weights for different feature channels. Finally, a weighted sum of these channel features is computed and passed through a ReLU activation function to eliminate negative interference, thereby generating the Grad-CAM heatmaps (Figure 6).

As shown in Figure 6, the high-activation regions of the proposed model focus on the key morphological features of the chili peppers. Conversely, the activation maps for ShuffleNetV2, MobileNetV3, Swin-Tiny, and MobileViT-S are dispersed. Their high-weight regions are scattered across the center of the fruits, missing the local details that determine shape differences. This precise localization is attributed to the dual spatial-channel attention mechanism of CBAM, which targets essential feature information, alongside the joint regularization strategy. The regularization prevents feature co-adaptation and effectively suppresses interference from localized noise and complex backgrounds, allowing the model to consistently focus on pixels that positively drive the classification. Therefore, the model’s feature extraction process aligns with visual fruit shape assessment, providing improved interpretability.

## 5. Discussion

While recent state-of-the-art lightweight networks have demonstrated remarkable efficiency on standard macro-level datasets, automated chili pepper shape recognition is inherently a fine-grained visual classification task. It heavily relies on preserving localized morphological variations and underlying geometric contours. As demonstrated in our comparative analysis against EfficientNet-B0 (Table 4) and MobileNetV3 (Table 11), extremely lightweight or aggressive downsampling architectures may inadvertently discard crucial low-level edge features. The dense connectivity pattern of DenseNet-121 mitigates this by facilitating deep feature reuse, explicitly passing low-level contour information across layers. When integrated with CBAM, this architecture proved highly effective in discriminating subtle inter-class similarities, providing a theoretically sound and empirically validated optimal trade-off between fine-grained accuracy and computational efficiency for this specific agricultural application.

It is also necessary to define the intended operational environment of the proposed model. Modern post-harvest optical sorting equipment typically utilizes controlled inspection chambers with standardized illumination and uniform conveyor backgrounds to physically isolate the targets. Therefore, the preprocessed images utilized in this dataset closely reflect the actual visual inputs encountered in these industrial systems, where complex external backgrounds are mechanically eliminated prior to imaging.

While the proposed DenseNet-121 + CBAM framework demonstrates effective classification capabilities, several limitations must be objectively acknowledged to guide future research. First, the current dataset is restricted to eight common pepper morphological categories. It does not yet encompass malformed fruits, rare hybrid varieties, or fruits with severe surface defects, which are occasionally encountered in real-world agricultural sorting. Second, although the model shows robustness against simulated environmental noise in single-fruit scenarios, its performance when dealing with highly complex backgrounds, specifically scenarios involving multiple overlapping peppers or severe partial occlusions, remains unverified. Therefore, future work will focus on expanding the dataset to include a wider diversity of pepper phenotypes and malformations. We plan to integrate this fine-grained classifier with advanced instance segmentation networks to effectively address overlapping fruits and occlusions in fully unconstrained end-to-end sorting environments. Furthermore, specific algorithmic and deployment improvements will be explored. We intend to investigate the integration of Transformer modules to improve long-distance feature modeling and capture more complex global structural dependencies. Finally, to optimize the framework for actual agricultural production lines, we will explore model quantization and compression techniques to further adapt the algorithm for real-time execution on resource-constrained edge devices, such as the NVIDIA Jetson platform.

## 6. Conclusions

To address the high morphological similarity among chili pepper shape categories and their susceptibility to environmental interference, this study developed an accurate and robust classification model. The model integrates a CBAM, an LS cross-entropy loss function, and a joint regularization strategy into the DenseNet-121 backbone. Its effectiveness was systematically evaluated, leading to the following conclusions:

1. To address the difficulty of extracting fine-grained shape features under environmental interference, the CBAM module was introduced to capture key regions such as fruit contours and tip curvature. Grad-CAM visualizations show that the proposed model localizes the morphological boundaries of the chili peppers, avoiding the background dispersion and feature fragmentation observed in certain lightweight networks and the Swin-Tiny model;
2. To address the susceptibility of deep convolutional networks to local optima and hard-label overconfidence in fine-grained tasks, cross-entropy loss with LS (LS = 0.1) was introduced to enhance the robustness of the decision boundaries. Quantitative results show that this strategy, combined with joint regularization (Dropout = 0.3, WD = 1 × 10^−4^) and an initial learning rate of 1 × 10^−3^, mitigates model overfitting to background noise in the training set;
3. The proposed model and four benchmark networks were trained and evaluated on the constructed chili pepper shape dataset. The results show that the proposed model achieved a precision of 90.09%, a recall of 89.60%, an F1-score of 89.53%, and an overall accuracy of 89.74%. Under mild image degradation typical of real-world production environments, the model maintained a robust F1-score of 87.24%. Furthermore, with 7.09 M parameters and a single-frame inference time of 7.35 ms, the model satisfies the low memory footprint and high-throughput requirements for real-time sorting on embedded devices.

Overall, the evaluation indicates that the proposed model achieves a balance among feature extraction, prediction accuracy, and computational efficiency, providing the technical foundation for large-scale deployment in agricultural sorting lines.

## Figures and Tables

**Table 1:** Chili Pepper Fruit Shape Image Dataset.

Fruit Shape	Original Images	Augmentation Factor	Augmented Images	Training Samples	Validation Samples	Test Samples
Lantern	152	2.63	400	281	59	60
Cone	130	3.08	400	282	61	57
Horn	272	1.47	400	287	57	56
Goat-horn	254	1.57	400	285	59	56
Short-finger	159	2.52	400	283	61	56
Long-finger	373	1.07	400	282	60	58
Linear	217	1.84	400	283	60	57
Round	75	5.33	400	284	58	58

**Table 2:** Hardware and Software Specifications for Model Training.

Component	Specification
Operating system	AutoDL (ubuntu22.04)
CPU	25 vCPU Intel(R) Xeon(R) Platinum 8470Q
GPU	RTX 5090 (32 GB)
CUDA version	12.8
Python version	3.12
PyTorch version	2.8.0

**Table 3:** Key Hyperparameter Configurations for DenseNet-121 Model Training.

Hyperparameter	Value	Description
Epochs (Max)	100	Maximum number of training epochs
Batch_Size	32	Mini-batch size for forward propagation
Input_Size	224 × 224	Standardized spatial resolution of input images
Optimizer	SGD	Stochastic Gradient Descent with momentum
Initial_Learning_Rate	1 × 10^−3^	Initial learning rate for network parameter updates
Learning_Rate_Scheduler	CosineAnnealingLR	Cosine annealing schedule (Tmax = 100)
Weight_Decay	1 × 10^−4^	L2 regularization penalty to mitigate overfitting
Momentum	0.9	Momentum factor to accelerate SGD and escape local minima
Patience	15	Early stopping patience based on validation F1-score
Dropout_Rate	0.3	Dropout probability applied after global average pooling
Loss_Function	CrossEntropyLoss	Standard classification loss function
Label_Smoothing	0.1	Smoothing factor to soften hard one-hot target distributions
Attention_Module	CBAM	Dual spatial and channel attention mechanism

**Table 4:** Experimental Results of Various Models.

Model	Test Acc (%)	Test F1 (%)	Parameters (M)	Model Size (MB)	Inference Time (ms)	Training Time (mins)	Comprehensive_Score
DenseNet-121	86.90	86.74	6.96	27.15	7.92	5.1	0.847
EfficientNet-B0	83.19	83.08	4.02	15.62	3.4	1.5	0.687
ResNet-50	81.44	81.42	23.52	90.05	2.49	1.6	0.539
VGG-16	78.17	77.65	134.29	512.3	1.38	3.8	0.102

**Table 5:** Effects of Optimizers on Model Performance.

Optimizer	Test_Recall (%)	Test_F1 (%)	Test_Accuracy (%)	Training_Time (min)
SGD_Momentum	89.40	89.34	89.52	3.59
Adam	85.27	85.21	85.37	4.29
AdamW	87.63	87.64	87.77	4.54

**Table 6:** Effects of Initial Learning Rate Configurations on Model Performance.

Learning_Rate	Test_Recall (%)	Test_F1 (%)	Test_Accuracy (%)
1 × 10^−1^	63.29	62.63	63.54
1 × 10^−2^	83.28	83.32	83.41
1 × 10^−3^	88.72	88.64	88.86
1 × 10^−4^	85.25	85.10	85.37
1 × 10^−5^	82.38	82.13	82.53

**Table 7:** Effects of Regularization on Model Performance.

Experiment	LS	Dropout	WD	Overfit_ Gap (%)	Test_ Accuracy (%)	Test_ F1 (%)	Mild_ Robust_F1 (%)	Severe_ Robust_F1 (%)
Exp0	0	0	0	13.39	86.90	86.70	85.47	83.25
Exp1	0	0	1 × 10^−4^	13.75	86.90	86.55	85.78	78.61
Exp2	0.1	0	1 × 10^−4^	13.68	87.77	87.54	87.53	78.05
Exp3	0	0.3	1 × 10^−4^	14.14	87.12	86.88	86.80	82.46
Exp4	0	0.5	1 × 10^−4^	13.46	89.74	89.67	85.64	76.92
Exp5	0	0	5 × 10^−4^	13.32	87.99	87.76	87.80	81.64
Exp6	0	0	1 × 10^−4^	11.37	87.55	87.42	88.13	83.14
Exp7	0.1	0.3	5 × 10^−4^	12.12	87.99	87.78	86.36	76.11
Exp8	0.1	0.3	1 × 10^−4^	12.63	88.43	88.11	89.53	81.29

**Table 8:** Effects of Attention Mechanisms on Model Performance.

Attention_Module	Test_Precision (%)	Test_Recall (%)	Test_F1 (%)	Test_Accuracy (%)
Baseline	86.72	86.32	86.10	86.46
CBAM	90.09	89.60	89.53	89.74
SE	89.26	88.72	88.69	88.86
CAM	88.28	87.85	87.77	87.99
SAM	84.40	83.46	83.27	83.62
ECA	89.62	89.43	89.37	89.52
CA	88.74	88.05	88.00	88.21

**Table 9:** Ablation Study Results.

Experiment	Attention	LS	Dropout	Test_ Precision (%)	Test_ Recall (%)	Test_ F1 (%)	Test_ Accuracy (%)
Exp1_Baseline	/	0.0	0.0	88.46	87.85	87.85	87.99
Exp2_ + CBAM	CBAM	0.0	0.0	88.91	88.80	88.78	88.86
Exp3_ + LS	CBAM	0.1	0.0	88.52	88.33	88.24	88.43
Exp4_ + Dropout	CBAM	0.1	0.3	90.09	89.60	89.53	89.74

**Table 10:** Fine-grained classification performance metrics of the optimal model across eight chili pepper shape categories.

Pepper_Shape	Precision (%)	Recall (%)	F1-Score (%)
Lantern	0.82	0.98	0.89
Cone	0.96	0.79	0.87
Horn	0.83	0.79	0.81
Goat_horn	0.86	1.00	0.93
Short_finger	0.90	0.95	0.92
Long_finger	0.84	0.73	0.78
Linear	0.98	1.00	0.99
Round	1.00	0.91	0.95
accuracy	0.90	0.90	0.90
macro_avg	0.90	0.89	0.89
weighted_avg	0.90	0.90	0.89

**Table 11:** Performance comparison of the proposed method and benchmark networks.

Model	Precision (%)	Recall (%)	F1-Score (%)	Accuracy (%)	Time (ms)
ShuffleNetV2	63.53	59.22	57.44	59.39	2.41
MobileNetV3	80.29	80.45	79.96	80.57	2.46
Swin-Tiny	84.75	84.15	83.97	84.28	5.18
MobileViT-S	82.65	82.38	82.24	82.53	4.14
DenseNet-121 (Proposed)	90.09	89.60	89.53	89.74	7.35

## Data Availability

The datasets and source code generated and analyzed during the current study are not publicly available at this moment due to a pending patent application. However, they are available from the corresponding author on reasonable request for academic purposes only.

## Supplementary Materials

The following supporting information can be downloaded at: https://www.mdpi.com/article/10.3390/plants15132103/s1.
