# Supplementary figures and images for: A Deep Learning Model for Chili Pepper Fruit Shape Classification Using DenseNet-121 and CBAM (part 2 of 2)
Source: Plants (Basel). 2026 Jul 7;15(13):2103. doi: 10.3390/plants15132103 (PMC13364266; doi:10.3390/plants15132103)

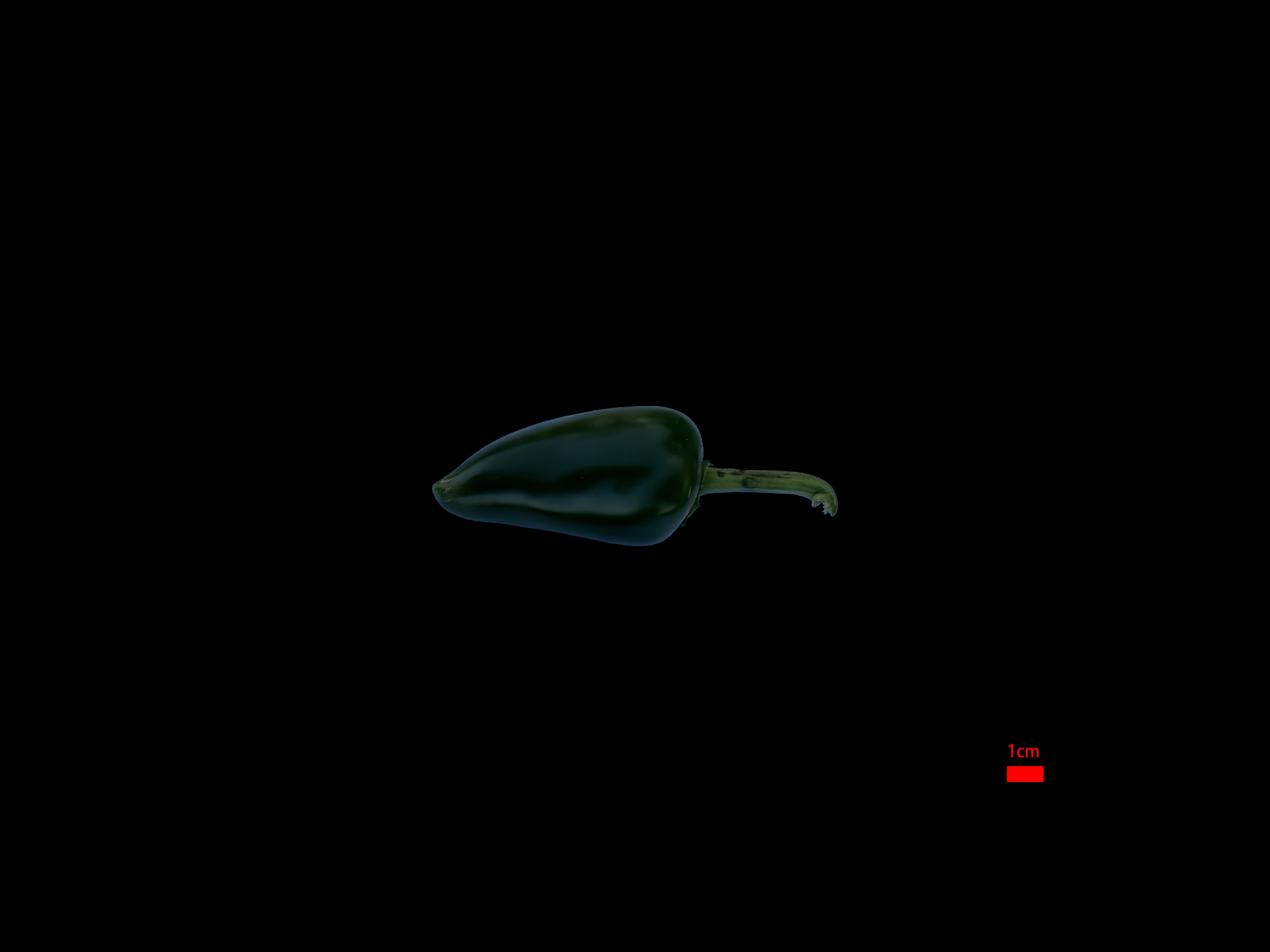

Supplement: Supplementary file 1 [file plants-15-02103-s001.zip › plants-4383327-supplementary/pepper_original_data/cone/34-1.jpg]

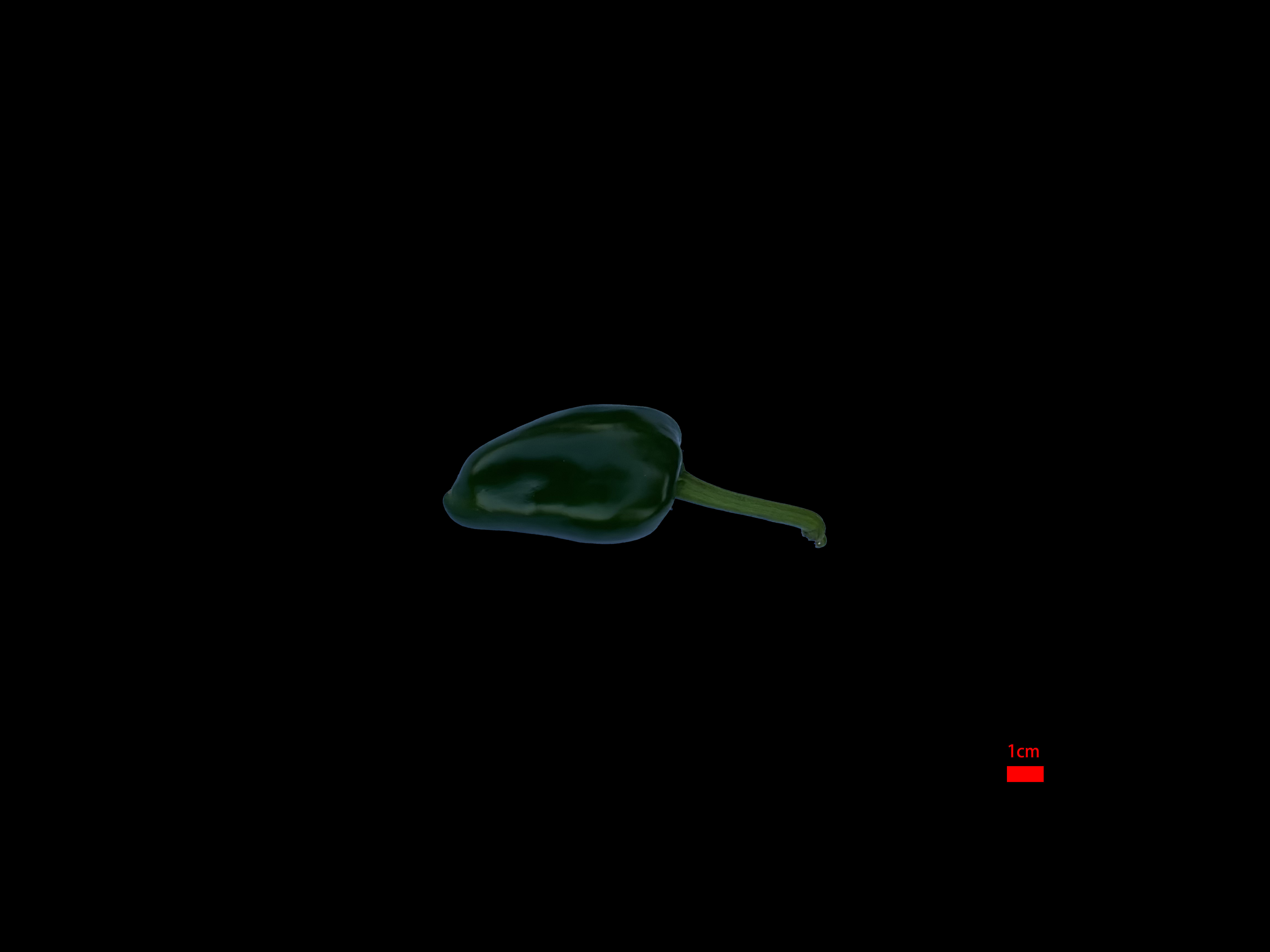

Supplement: Supplementary file 1 [file plants-15-02103-s001.zip › plants-4383327-supplementary/pepper_original_data/cone/34-2.jpg]

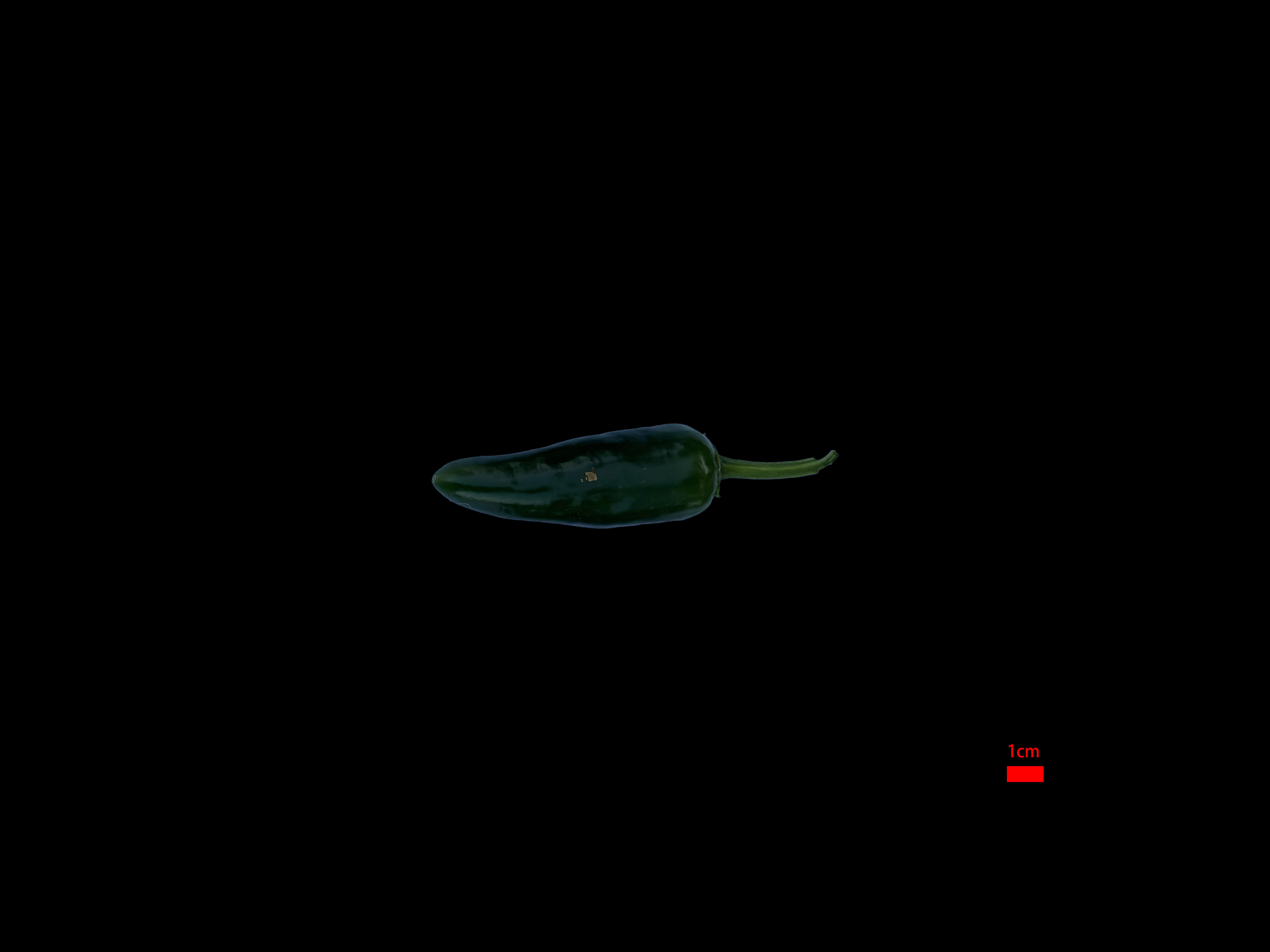

Supplement: Supplementary file 1 [file plants-15-02103-s001.zip › plants-4383327-supplementary/pepper_original_data/cone/34-4.jpg]

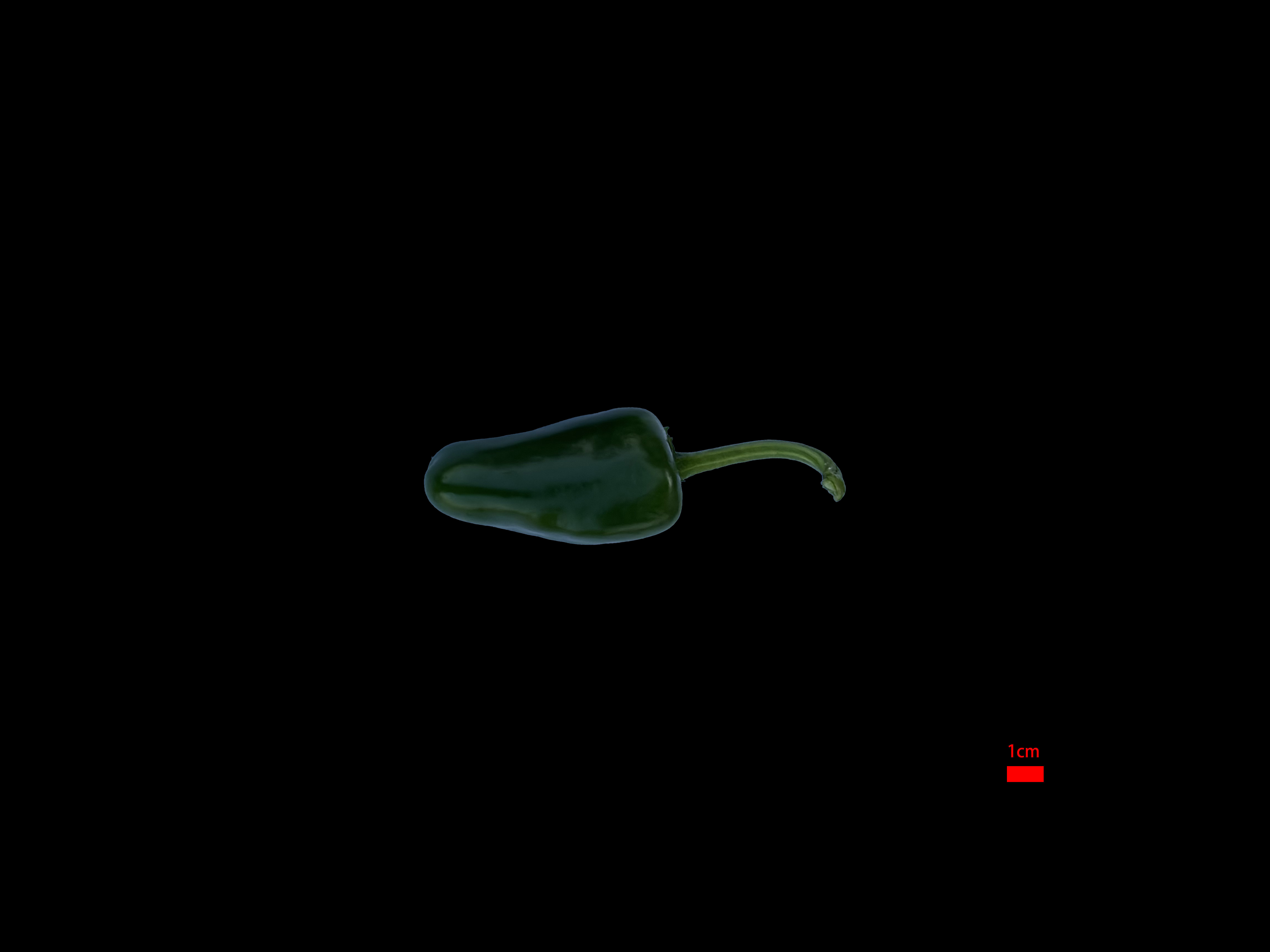

Supplement: Supplementary file 1 [file plants-15-02103-s001.zip › plants-4383327-supplementary/pepper_original_data/cone/34-5.jpg]

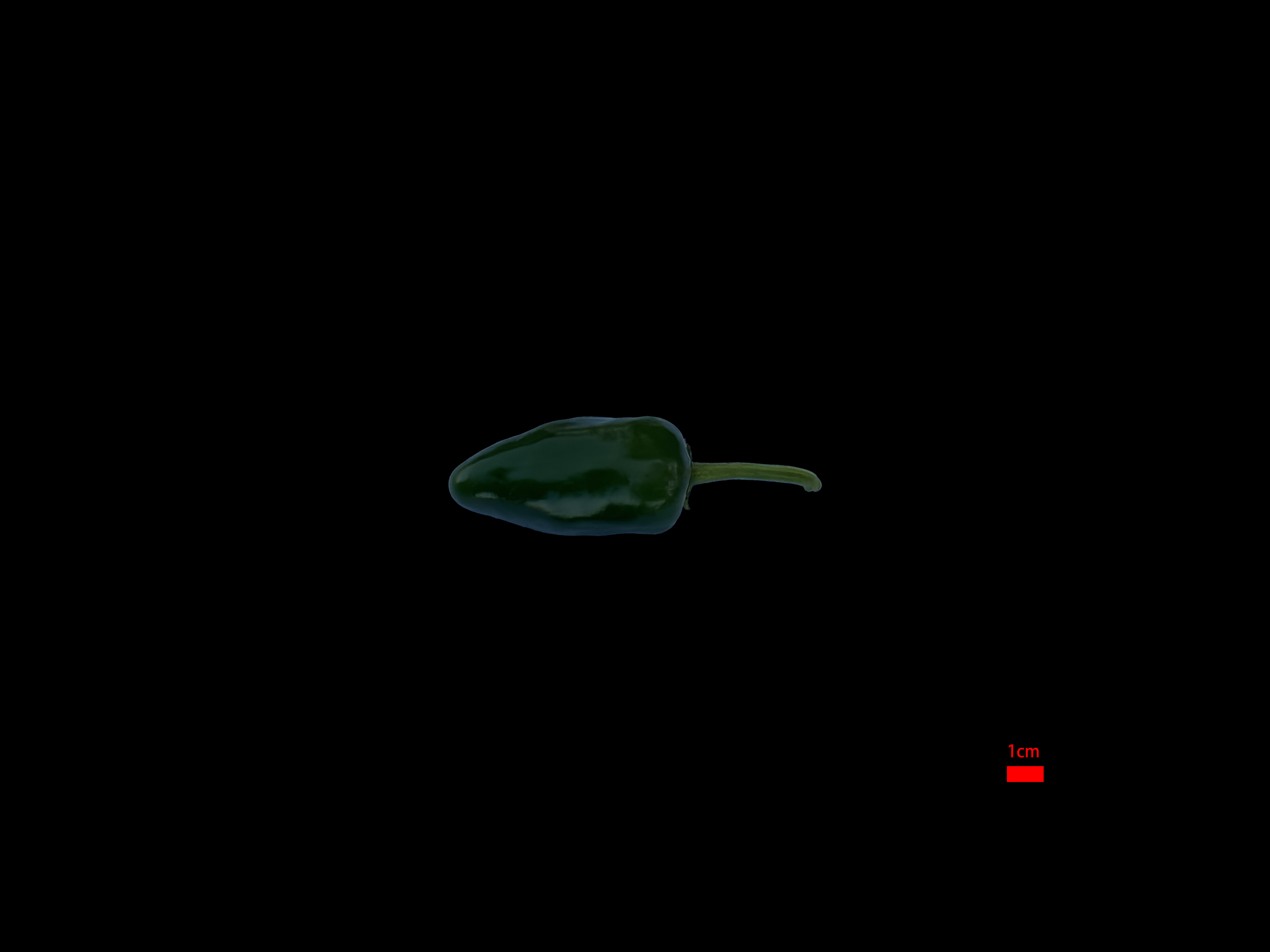

Supplement: Supplementary file 1 [file plants-15-02103-s001.zip › plants-4383327-supplementary/pepper_original_data/cone/34-6.jpg]

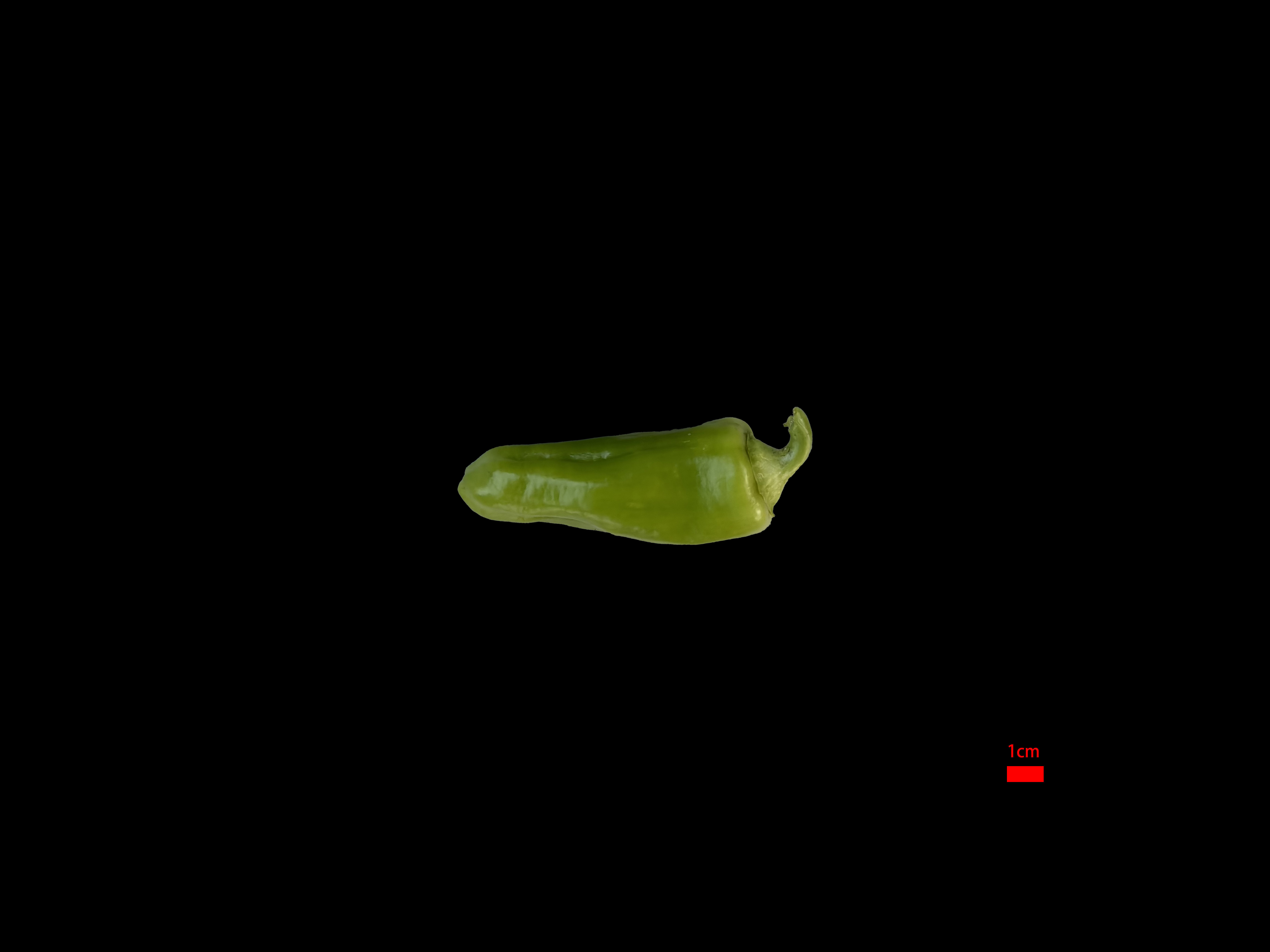

Supplement: Supplementary file 1 [file plants-15-02103-s001.zip › plants-4383327-supplementary/pepper_original_data/cone/41-10.jpg]

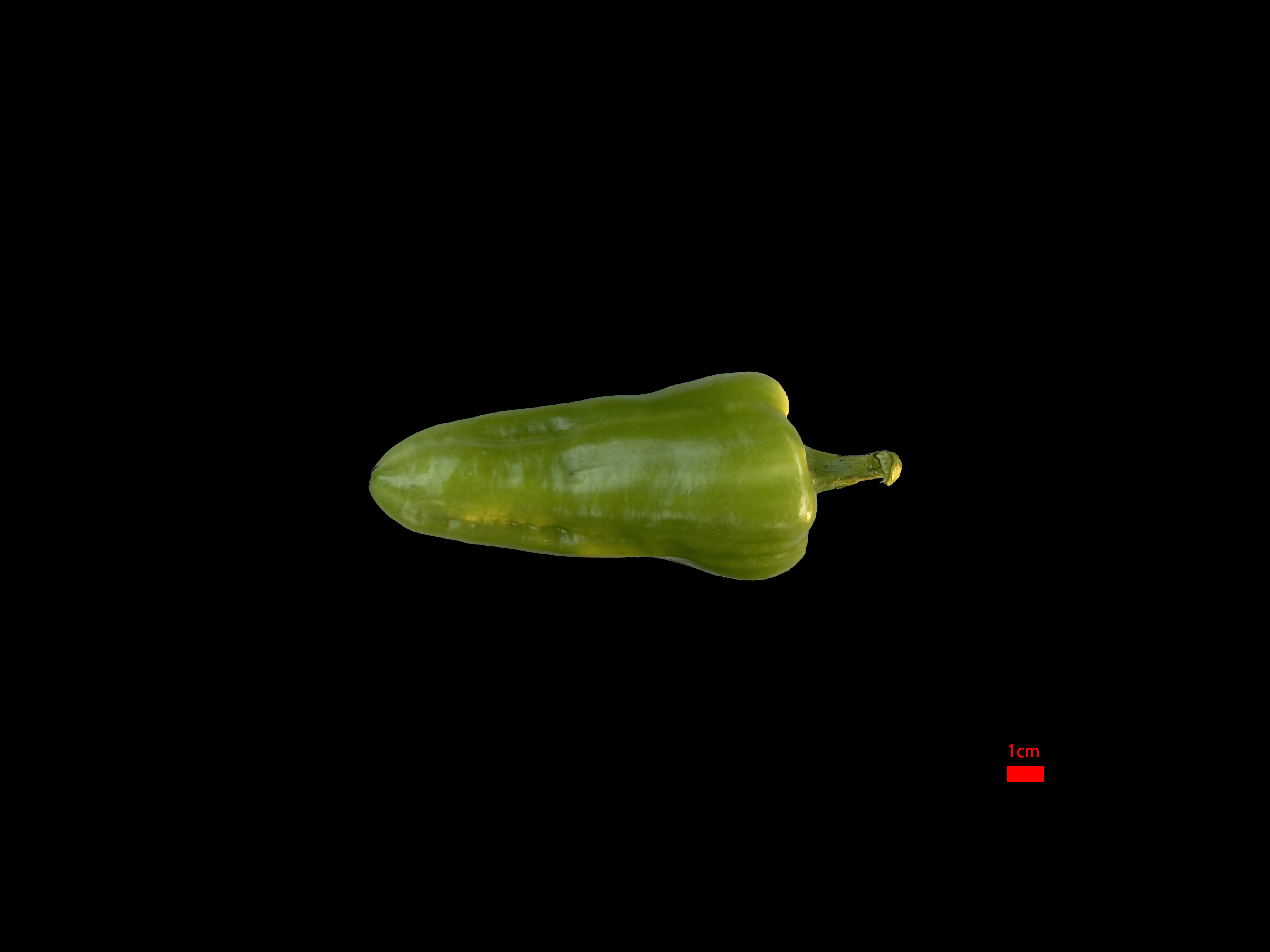

Supplement: Supplementary file 1 [file plants-15-02103-s001.zip › plants-4383327-supplementary/pepper_original_data/cone/41-2.jpg]

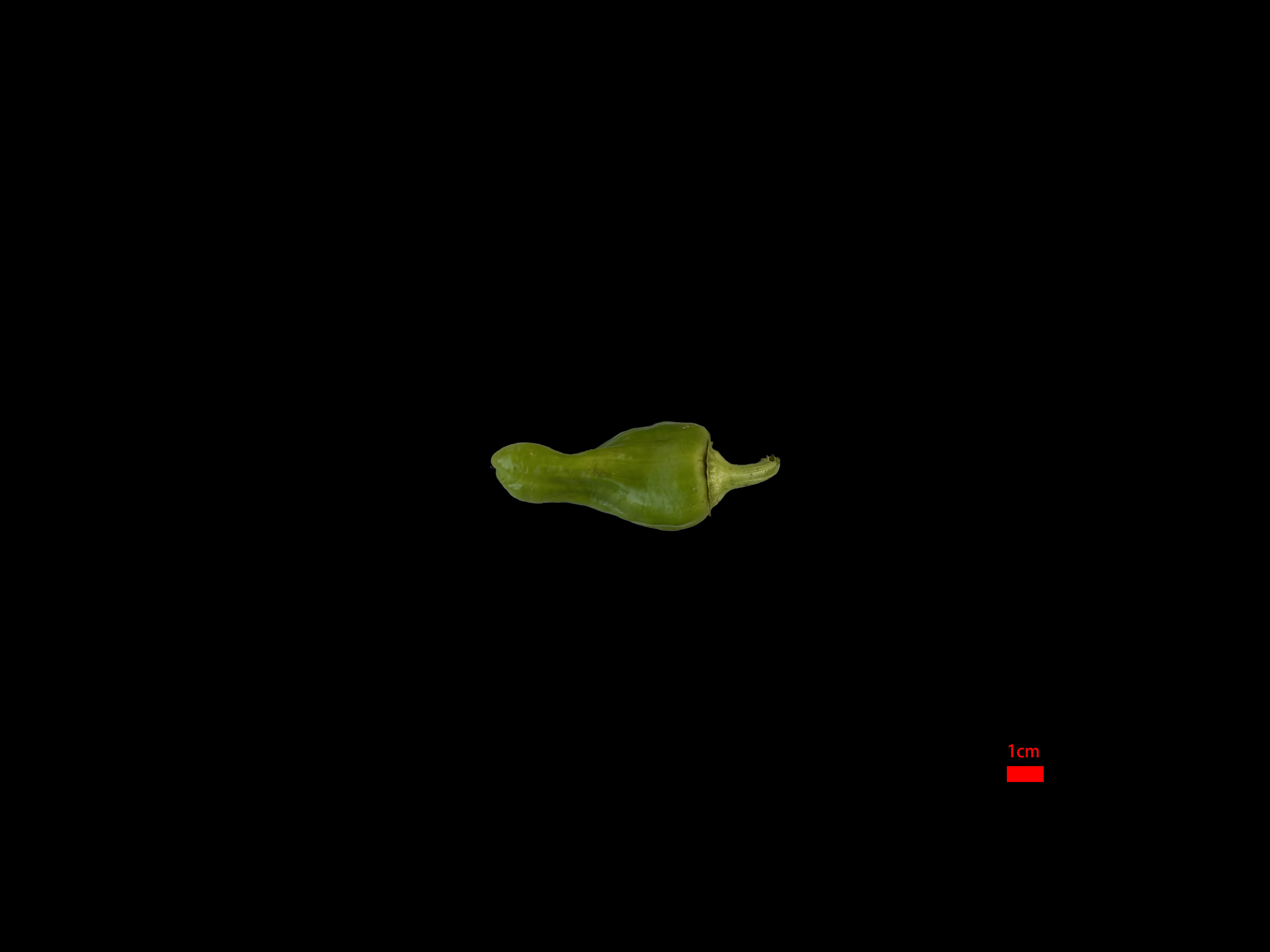

Supplement: Supplementary file 1 [file plants-15-02103-s001.zip › plants-4383327-supplementary/pepper_original_data/cone/41-7.jpg]

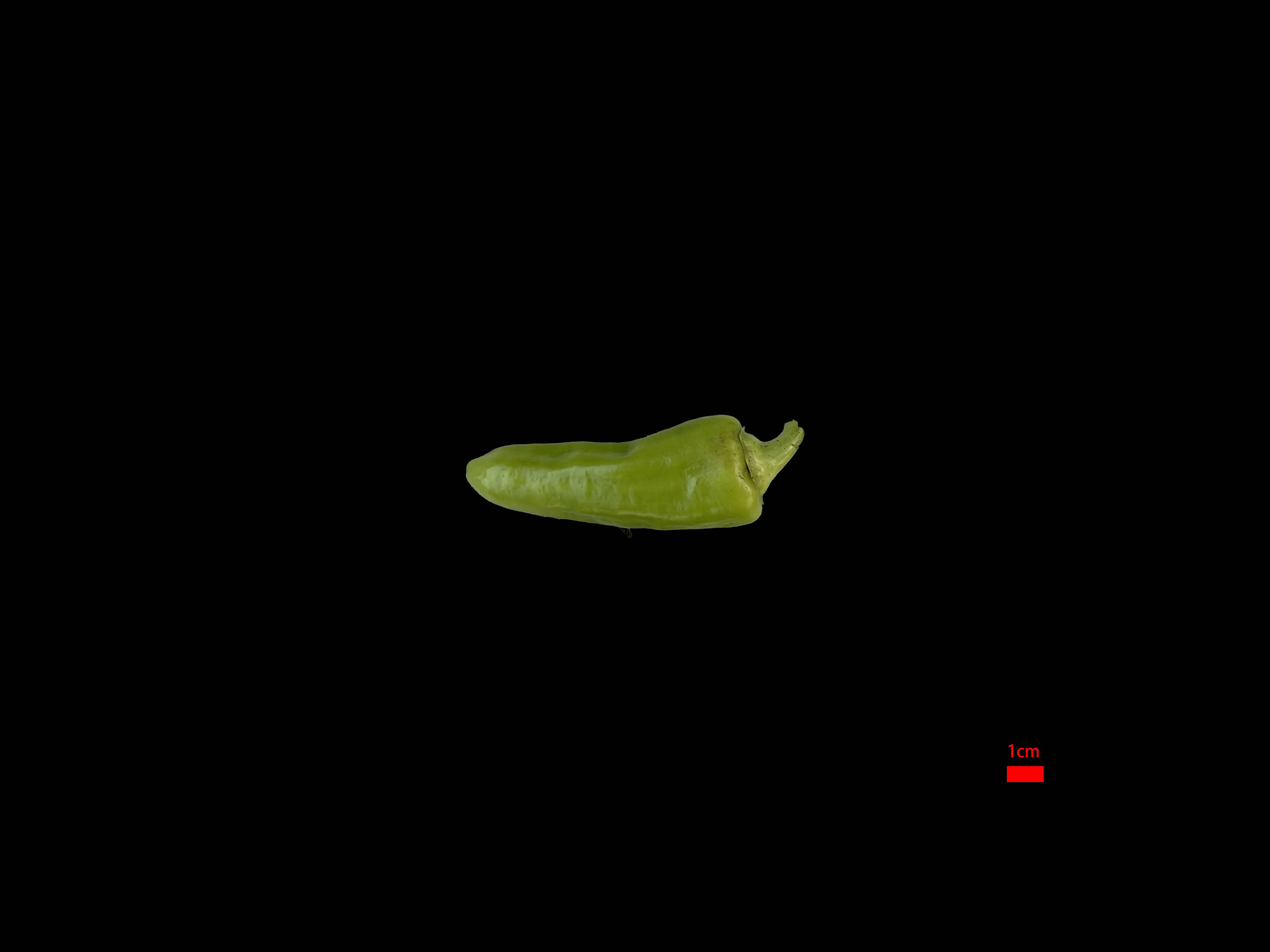

Supplement: Supplementary file 1 [file plants-15-02103-s001.zip › plants-4383327-supplementary/pepper_original_data/cone/41-8.jpg]

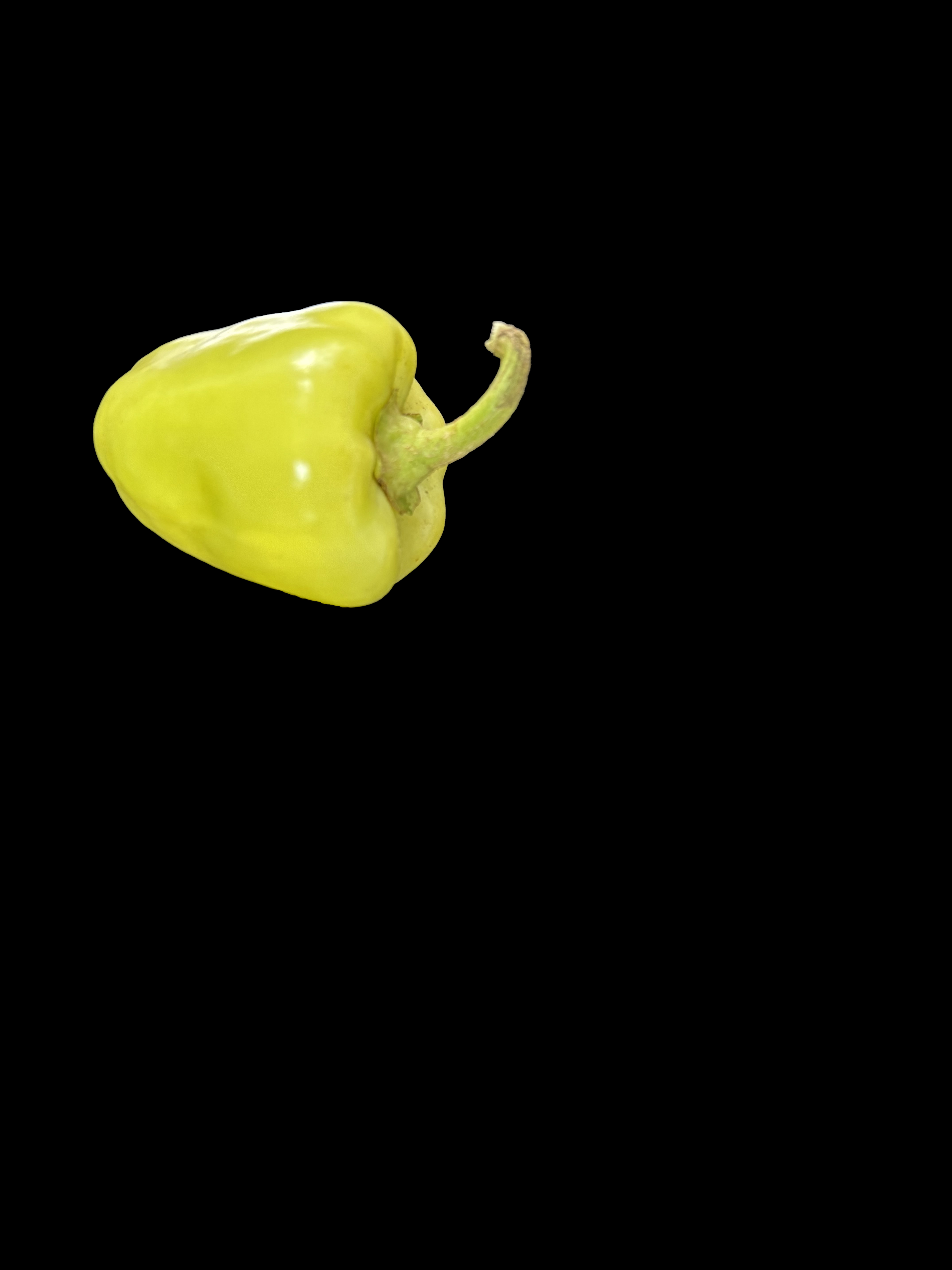

Supplement: Supplementary file 1 [file plants-15-02103-s001.zip › plants-4383327-supplementary/pepper_original_data/cone/47.jpg]

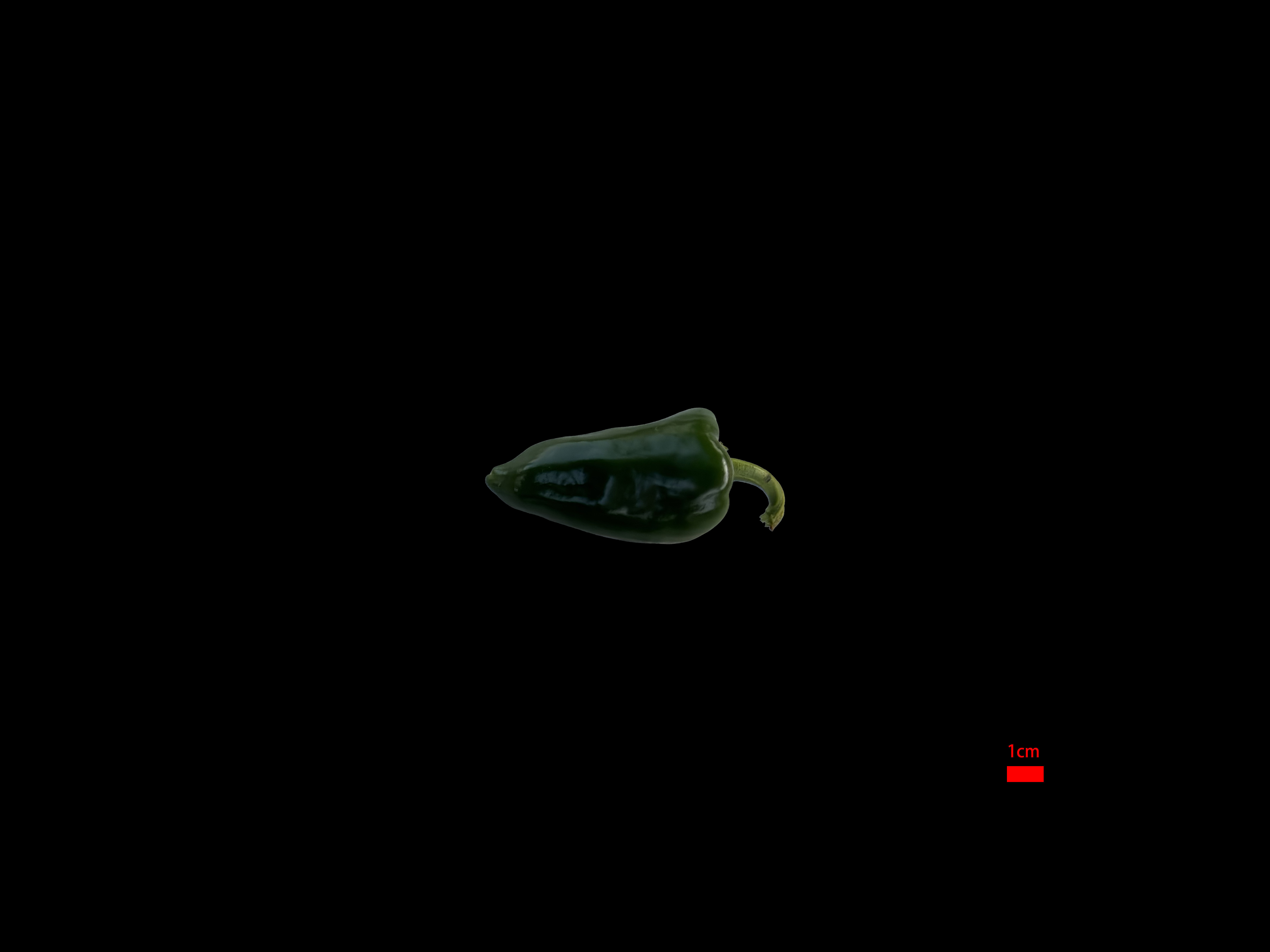

Supplement: Supplementary file 1 [file plants-15-02103-s001.zip › plants-4383327-supplementary/pepper_original_data/cone/50-1.jpg]

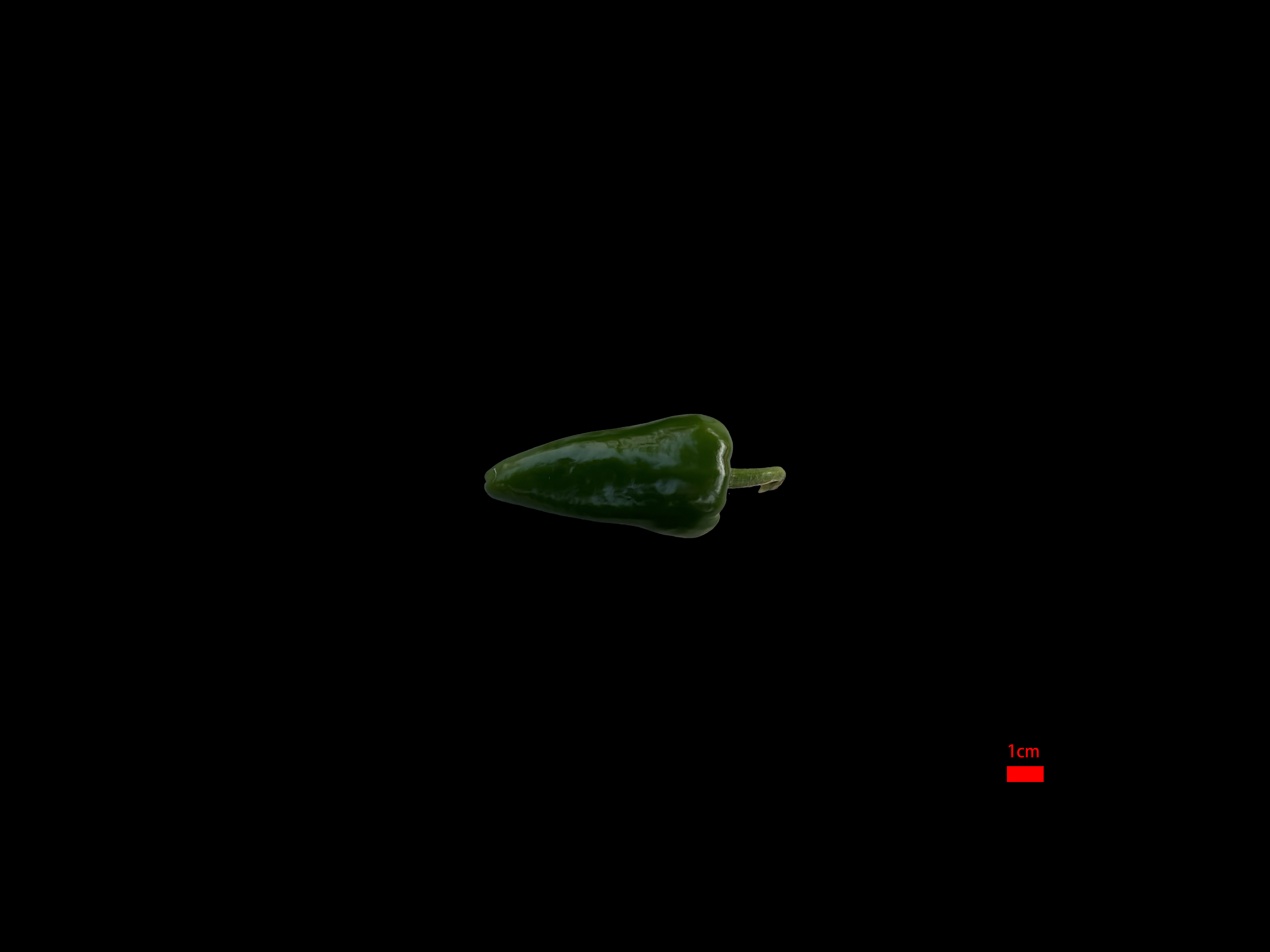

Supplement: Supplementary file 1 [file plants-15-02103-s001.zip › plants-4383327-supplementary/pepper_original_data/cone/50-2.jpg]

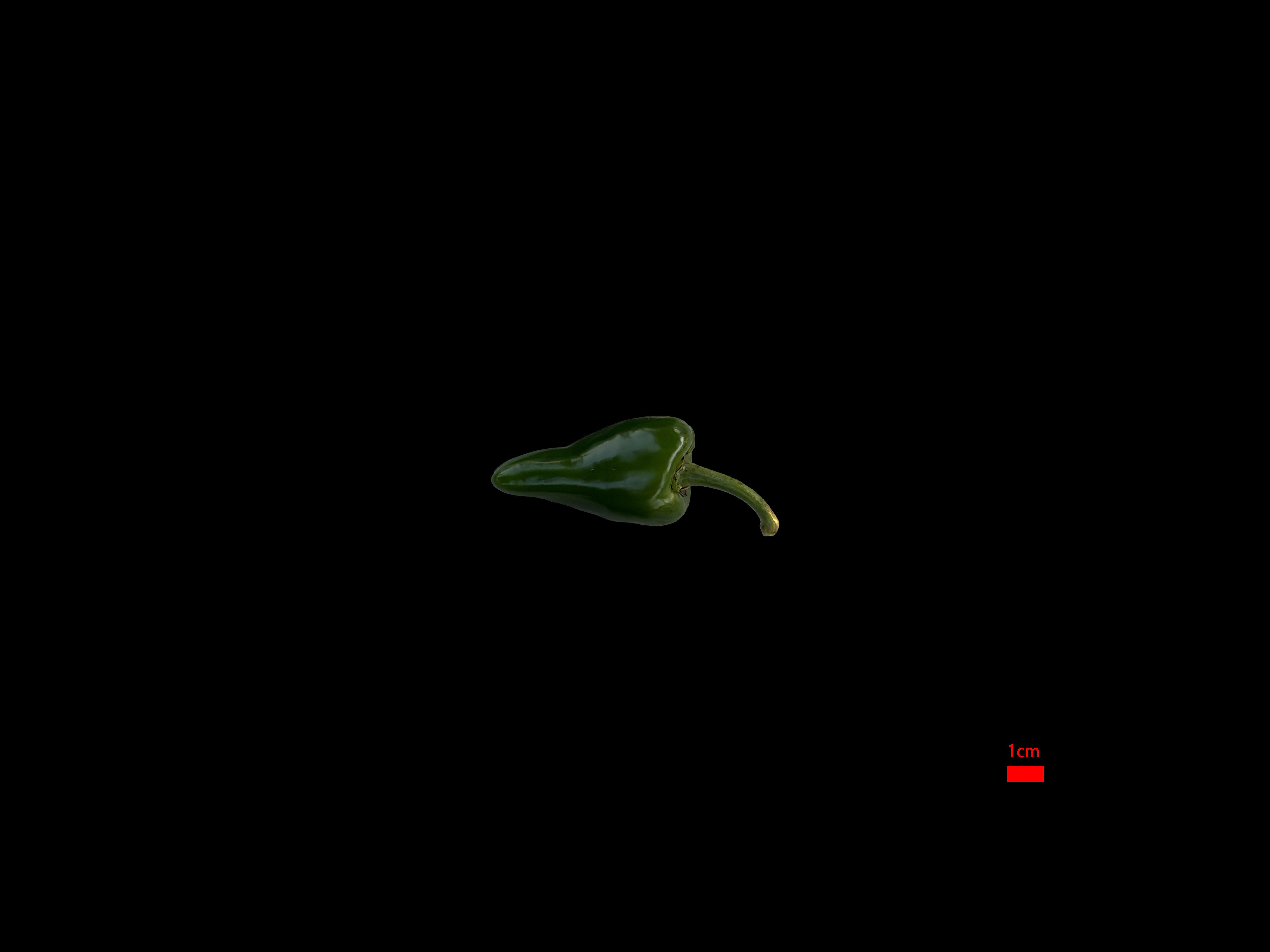

Supplement: Supplementary file 1 [file plants-15-02103-s001.zip › plants-4383327-supplementary/pepper_original_data/cone/50-3.jpg]

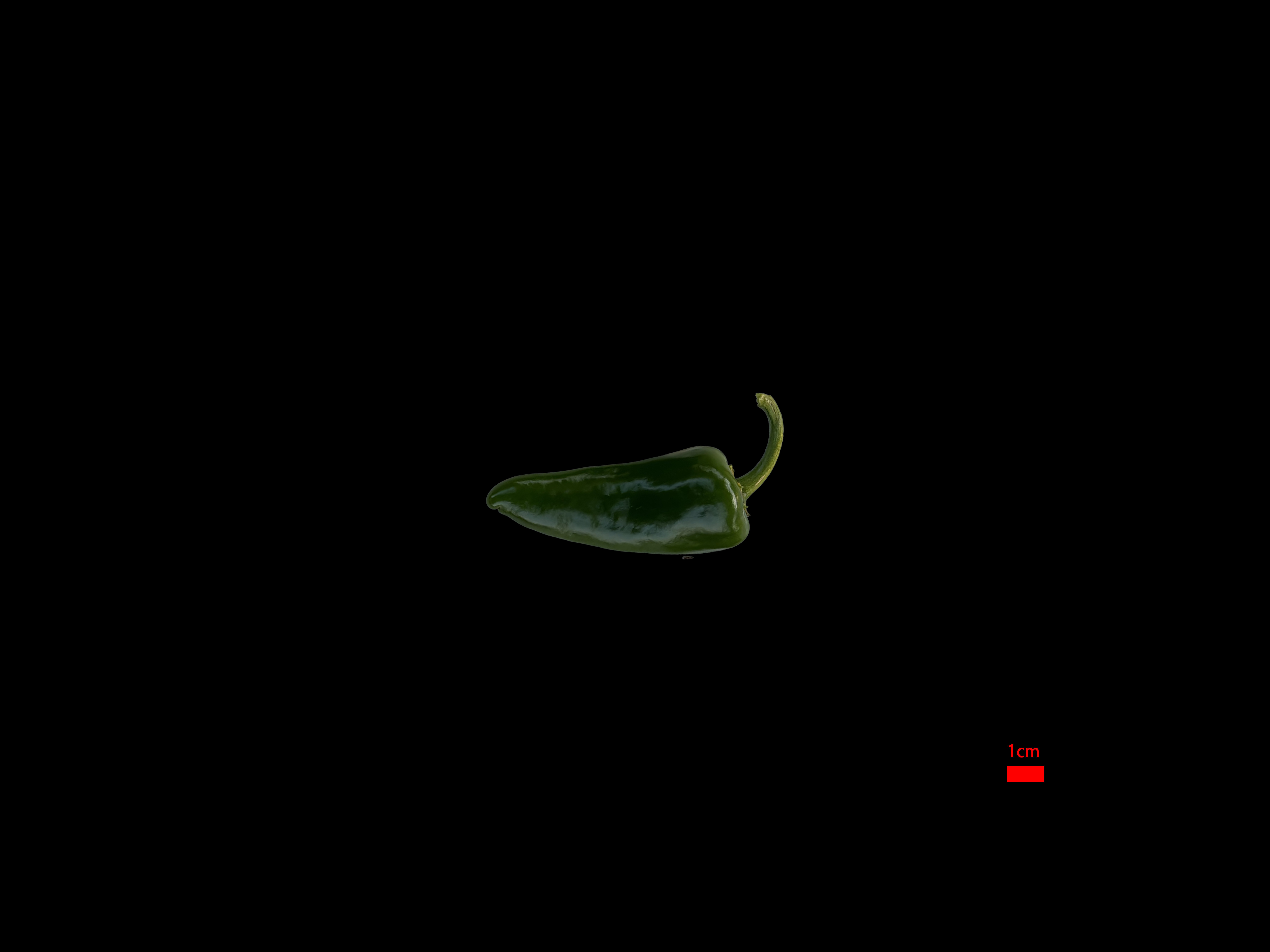

Supplement: Supplementary file 1 [file plants-15-02103-s001.zip › plants-4383327-supplementary/pepper_original_data/cone/50-4.jpg]

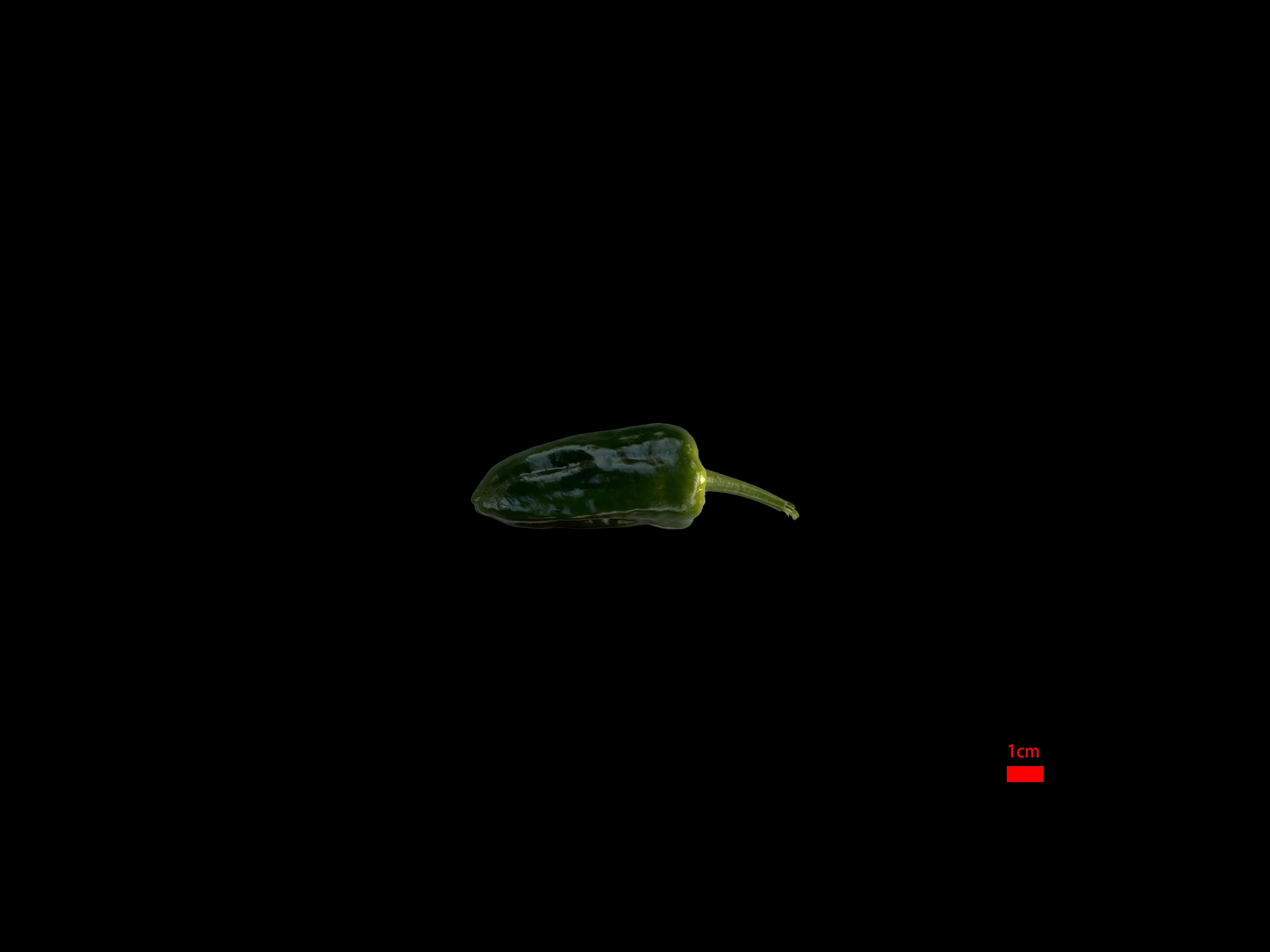

Supplement: Supplementary file 1 [file plants-15-02103-s001.zip › plants-4383327-supplementary/pepper_original_data/cone/50-5.jpg]

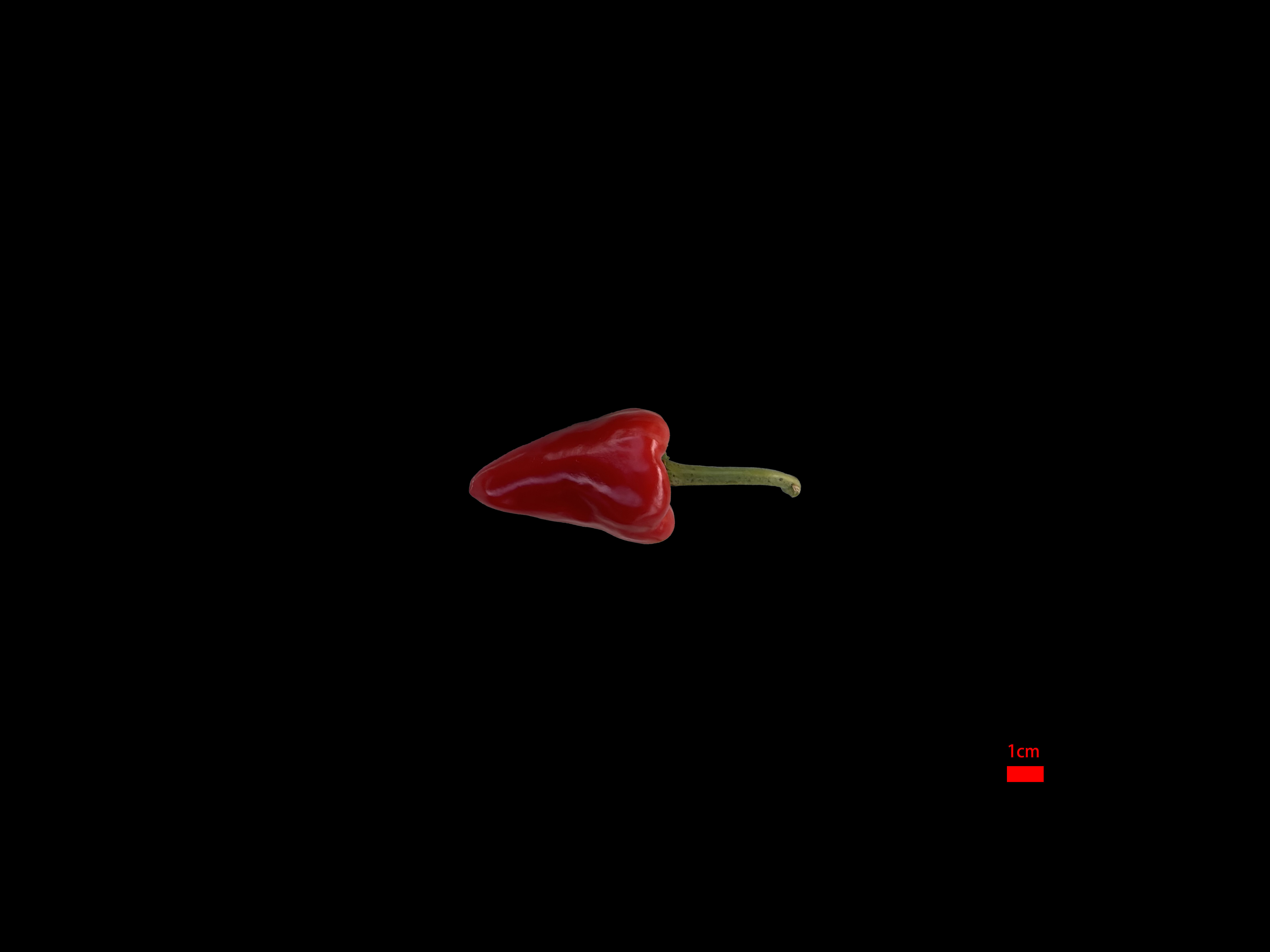

Supplement: Supplementary file 1 [file plants-15-02103-s001.zip › plants-4383327-supplementary/pepper_original_data/cone/50-6.jpg]

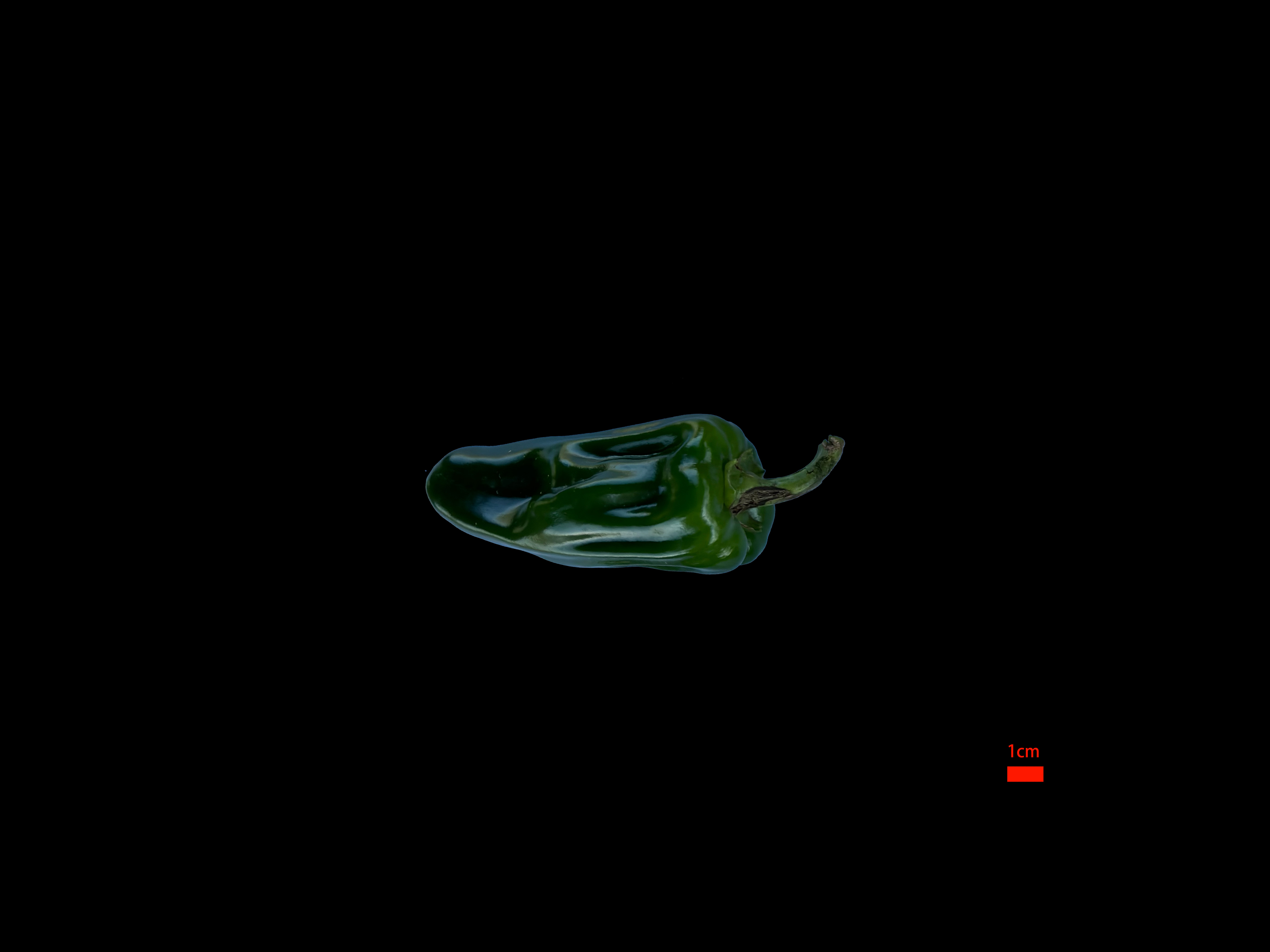

Supplement: Supplementary file 1 [file plants-15-02103-s001.zip › plants-4383327-supplementary/pepper_original_data/cone/81-10.jpg]

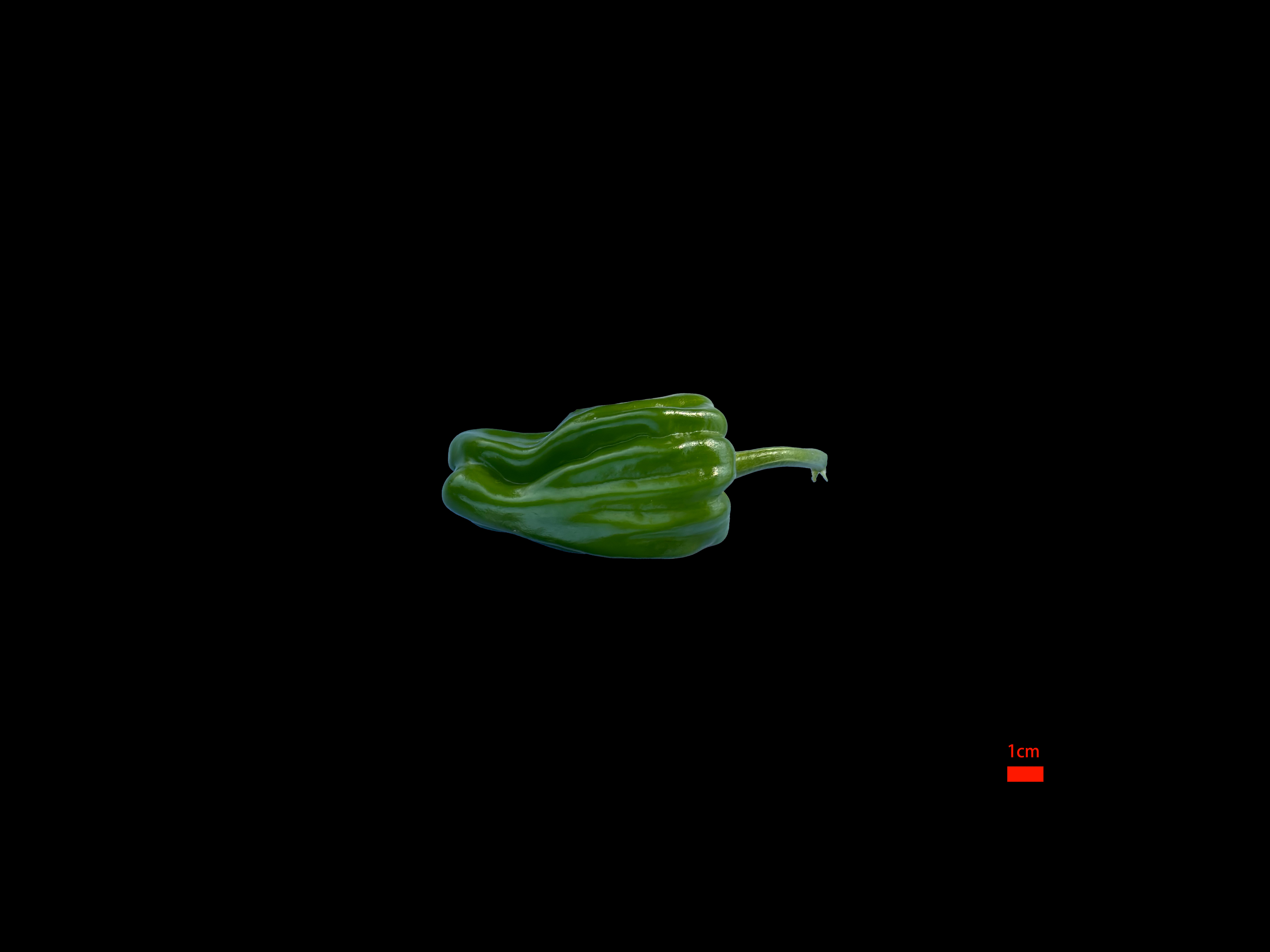

Supplement: Supplementary file 1 [file plants-15-02103-s001.zip › plants-4383327-supplementary/pepper_original_data/cone/81-2.jpg]

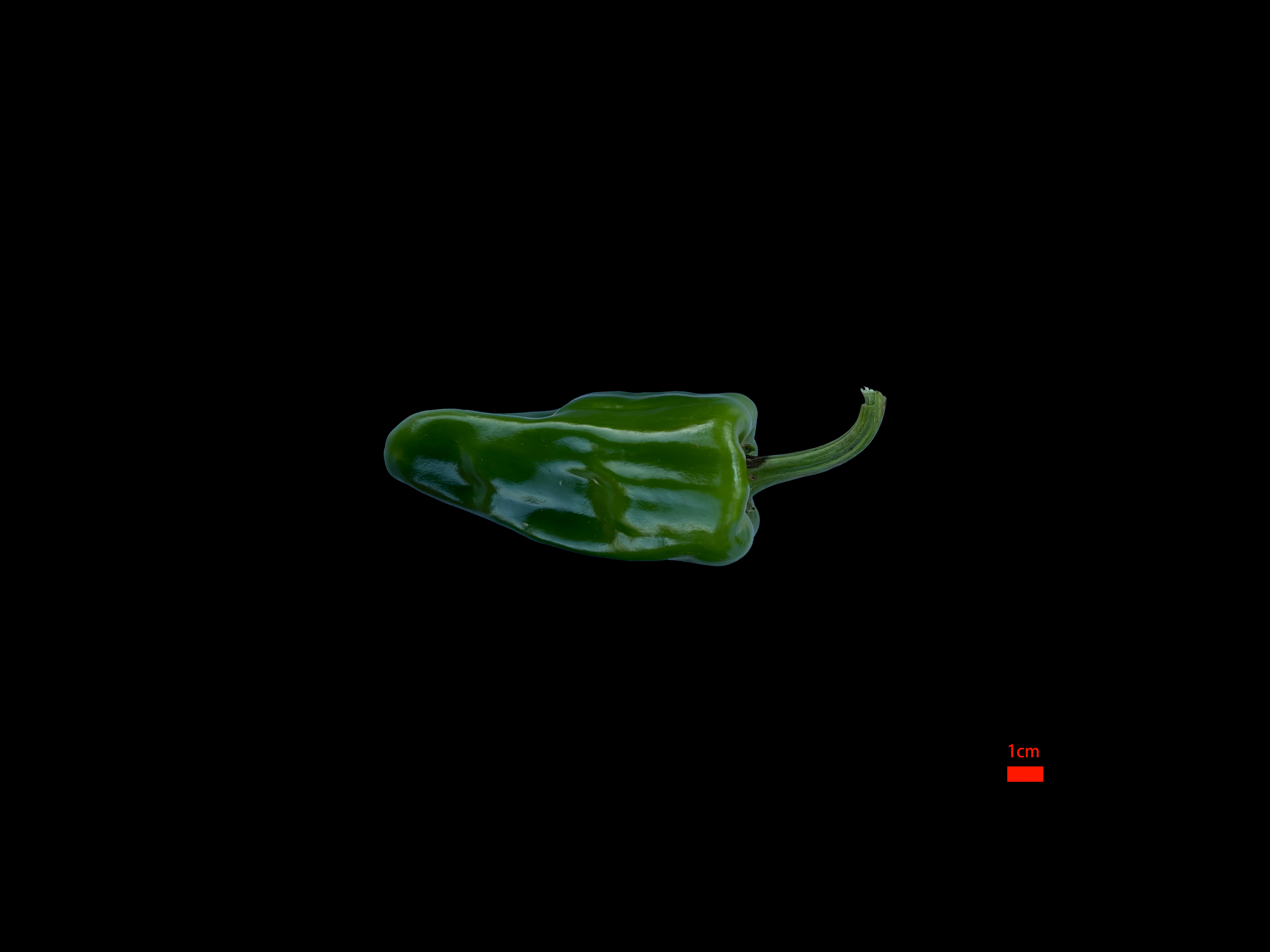

Supplement: Supplementary file 1 [file plants-15-02103-s001.zip › plants-4383327-supplementary/pepper_original_data/cone/81-7.jpg]

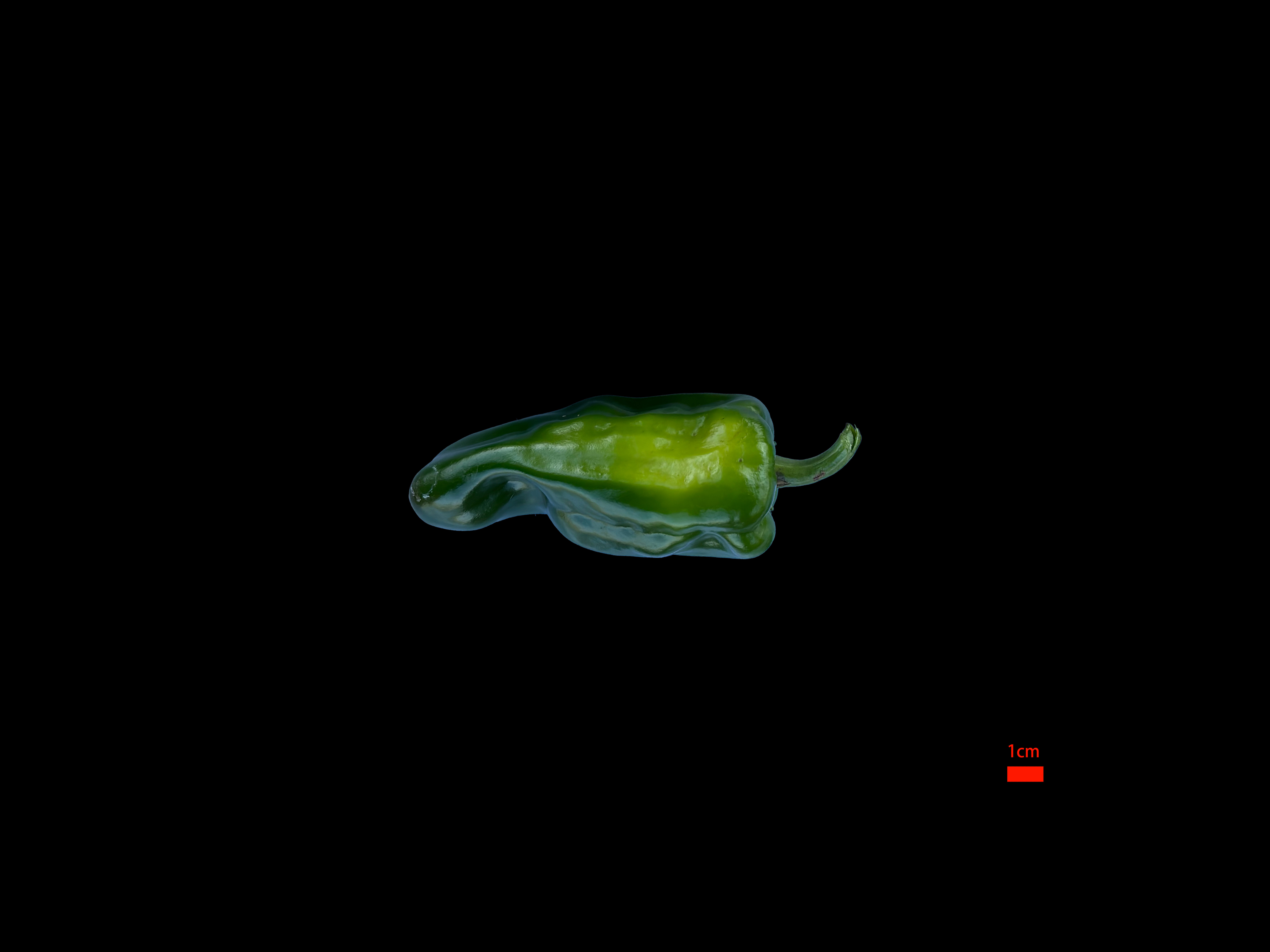

Supplement: Supplementary file 1 [file plants-15-02103-s001.zip › plants-4383327-supplementary/pepper_original_data/cone/81-8.jpg]

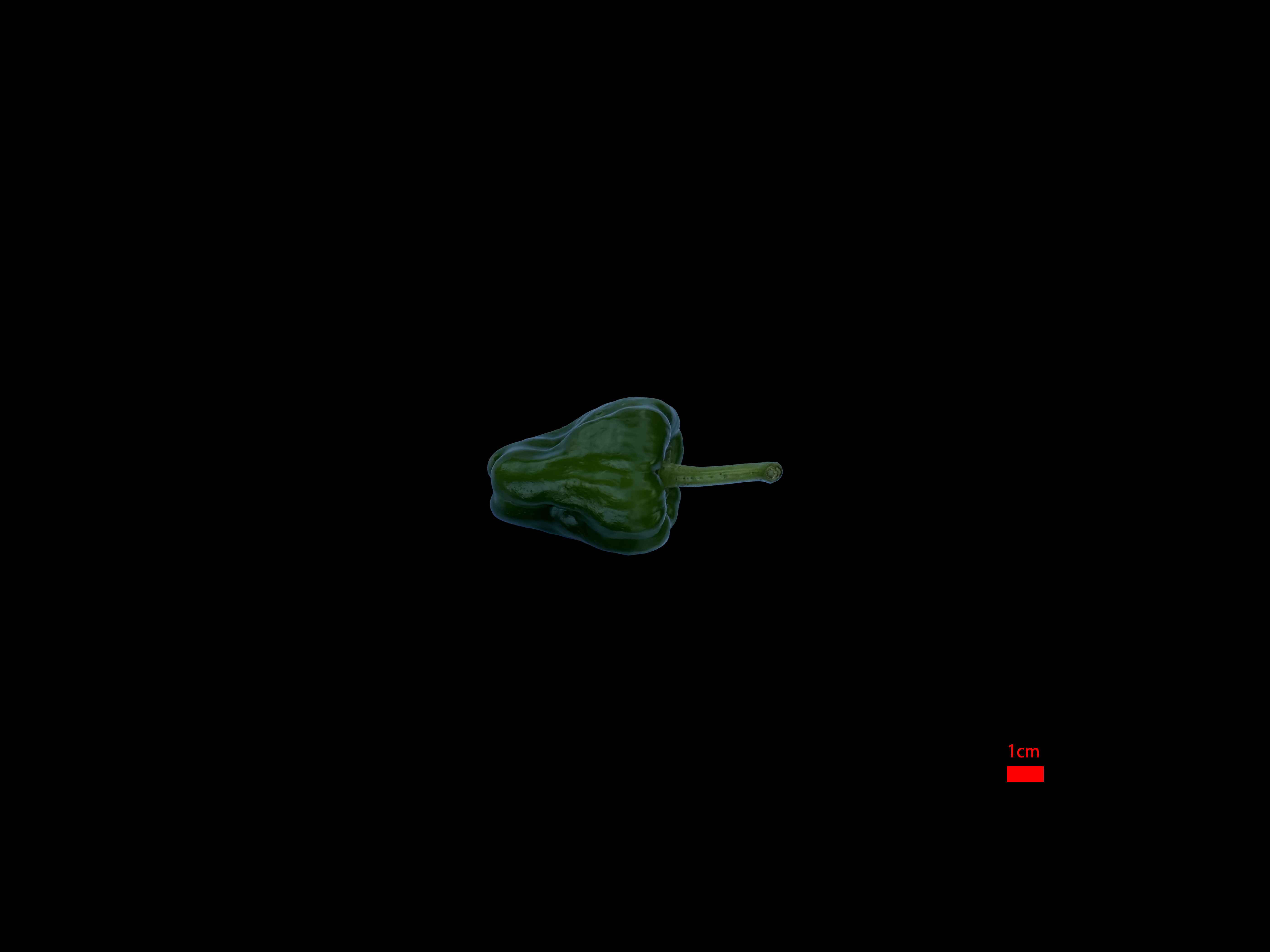

Supplement: Supplementary file 1 [file plants-15-02103-s001.zip › plants-4383327-supplementary/pepper_original_data/cone/90-1.jpg]

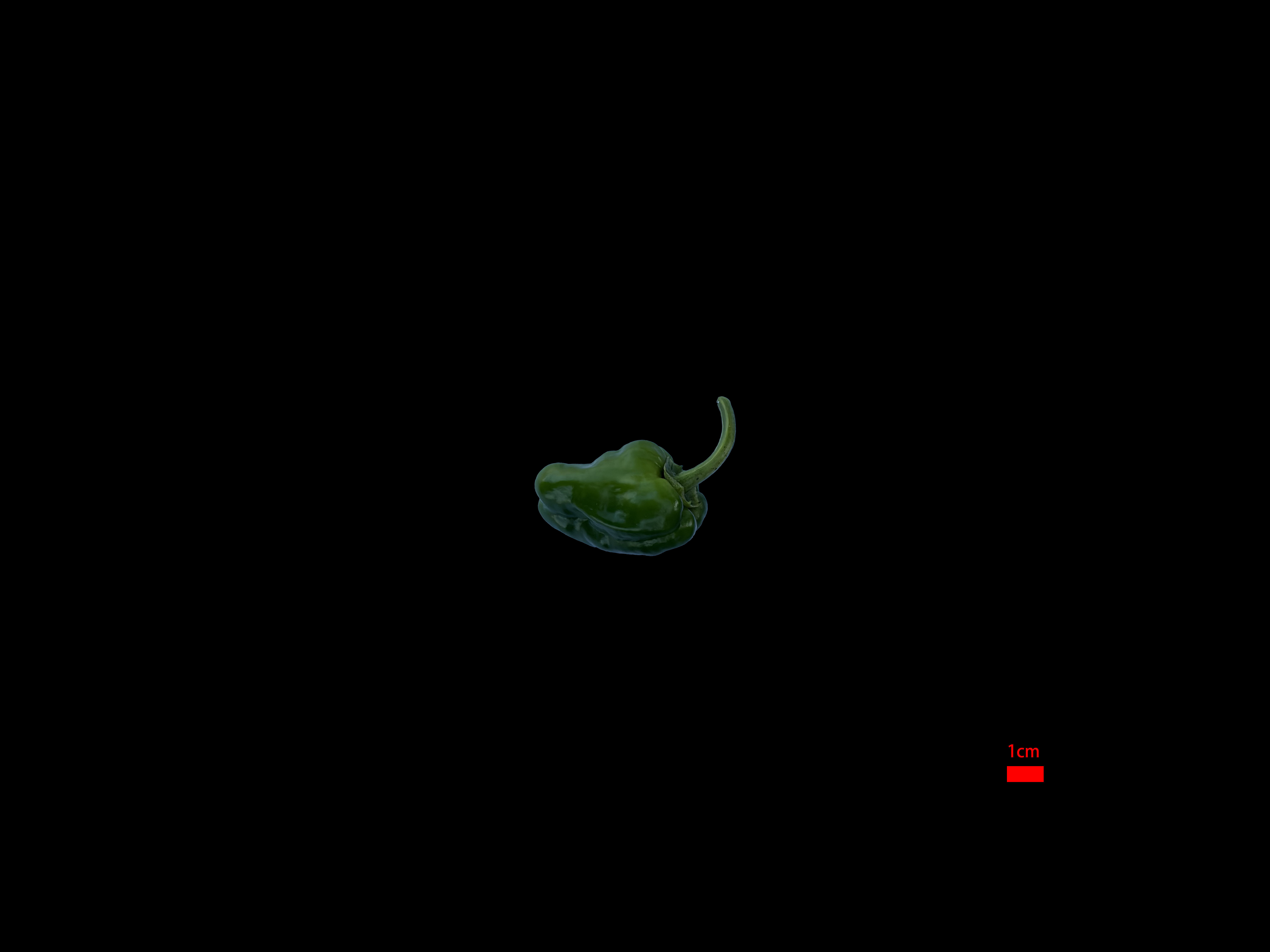

Supplement: Supplementary file 1 [file plants-15-02103-s001.zip › plants-4383327-supplementary/pepper_original_data/cone/90-10.jpg]

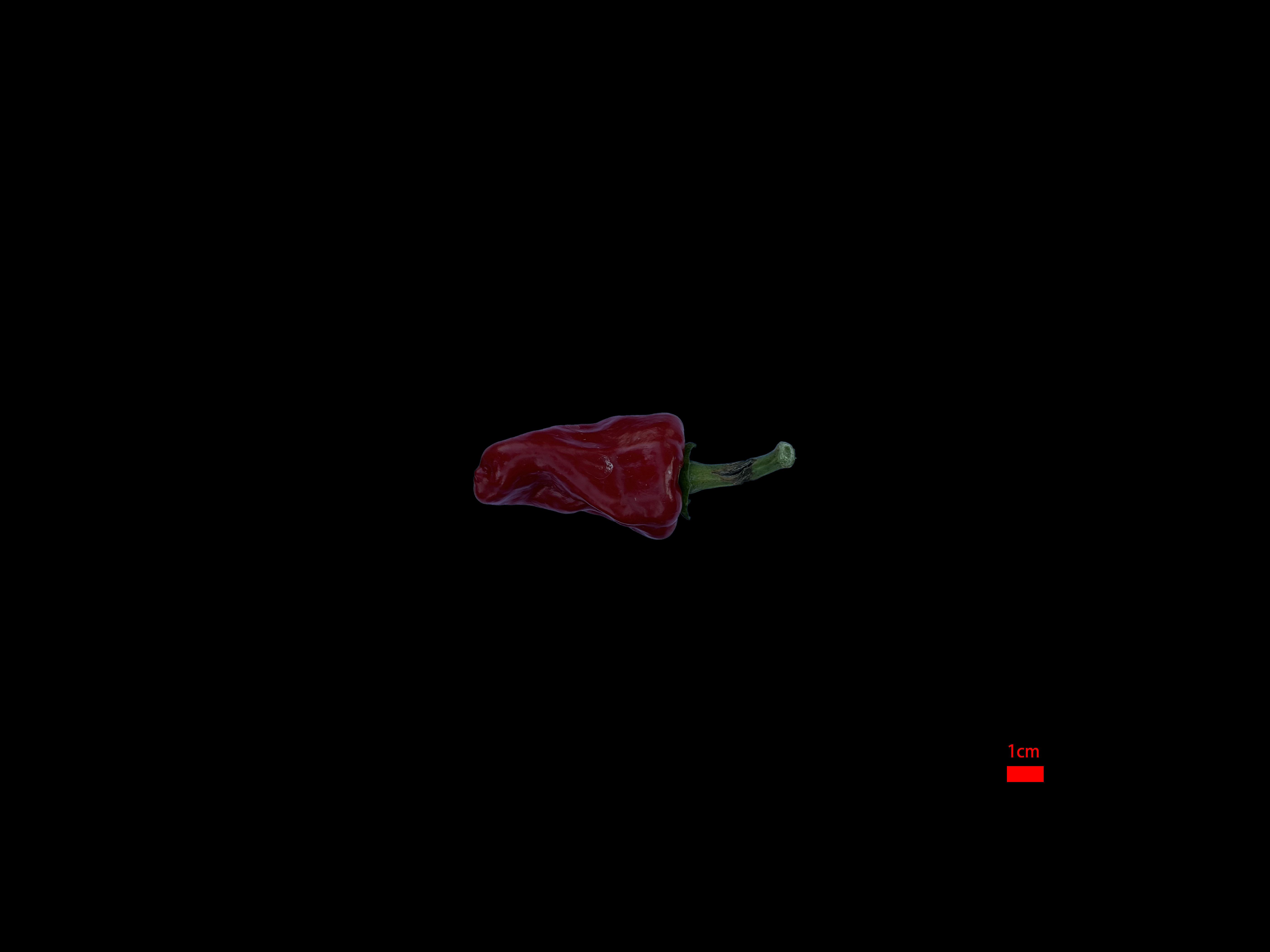

Supplement: Supplementary file 1 [file plants-15-02103-s001.zip › plants-4383327-supplementary/pepper_original_data/cone/90-3.jpg]

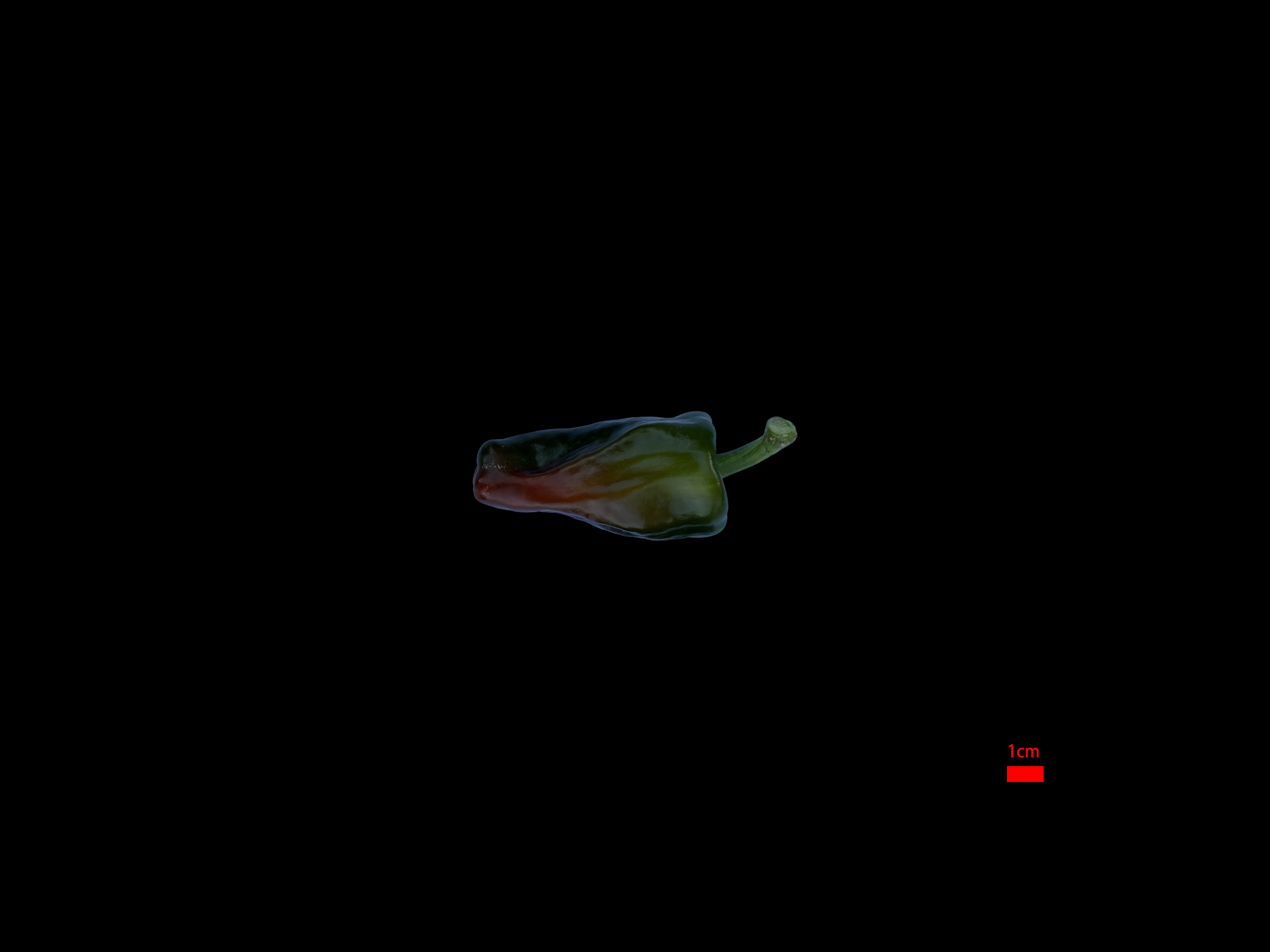

Supplement: Supplementary file 1 [file plants-15-02103-s001.zip › plants-4383327-supplementary/pepper_original_data/cone/90-4.jpg]

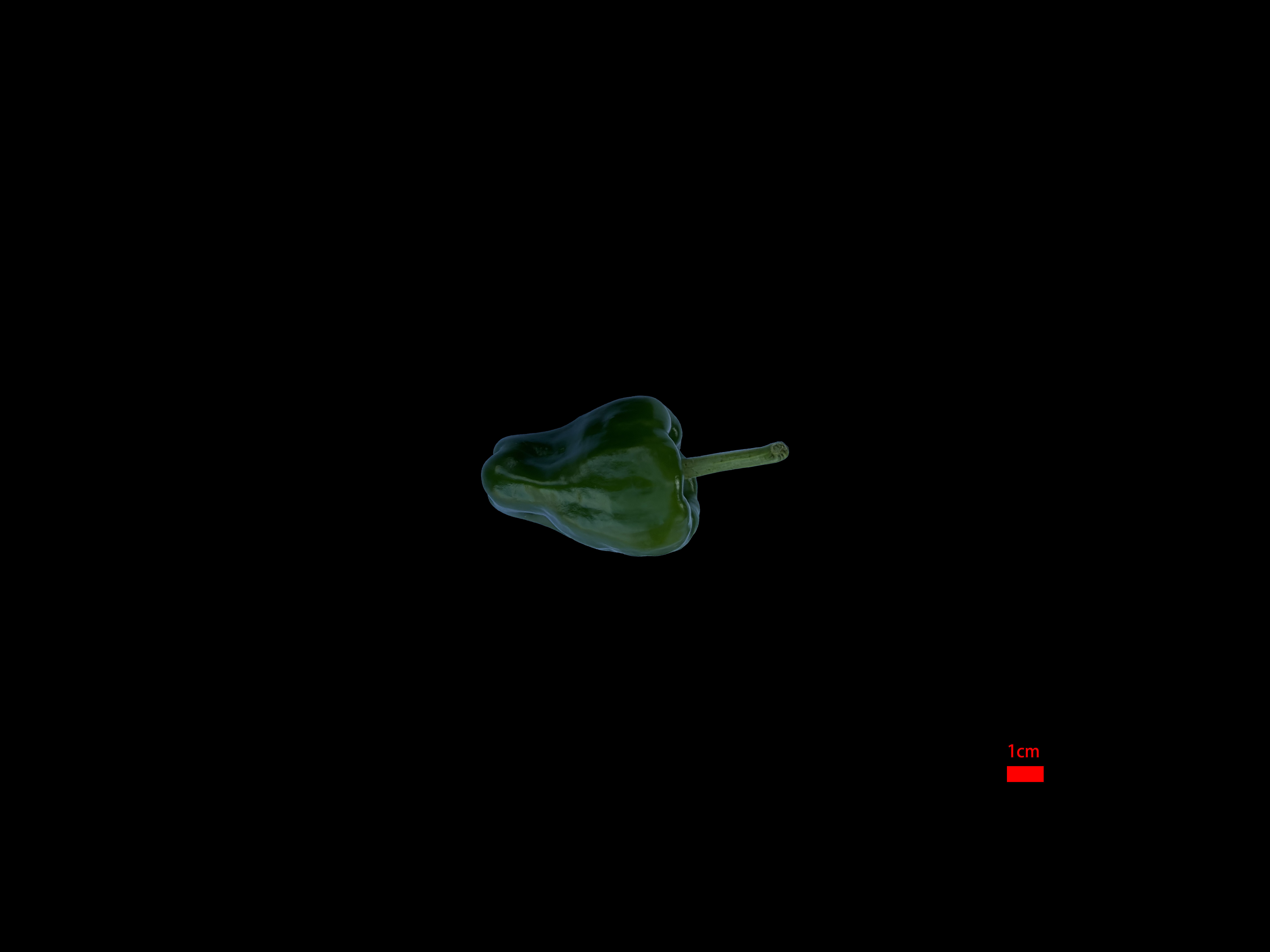

Supplement: Supplementary file 1 [file plants-15-02103-s001.zip › plants-4383327-supplementary/pepper_original_data/cone/90-5.jpg]

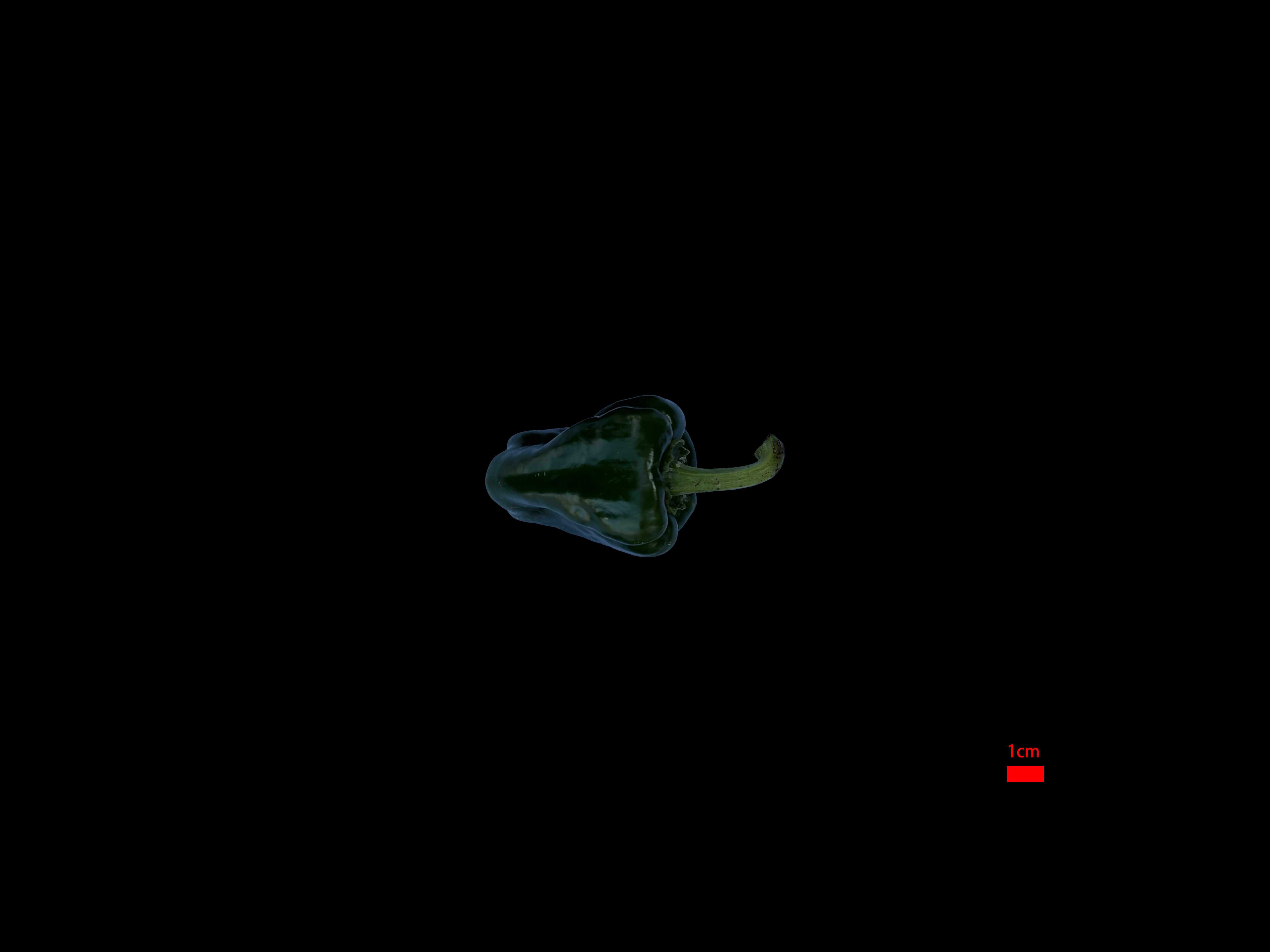

Supplement: Supplementary file 1 [file plants-15-02103-s001.zip › plants-4383327-supplementary/pepper_original_data/cone/90-8.jpg]

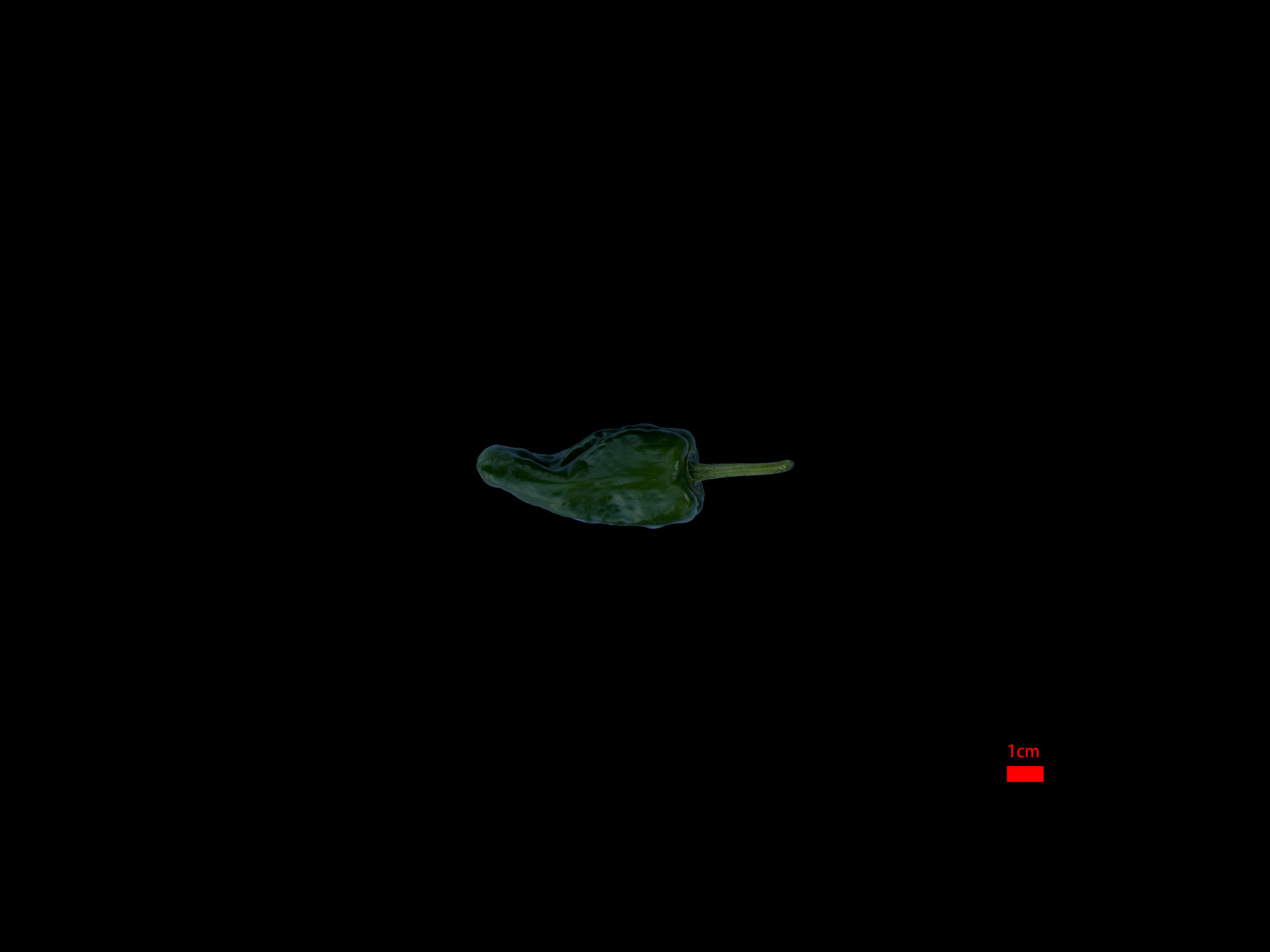

Supplement: Supplementary file 1 [file plants-15-02103-s001.zip › plants-4383327-supplementary/pepper_original_data/cone/90-9.jpg]

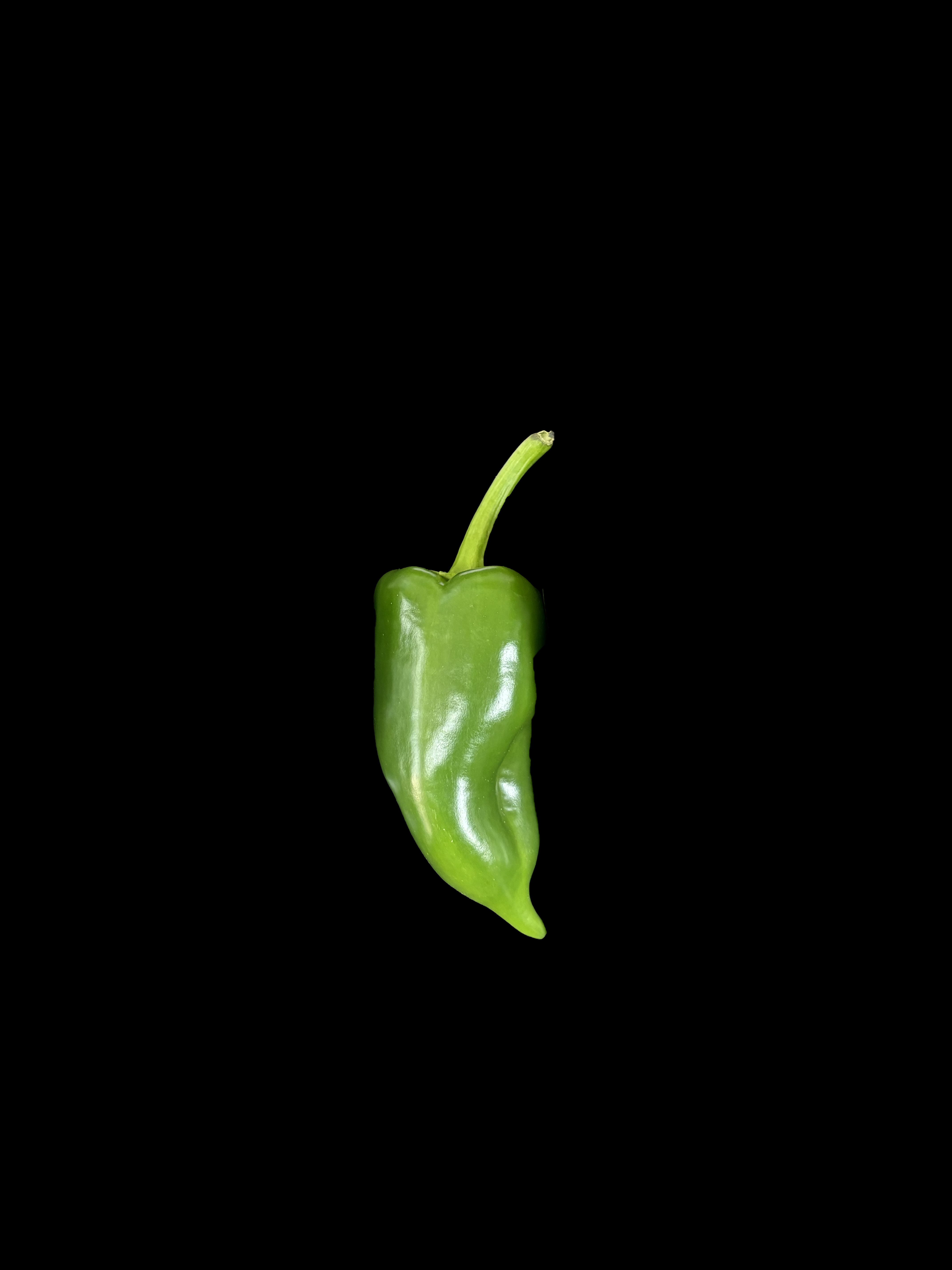

Supplement: Supplementary file 1 [file plants-15-02103-s001.zip › plants-4383327-supplementary/pepper_original_data/cone/dsb.1.jpg]

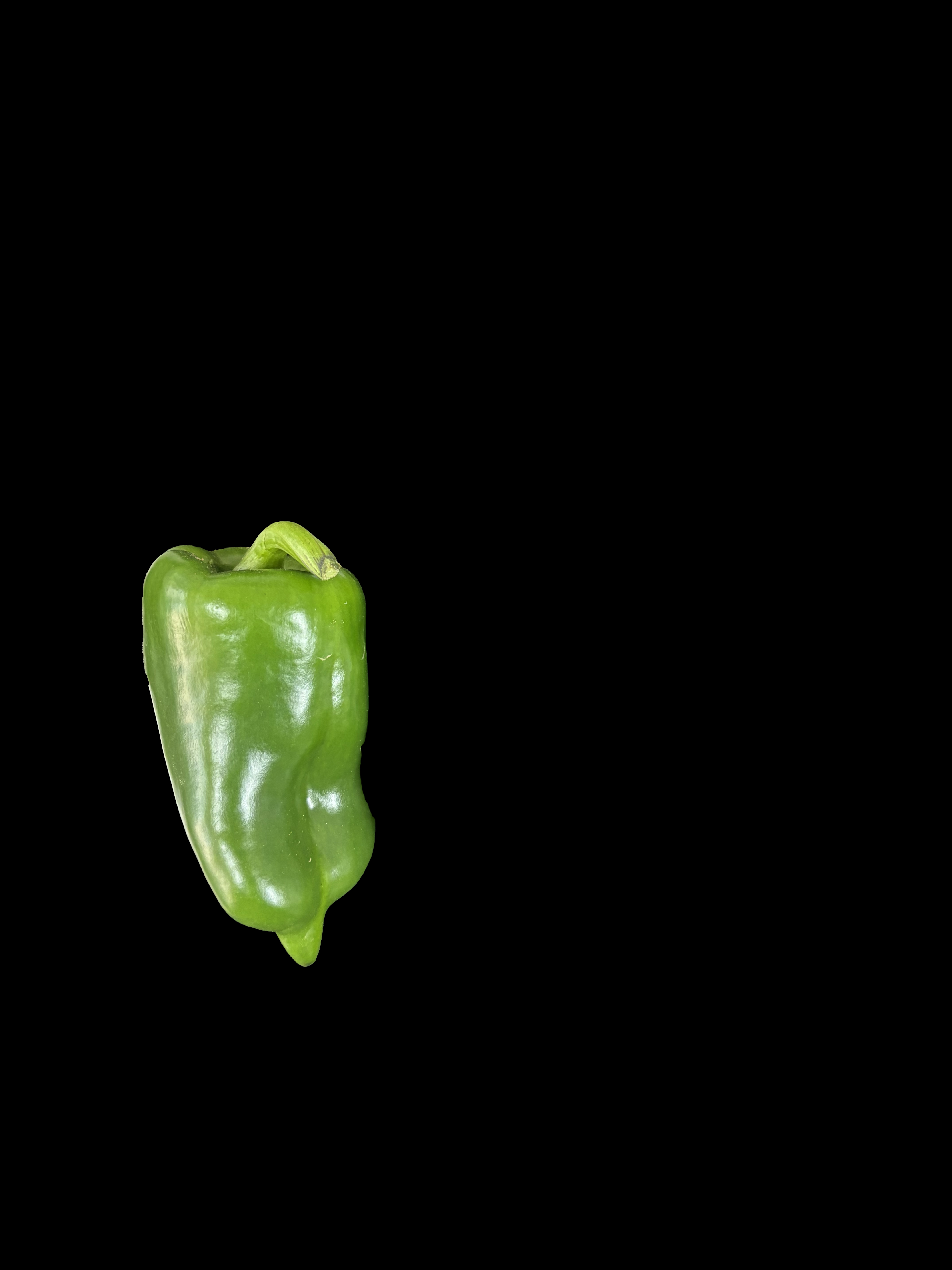

Supplement: Supplementary file 1 [file plants-15-02103-s001.zip › plants-4383327-supplementary/pepper_original_data/cone/dsb.2.jpg]

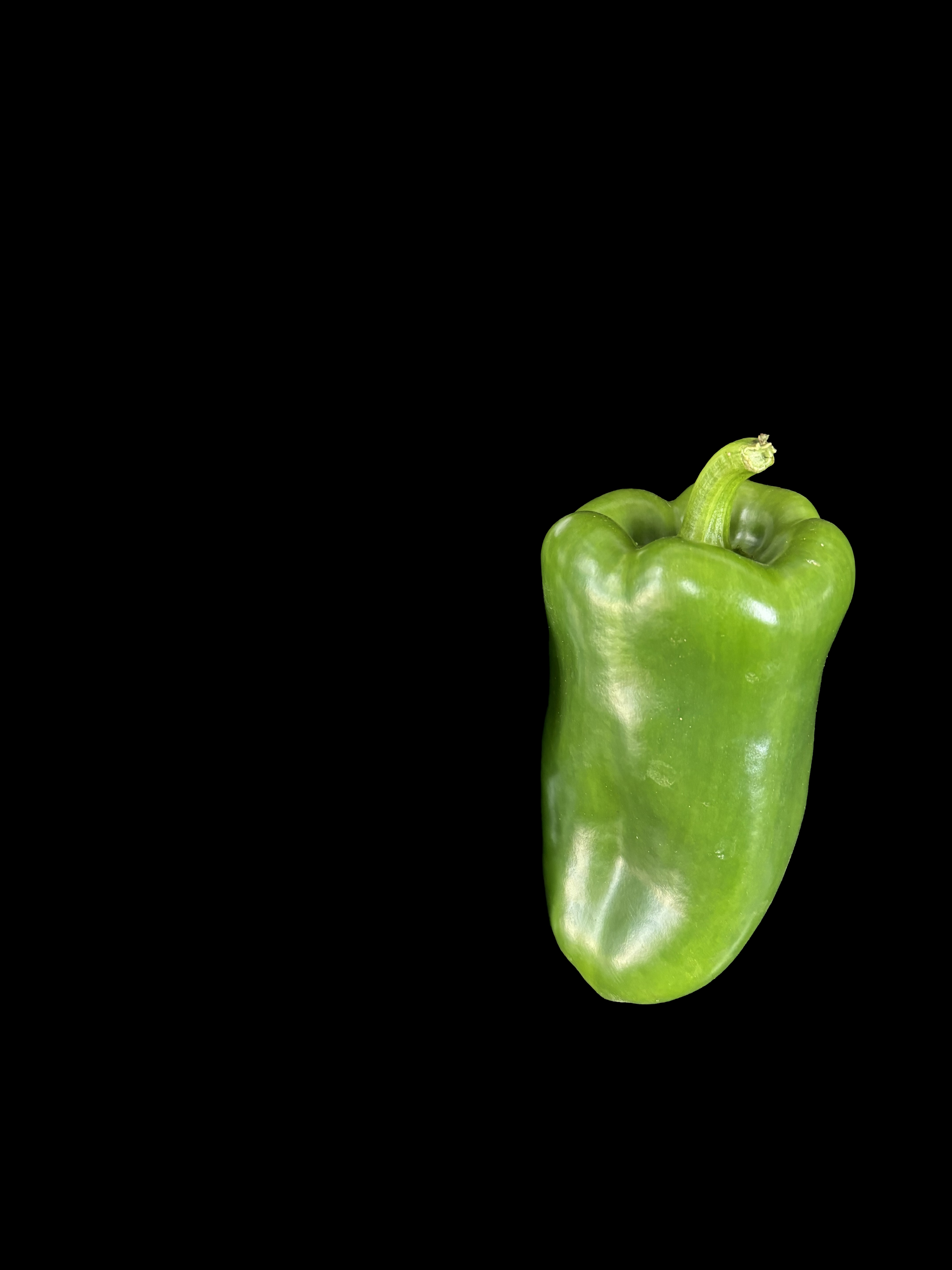

Supplement: Supplementary file 1 [file plants-15-02103-s001.zip › plants-4383327-supplementary/pepper_original_data/cone/dsb.jpg]

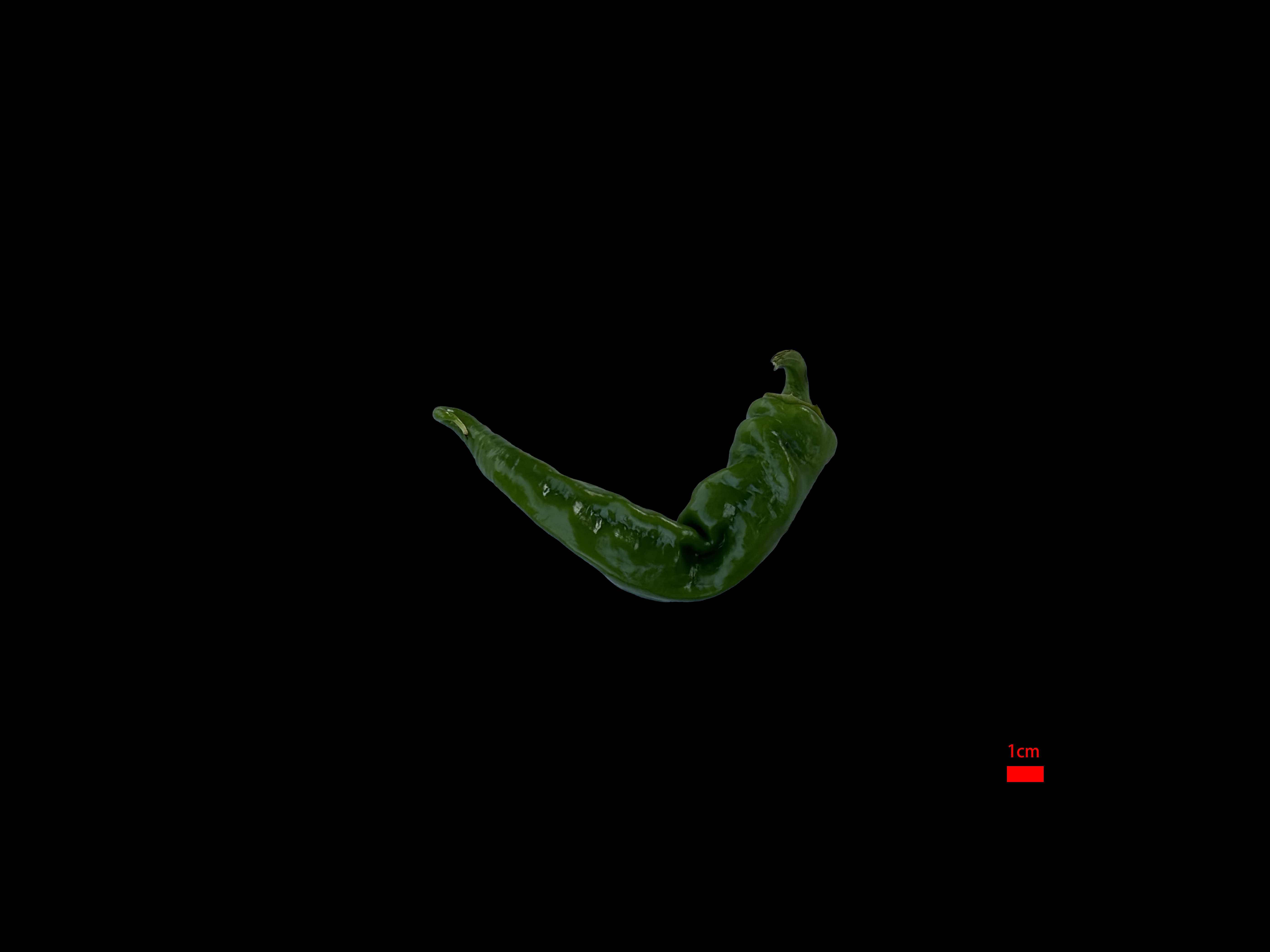

Supplement: Supplementary file 1 [file plants-15-02103-s001.zip › plants-4383327-supplementary/pepper_original_data/Goat_horn/100-1.jpg]

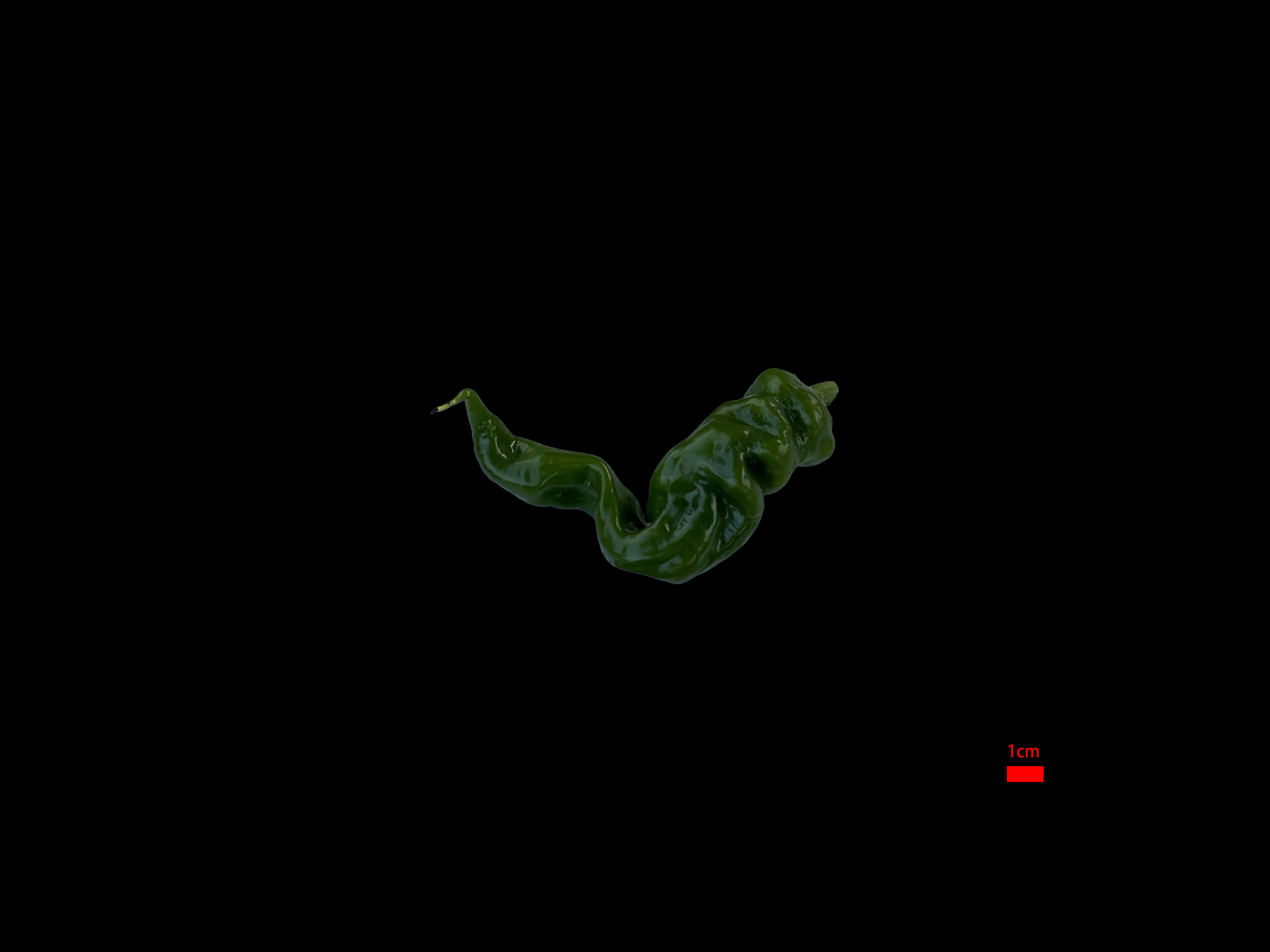

Supplement: Supplementary file 1 [file plants-15-02103-s001.zip › plants-4383327-supplementary/pepper_original_data/Goat_horn/100-2.jpg]

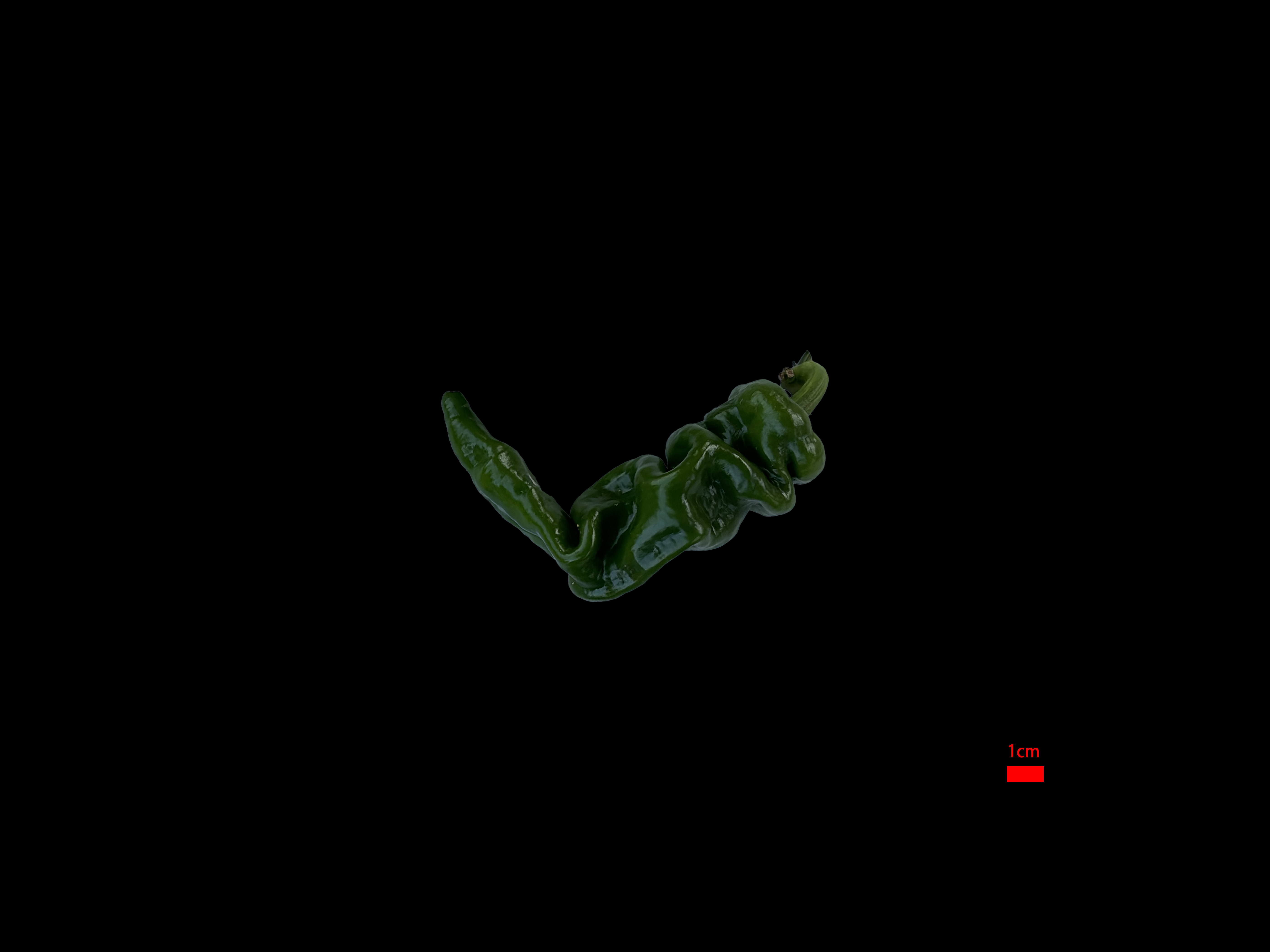

Supplement: Supplementary file 1 [file plants-15-02103-s001.zip › plants-4383327-supplementary/pepper_original_data/Goat_horn/100-3.jpg]

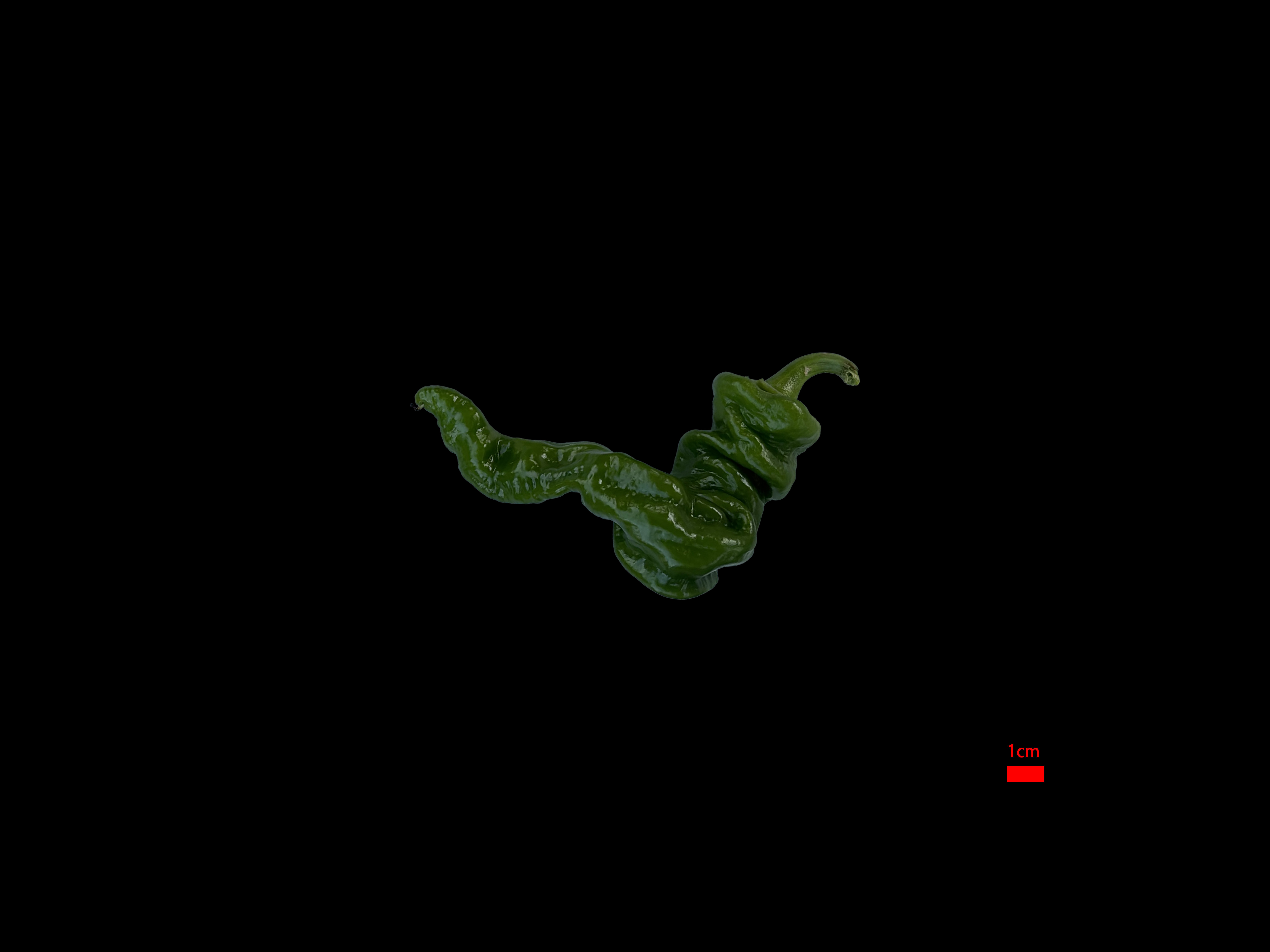

Supplement: Supplementary file 1 [file plants-15-02103-s001.zip › plants-4383327-supplementary/pepper_original_data/Goat_horn/100-4.jpg]

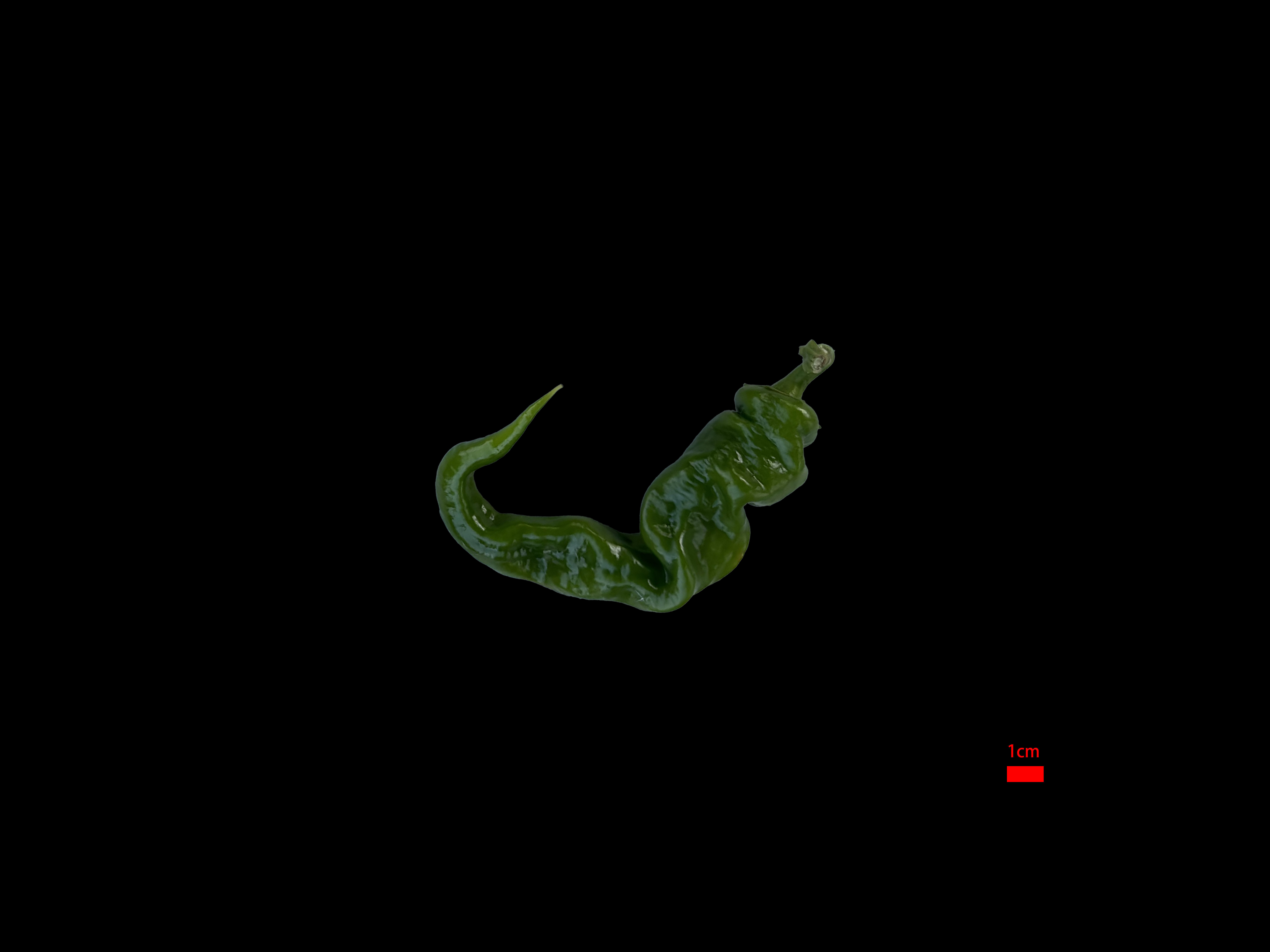

Supplement: Supplementary file 1 [file plants-15-02103-s001.zip › plants-4383327-supplementary/pepper_original_data/Goat_horn/100-5.jpg]

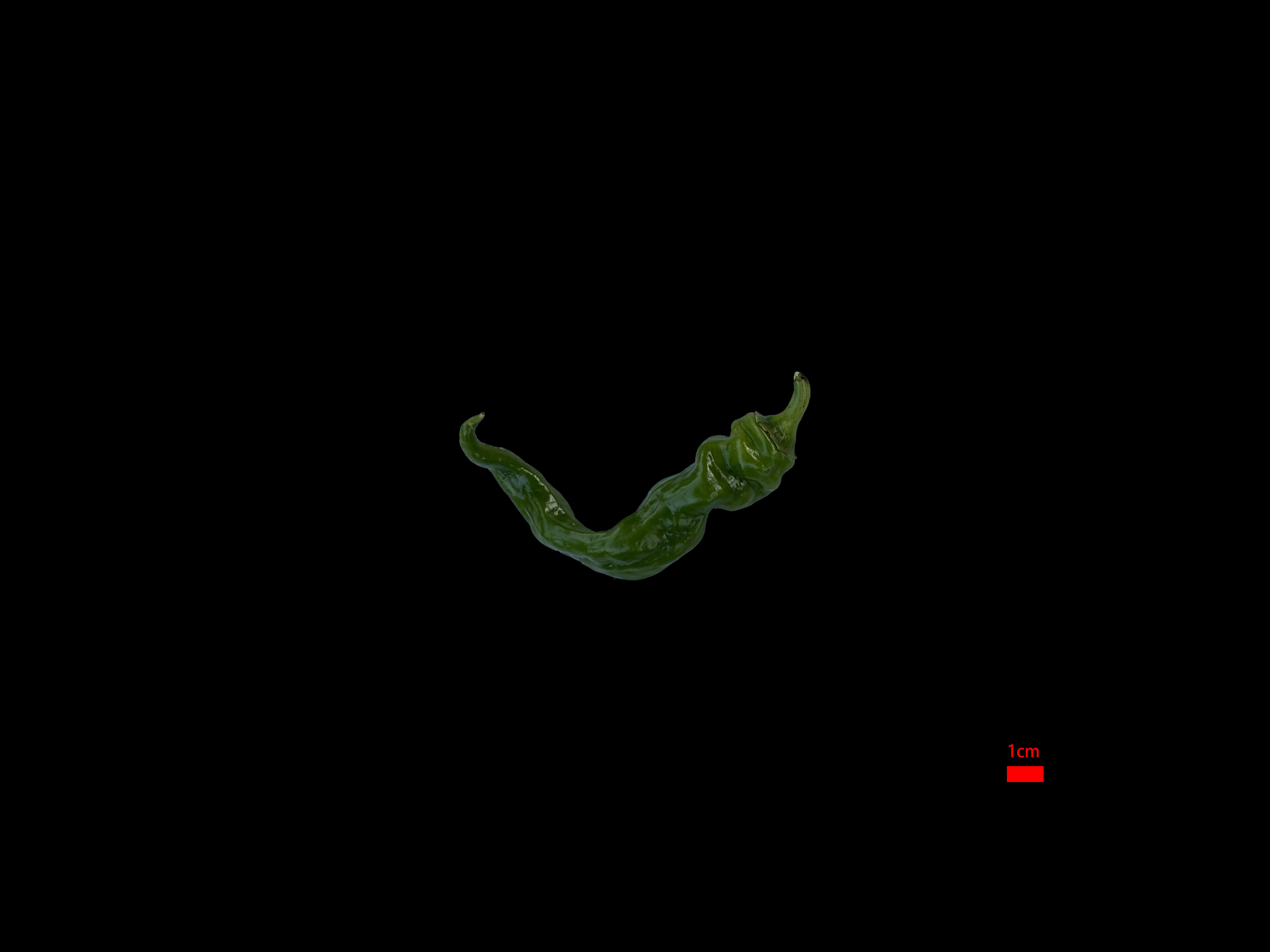

Supplement: Supplementary file 1 [file plants-15-02103-s001.zip › plants-4383327-supplementary/pepper_original_data/Goat_horn/100-8.jpg]

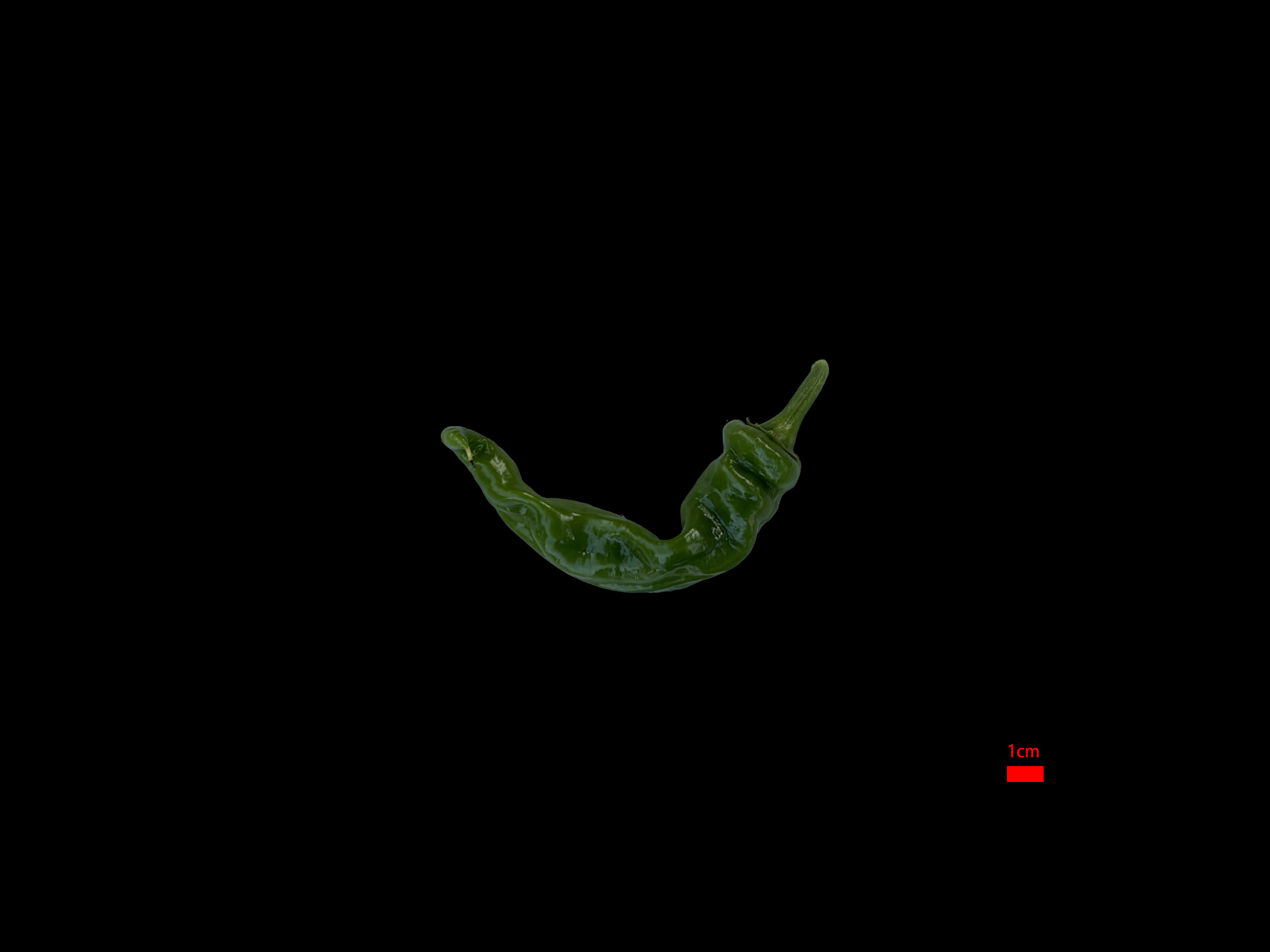

Supplement: Supplementary file 1 [file plants-15-02103-s001.zip › plants-4383327-supplementary/pepper_original_data/Goat_horn/100-9.jpg]

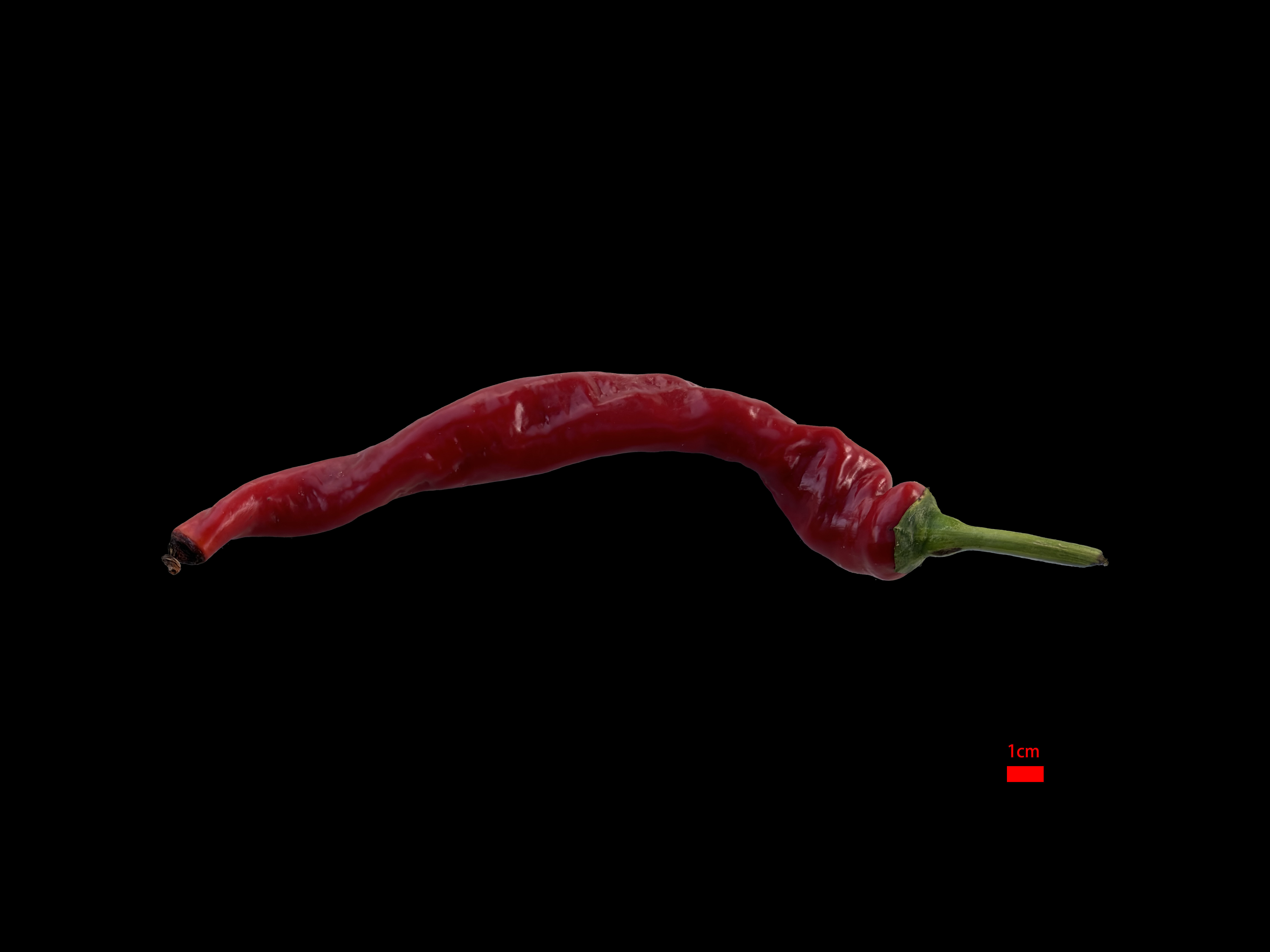

Supplement: Supplementary file 1 [file plants-15-02103-s001.zip › plants-4383327-supplementary/pepper_original_data/Goat_horn/101-1.jpg]

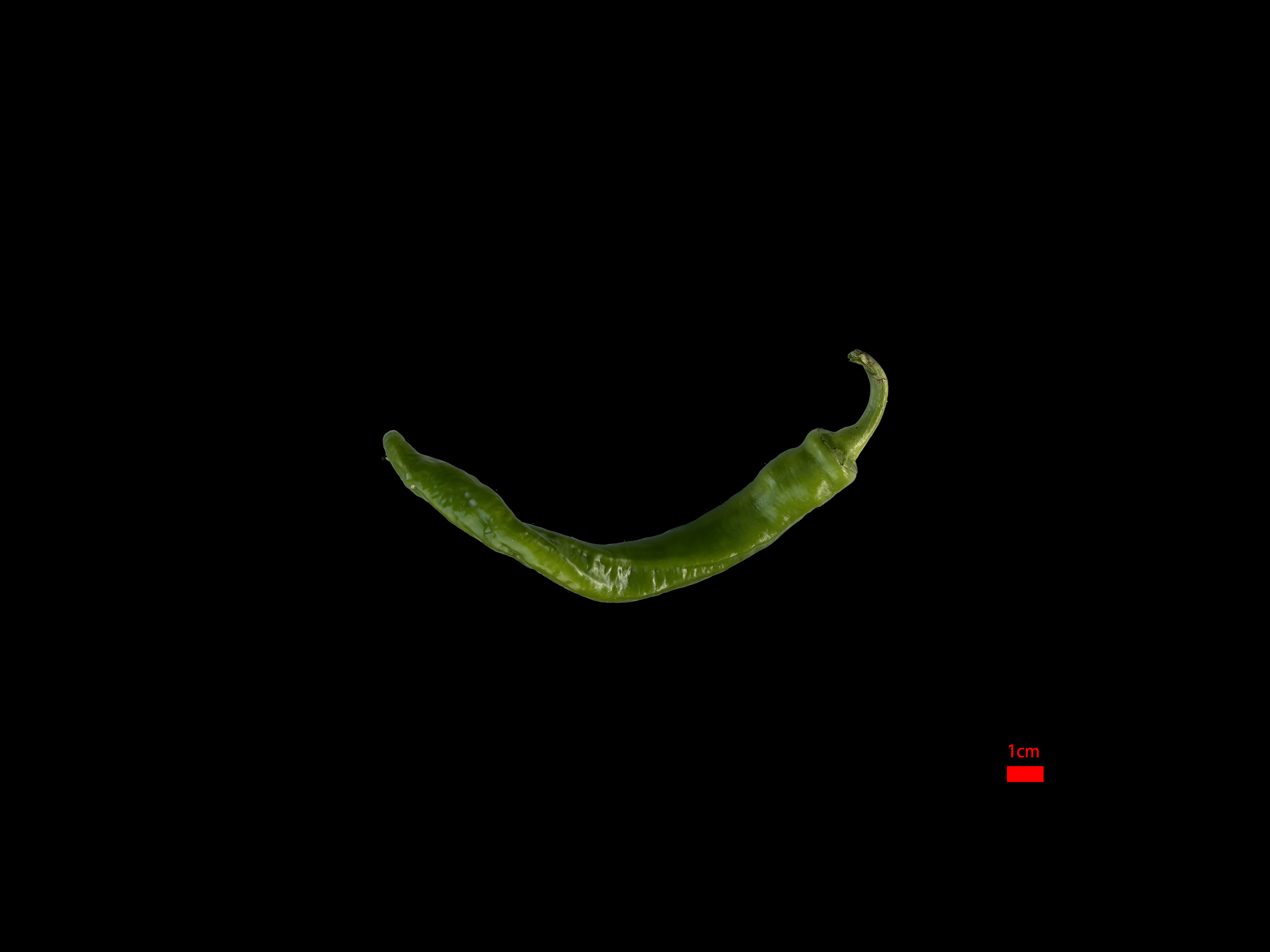

Supplement: Supplementary file 1 [file plants-15-02103-s001.zip › plants-4383327-supplementary/pepper_original_data/Goat_horn/101-2.jpg]

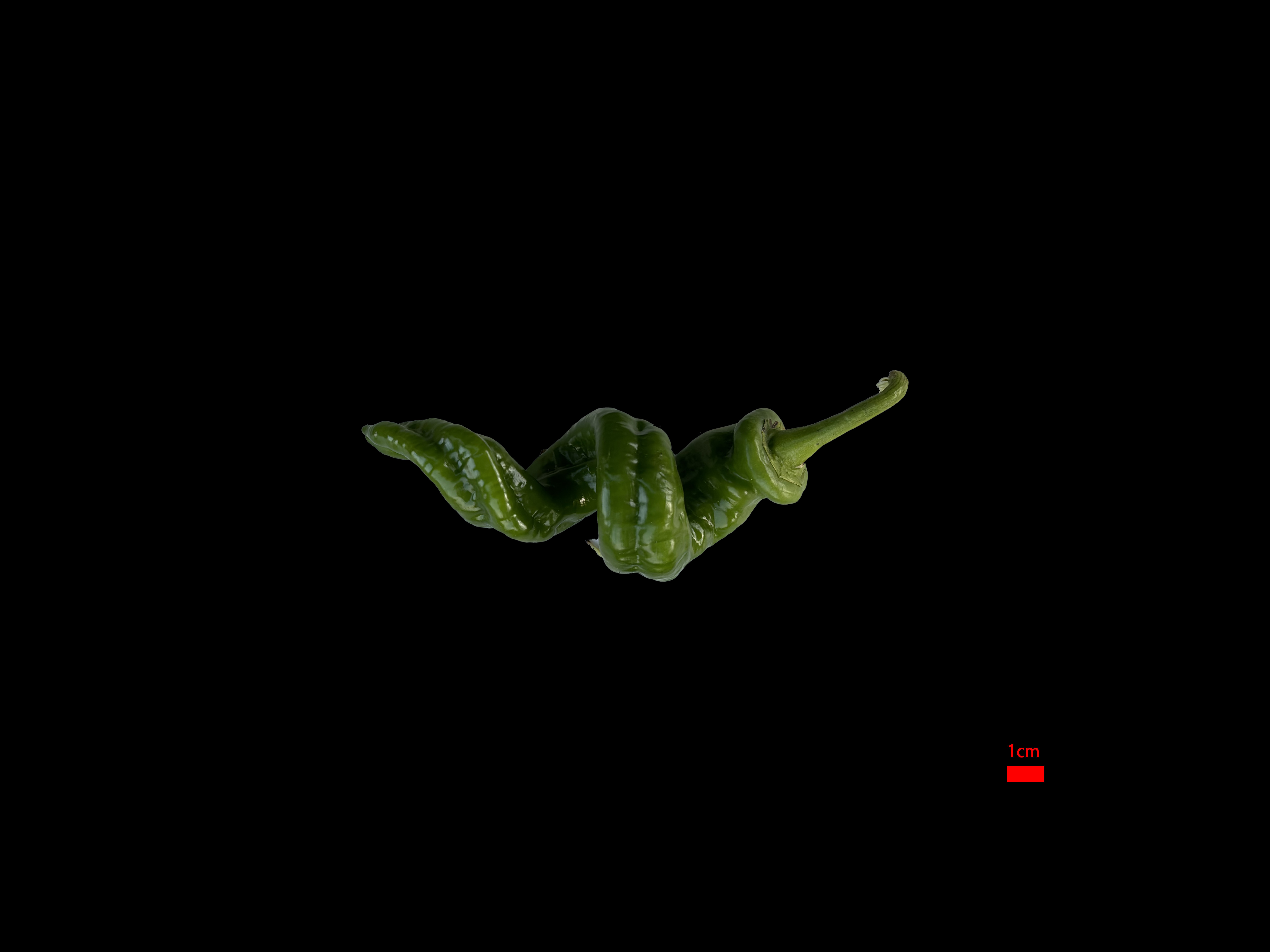

Supplement: Supplementary file 1 [file plants-15-02103-s001.zip › plants-4383327-supplementary/pepper_original_data/Goat_horn/101-3.jpg]

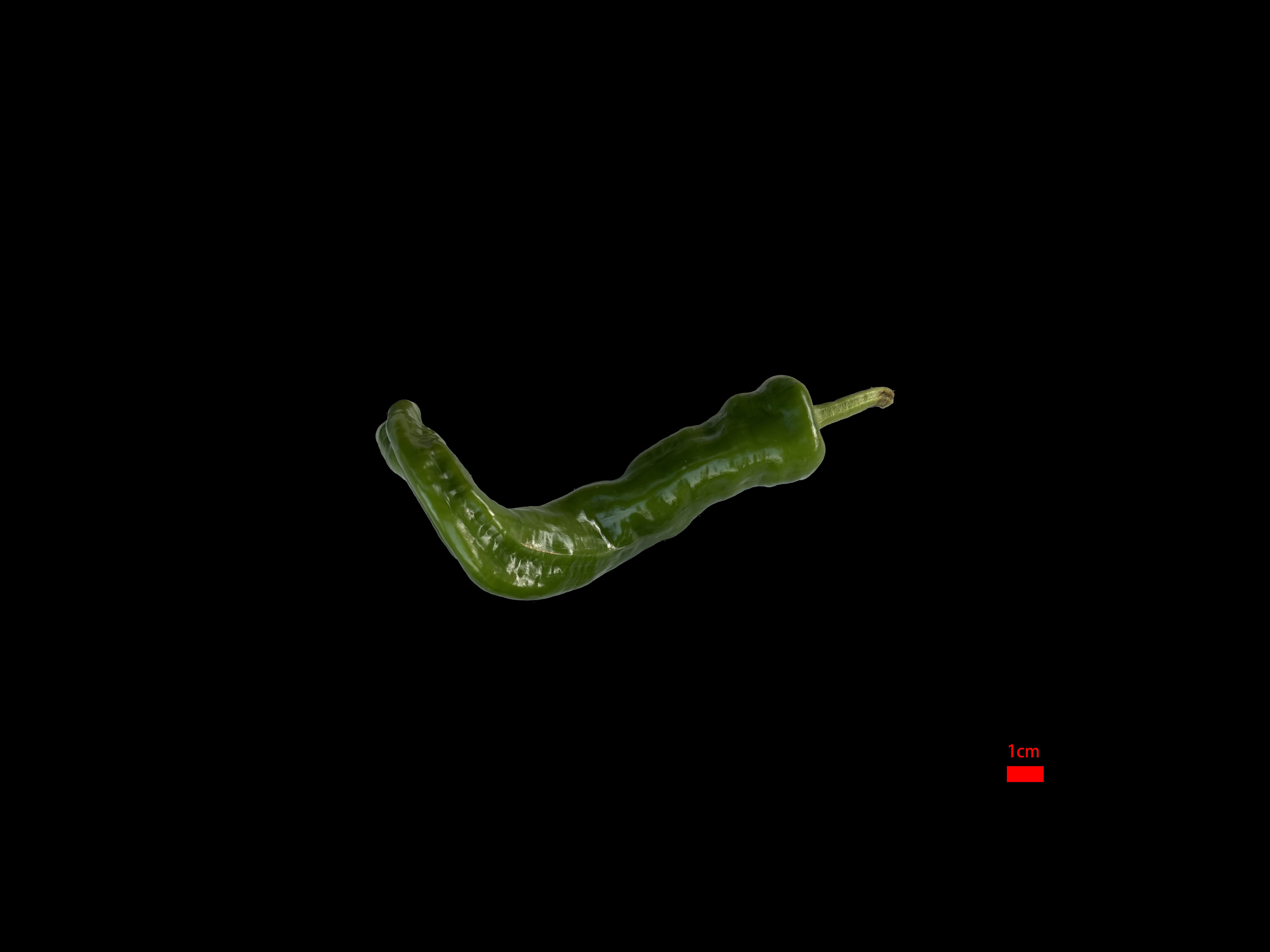

Supplement: Supplementary file 1 [file plants-15-02103-s001.zip › plants-4383327-supplementary/pepper_original_data/Goat_horn/101-4.jpg]

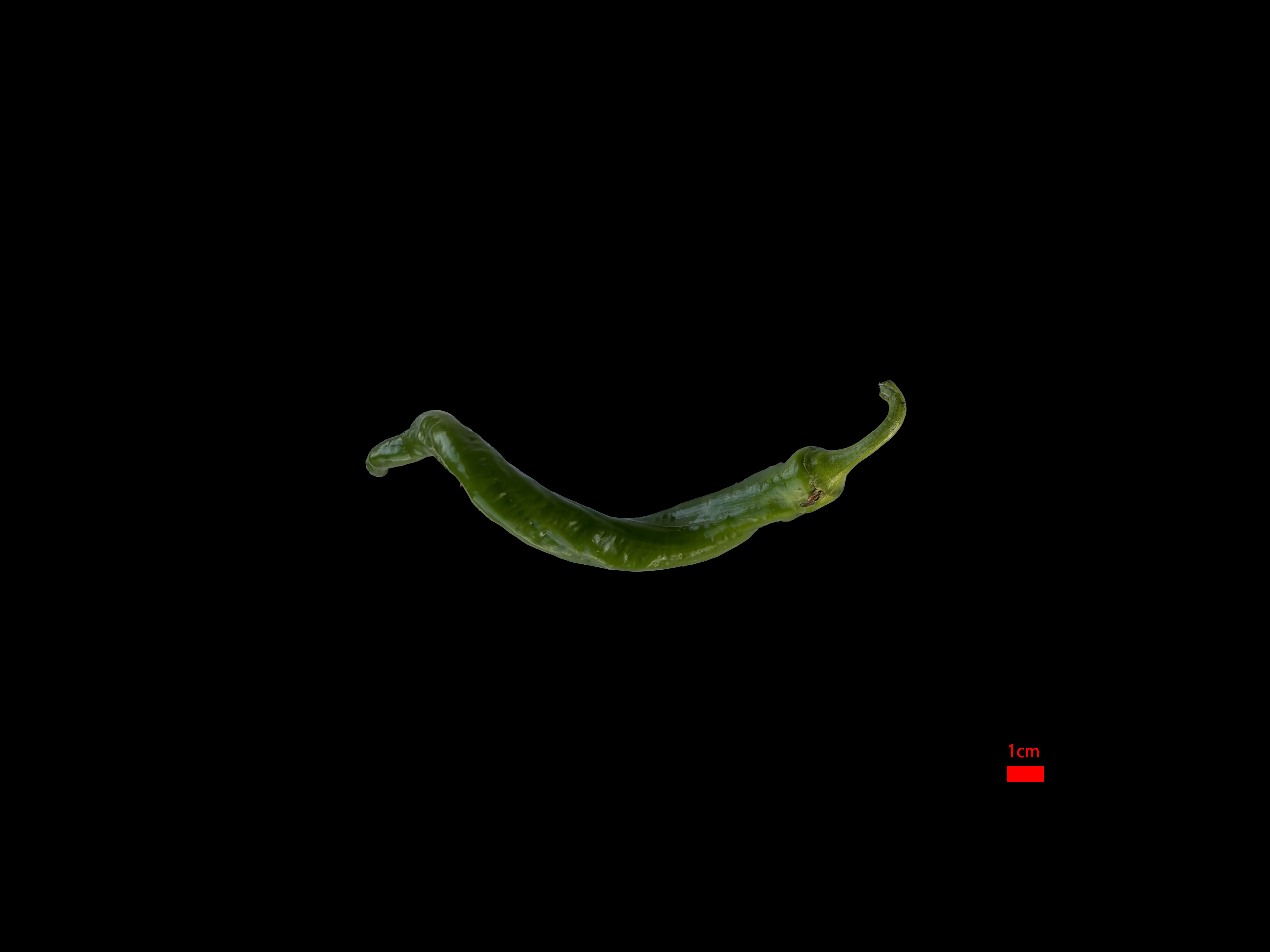

Supplement: Supplementary file 1 [file plants-15-02103-s001.zip › plants-4383327-supplementary/pepper_original_data/Goat_horn/101-5.jpg]

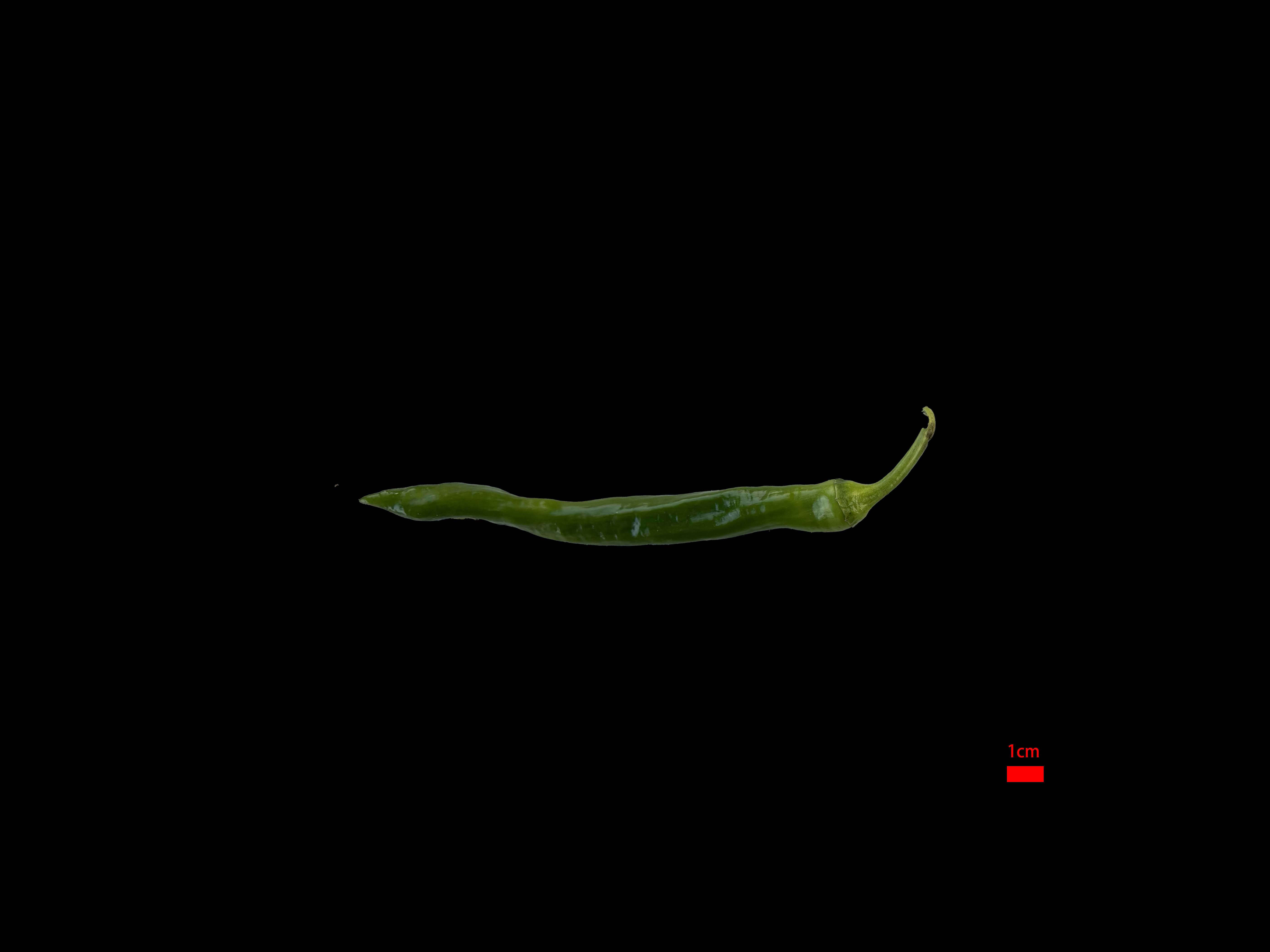

Supplement: Supplementary file 1 [file plants-15-02103-s001.zip › plants-4383327-supplementary/pepper_original_data/Goat_horn/101-6.jpg]

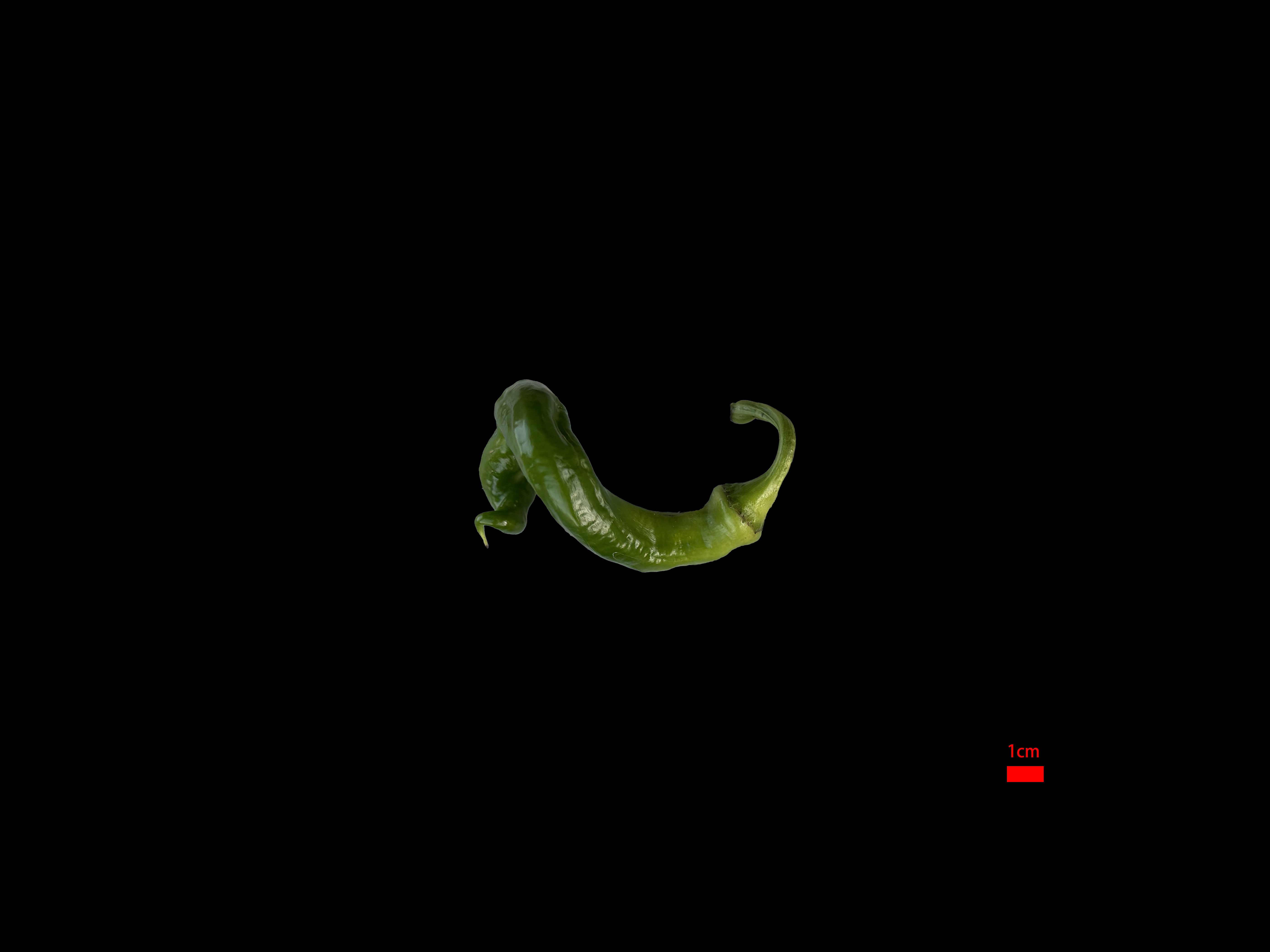

Supplement: Supplementary file 1 [file plants-15-02103-s001.zip › plants-4383327-supplementary/pepper_original_data/Goat_horn/101-7.jpg]

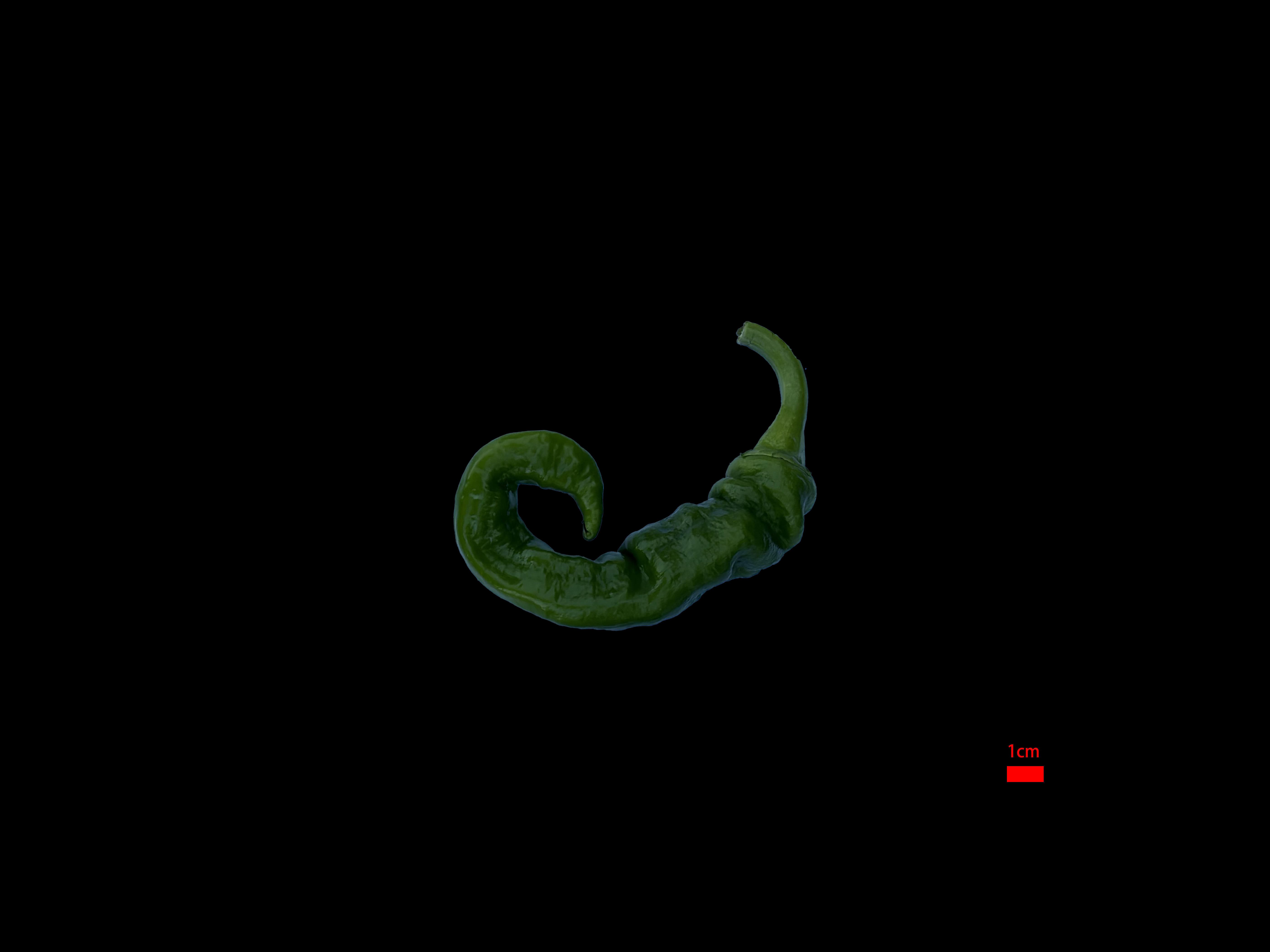

Supplement: Supplementary file 1 [file plants-15-02103-s001.zip › plants-4383327-supplementary/pepper_original_data/Goat_horn/102-1.jpg]

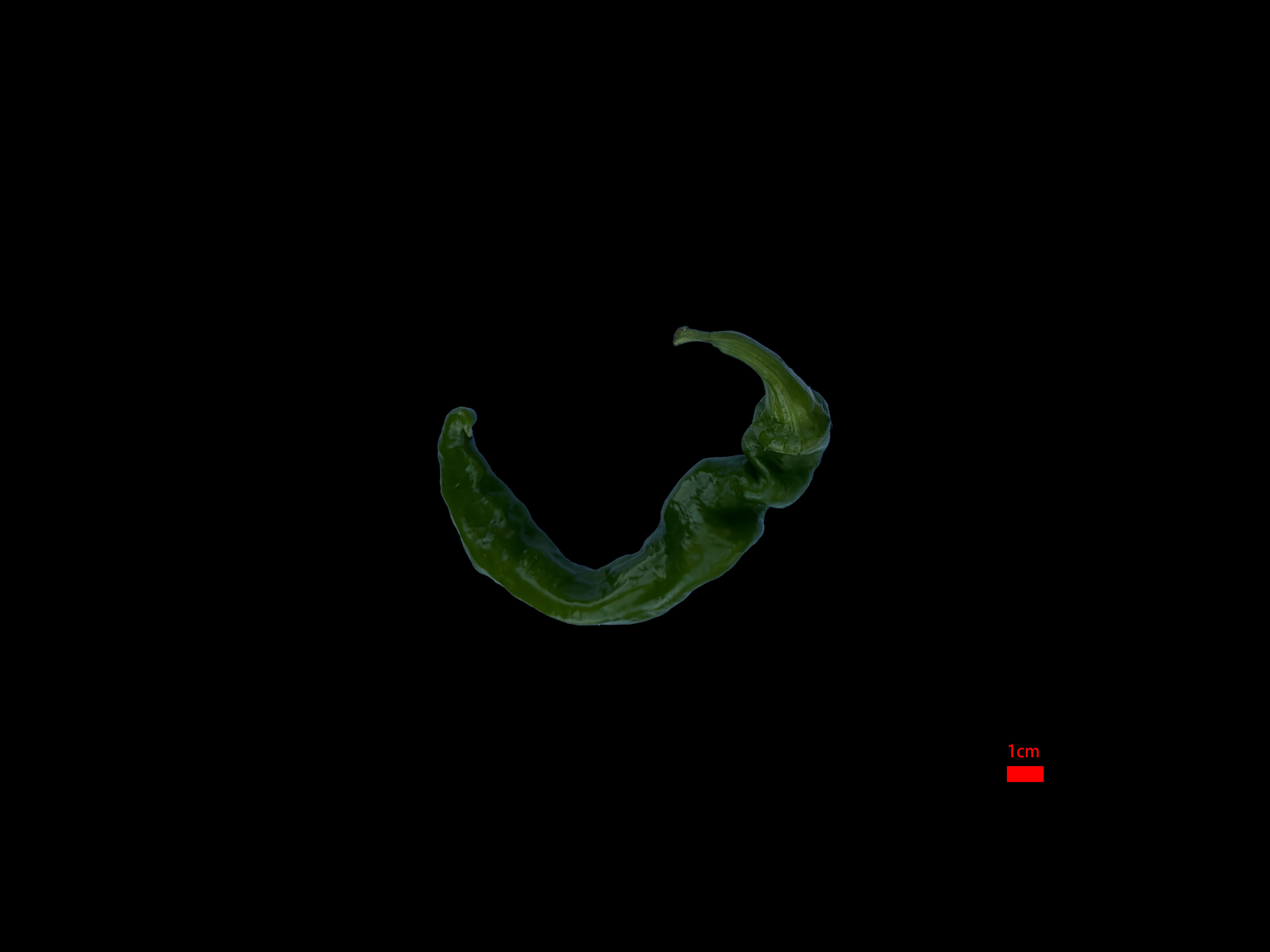

Supplement: Supplementary file 1 [file plants-15-02103-s001.zip › plants-4383327-supplementary/pepper_original_data/Goat_horn/102-10.jpg]

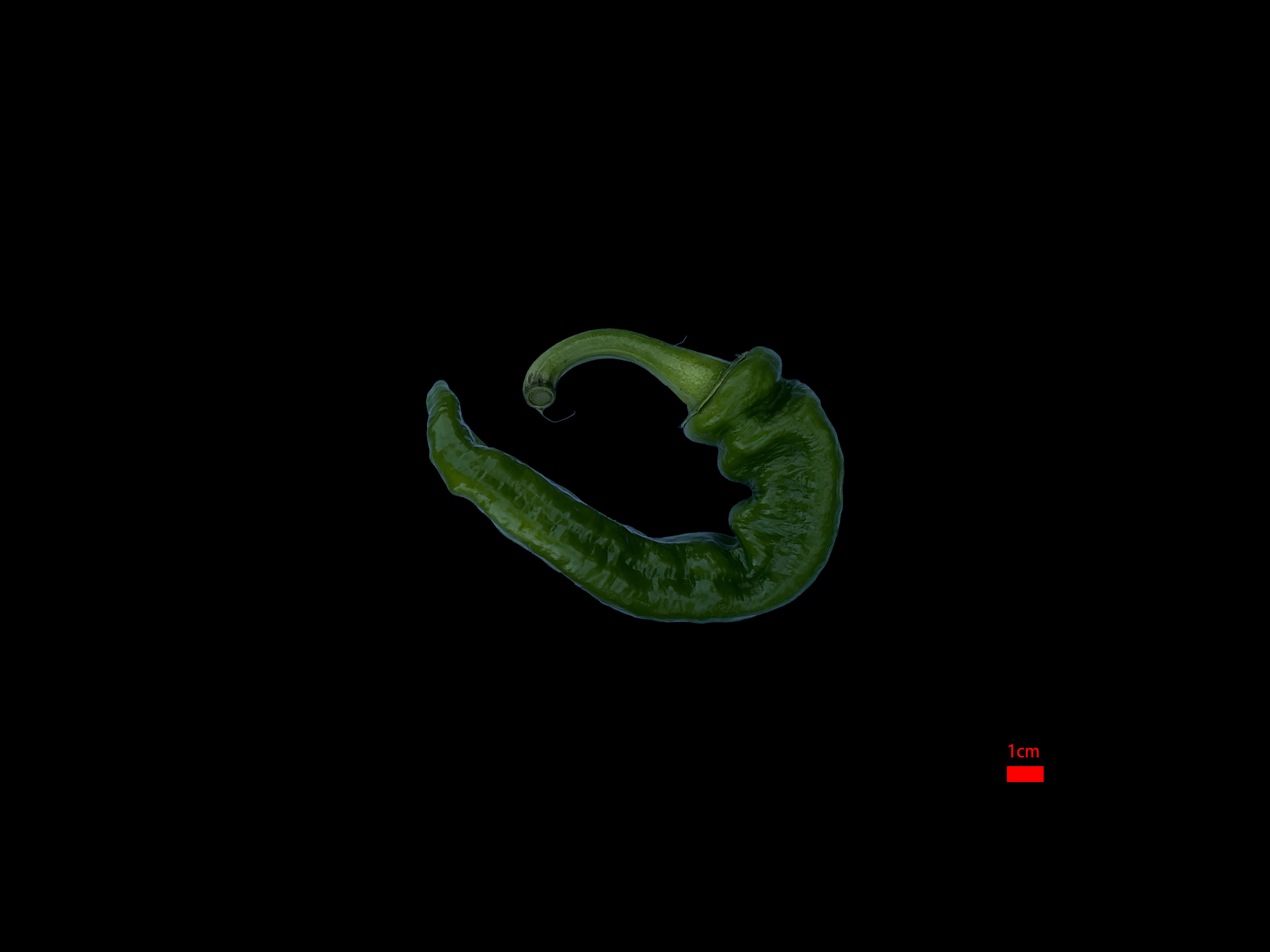

Supplement: Supplementary file 1 [file plants-15-02103-s001.zip › plants-4383327-supplementary/pepper_original_data/Goat_horn/102-2.jpg]

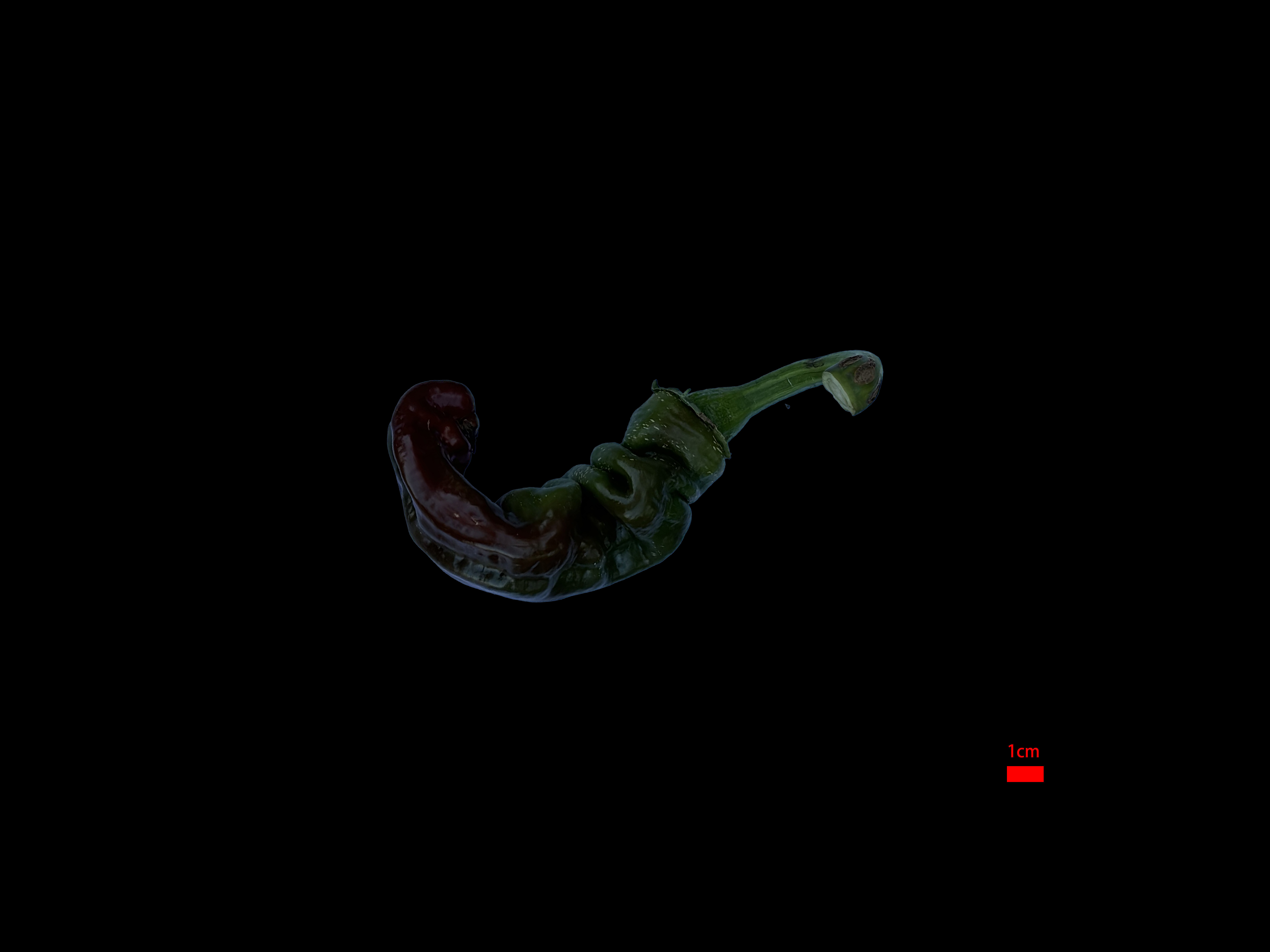

Supplement: Supplementary file 1 [file plants-15-02103-s001.zip › plants-4383327-supplementary/pepper_original_data/Goat_horn/102-3.jpg]

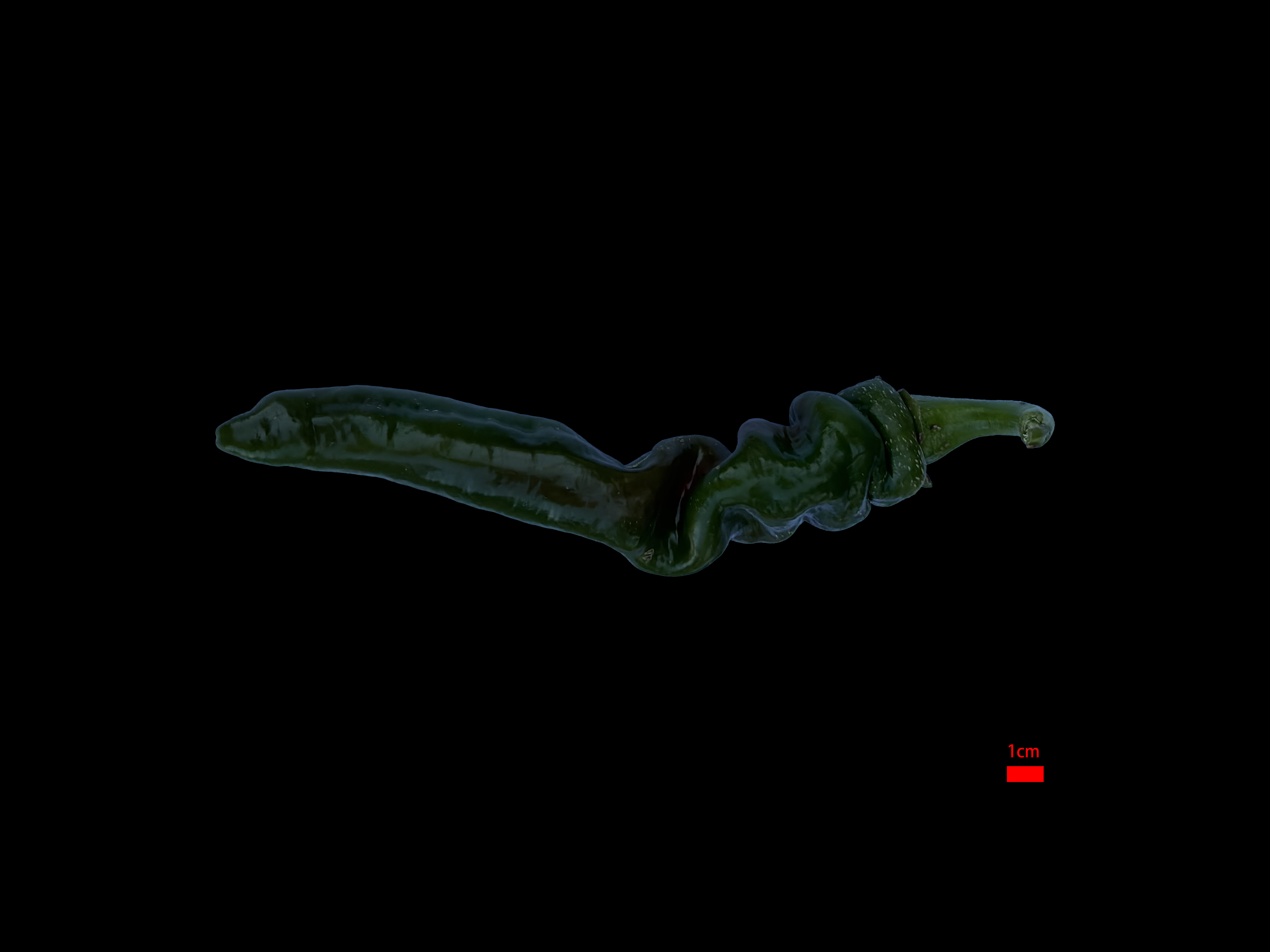

Supplement: Supplementary file 1 [file plants-15-02103-s001.zip › plants-4383327-supplementary/pepper_original_data/Goat_horn/102-4.jpg]

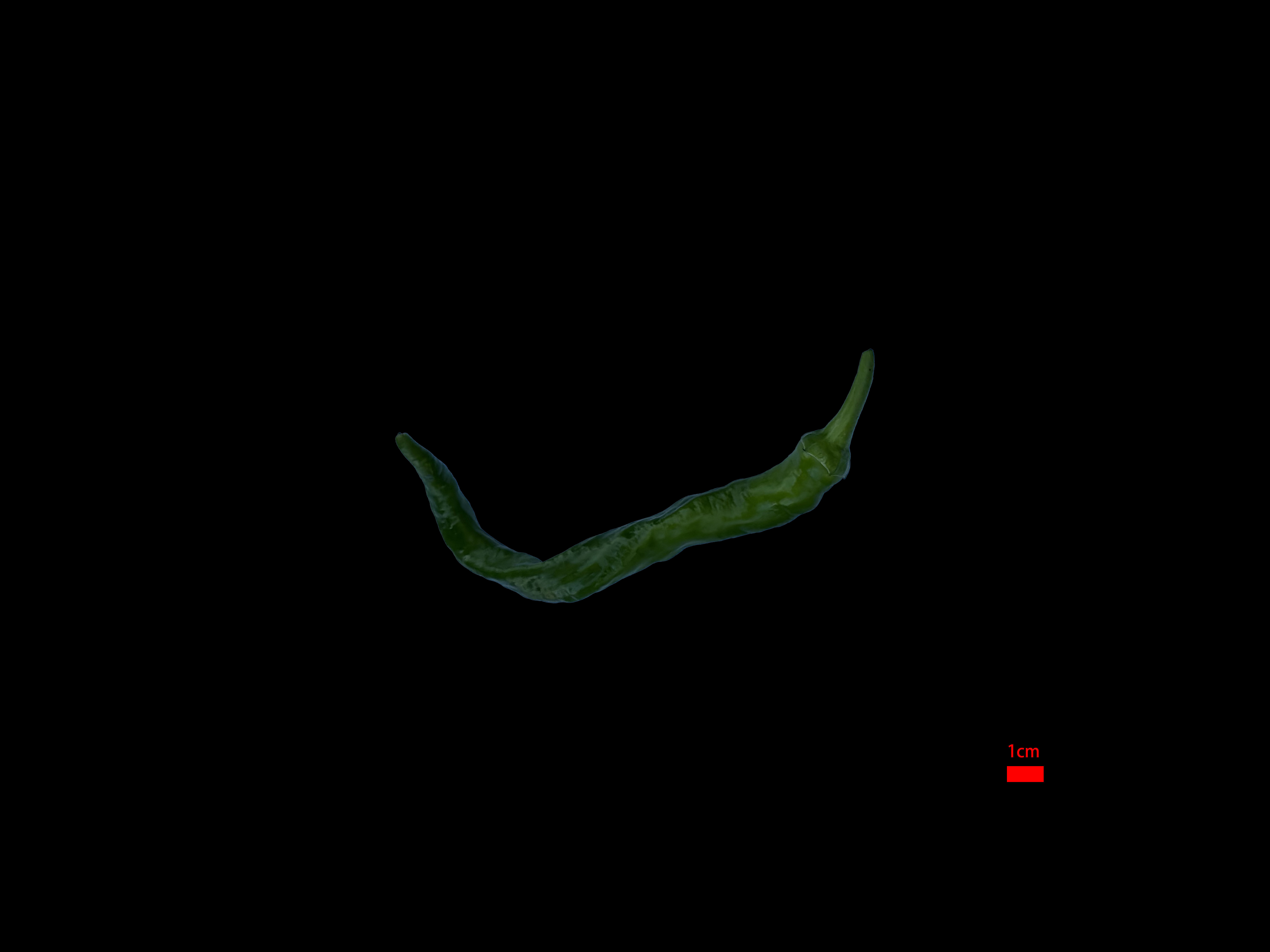

Supplement: Supplementary file 1 [file plants-15-02103-s001.zip › plants-4383327-supplementary/pepper_original_data/Goat_horn/102-8.jpg]

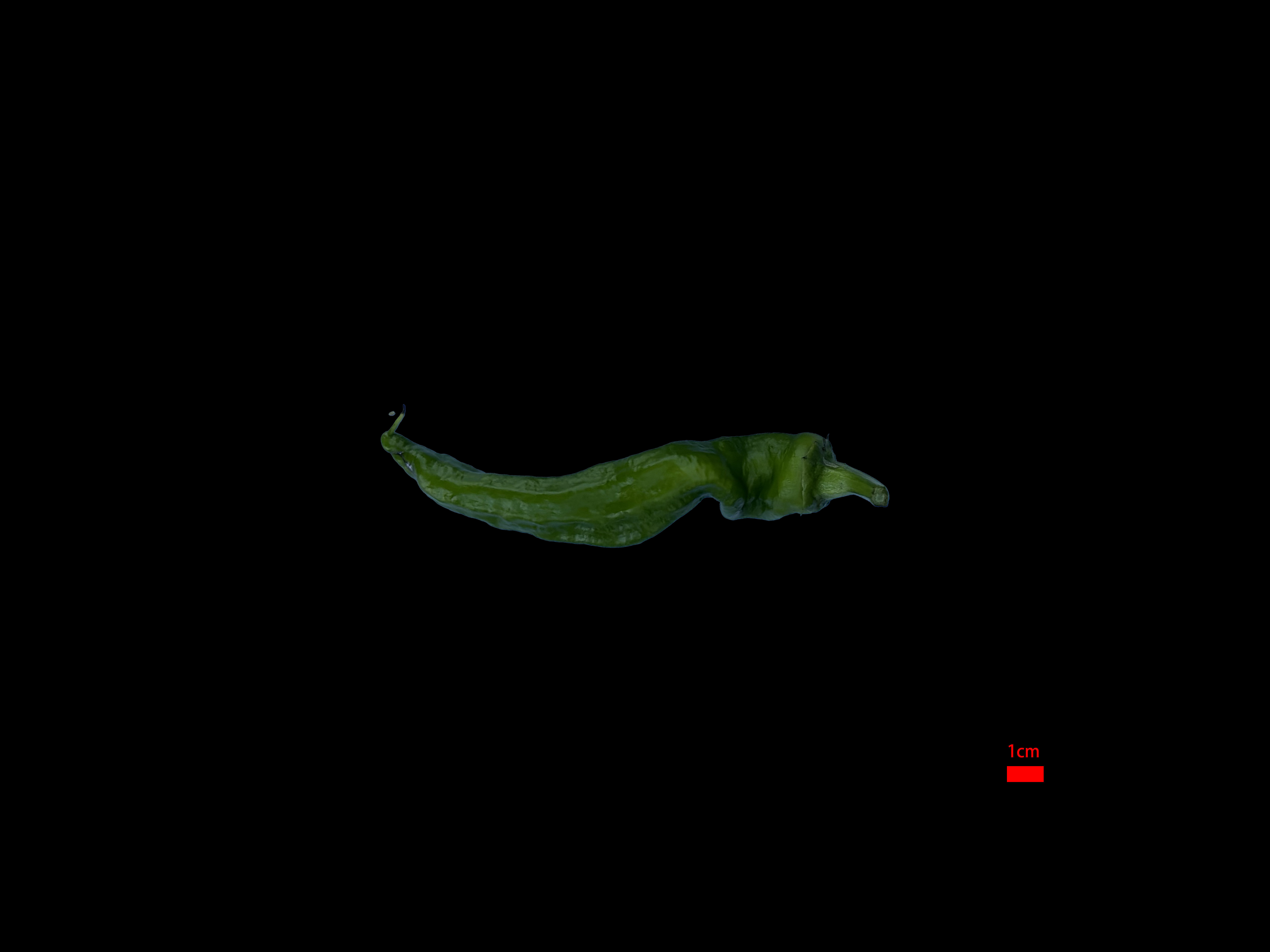

Supplement: Supplementary file 1 [file plants-15-02103-s001.zip › plants-4383327-supplementary/pepper_original_data/Goat_horn/102-9.jpg]

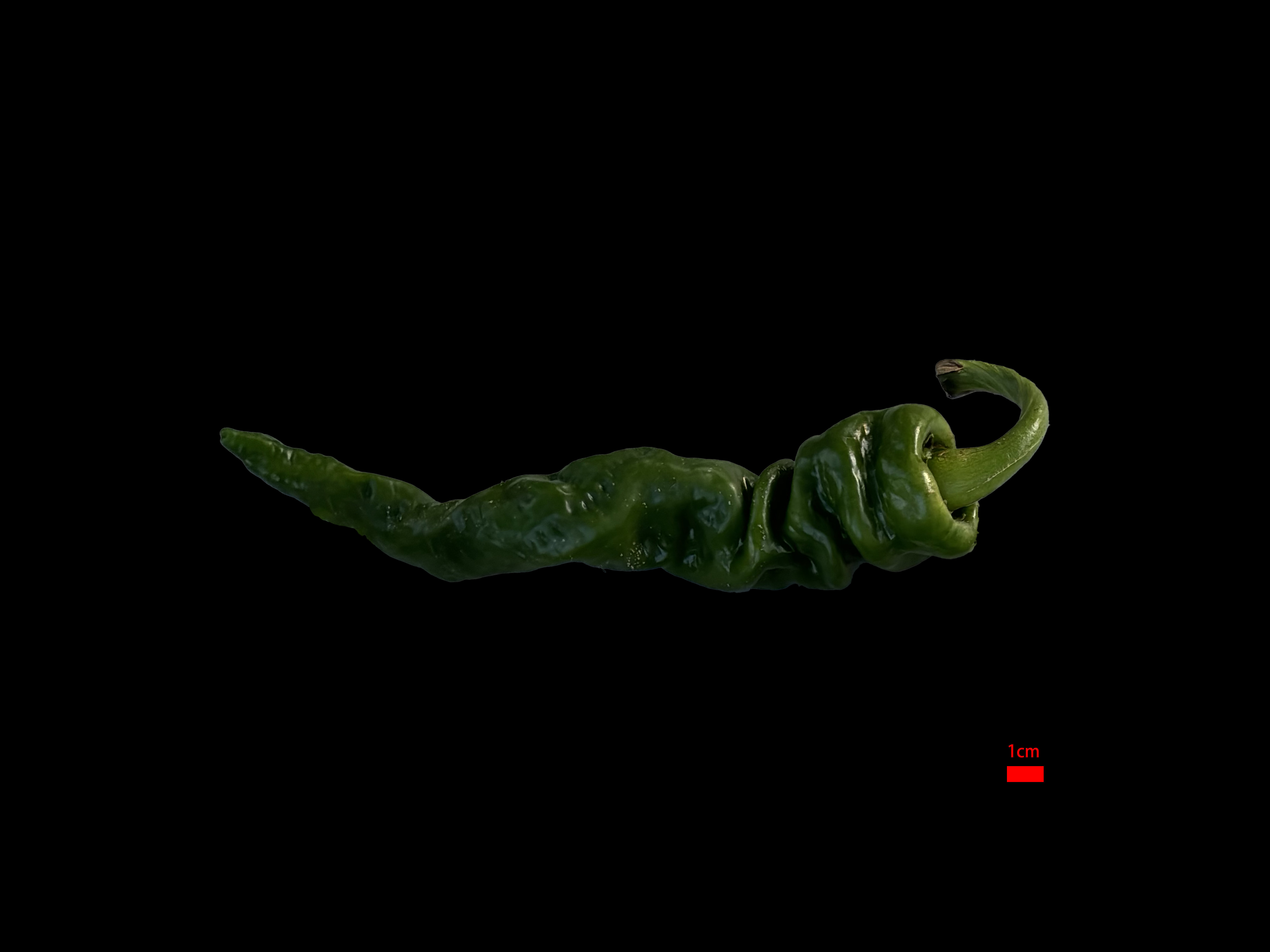

Supplement: Supplementary file 1 [file plants-15-02103-s001.zip › plants-4383327-supplementary/pepper_original_data/Goat_horn/103-1.jpg]

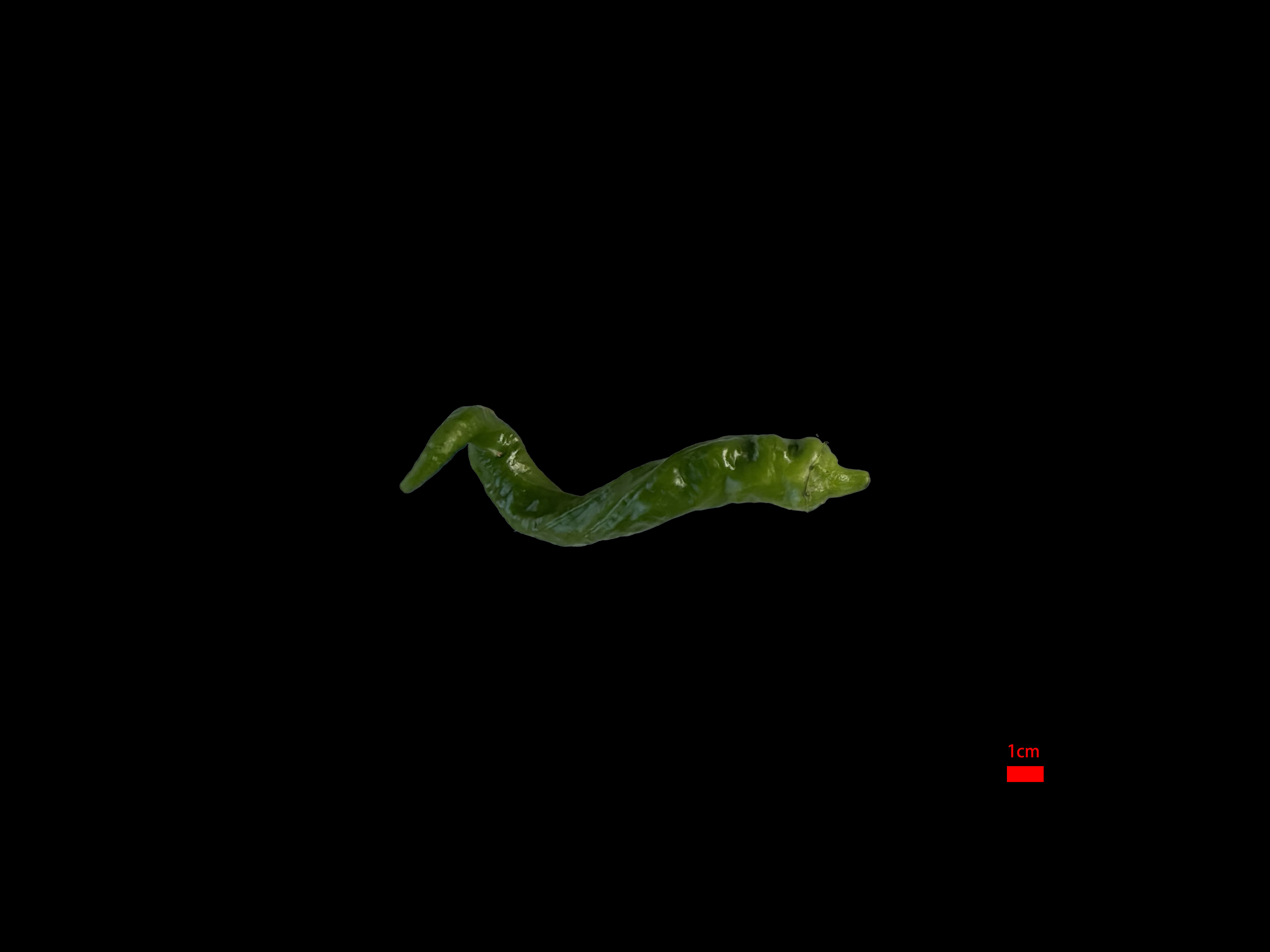

Supplement: Supplementary file 1 [file plants-15-02103-s001.zip › plants-4383327-supplementary/pepper_original_data/Goat_horn/103-11.jpg]

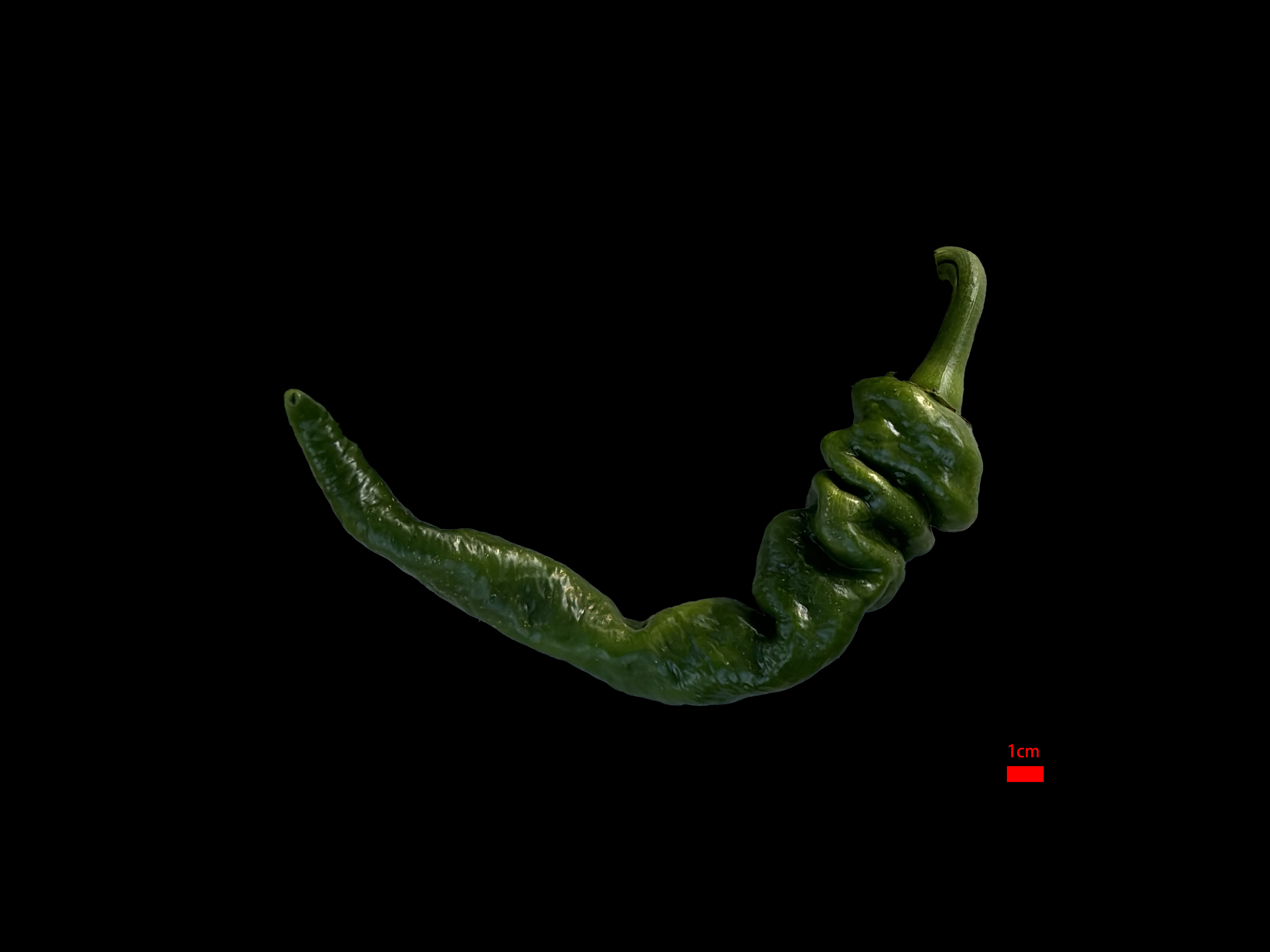

Supplement: Supplementary file 1 [file plants-15-02103-s001.zip › plants-4383327-supplementary/pepper_original_data/Goat_horn/103-2.jpg]

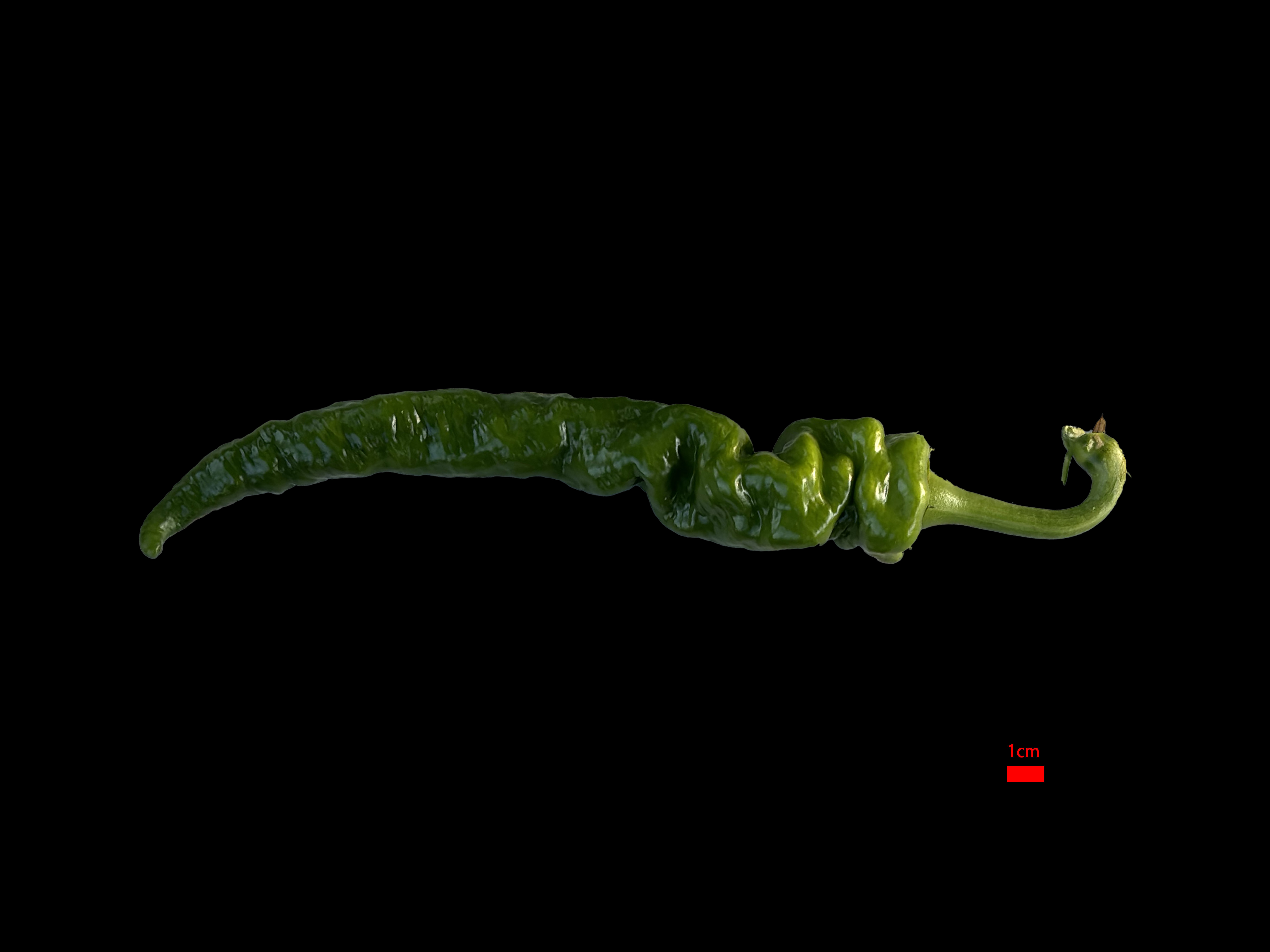

Supplement: Supplementary file 1 [file plants-15-02103-s001.zip › plants-4383327-supplementary/pepper_original_data/Goat_horn/103-3.jpg]

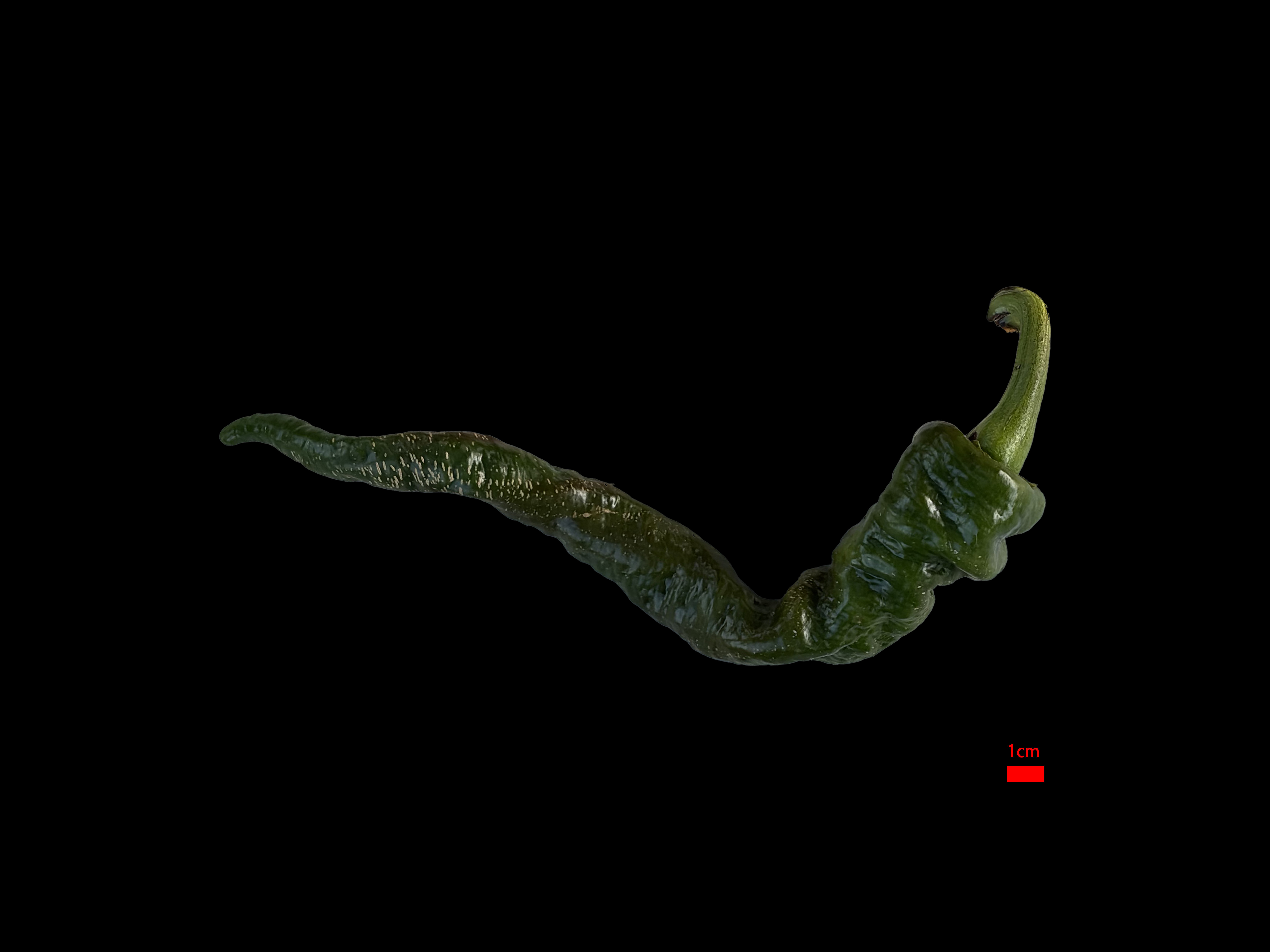

Supplement: Supplementary file 1 [file plants-15-02103-s001.zip › plants-4383327-supplementary/pepper_original_data/Goat_horn/103-4.jpg]

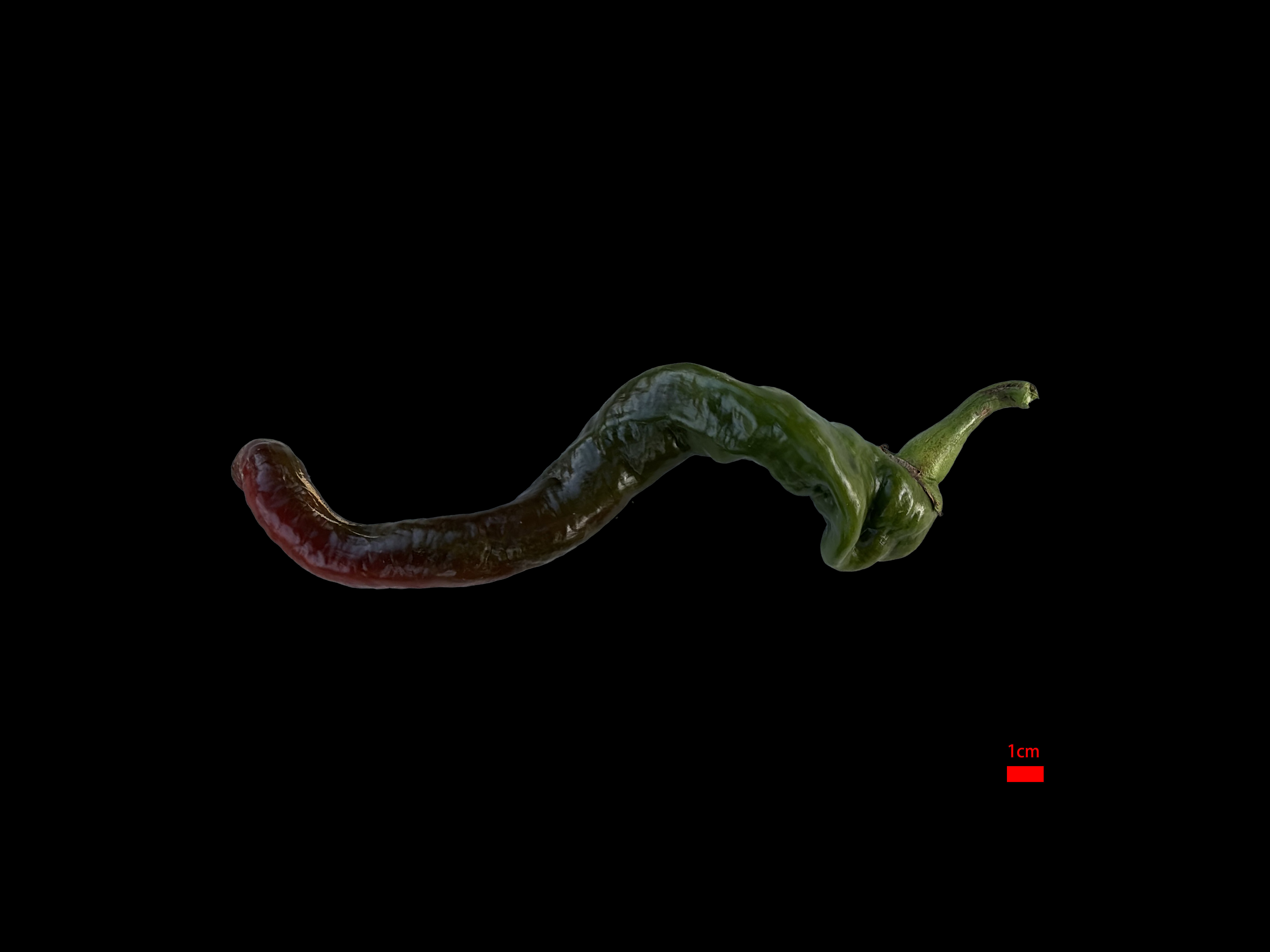

Supplement: Supplementary file 1 [file plants-15-02103-s001.zip › plants-4383327-supplementary/pepper_original_data/Goat_horn/103-5.jpg]

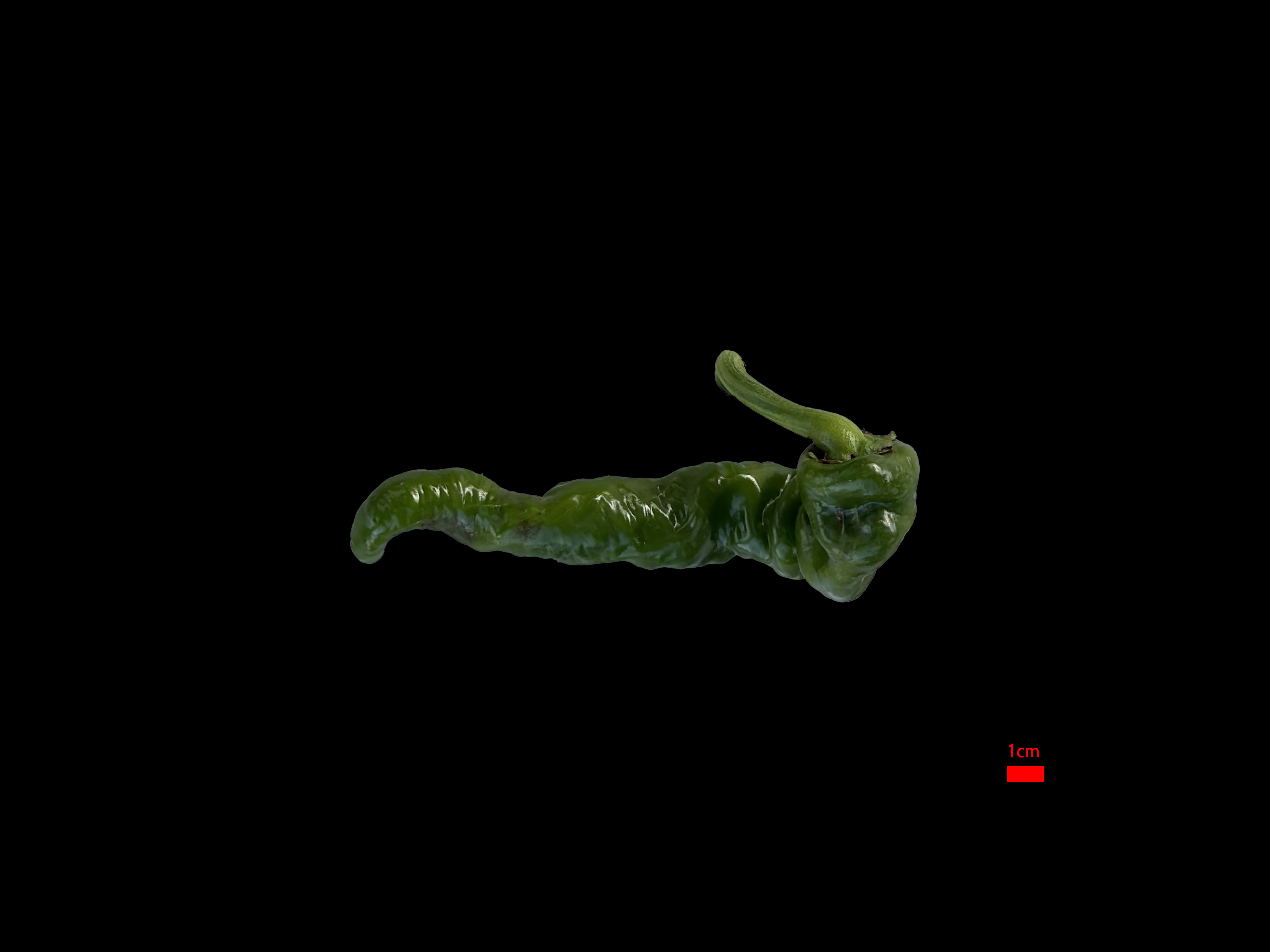

Supplement: Supplementary file 1 [file plants-15-02103-s001.zip › plants-4383327-supplementary/pepper_original_data/Goat_horn/103-6.jpg]

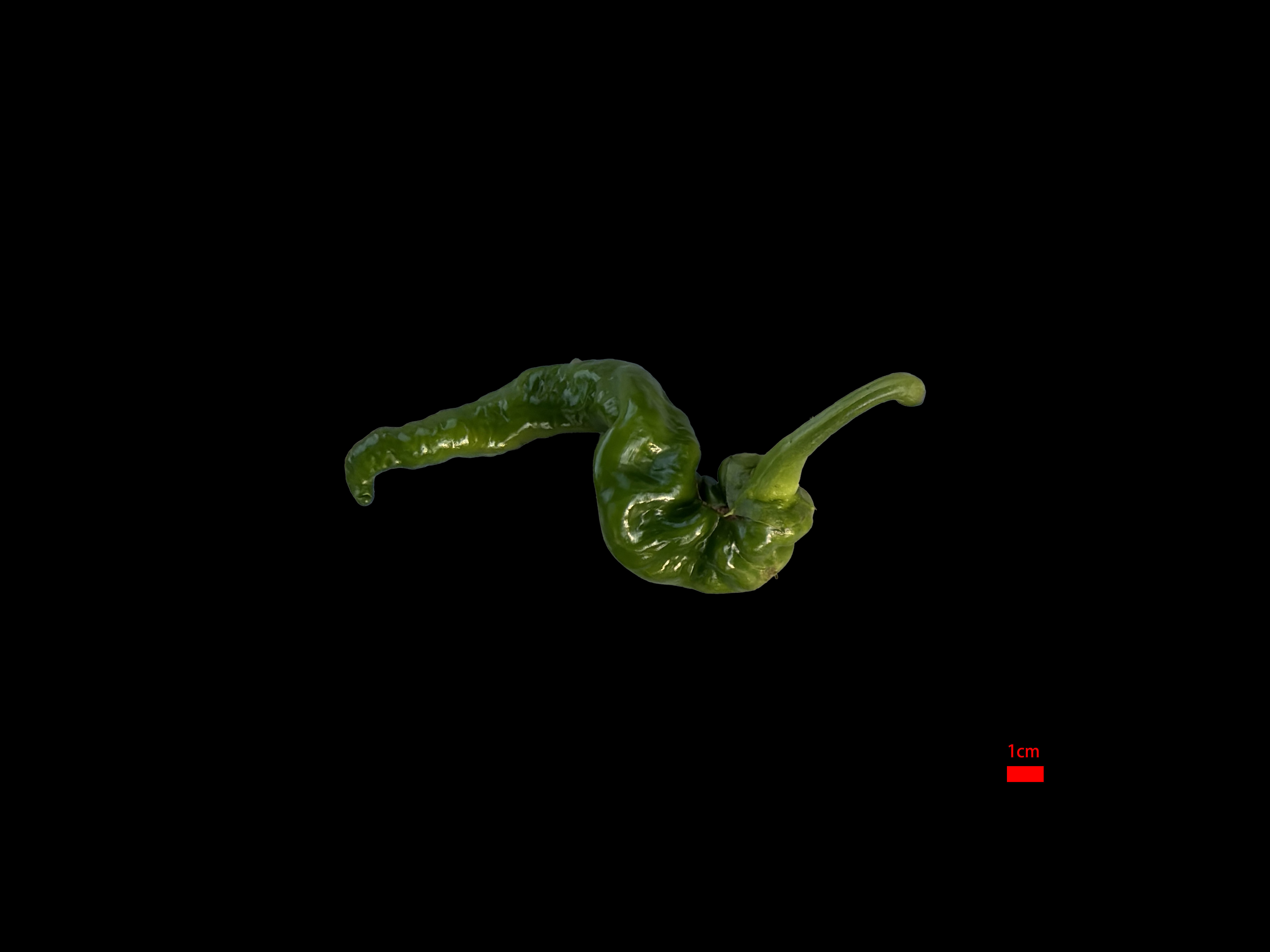

Supplement: Supplementary file 1 [file plants-15-02103-s001.zip › plants-4383327-supplementary/pepper_original_data/Goat_horn/103-9.jpg]

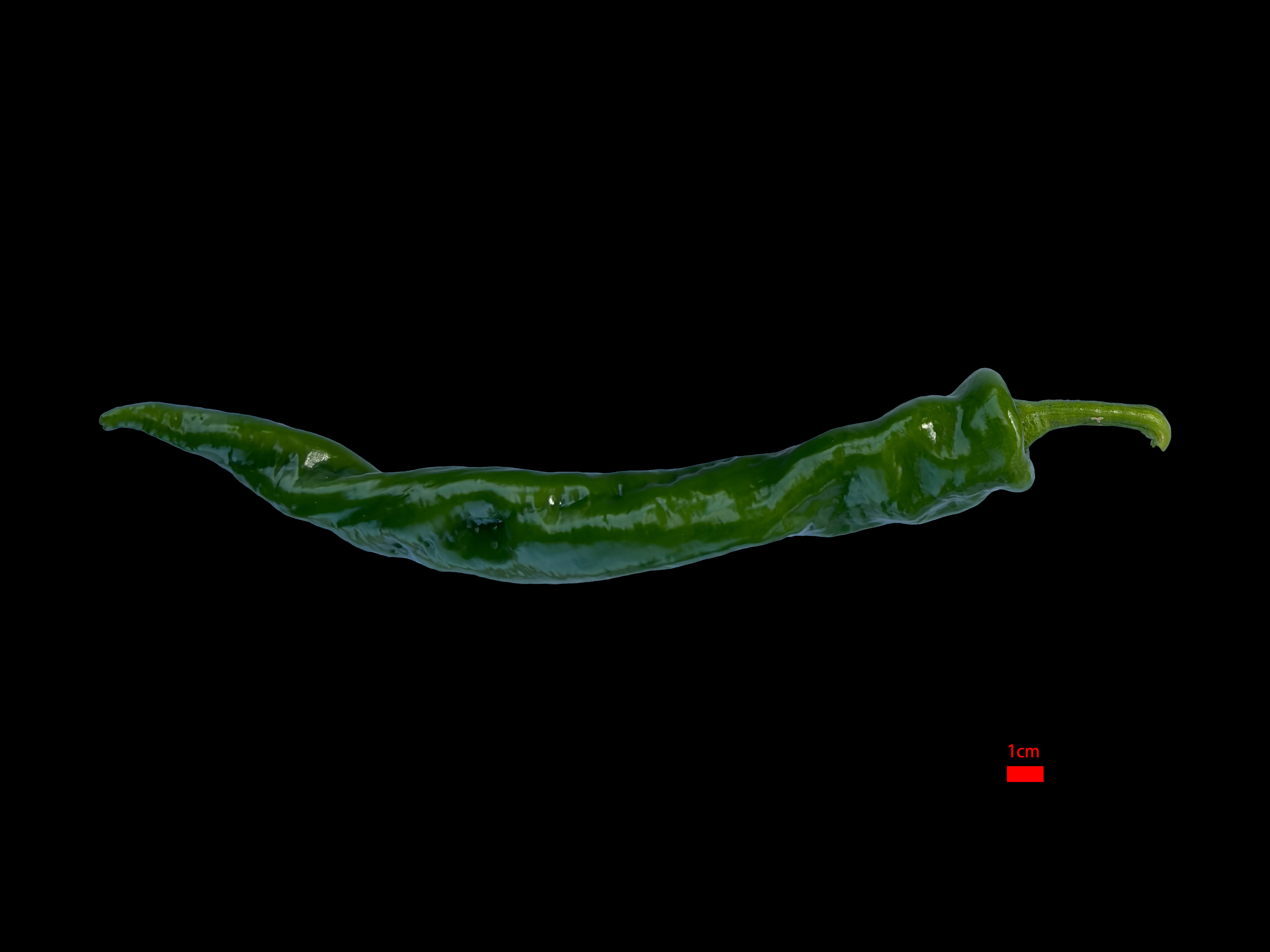

Supplement: Supplementary file 1 [file plants-15-02103-s001.zip › plants-4383327-supplementary/pepper_original_data/Goat_horn/104-1.jpg]

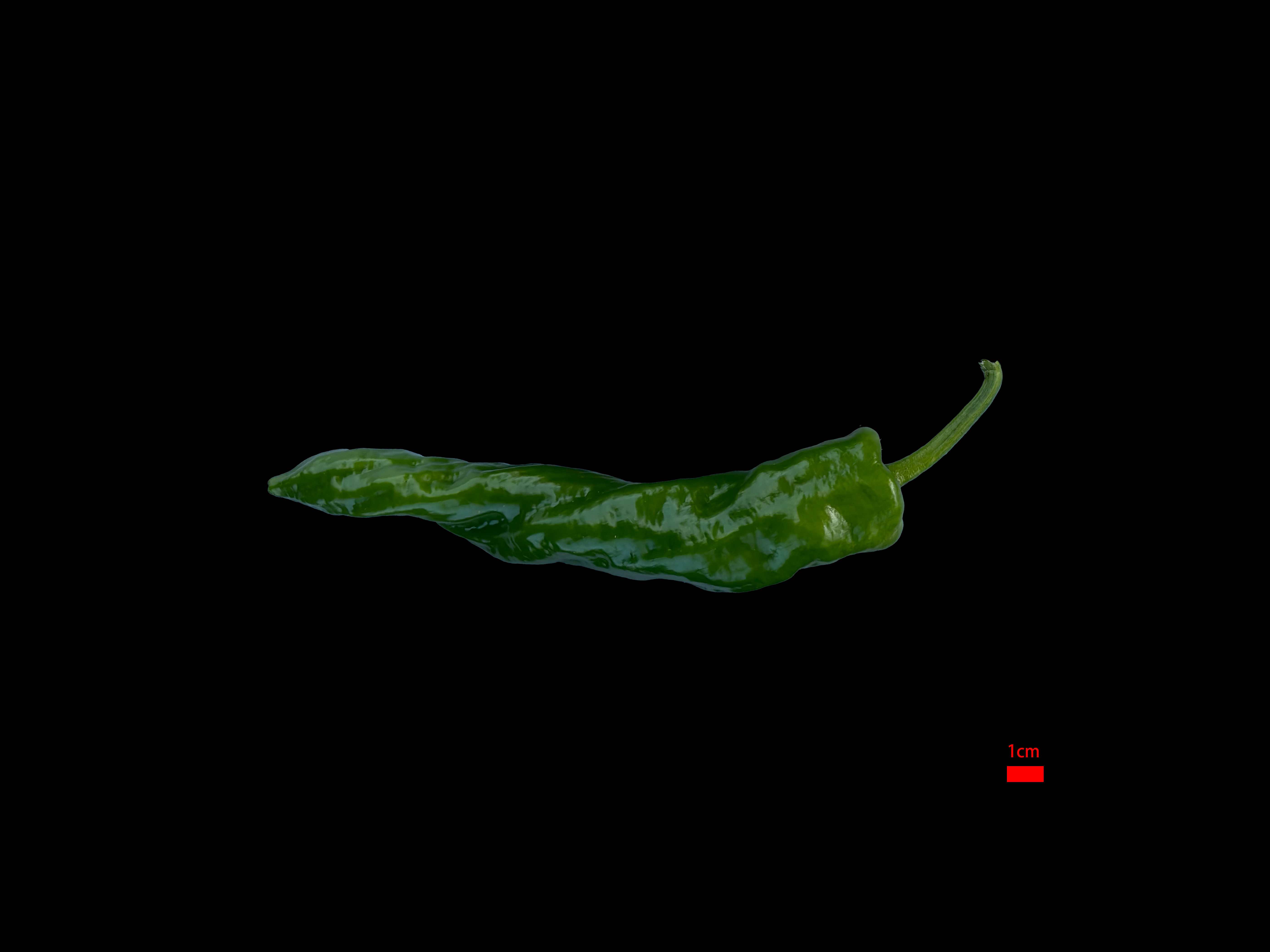

Supplement: Supplementary file 1 [file plants-15-02103-s001.zip › plants-4383327-supplementary/pepper_original_data/Goat_horn/104-2.jpg]

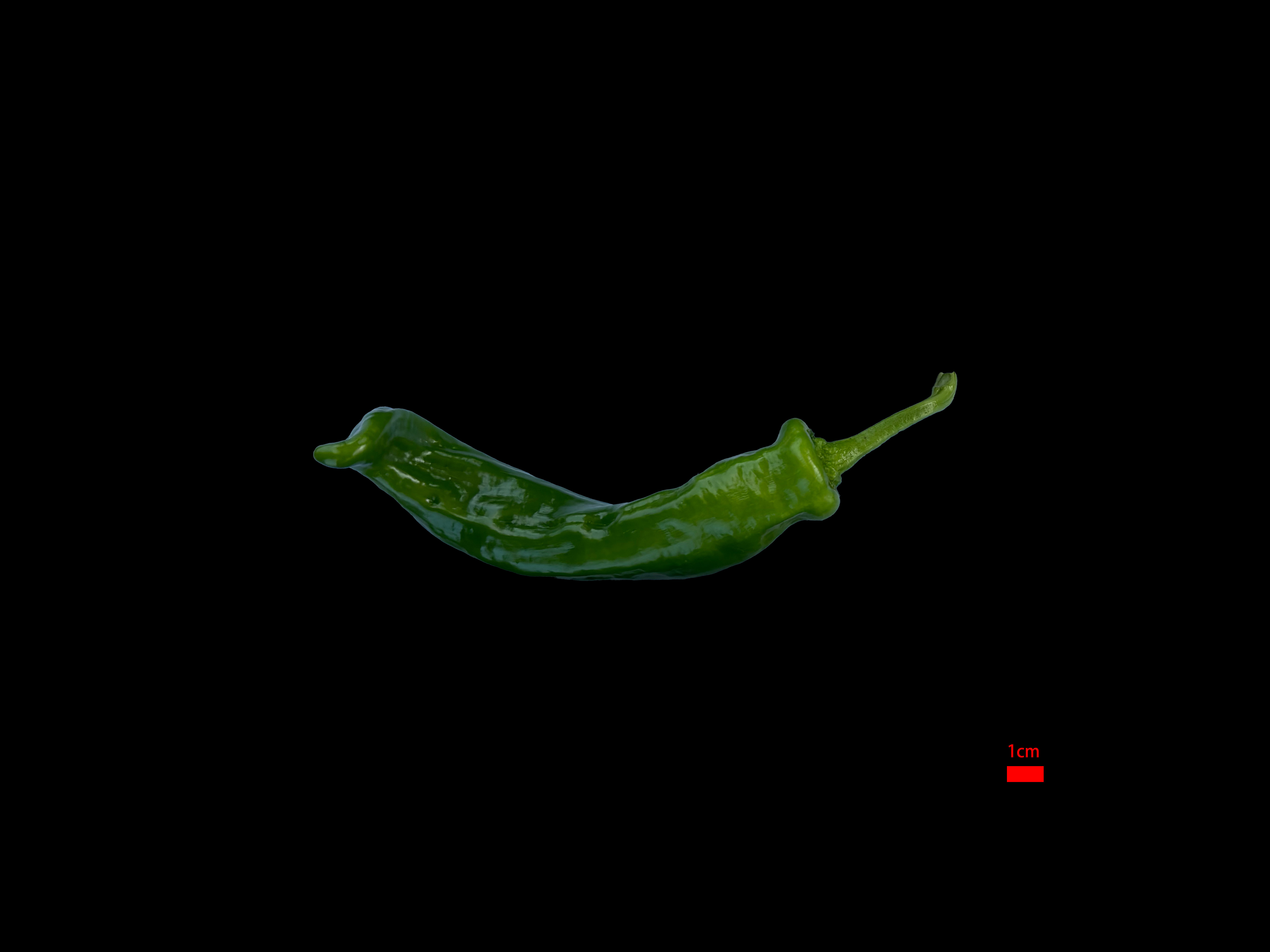

Supplement: Supplementary file 1 [file plants-15-02103-s001.zip › plants-4383327-supplementary/pepper_original_data/Goat_horn/104-3.jpg]

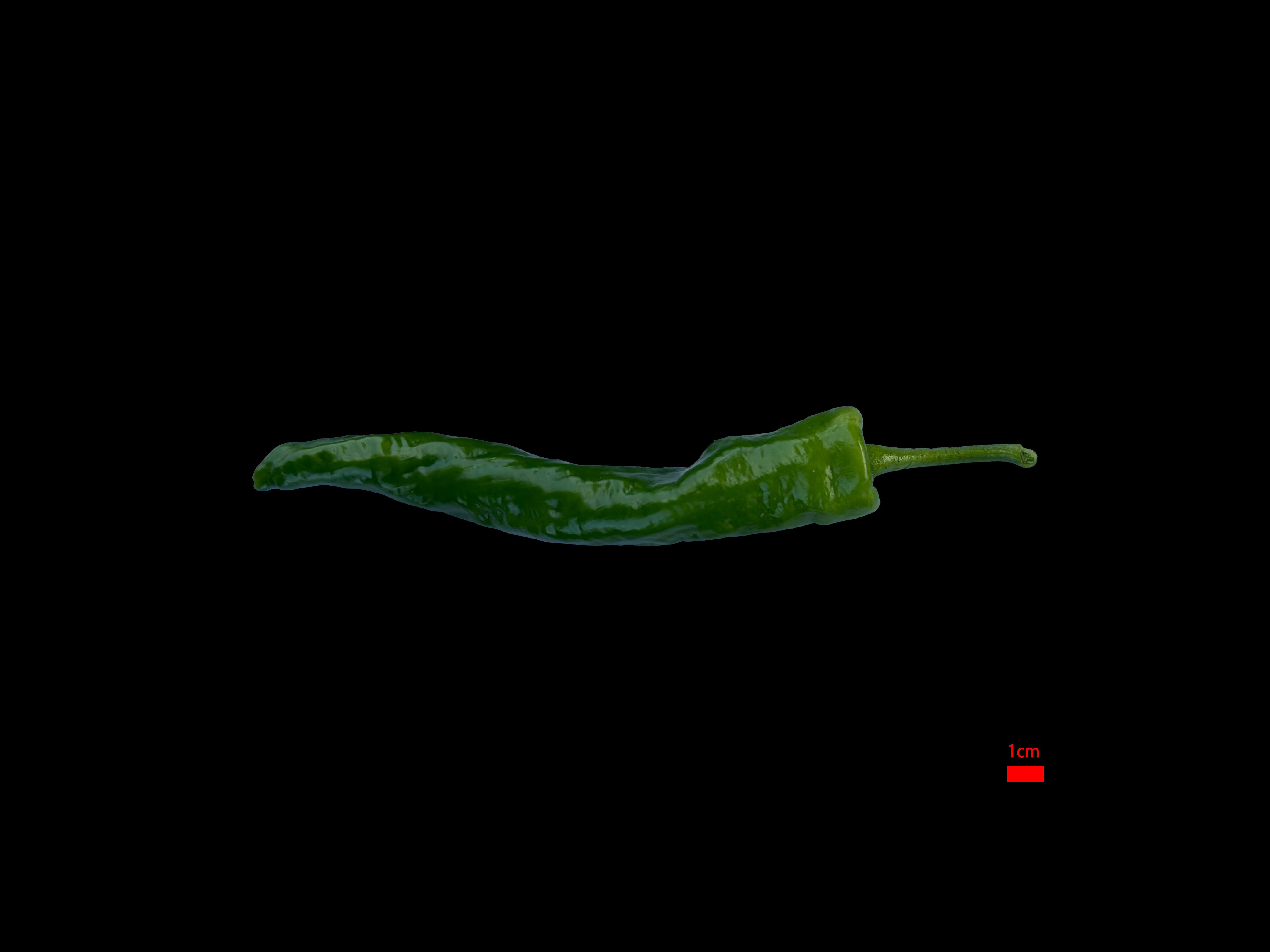

Supplement: Supplementary file 1 [file plants-15-02103-s001.zip › plants-4383327-supplementary/pepper_original_data/Goat_horn/104-4.jpg]

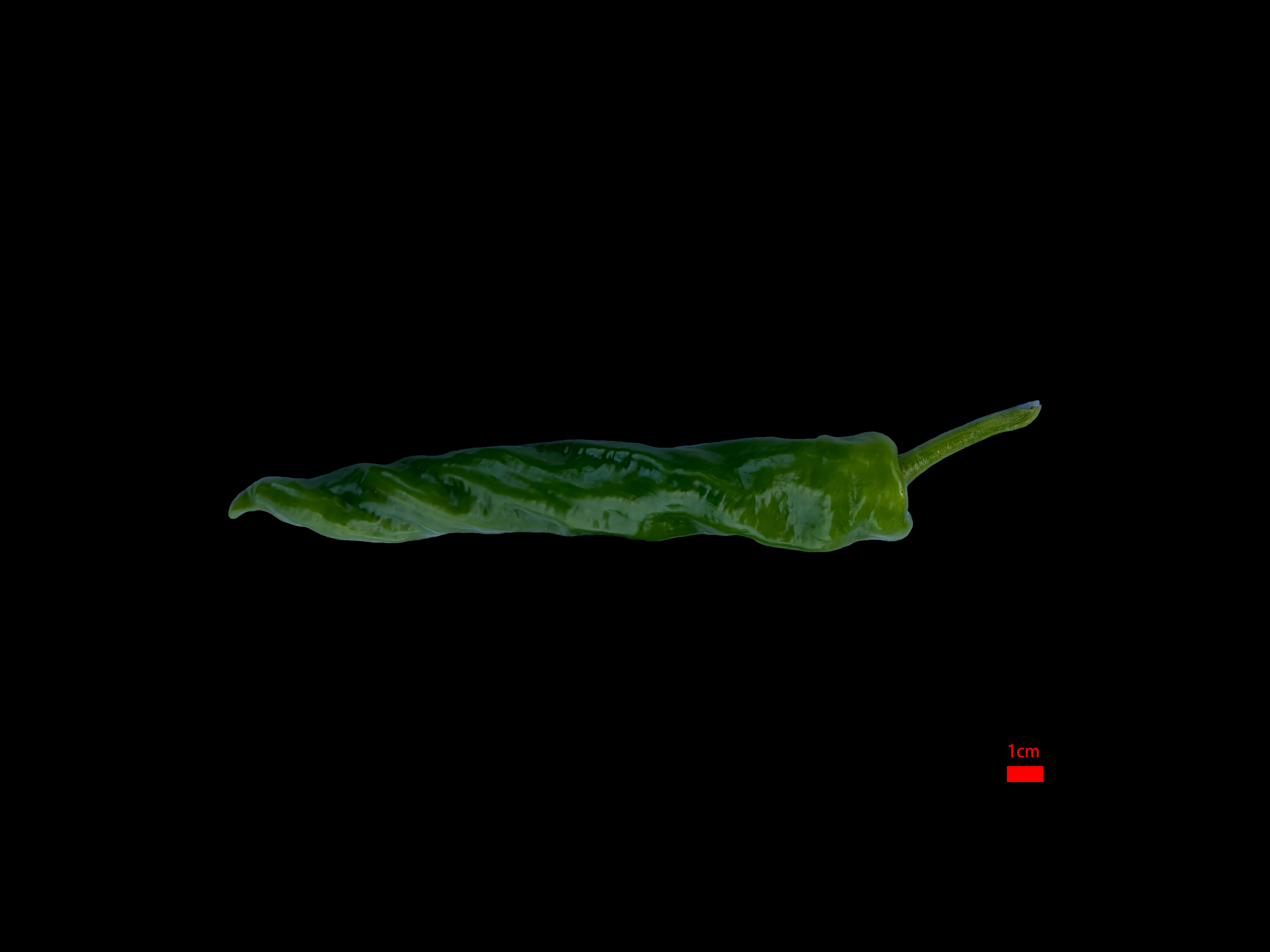

Supplement: Supplementary file 1 [file plants-15-02103-s001.zip › plants-4383327-supplementary/pepper_original_data/Goat_horn/104-5.jpg]

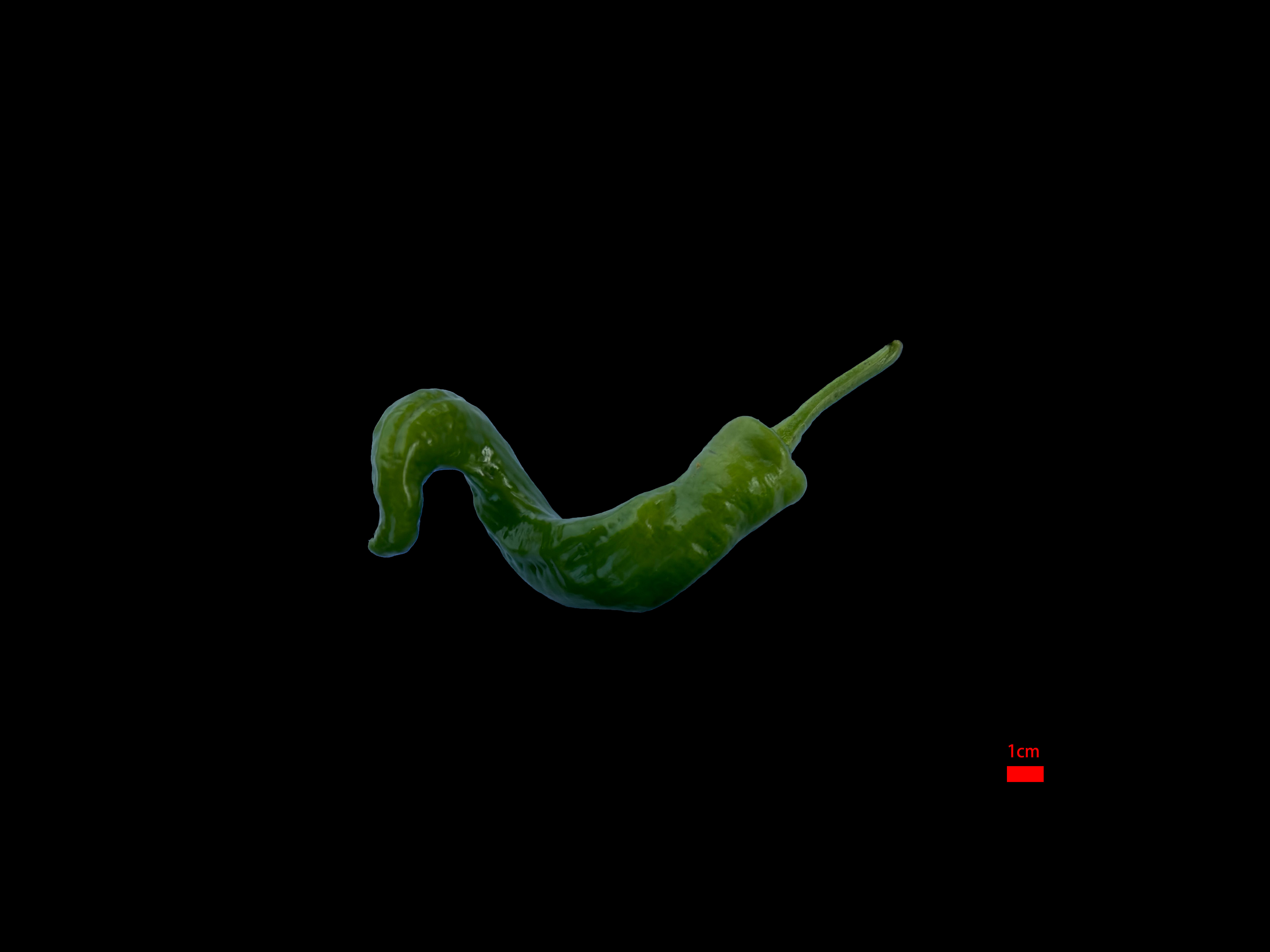

Supplement: Supplementary file 1 [file plants-15-02103-s001.zip › plants-4383327-supplementary/pepper_original_data/Goat_horn/104-6.jpg]

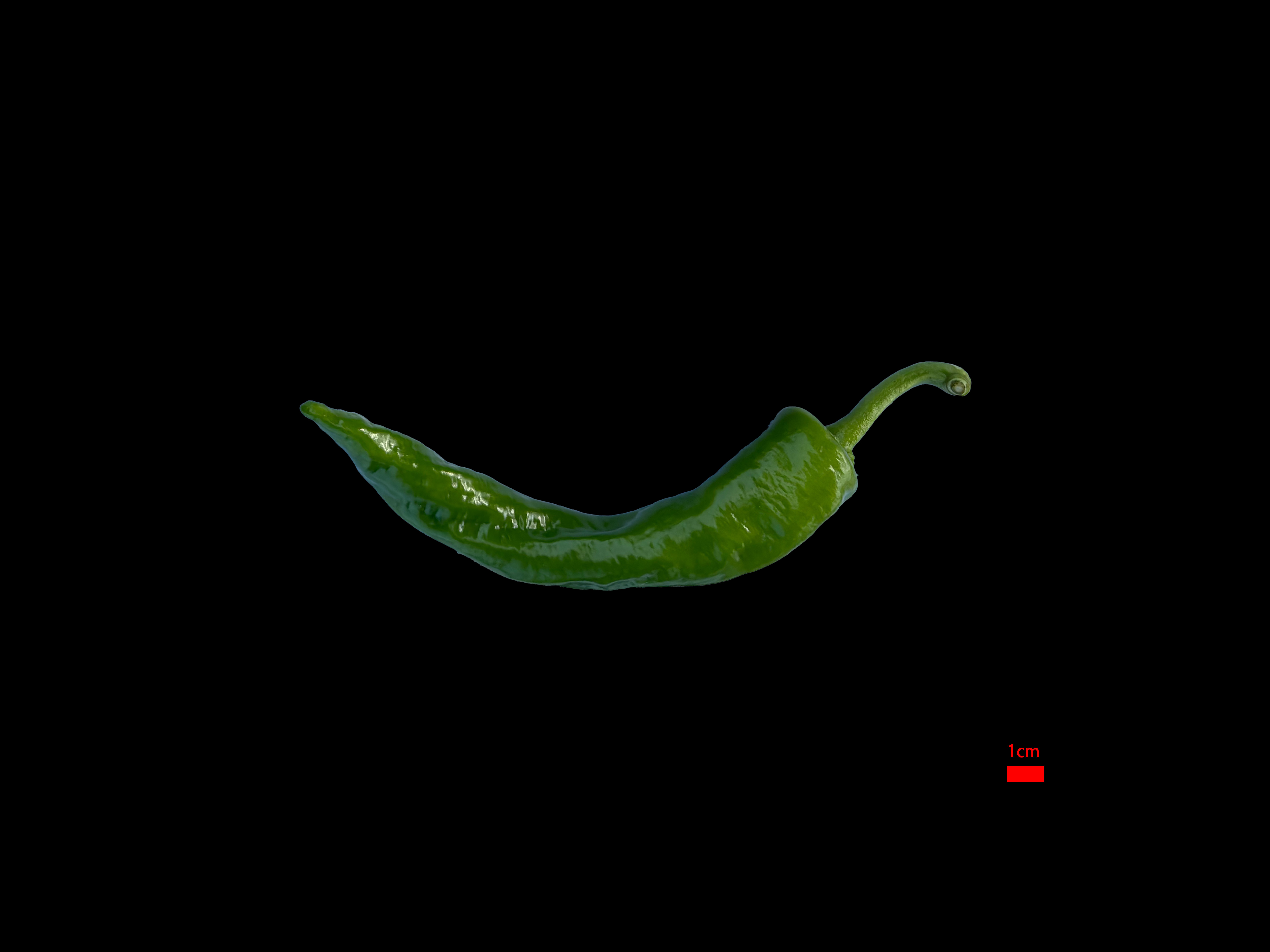

Supplement: Supplementary file 1 [file plants-15-02103-s001.zip › plants-4383327-supplementary/pepper_original_data/Goat_horn/104-7.jpg]

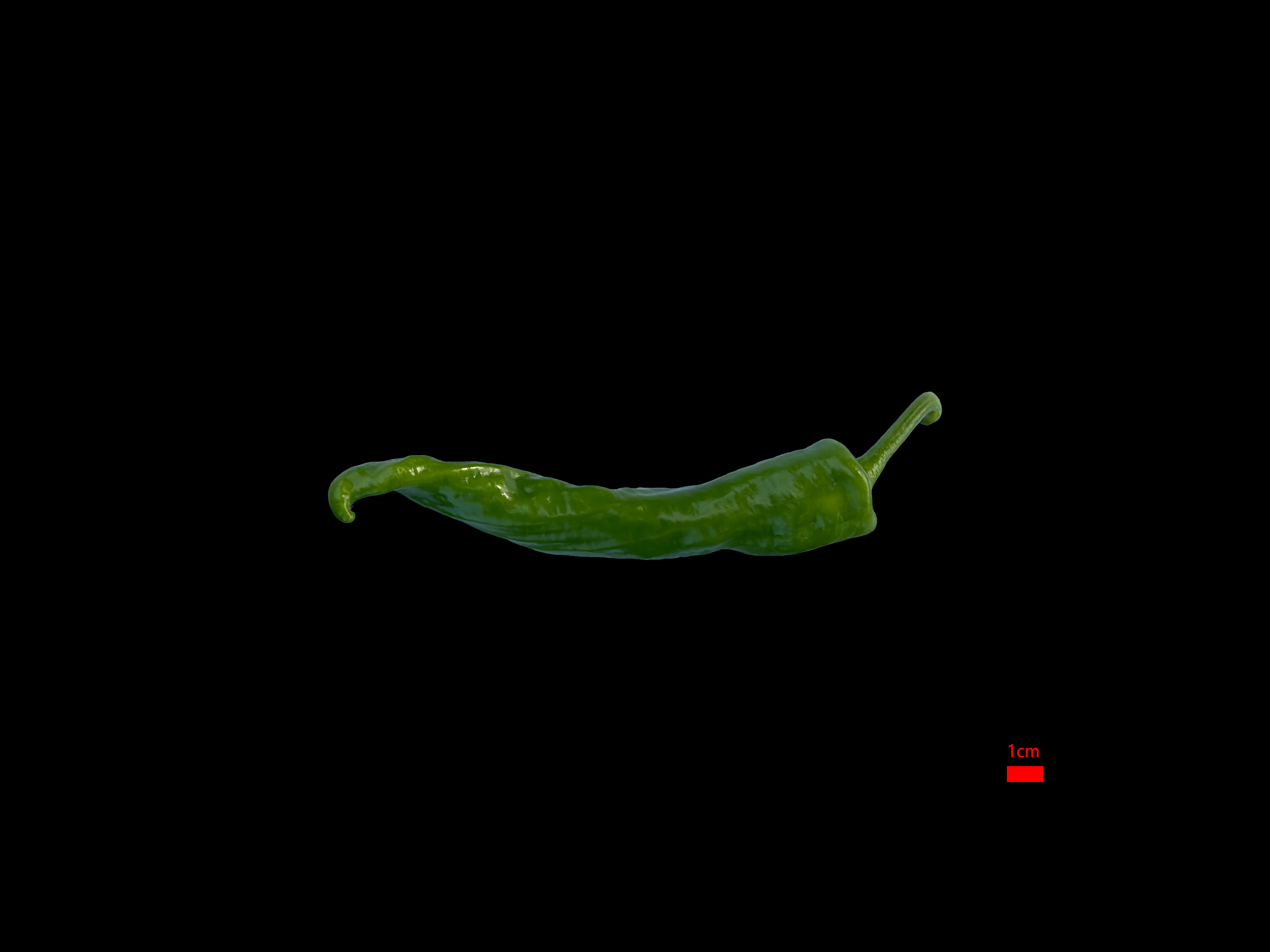

Supplement: Supplementary file 1 [file plants-15-02103-s001.zip › plants-4383327-supplementary/pepper_original_data/Goat_horn/104-8.jpg]

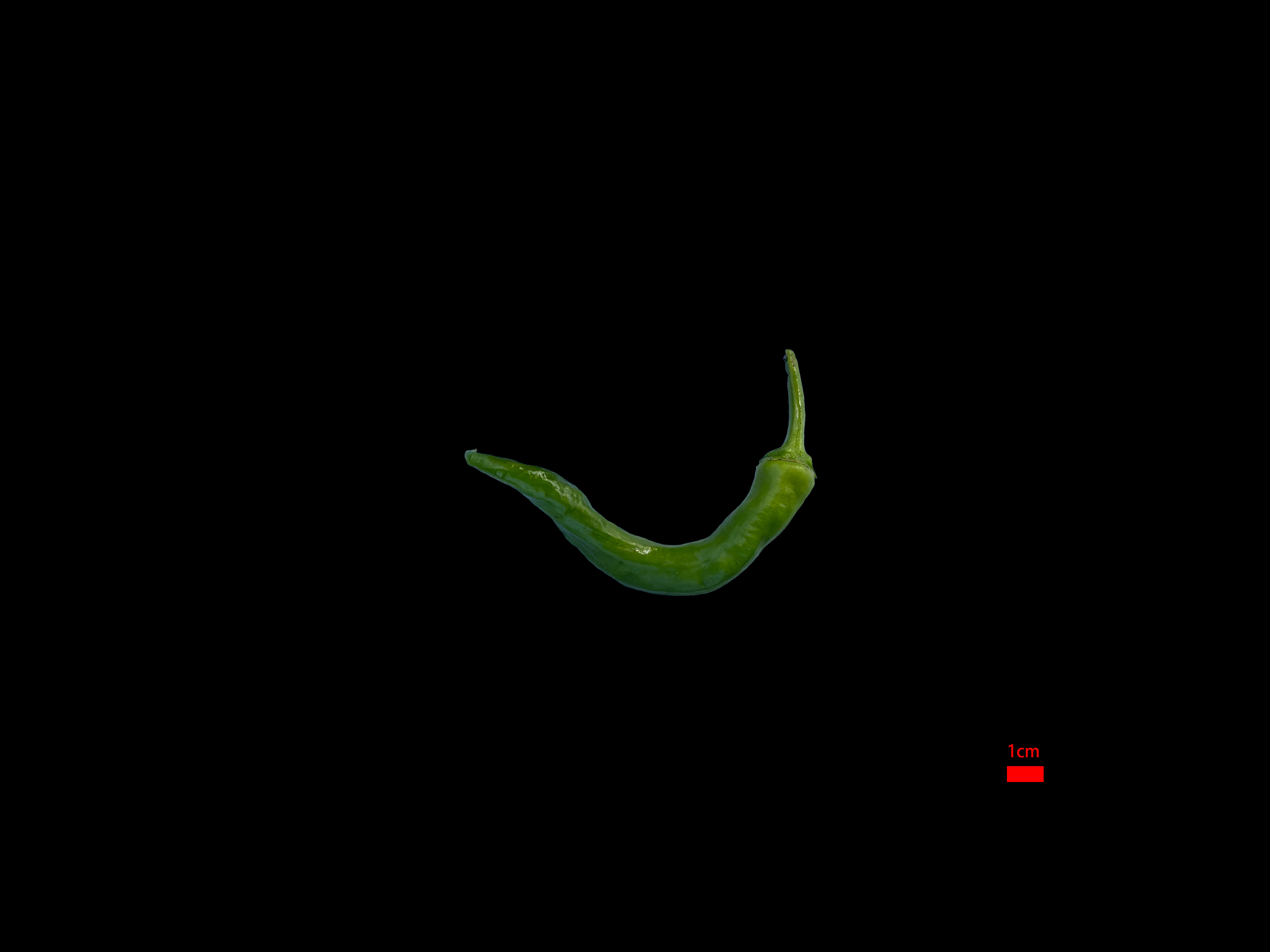

Supplement: Supplementary file 1 [file plants-15-02103-s001.zip › plants-4383327-supplementary/pepper_original_data/Goat_horn/104-9.jpg]

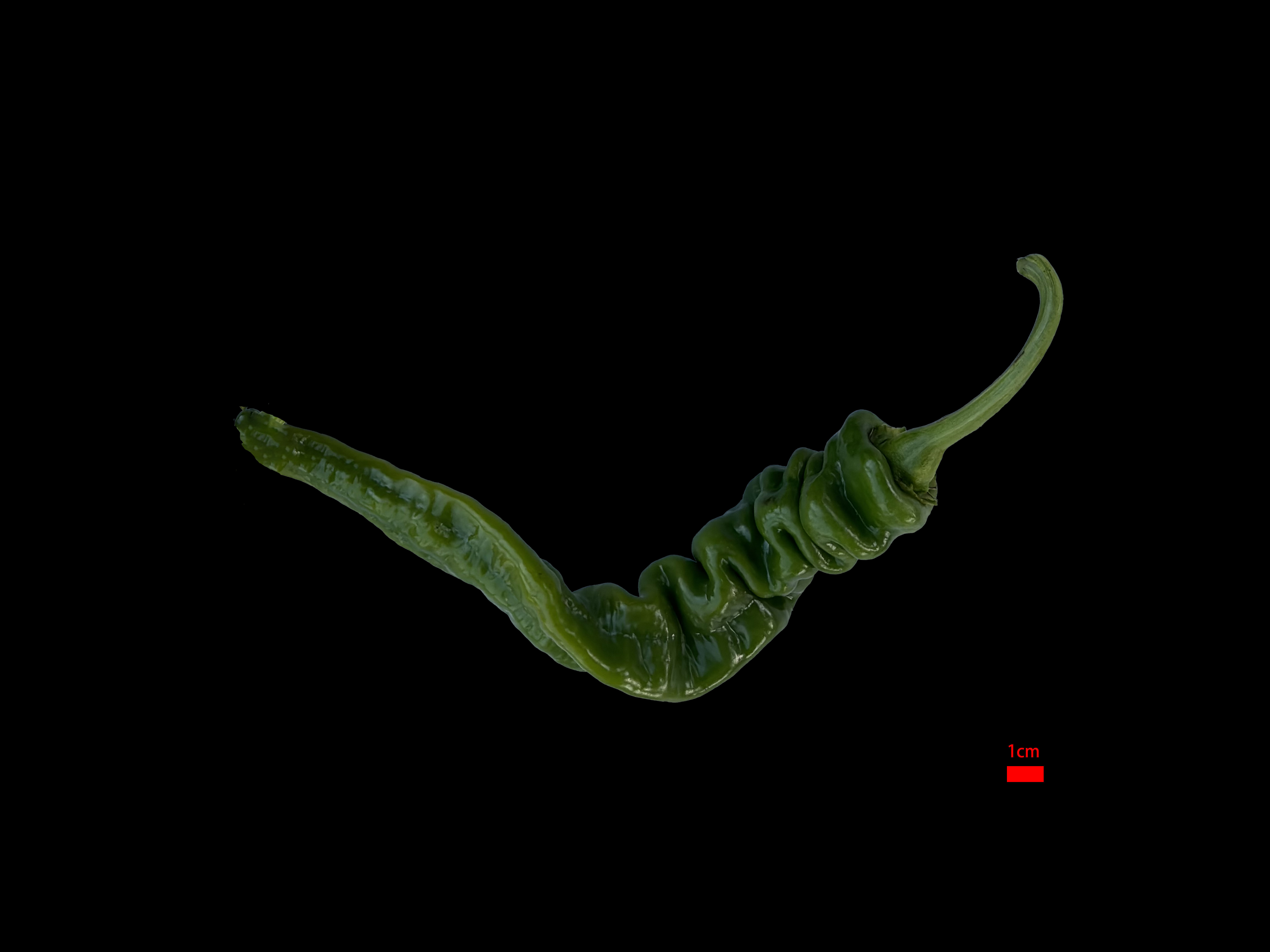

Supplement: Supplementary file 1 [file plants-15-02103-s001.zip › plants-4383327-supplementary/pepper_original_data/Goat_horn/126-1.jpg]

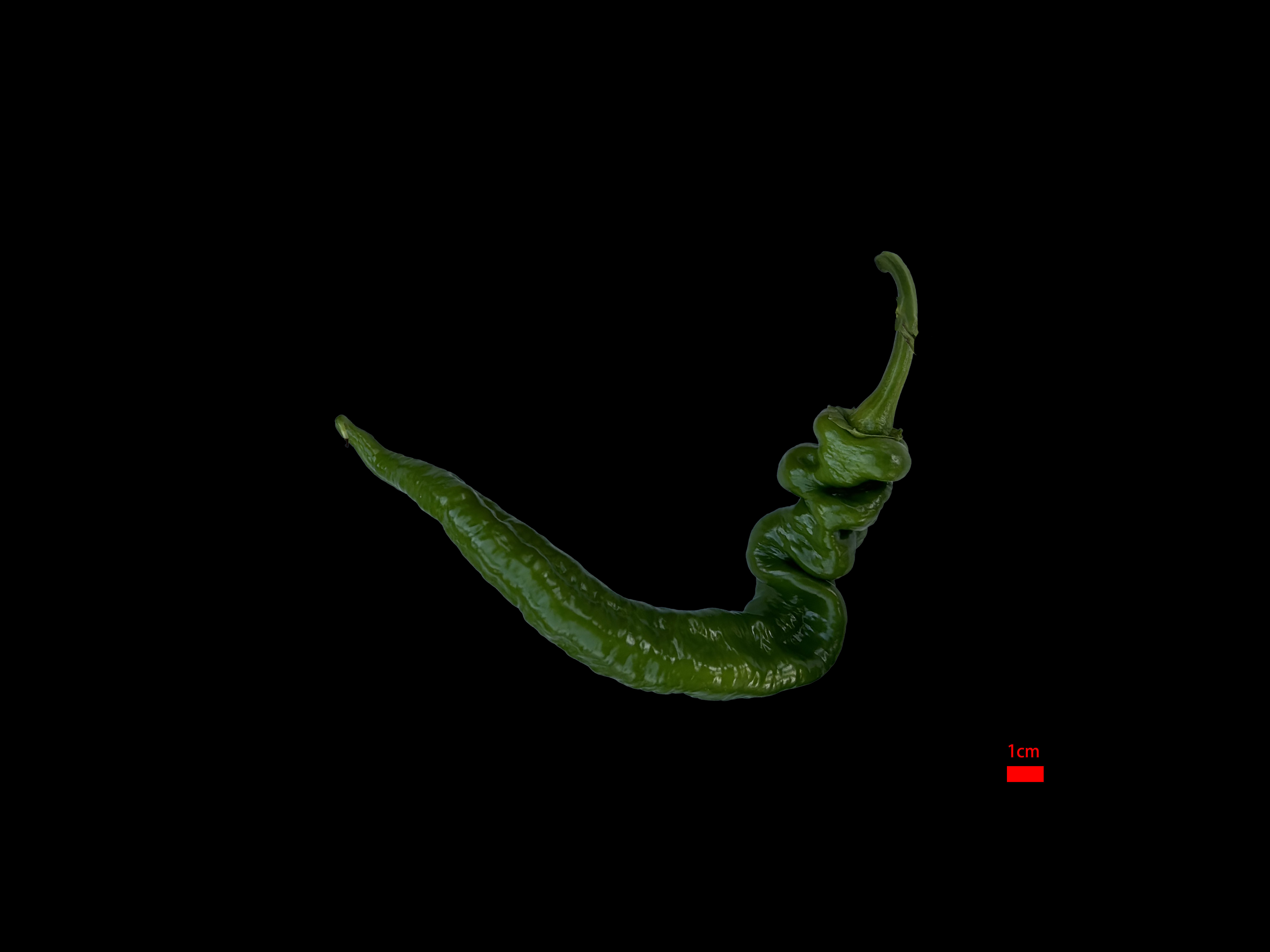

Supplement: Supplementary file 1 [file plants-15-02103-s001.zip › plants-4383327-supplementary/pepper_original_data/Goat_horn/126-2.jpg]

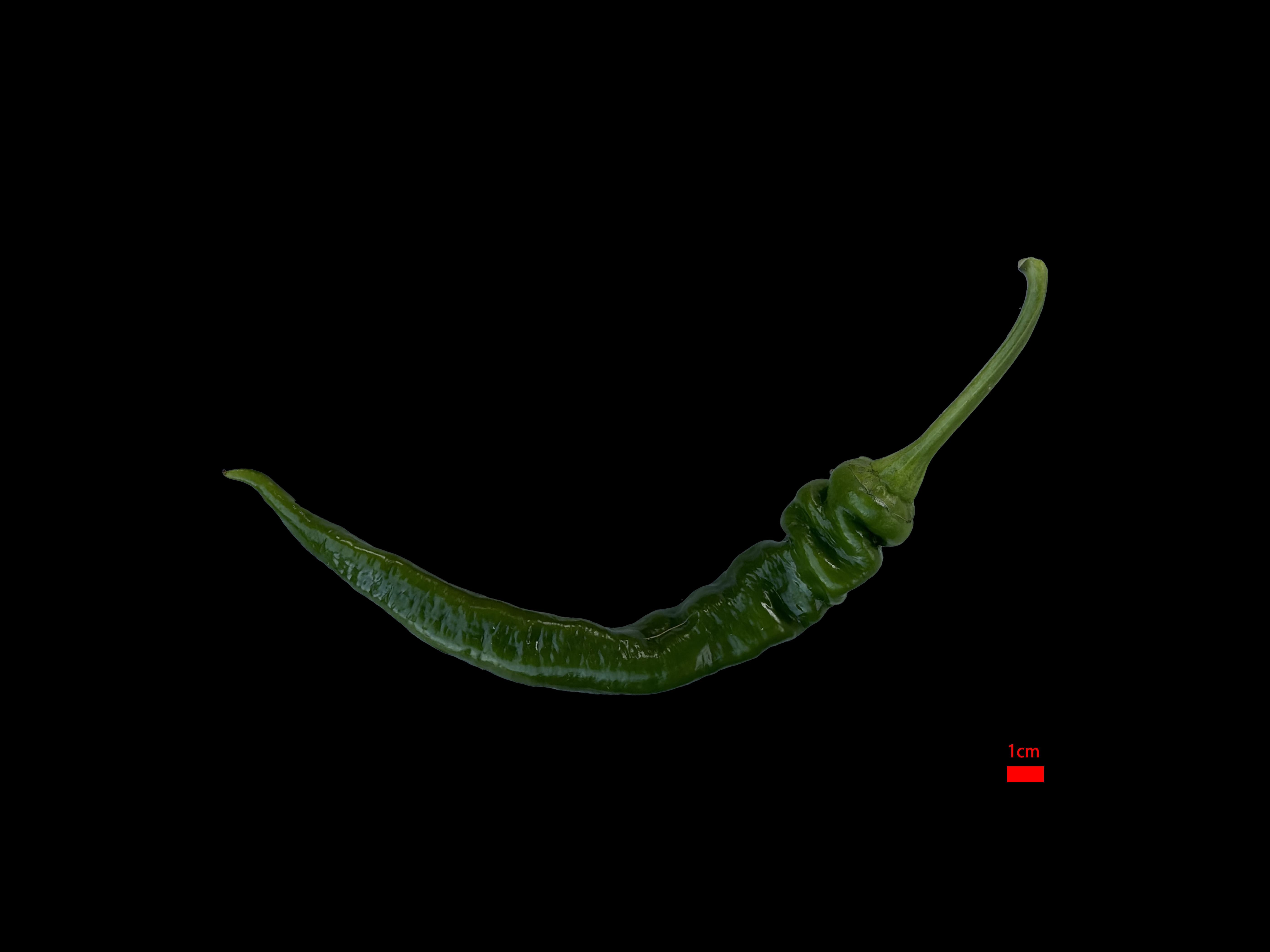

Supplement: Supplementary file 1 [file plants-15-02103-s001.zip › plants-4383327-supplementary/pepper_original_data/Goat_horn/126-4.jpg]

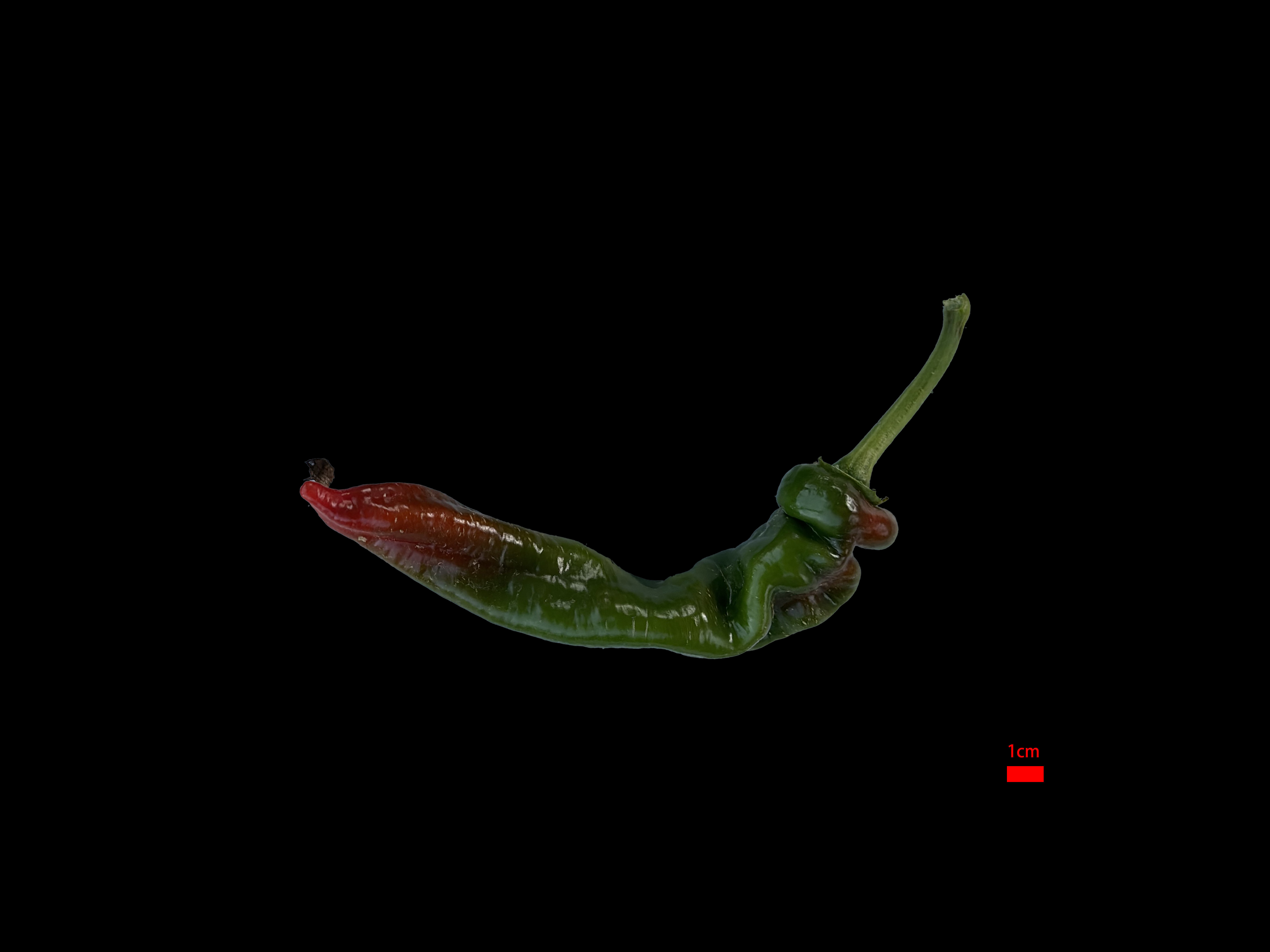

Supplement: Supplementary file 1 [file plants-15-02103-s001.zip › plants-4383327-supplementary/pepper_original_data/Goat_horn/126-5.jpg]

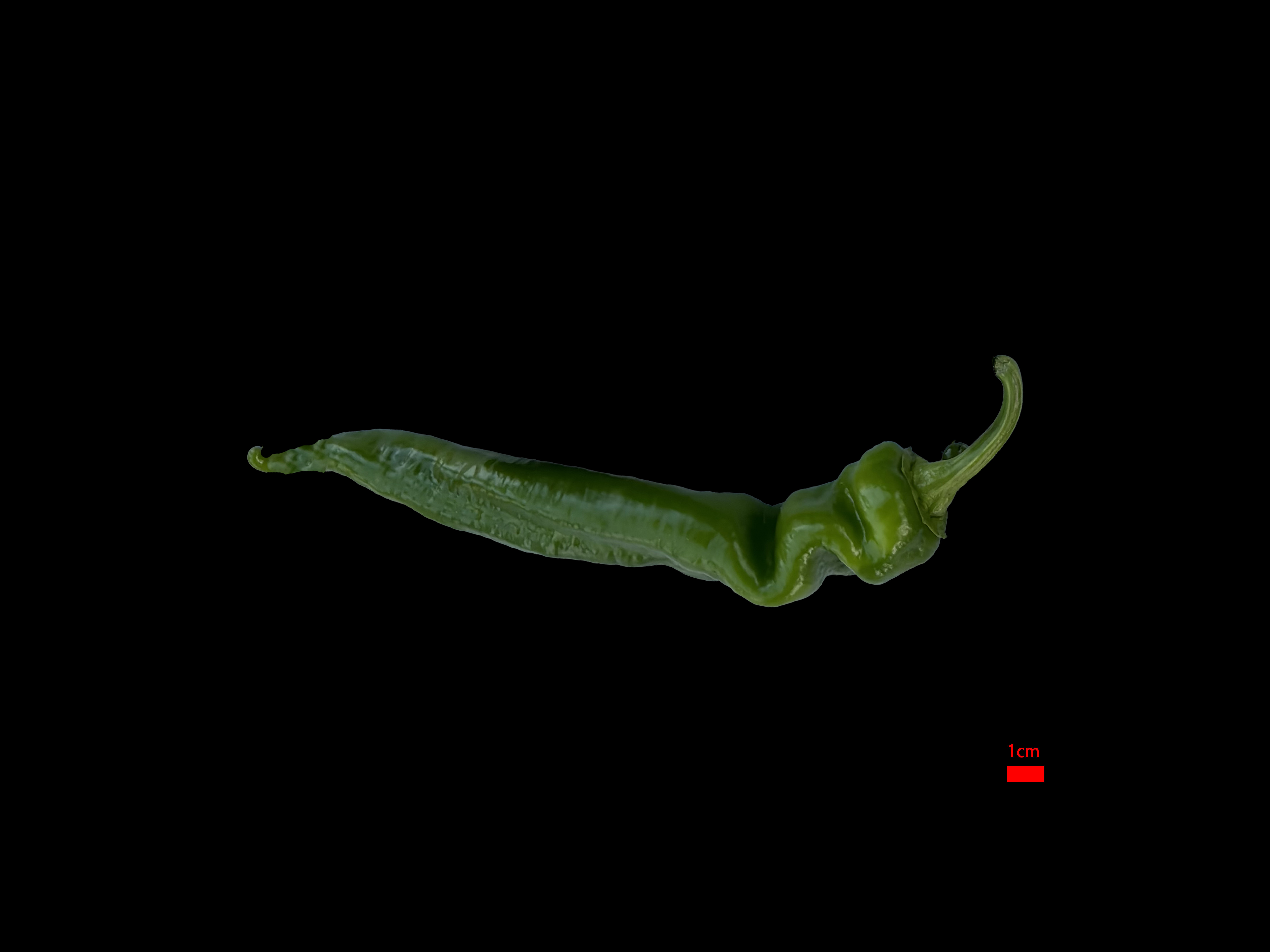

Supplement: Supplementary file 1 [file plants-15-02103-s001.zip › plants-4383327-supplementary/pepper_original_data/Goat_horn/126-6.jpg]

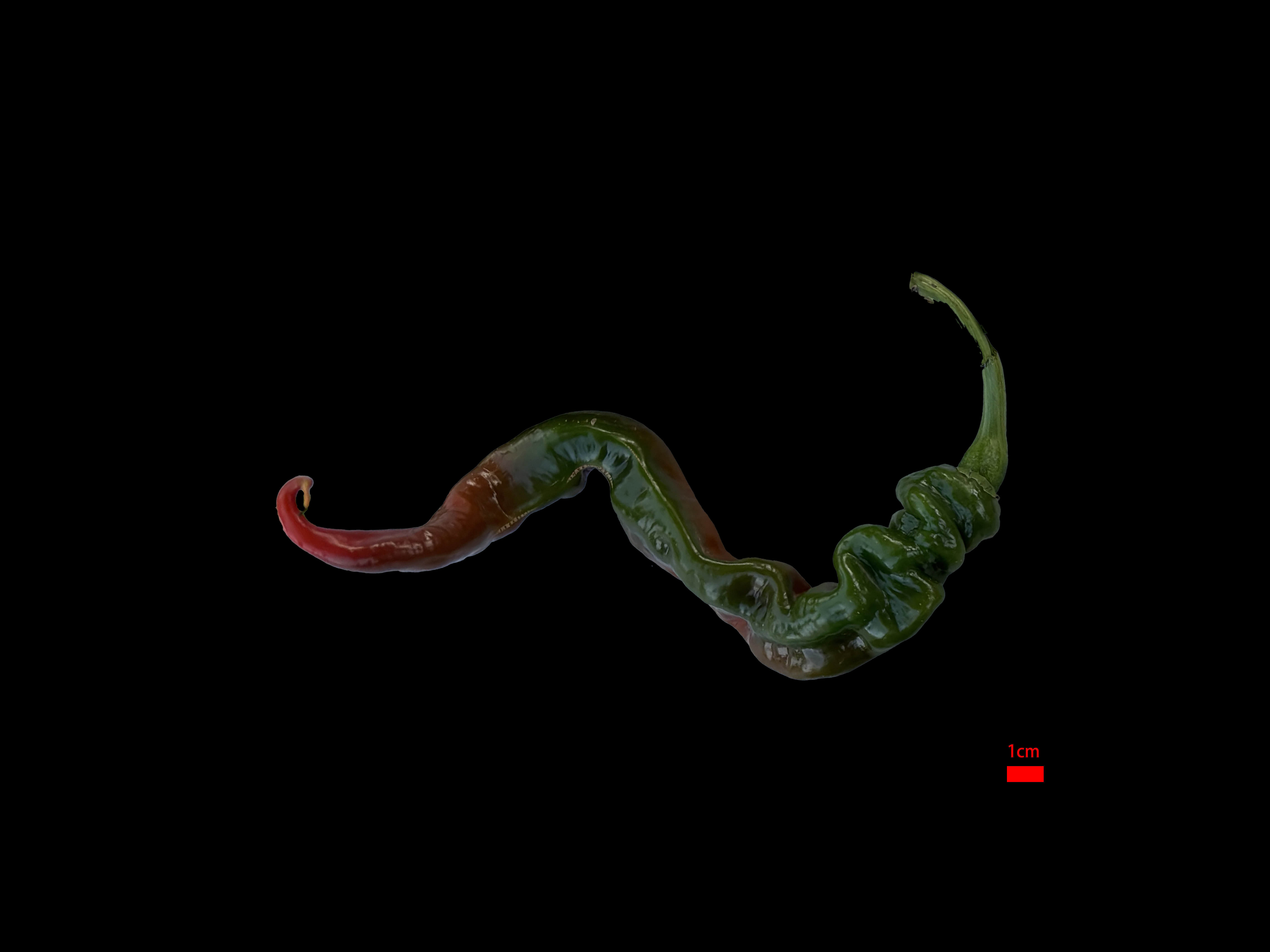

Supplement: Supplementary file 1 [file plants-15-02103-s001.zip › plants-4383327-supplementary/pepper_original_data/Goat_horn/126-7.jpg]

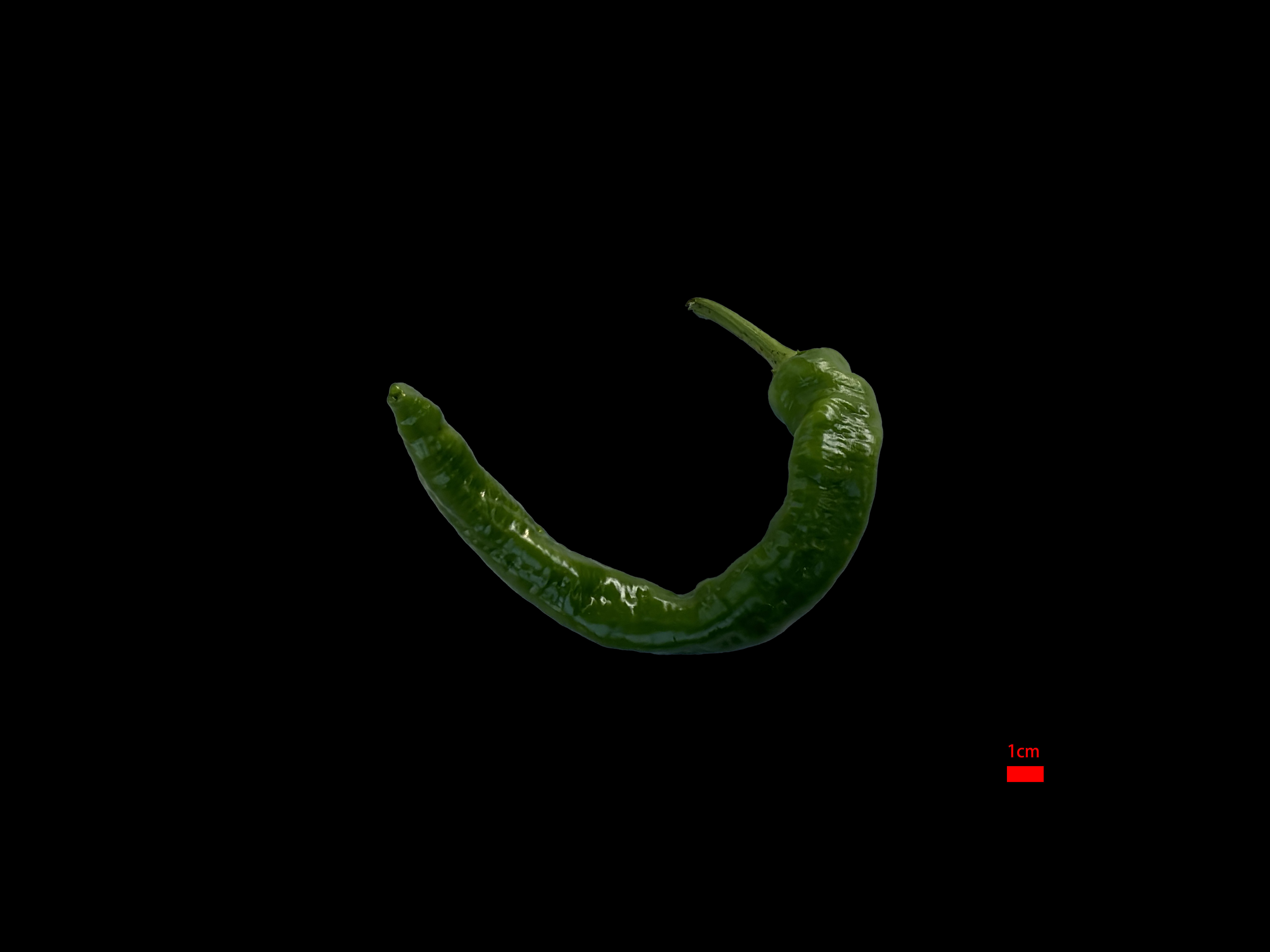

Supplement: Supplementary file 1 [file plants-15-02103-s001.zip › plants-4383327-supplementary/pepper_original_data/Goat_horn/130-1.jpg]

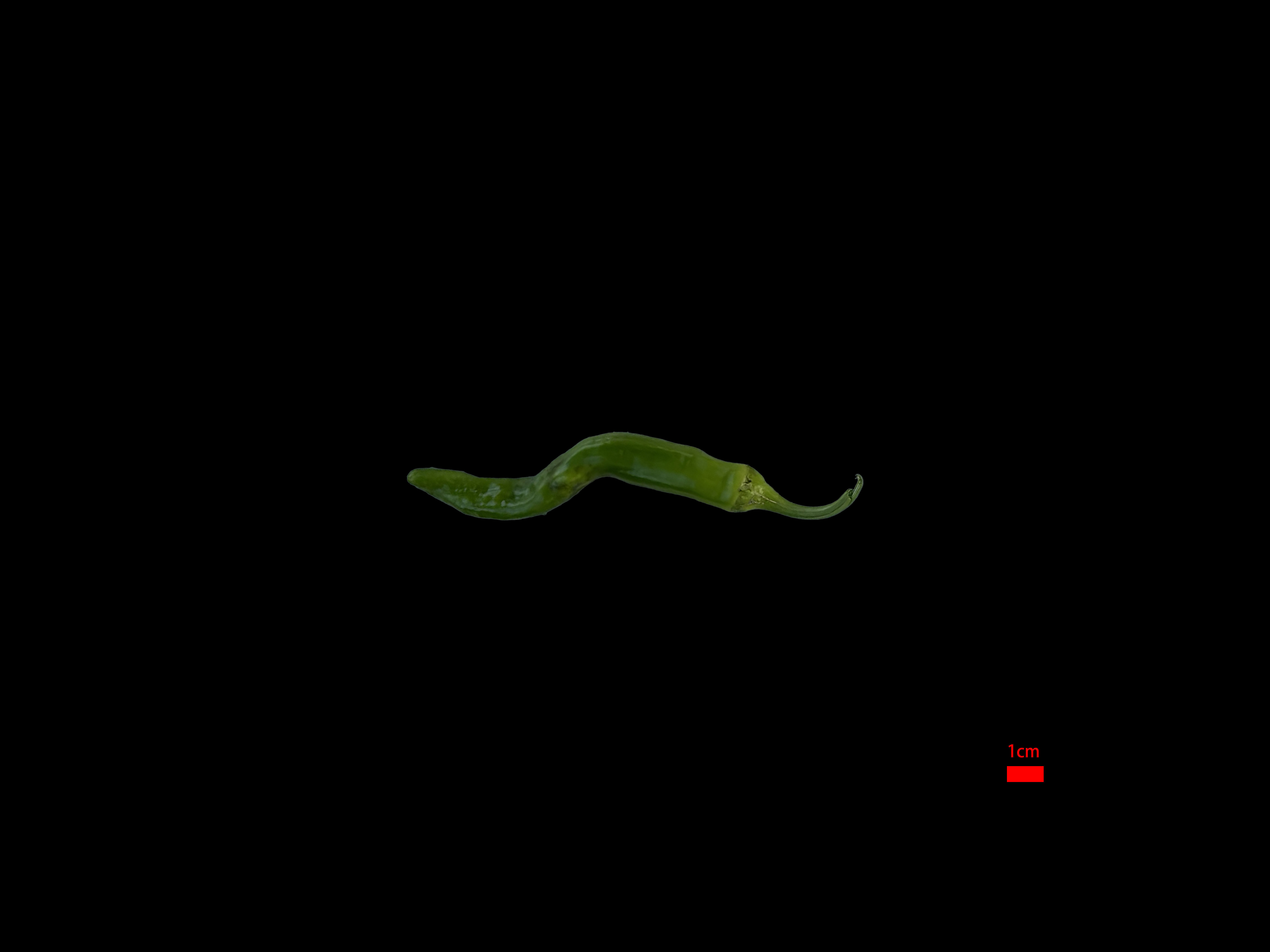

Supplement: Supplementary file 1 [file plants-15-02103-s001.zip › plants-4383327-supplementary/pepper_original_data/Goat_horn/130-10.jpg]

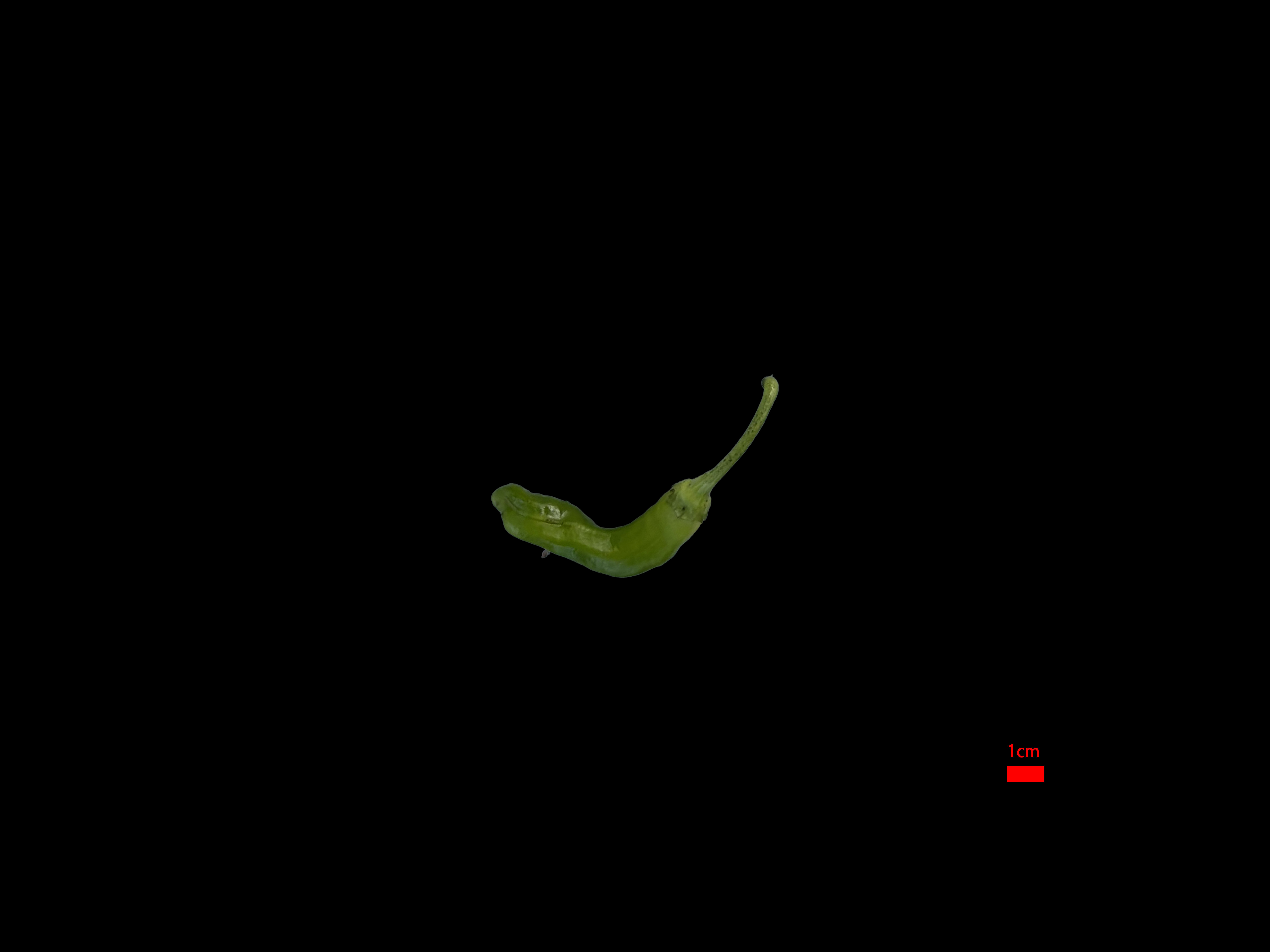

Supplement: Supplementary file 1 [file plants-15-02103-s001.zip › plants-4383327-supplementary/pepper_original_data/Goat_horn/130-11.jpg]

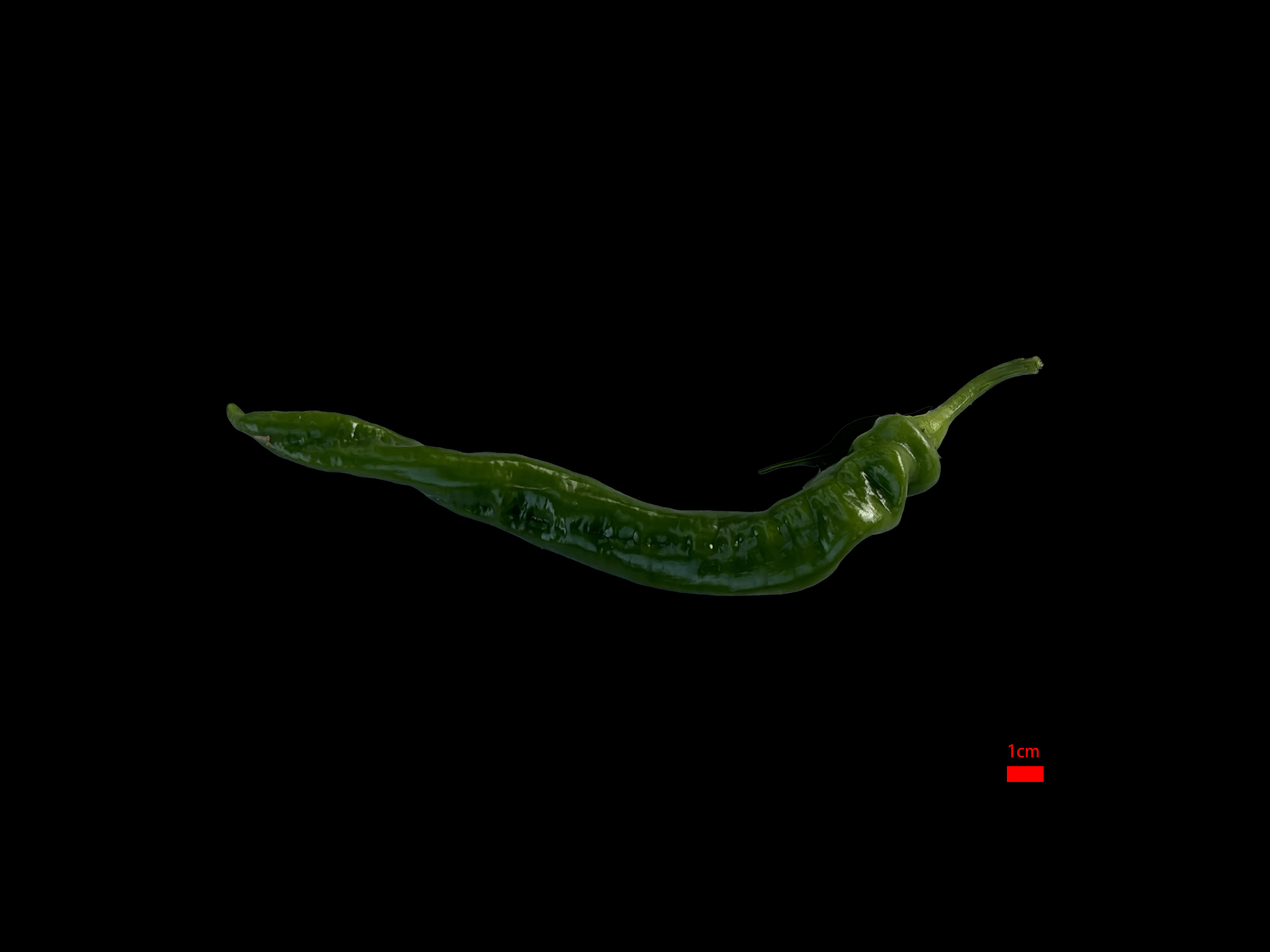

Supplement: Supplementary file 1 [file plants-15-02103-s001.zip › plants-4383327-supplementary/pepper_original_data/Goat_horn/130-2.jpg]

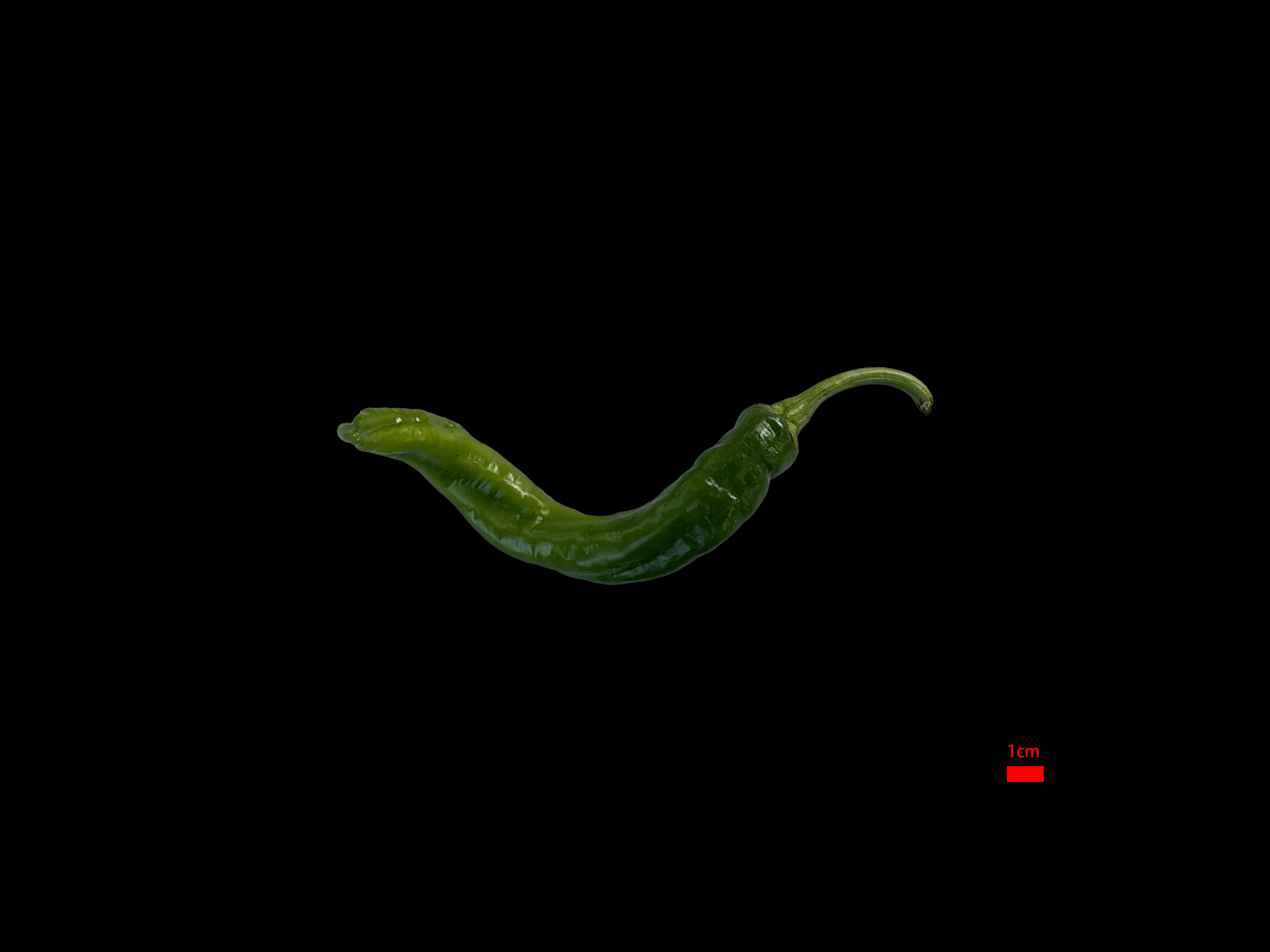

Supplement: Supplementary file 1 [file plants-15-02103-s001.zip › plants-4383327-supplementary/pepper_original_data/Goat_horn/130-3.jpg]

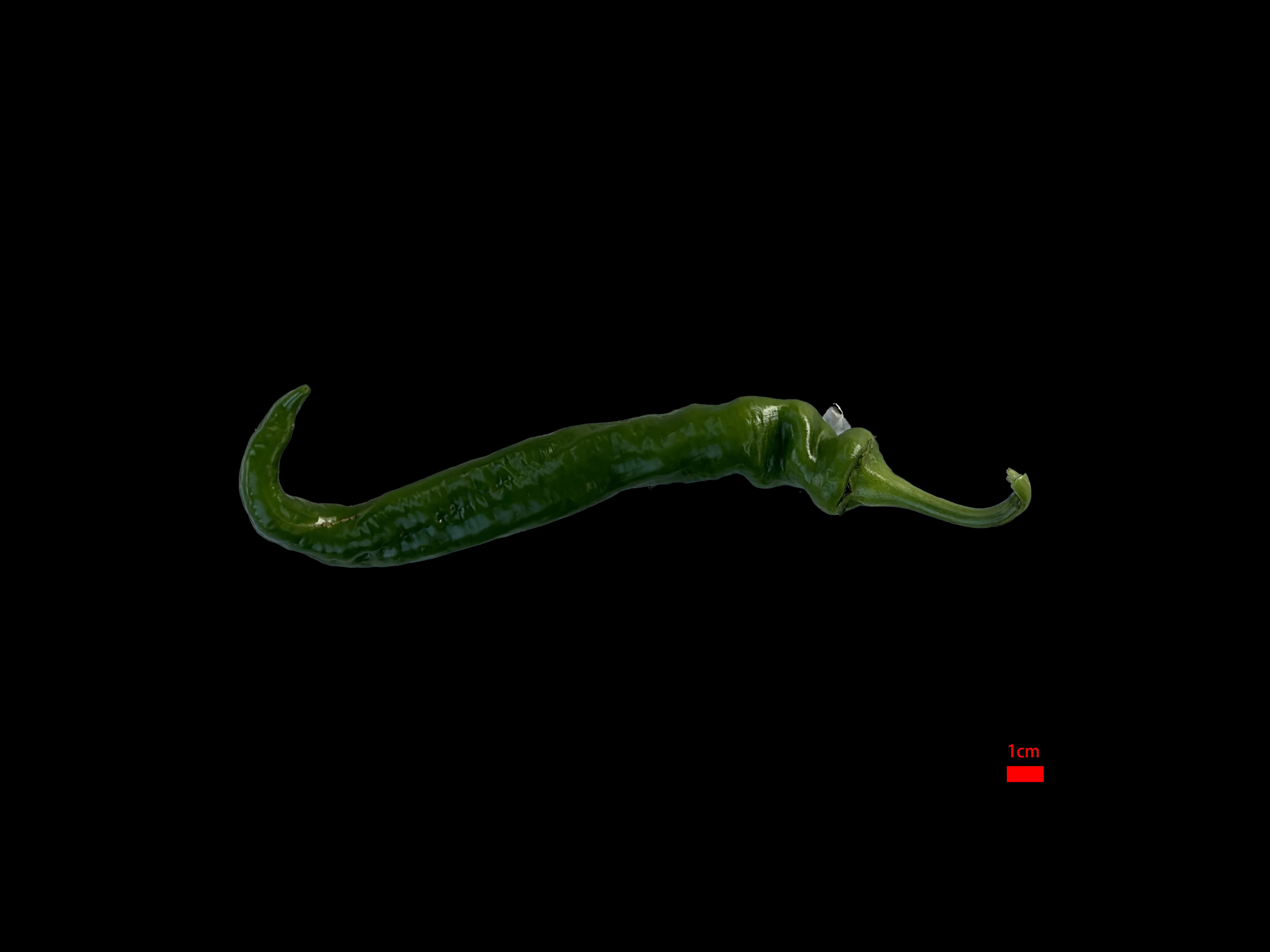

Supplement: Supplementary file 1 [file plants-15-02103-s001.zip › plants-4383327-supplementary/pepper_original_data/Goat_horn/130-4.jpg]

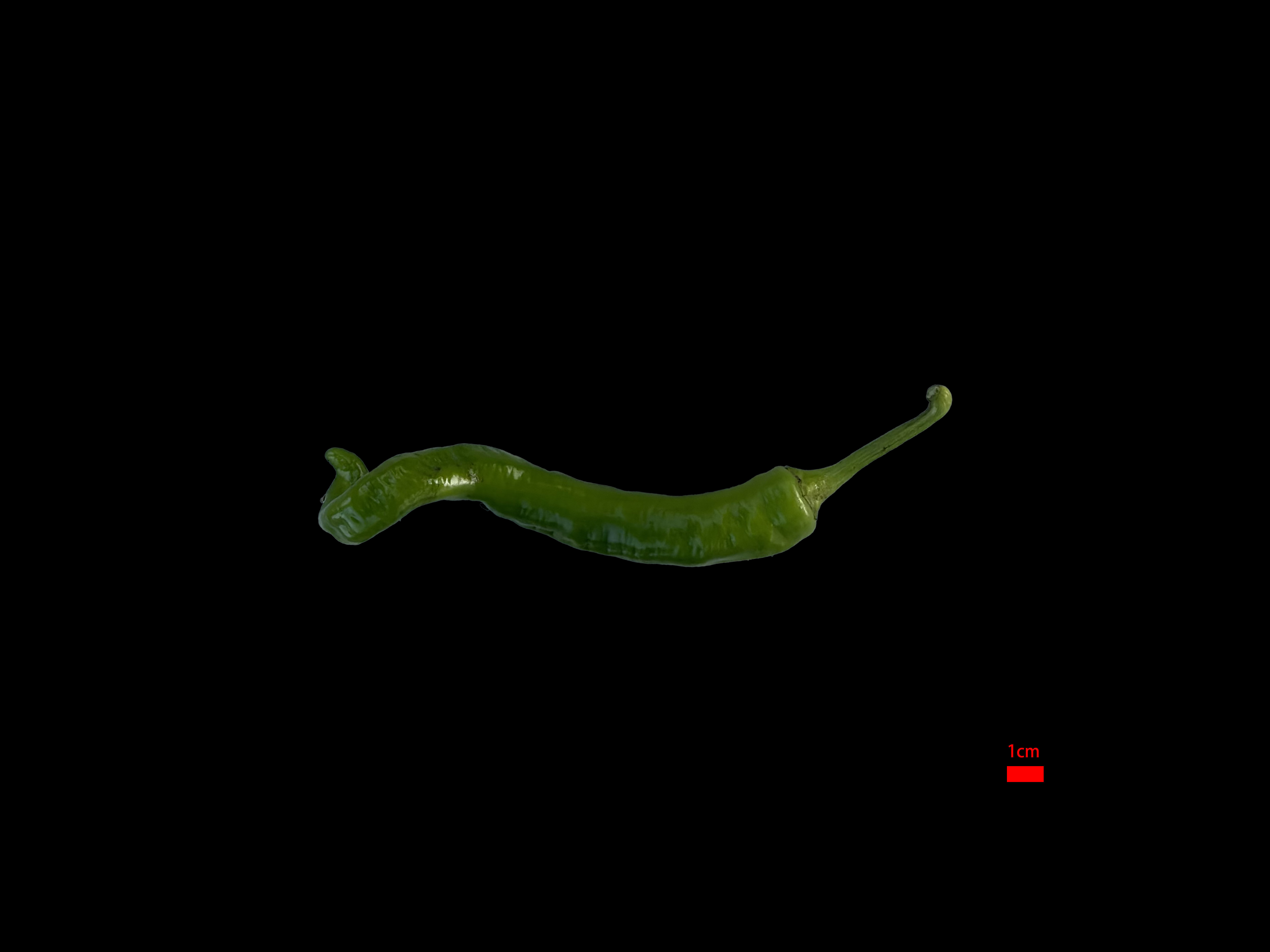

Supplement: Supplementary file 1 [file plants-15-02103-s001.zip › plants-4383327-supplementary/pepper_original_data/Goat_horn/130-5.jpg]

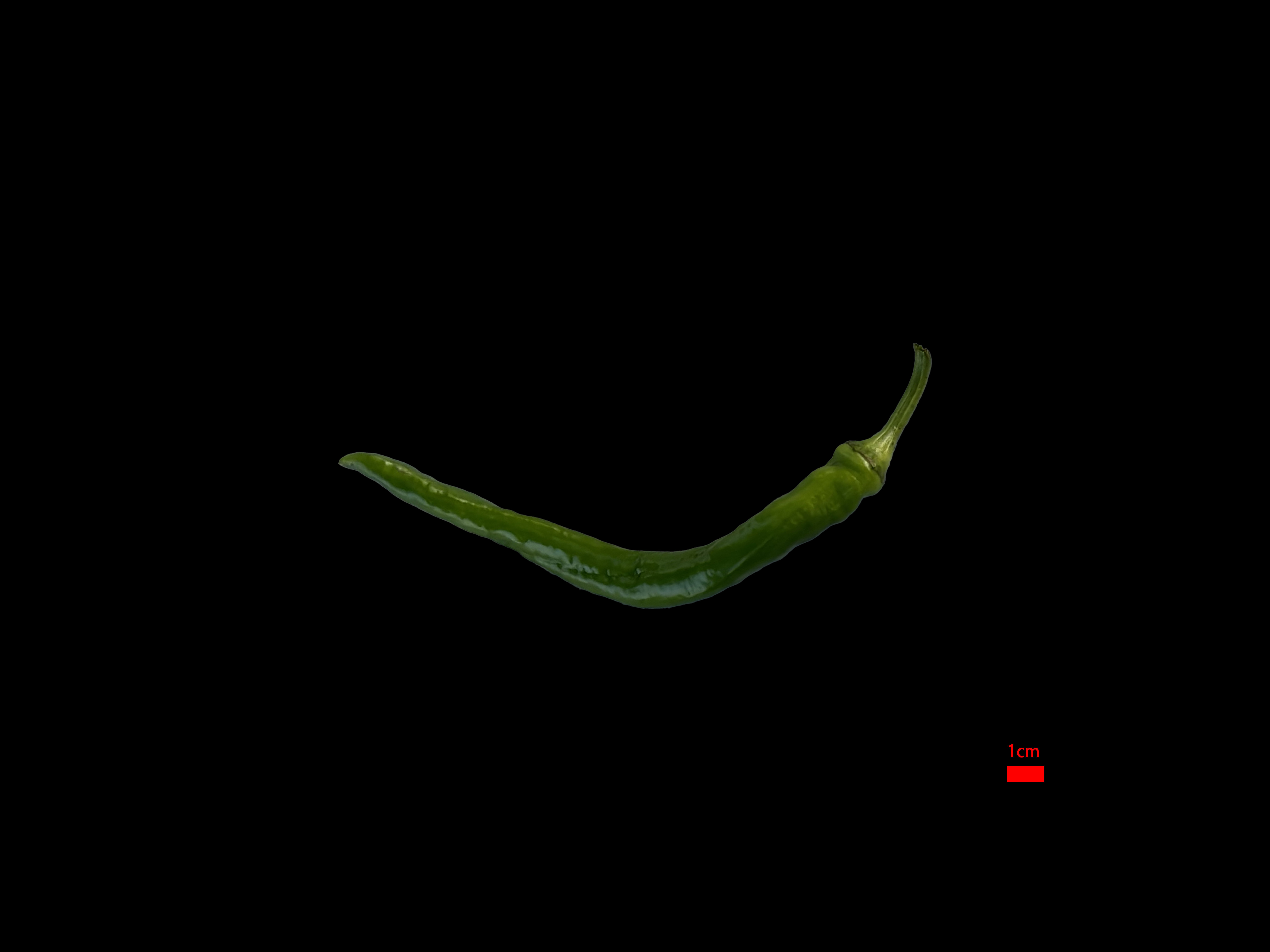

Supplement: Supplementary file 1 [file plants-15-02103-s001.zip › plants-4383327-supplementary/pepper_original_data/Goat_horn/130-6.jpg]

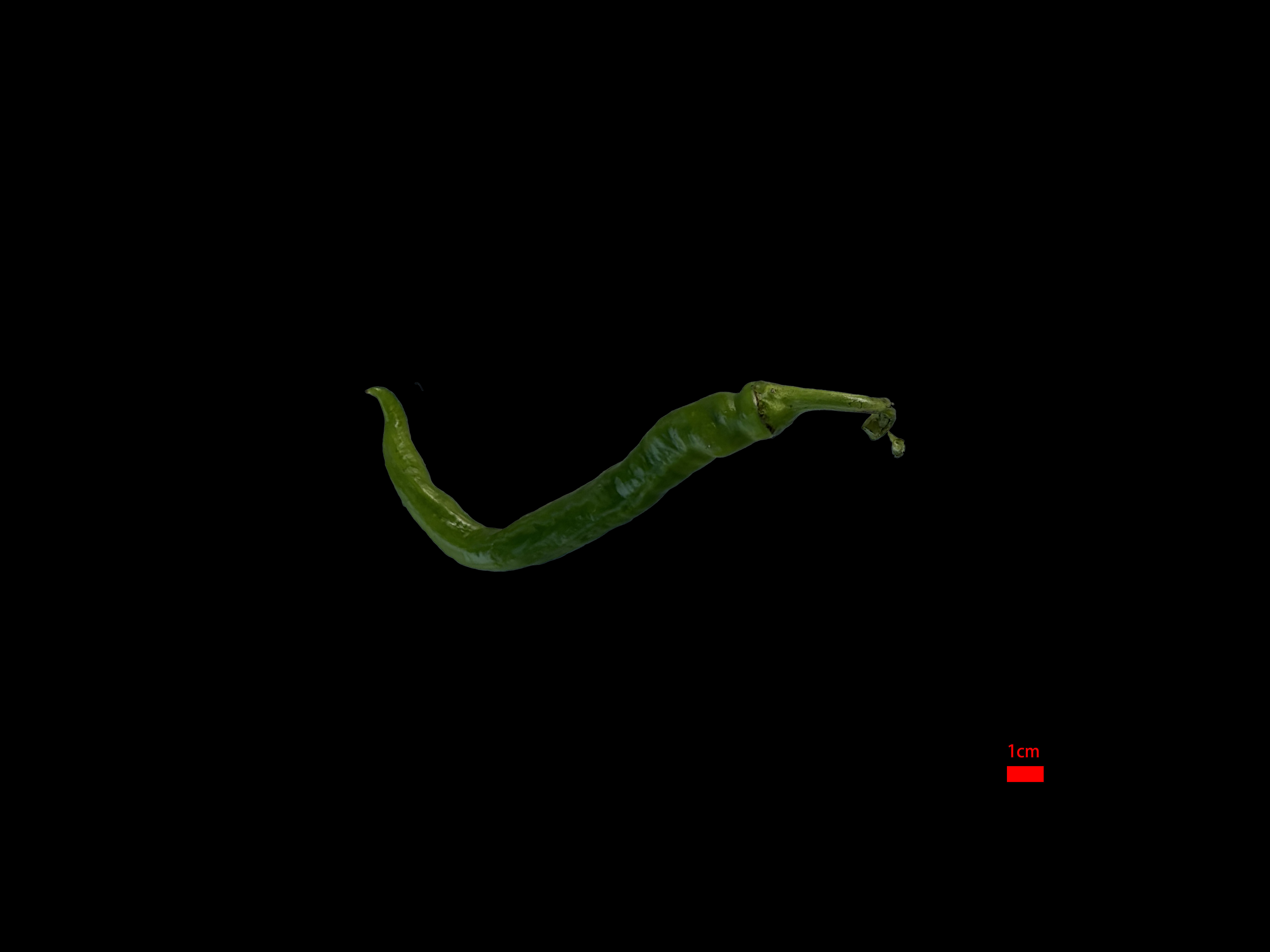

Supplement: Supplementary file 1 [file plants-15-02103-s001.zip › plants-4383327-supplementary/pepper_original_data/Goat_horn/130-7.jpg]

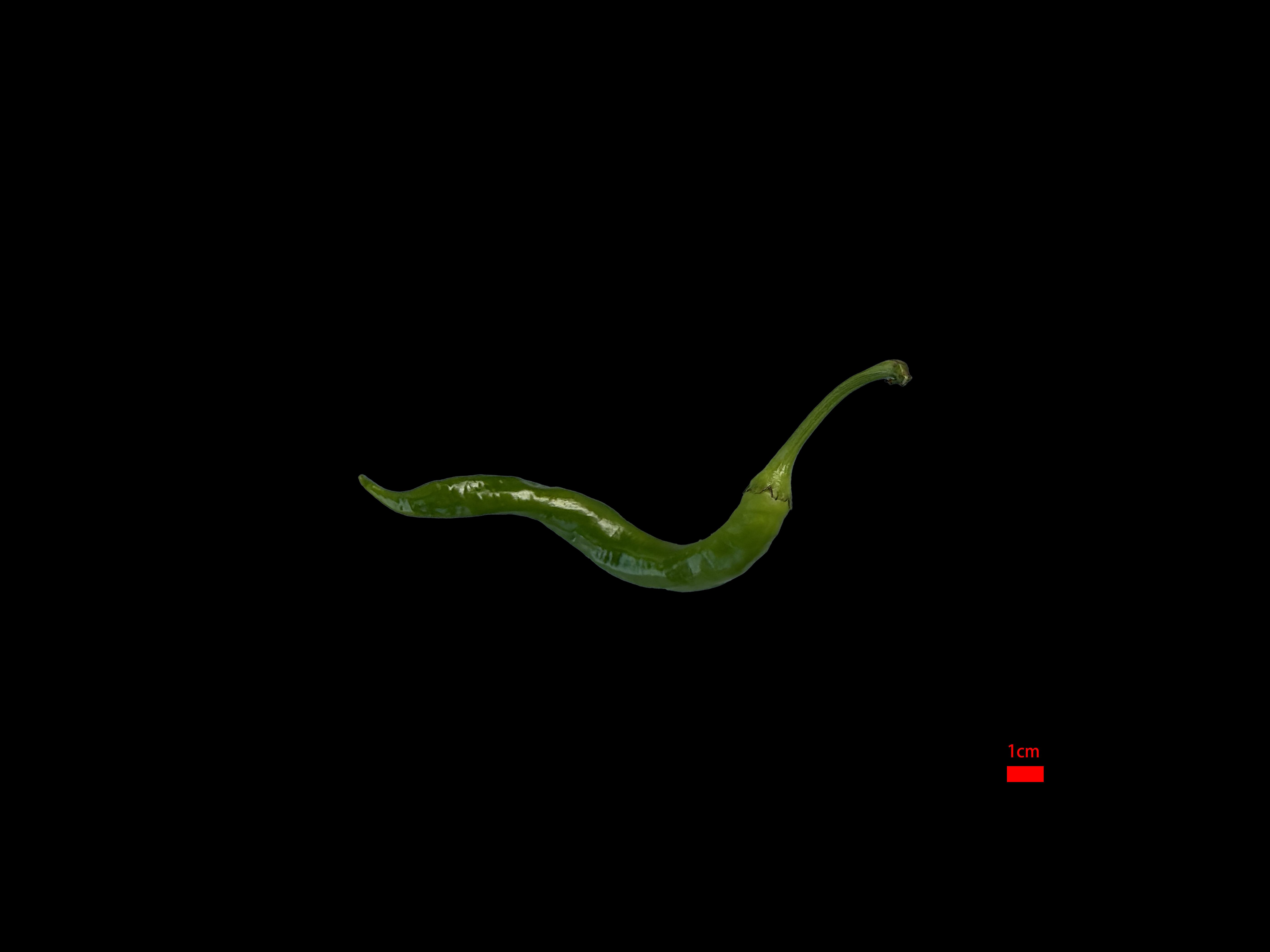

Supplement: Supplementary file 1 [file plants-15-02103-s001.zip › plants-4383327-supplementary/pepper_original_data/Goat_horn/130-9.jpg]

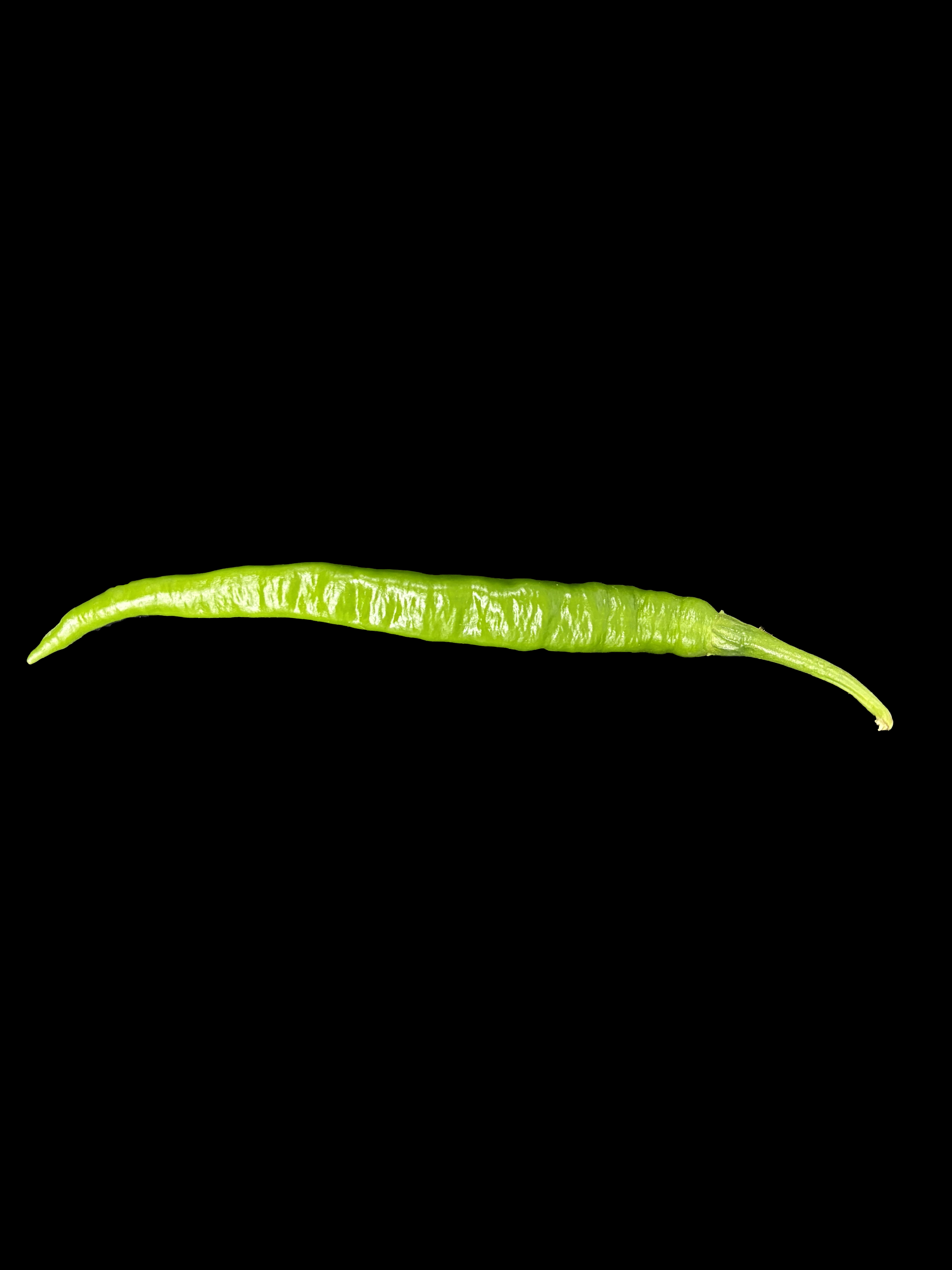

Supplement: Supplementary file 1 [file plants-15-02103-s001.zip › plants-4383327-supplementary/pepper_original_data/Goat_horn/136.1.jpg]

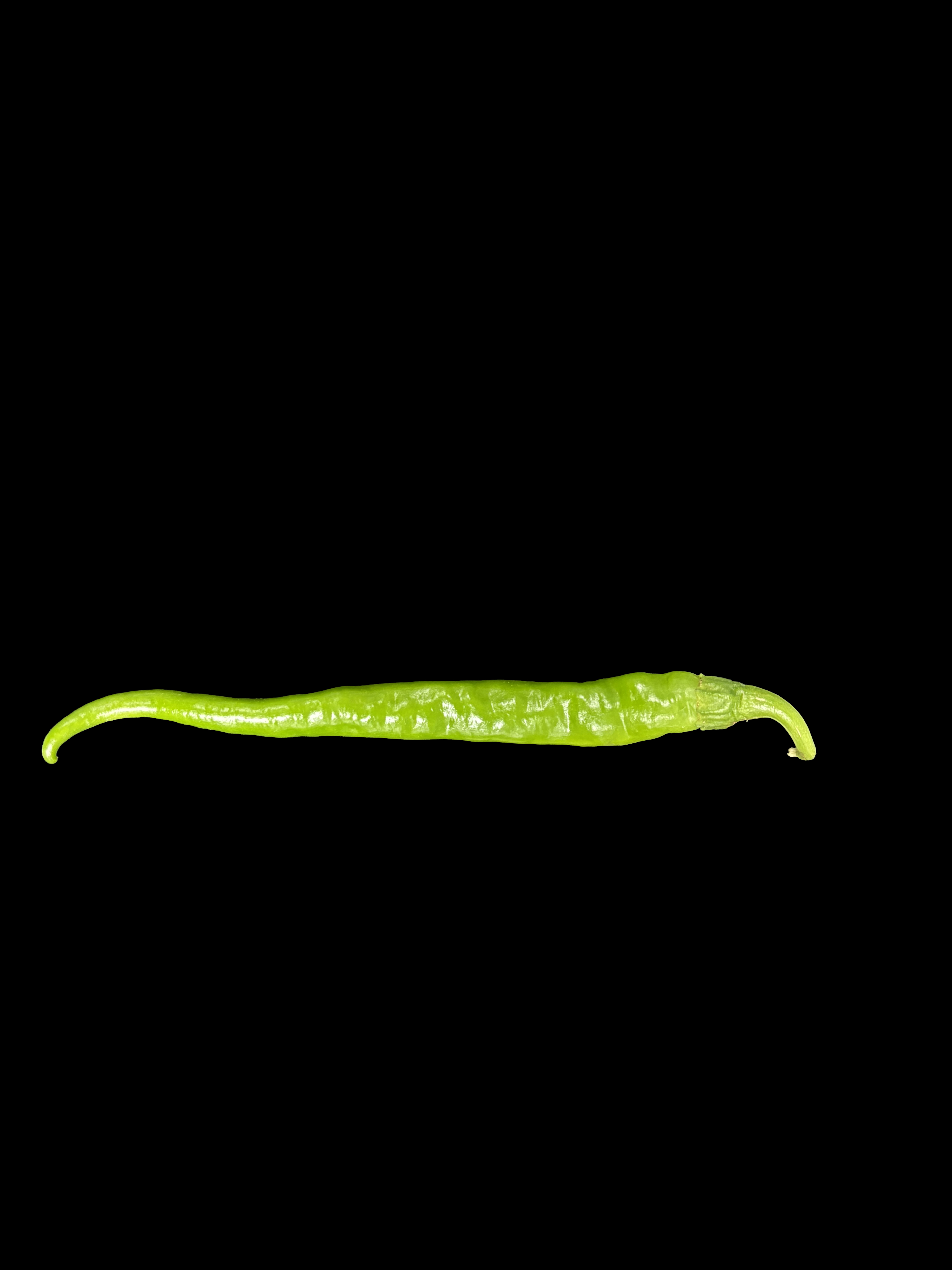

Supplement: Supplementary file 1 [file plants-15-02103-s001.zip › plants-4383327-supplementary/pepper_original_data/Goat_horn/136.2.jpg]

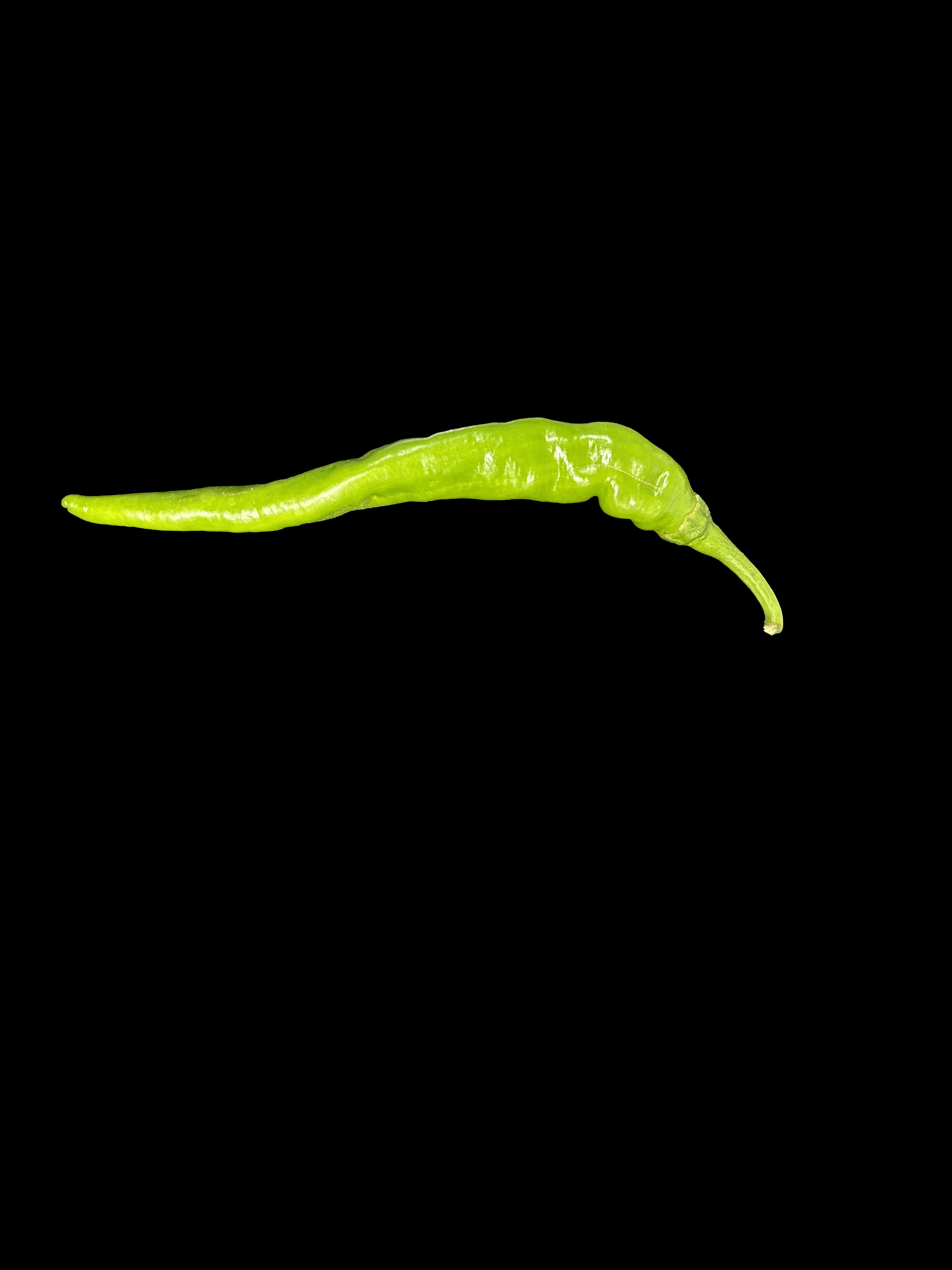

Supplement: Supplementary file 1 [file plants-15-02103-s001.zip › plants-4383327-supplementary/pepper_original_data/Goat_horn/136.jpg]

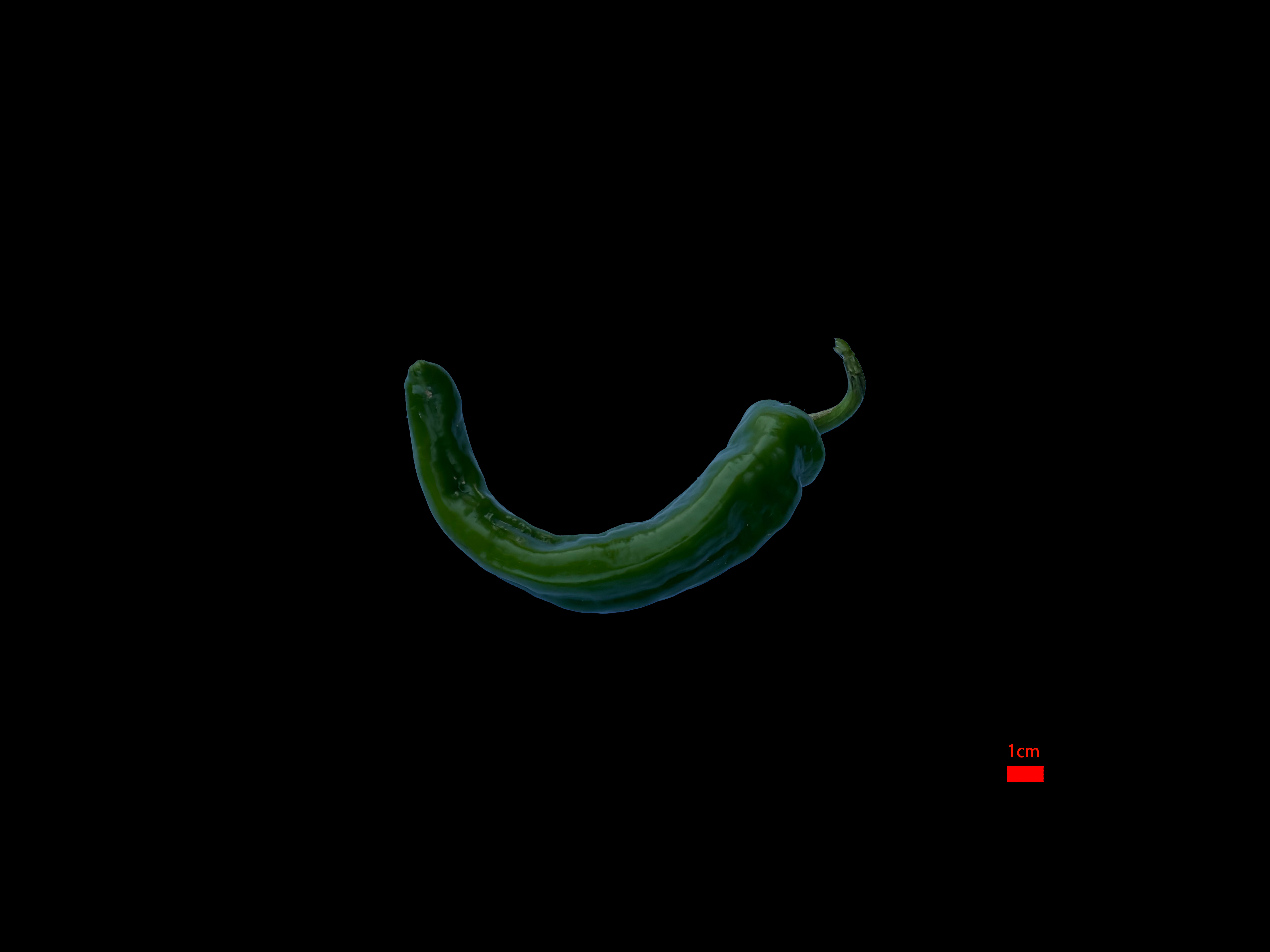

Supplement: Supplementary file 1 [file plants-15-02103-s001.zip › plants-4383327-supplementary/pepper_original_data/Goat_horn/139-1.jpg]

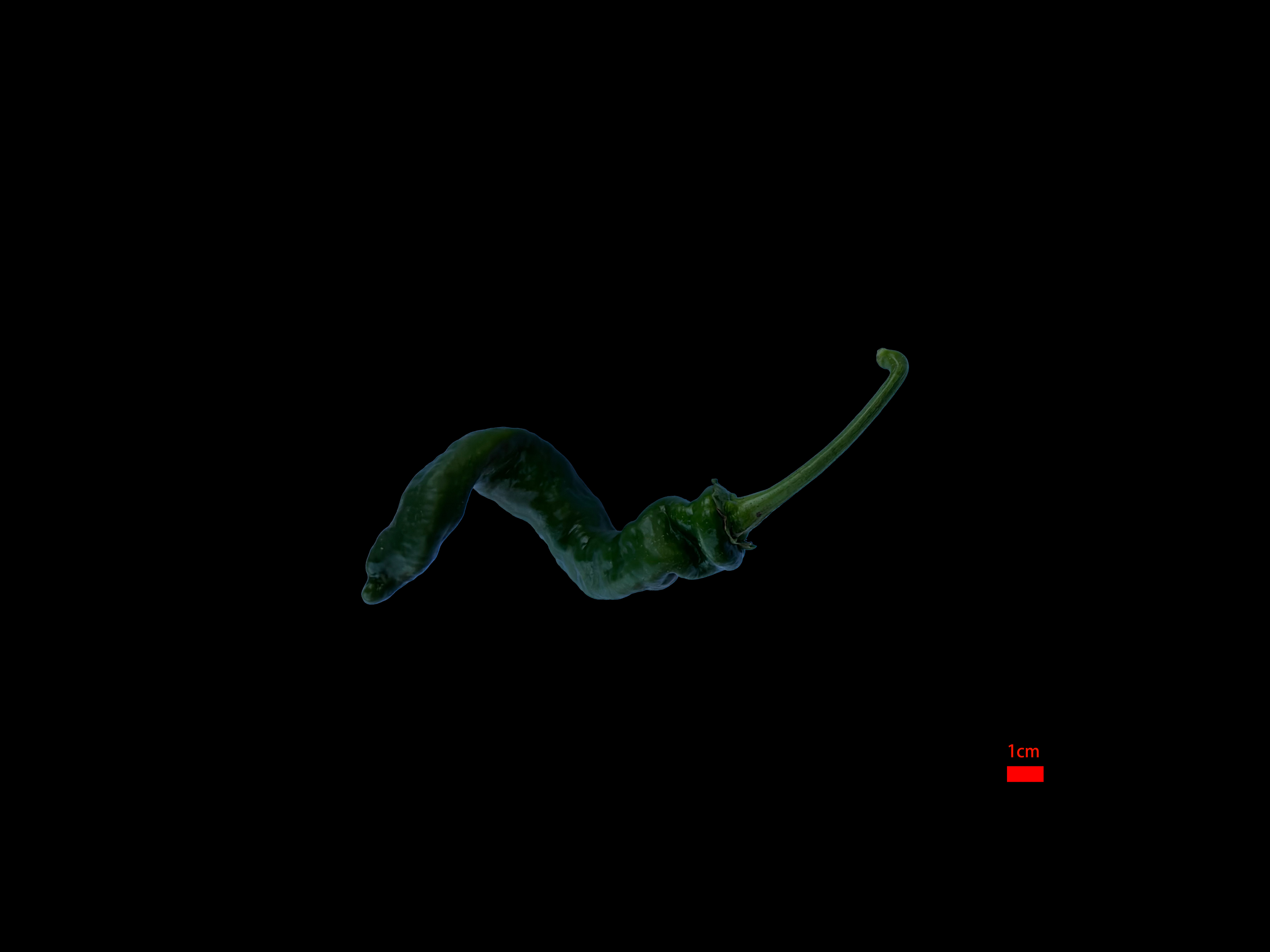

Supplement: Supplementary file 1 [file plants-15-02103-s001.zip › plants-4383327-supplementary/pepper_original_data/Goat_horn/139-11.jpg]

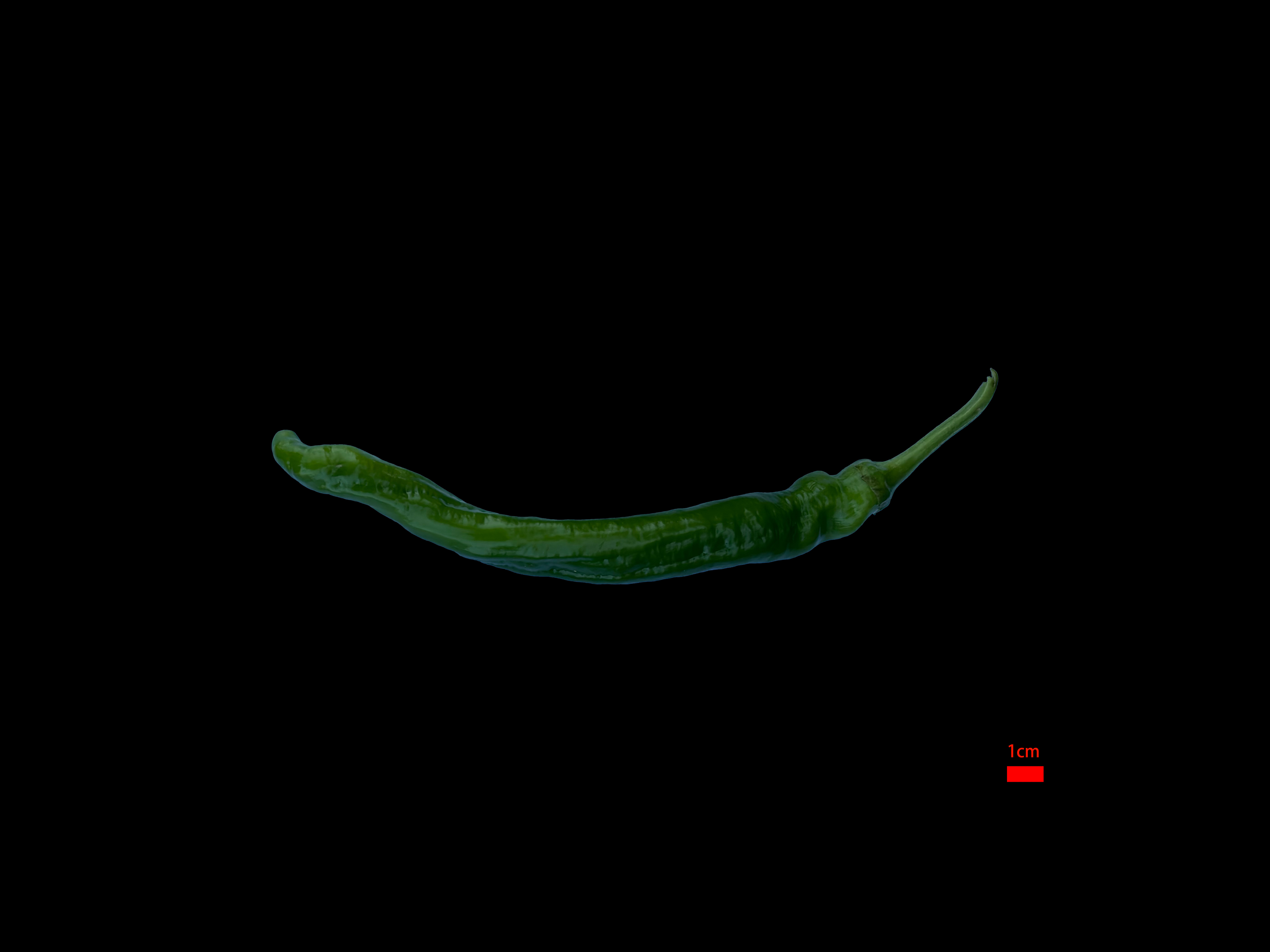

Supplement: Supplementary file 1 [file plants-15-02103-s001.zip › plants-4383327-supplementary/pepper_original_data/Goat_horn/139-2.jpg]

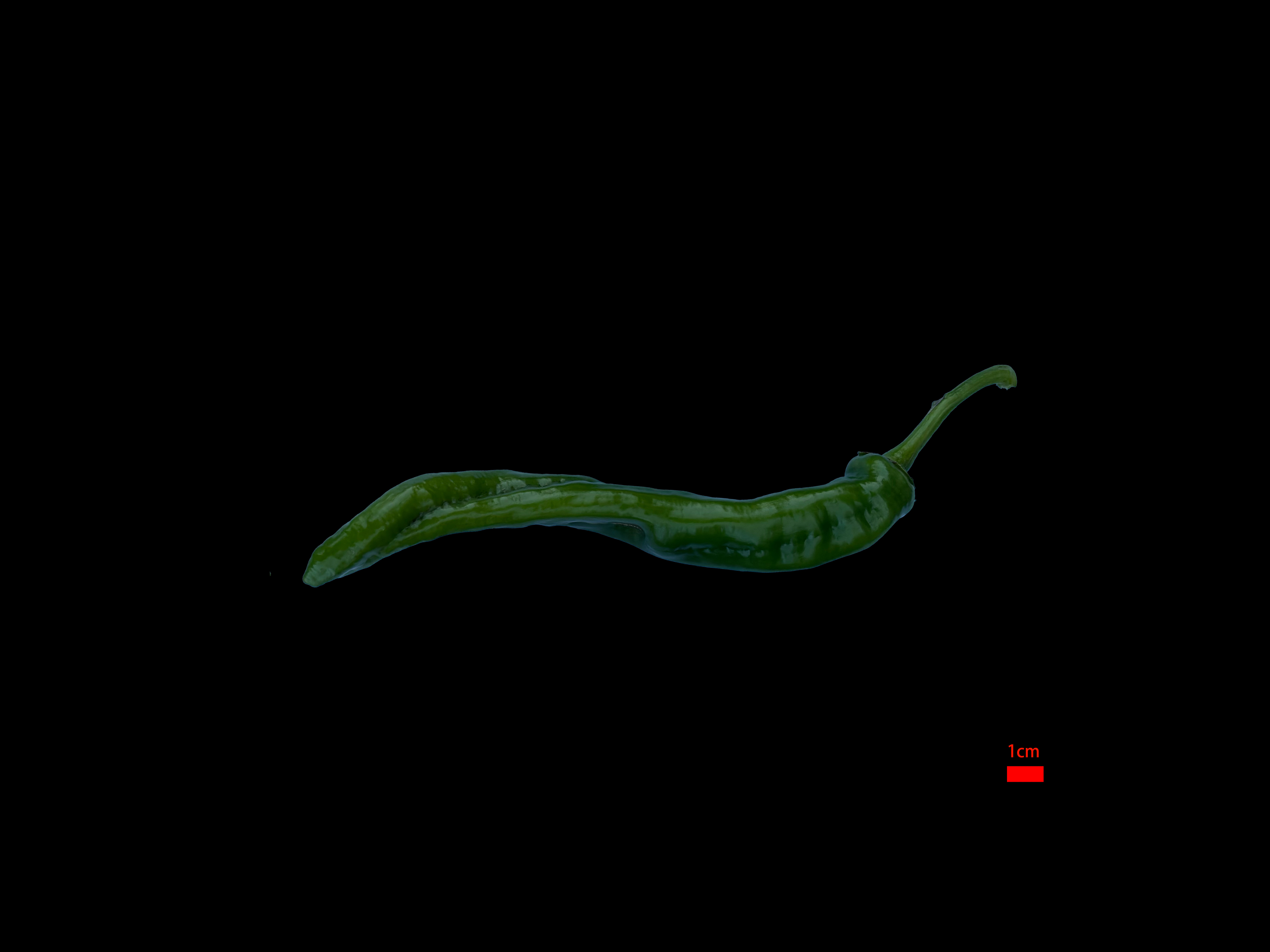

Supplement: Supplementary file 1 [file plants-15-02103-s001.zip › plants-4383327-supplementary/pepper_original_data/Goat_horn/139-3.jpg]

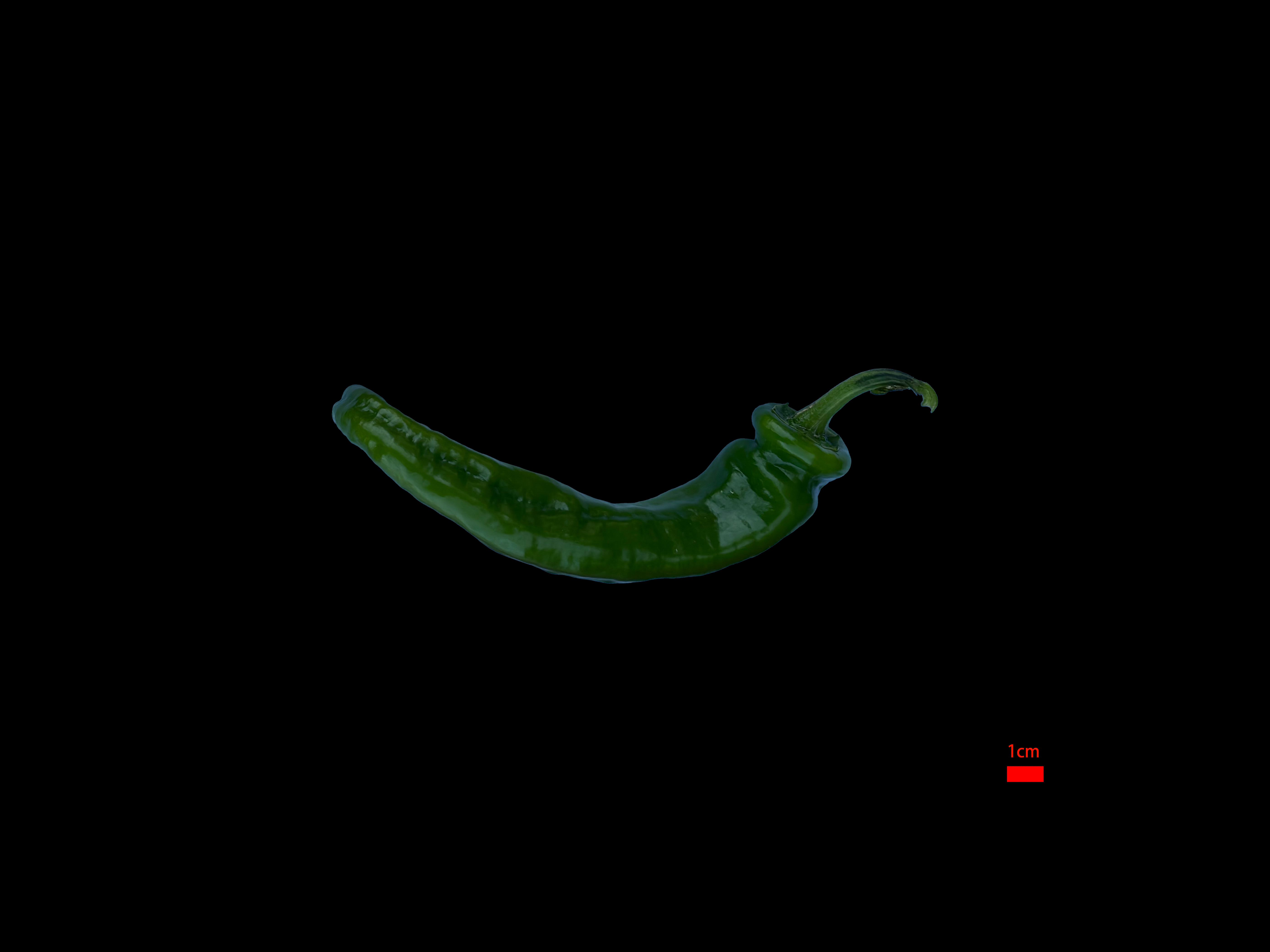

Supplement: Supplementary file 1 [file plants-15-02103-s001.zip › plants-4383327-supplementary/pepper_original_data/Goat_horn/139-4.jpg]

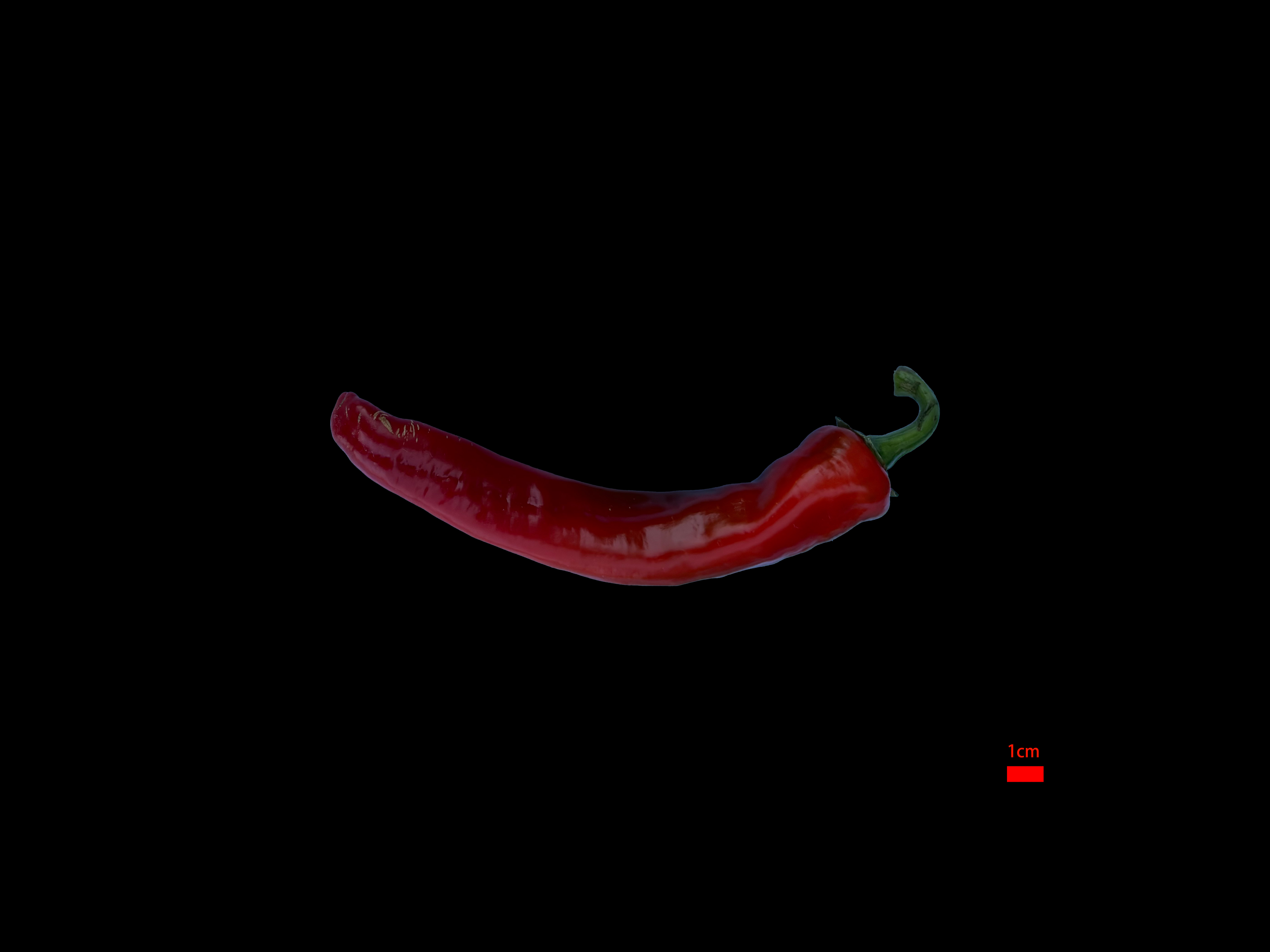

Supplement: Supplementary file 1 [file plants-15-02103-s001.zip › plants-4383327-supplementary/pepper_original_data/Goat_horn/139-5.jpg]

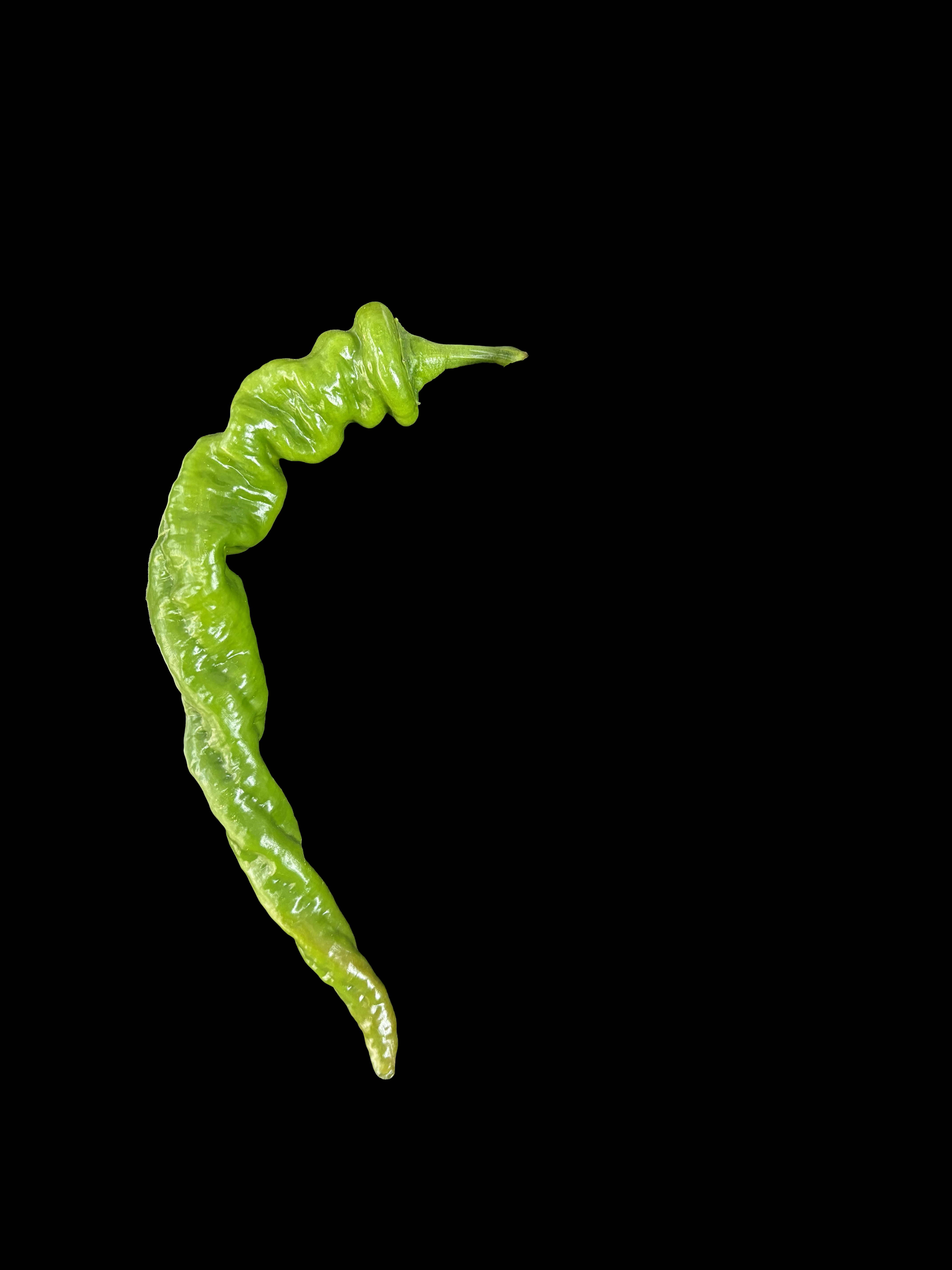

Supplement: Supplementary file 1 [file plants-15-02103-s001.zip › plants-4383327-supplementary/pepper_original_data/Goat_horn/15-2A.jpg]

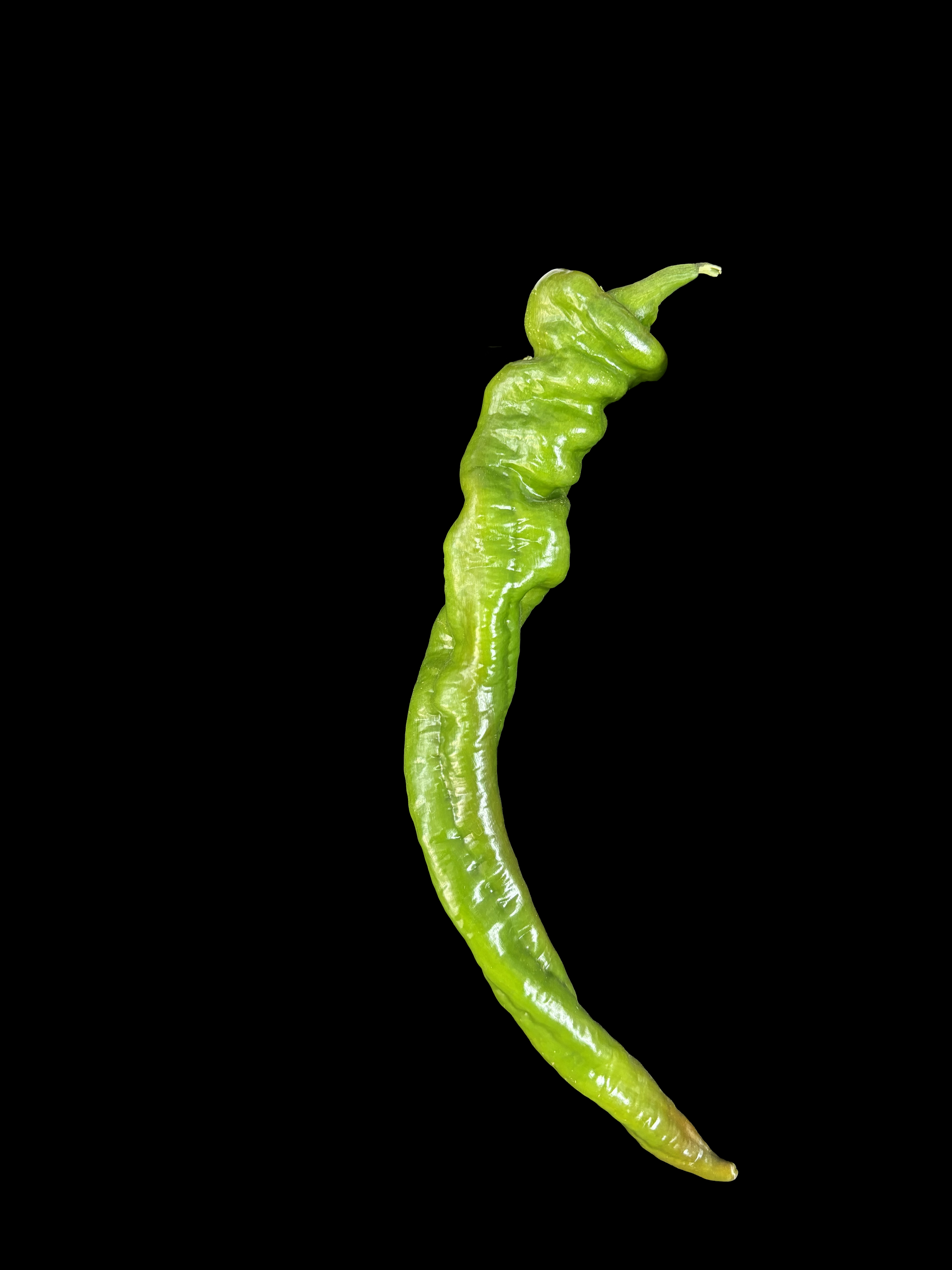

Supplement: Supplementary file 1 [file plants-15-02103-s001.zip › plants-4383327-supplementary/pepper_original_data/Goat_horn/15-2A1.jpg]

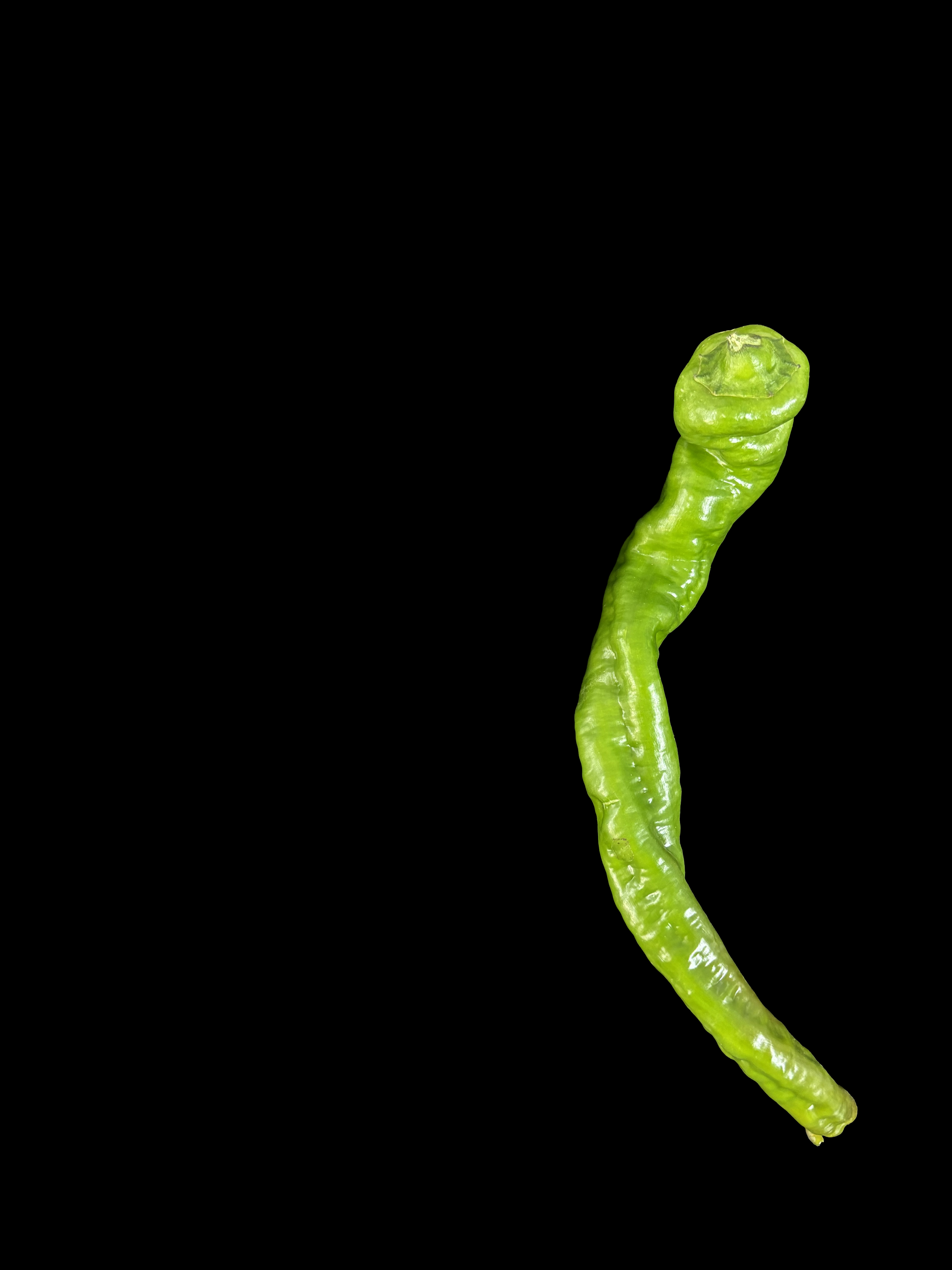

Supplement: Supplementary file 1 [file plants-15-02103-s001.zip › plants-4383327-supplementary/pepper_original_data/Goat_horn/15-2A2.jpg]

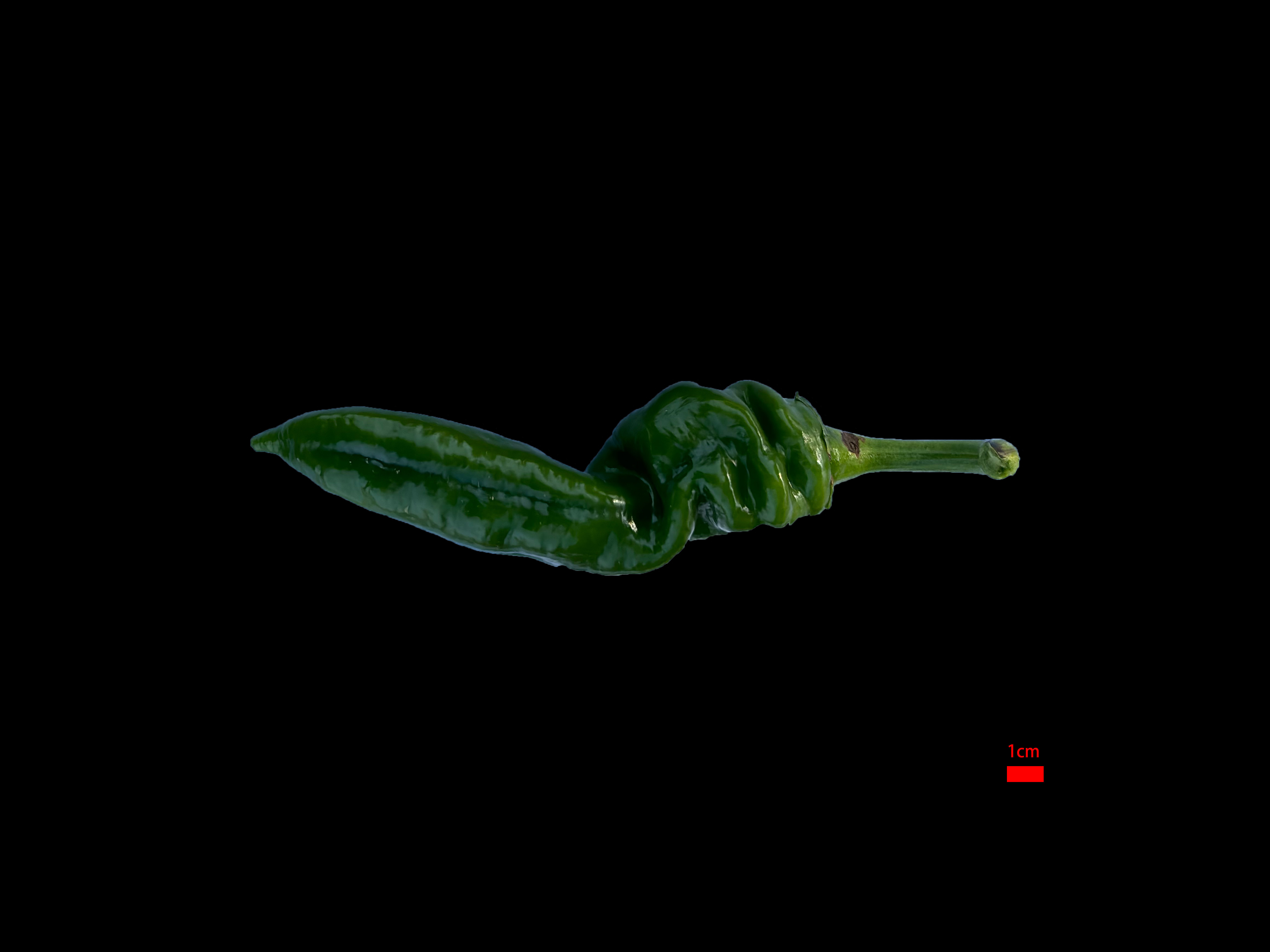

Supplement: Supplementary file 1 [file plants-15-02103-s001.zip › plants-4383327-supplementary/pepper_original_data/Goat_horn/150-5.jpg]

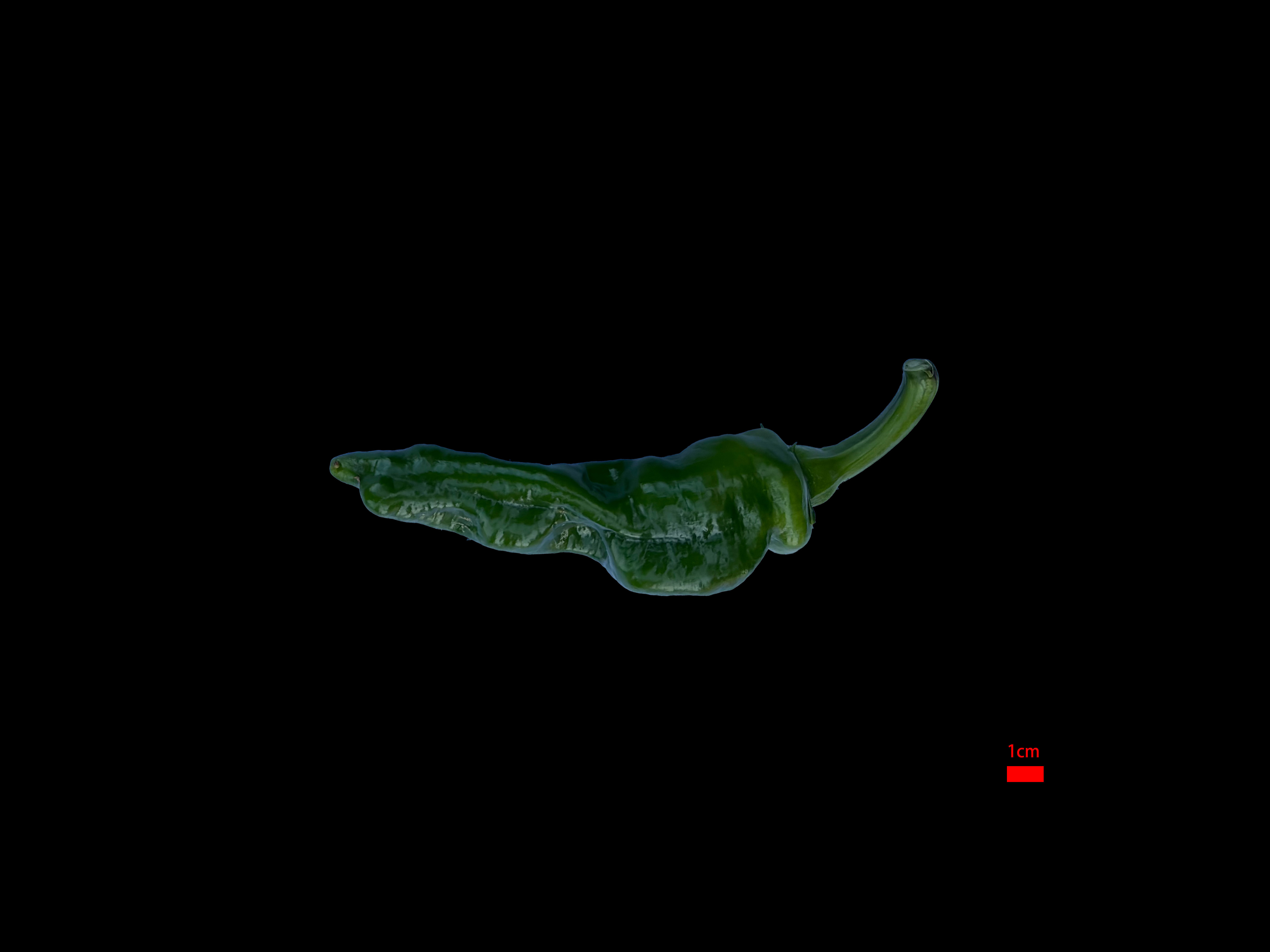

Supplement: Supplementary file 1 [file plants-15-02103-s001.zip › plants-4383327-supplementary/pepper_original_data/Goat_horn/151-1.jpg]

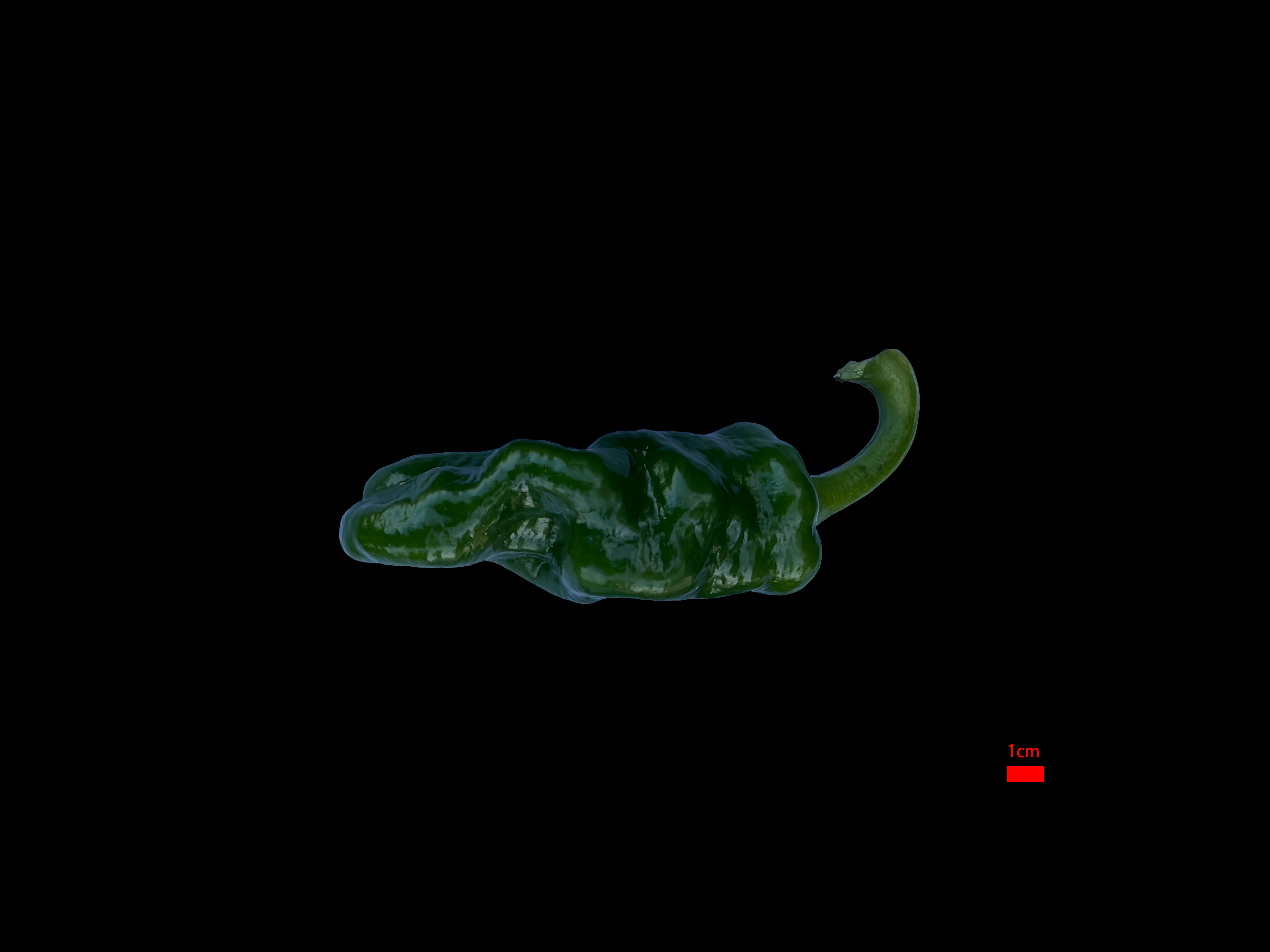

Supplement: Supplementary file 1 [file plants-15-02103-s001.zip › plants-4383327-supplementary/pepper_original_data/Goat_horn/151-2.jpg]

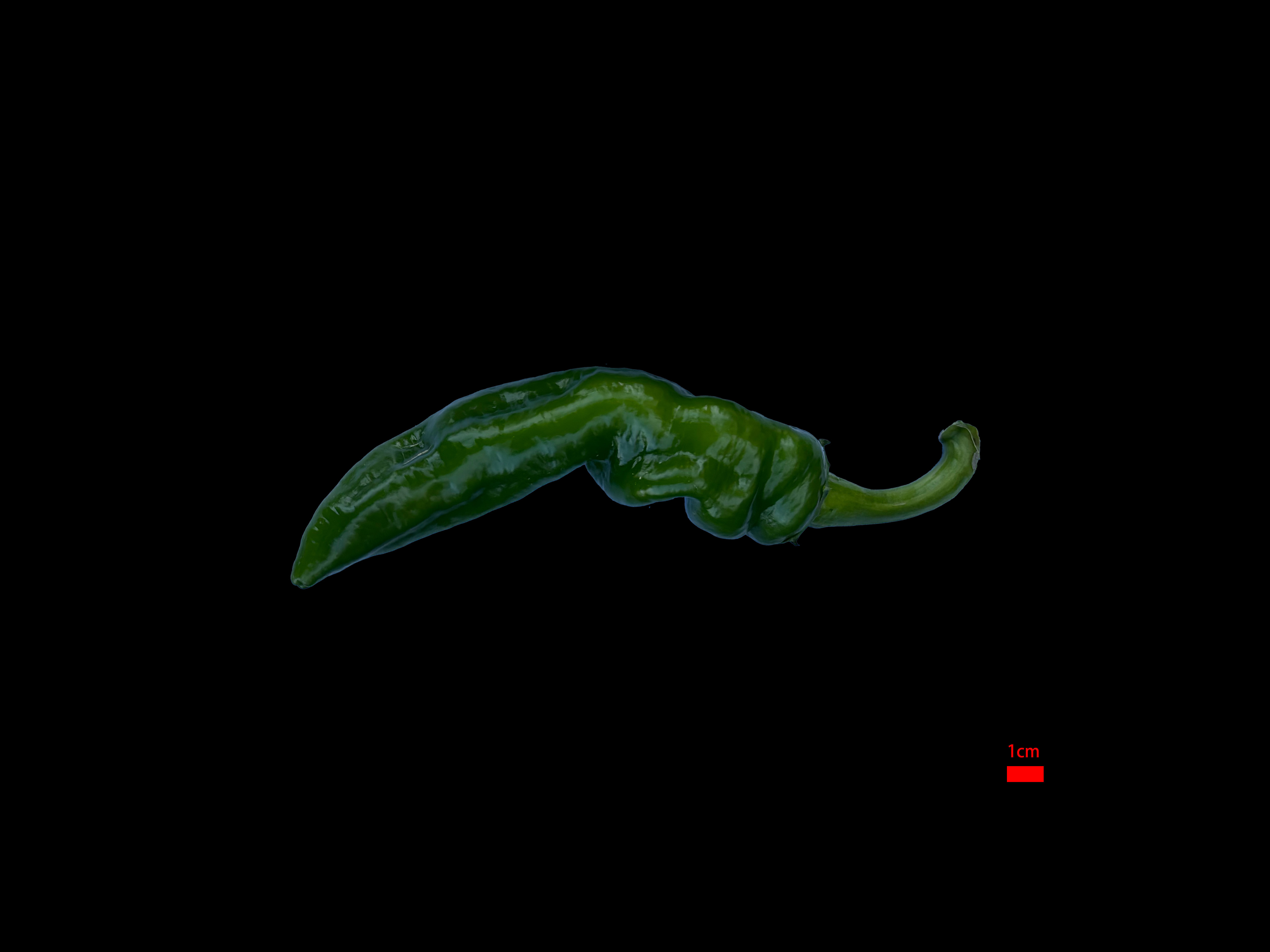

Supplement: Supplementary file 1 [file plants-15-02103-s001.zip › plants-4383327-supplementary/pepper_original_data/Goat_horn/151-3.jpg]
