# Supplementary figures and images for: A Deep Learning Model for Chili Pepper Fruit Shape Classification Using DenseNet-121 and CBAM (part 1 of 2)
Source: Plants (Basel). 2026 Jul 7;15(13):2103. doi: 10.3390/plants15132103 (PMC13364266; doi:10.3390/plants15132103)

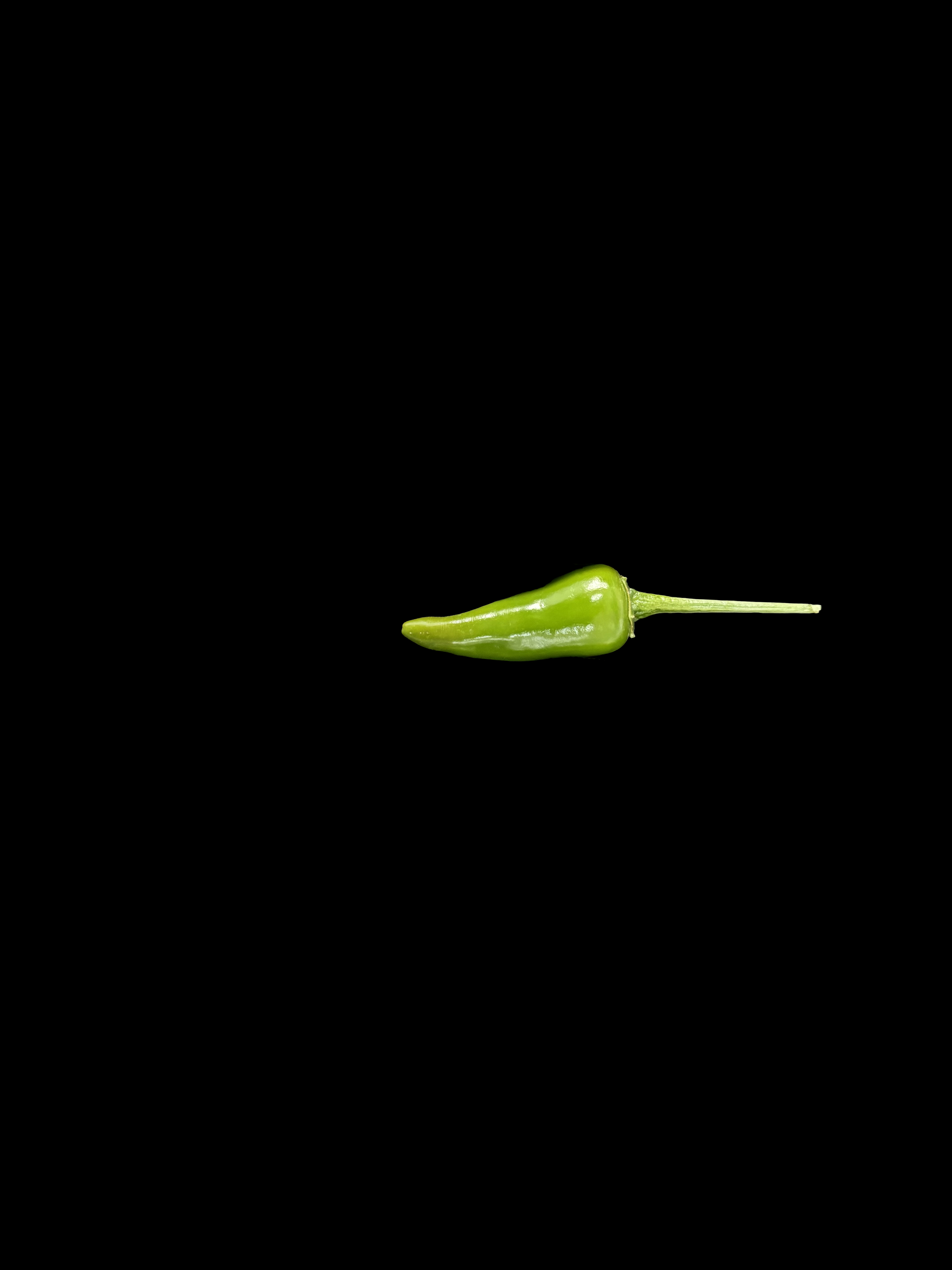

Supplement: Supplementary file 1 [file plants-15-02103-s001.zip › plants-4383327-supplementary/pepper_original_data/cone/1.1.jpg]

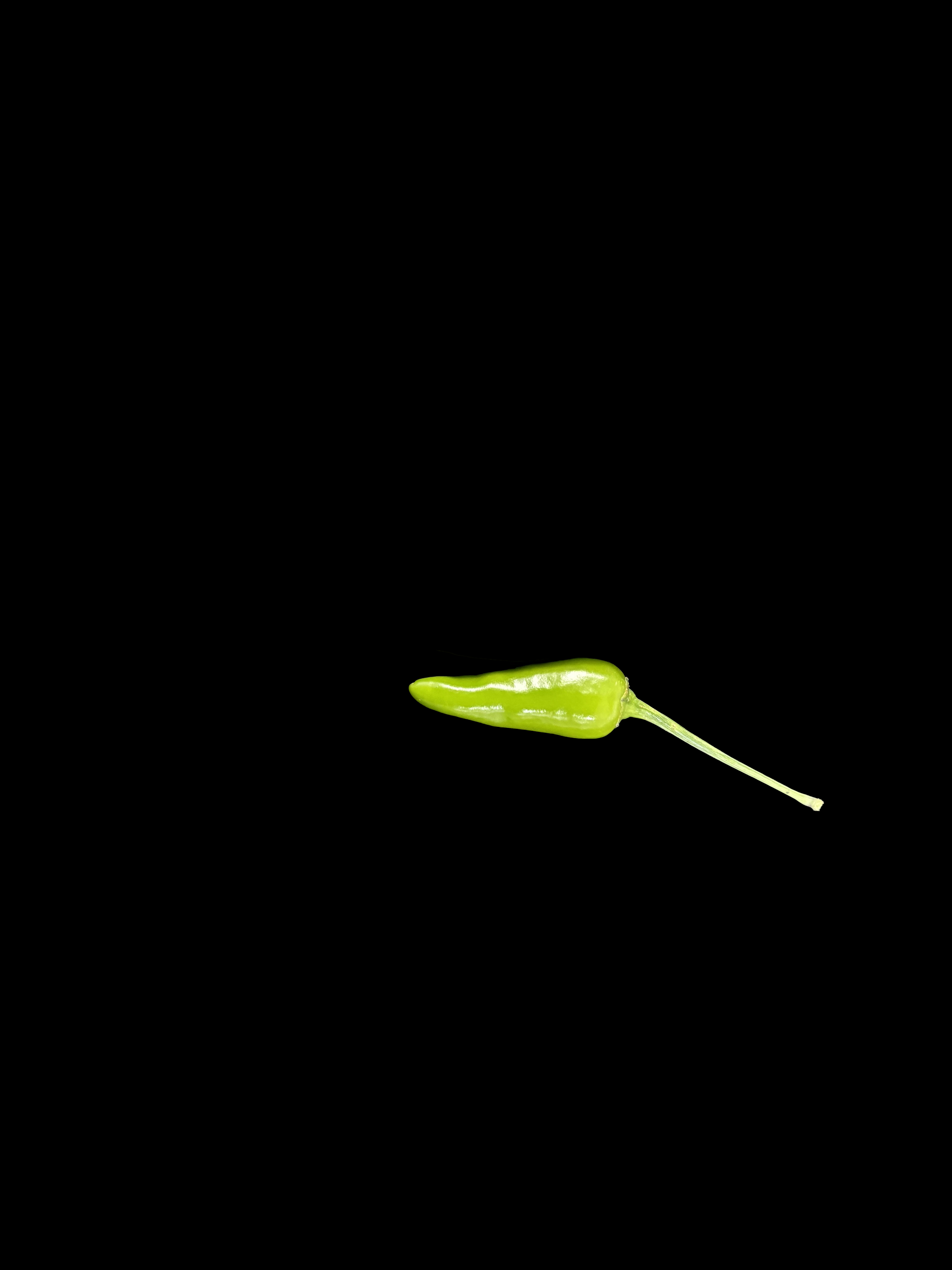

Supplement: Supplementary file 1 [file plants-15-02103-s001.zip › plants-4383327-supplementary/pepper_original_data/cone/1.2.jpg]

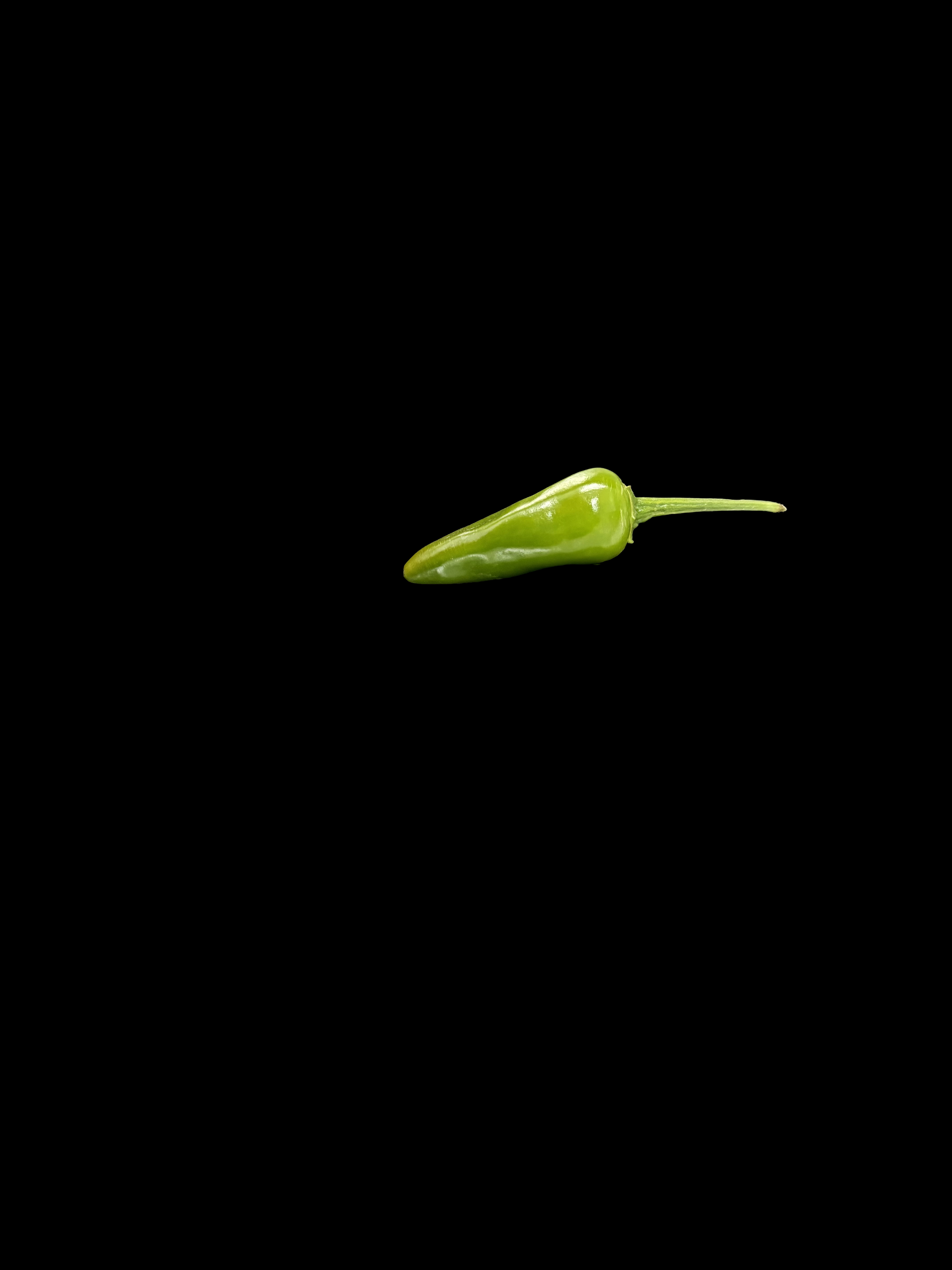

Supplement: Supplementary file 1 [file plants-15-02103-s001.zip › plants-4383327-supplementary/pepper_original_data/cone/1.jpg]

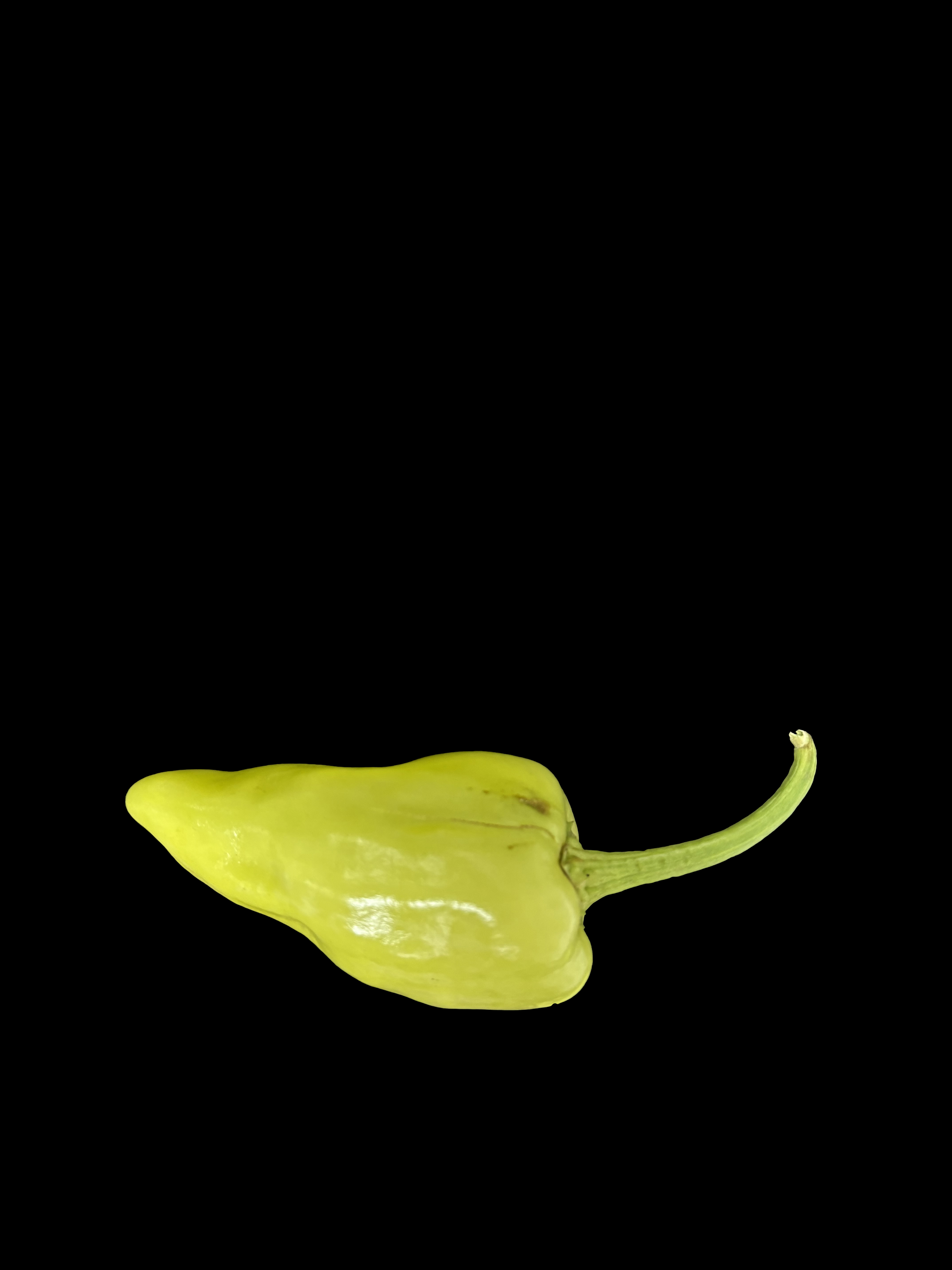

Supplement: Supplementary file 1 [file plants-15-02103-s001.zip › plants-4383327-supplementary/pepper_original_data/cone/105.1.jpg]

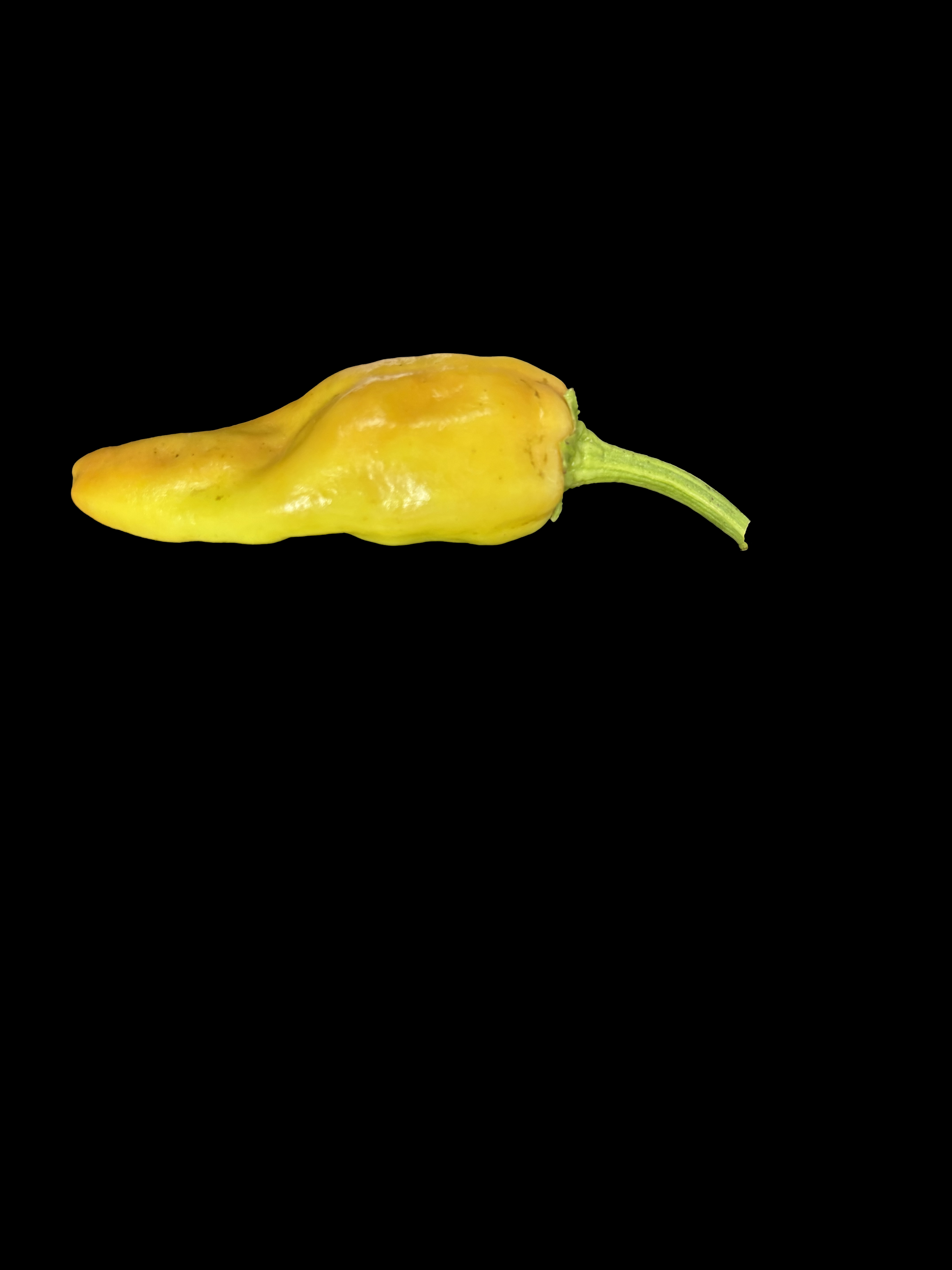

Supplement: Supplementary file 1 [file plants-15-02103-s001.zip › plants-4383327-supplementary/pepper_original_data/cone/105.11.jpg]

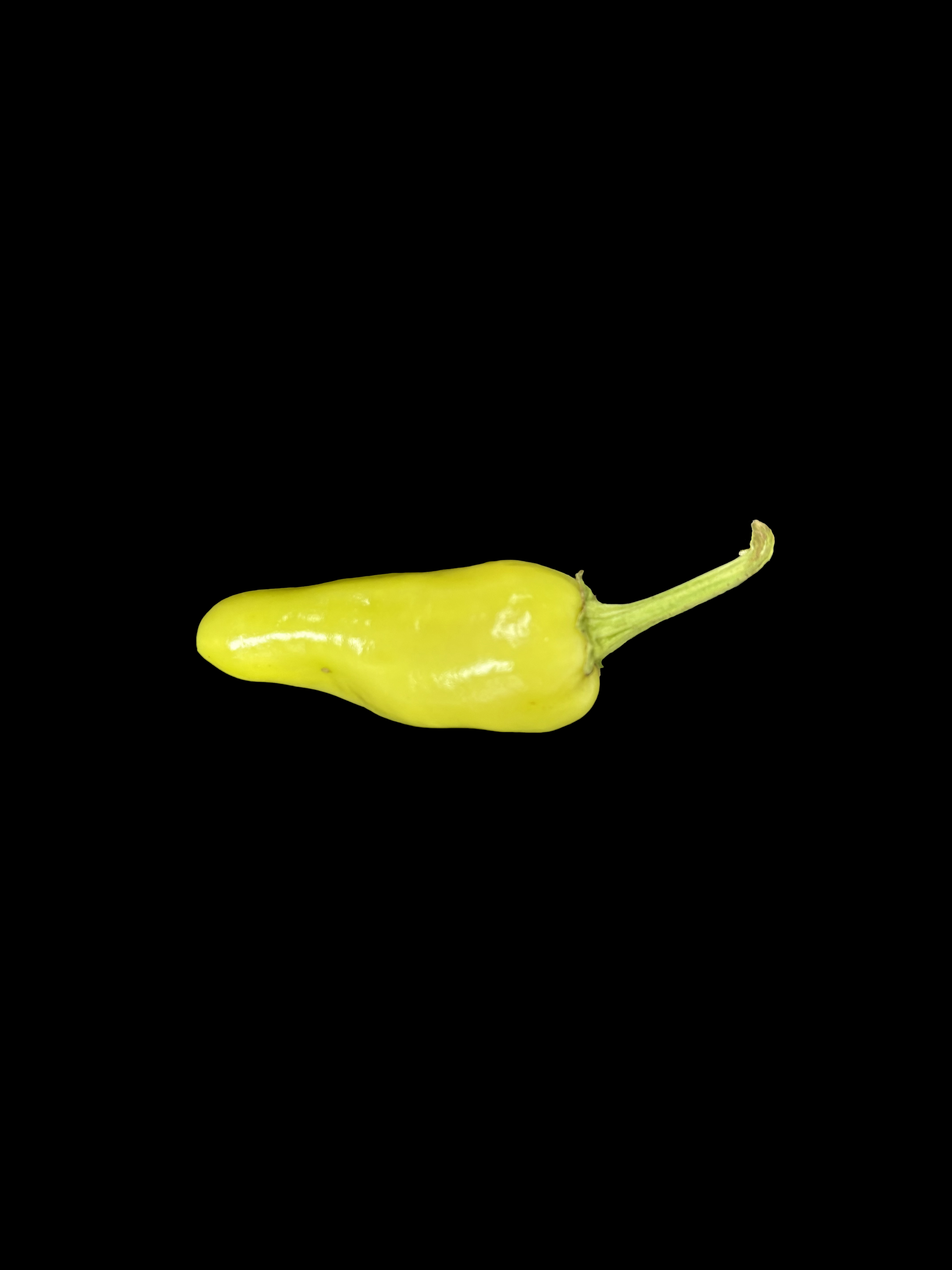

Supplement: Supplementary file 1 [file plants-15-02103-s001.zip › plants-4383327-supplementary/pepper_original_data/cone/105.jpg]

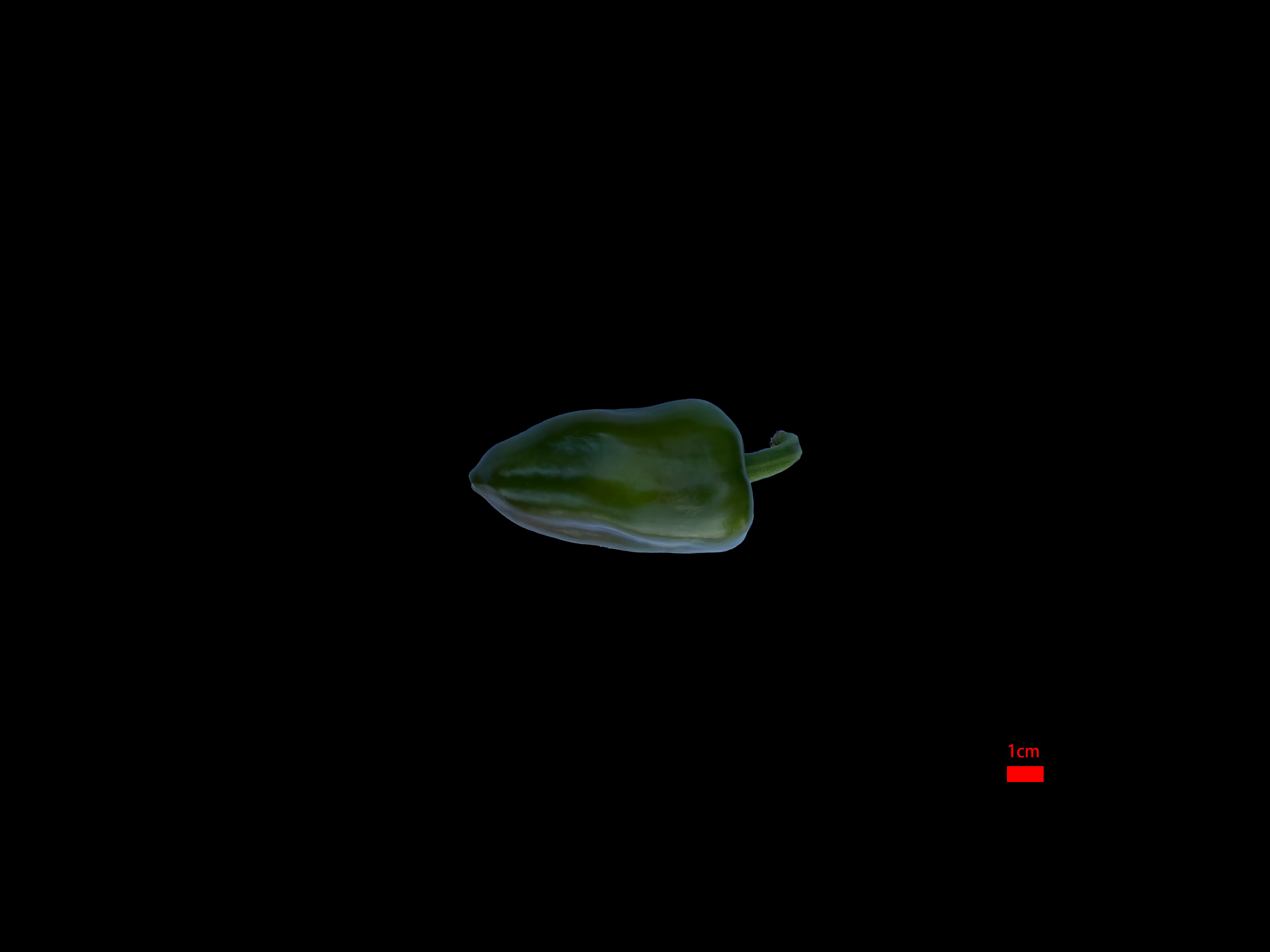

Supplement: Supplementary file 1 [file plants-15-02103-s001.zip › plants-4383327-supplementary/pepper_original_data/cone/106-1.jpg]

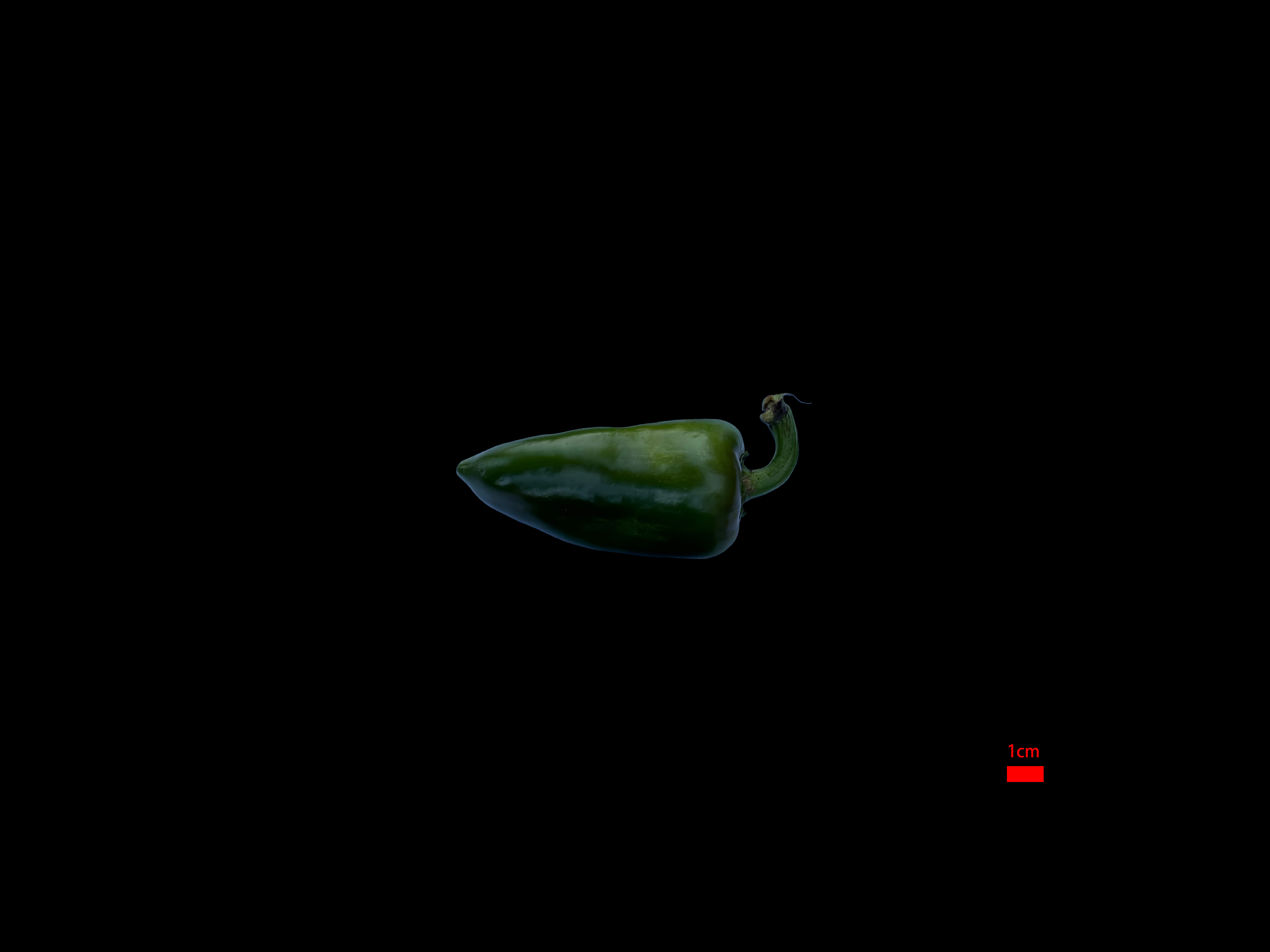

Supplement: Supplementary file 1 [file plants-15-02103-s001.zip › plants-4383327-supplementary/pepper_original_data/cone/106-2.jpg]

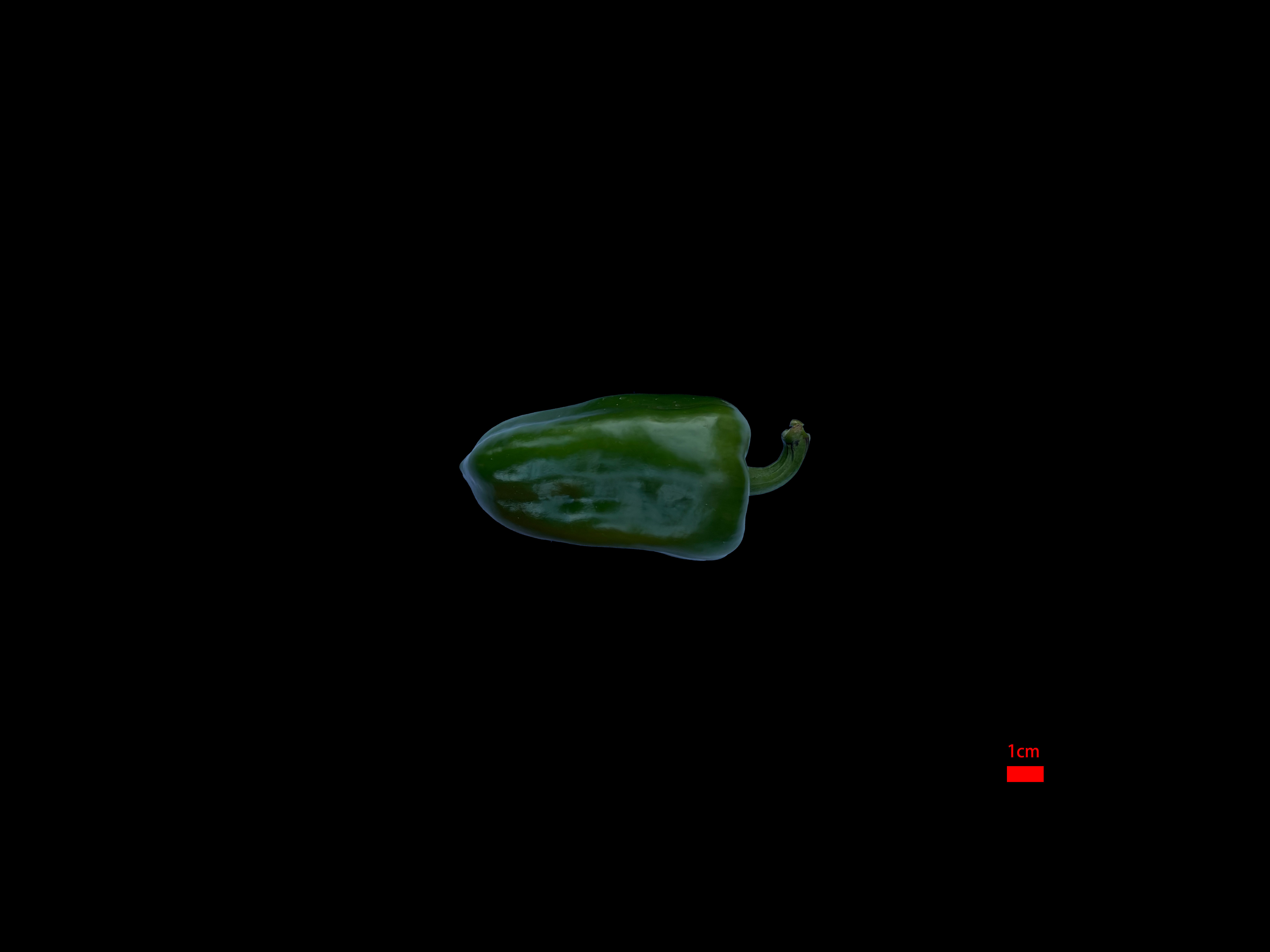

Supplement: Supplementary file 1 [file plants-15-02103-s001.zip › plants-4383327-supplementary/pepper_original_data/cone/106-3.jpg]

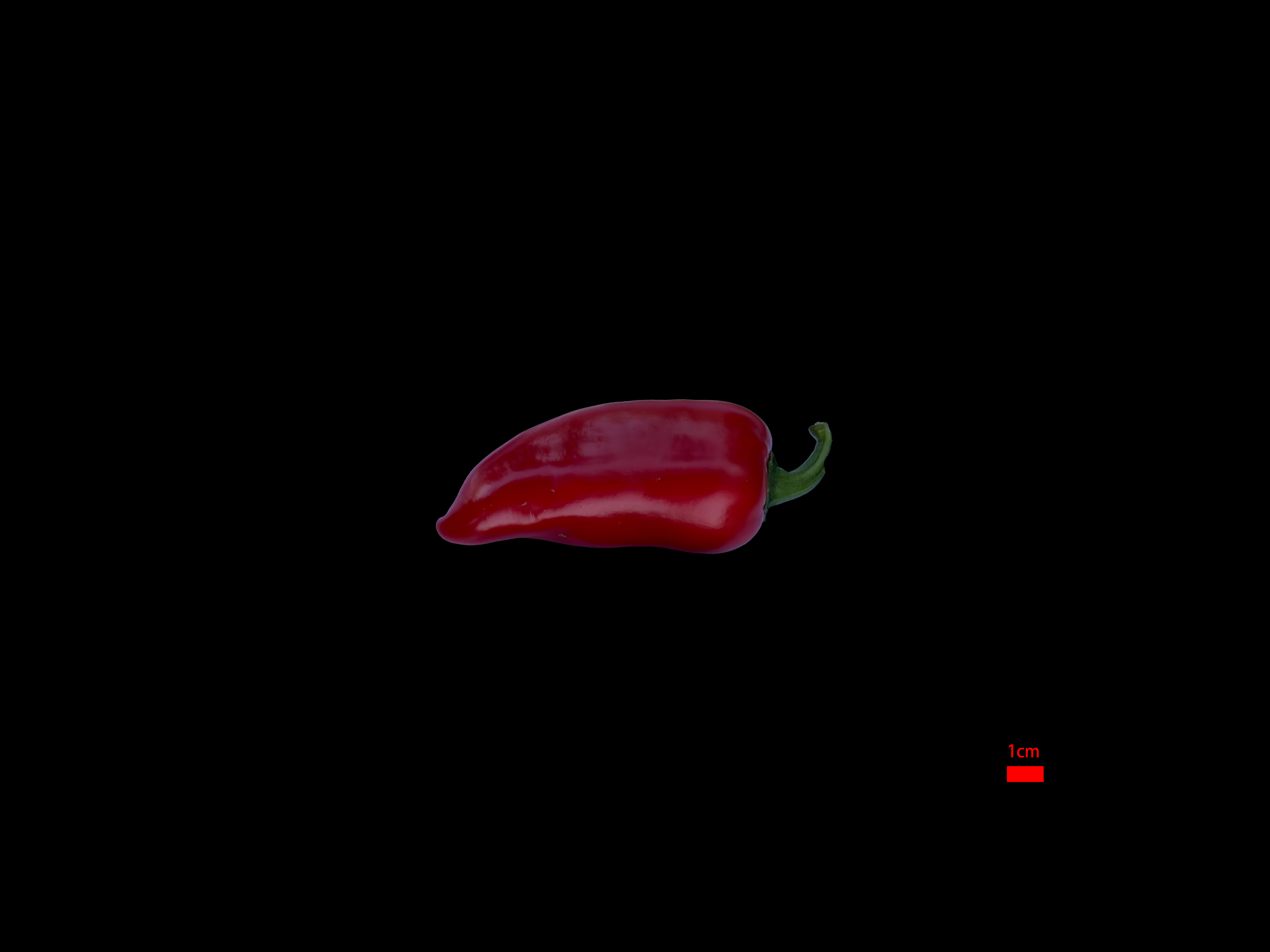

Supplement: Supplementary file 1 [file plants-15-02103-s001.zip › plants-4383327-supplementary/pepper_original_data/cone/106-5.jpg]

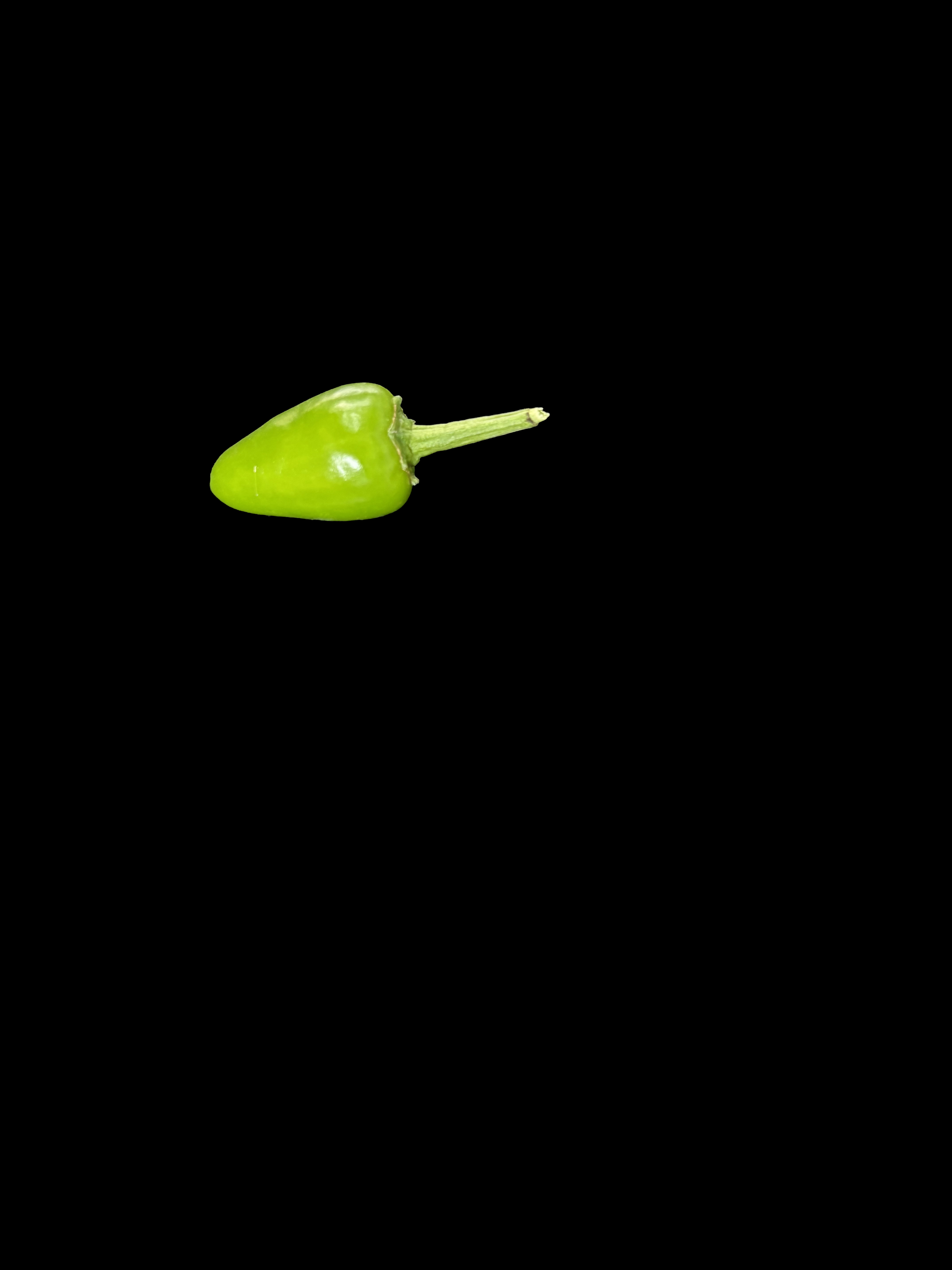

Supplement: Supplementary file 1 [file plants-15-02103-s001.zip › plants-4383327-supplementary/pepper_original_data/cone/111.1.jpg]

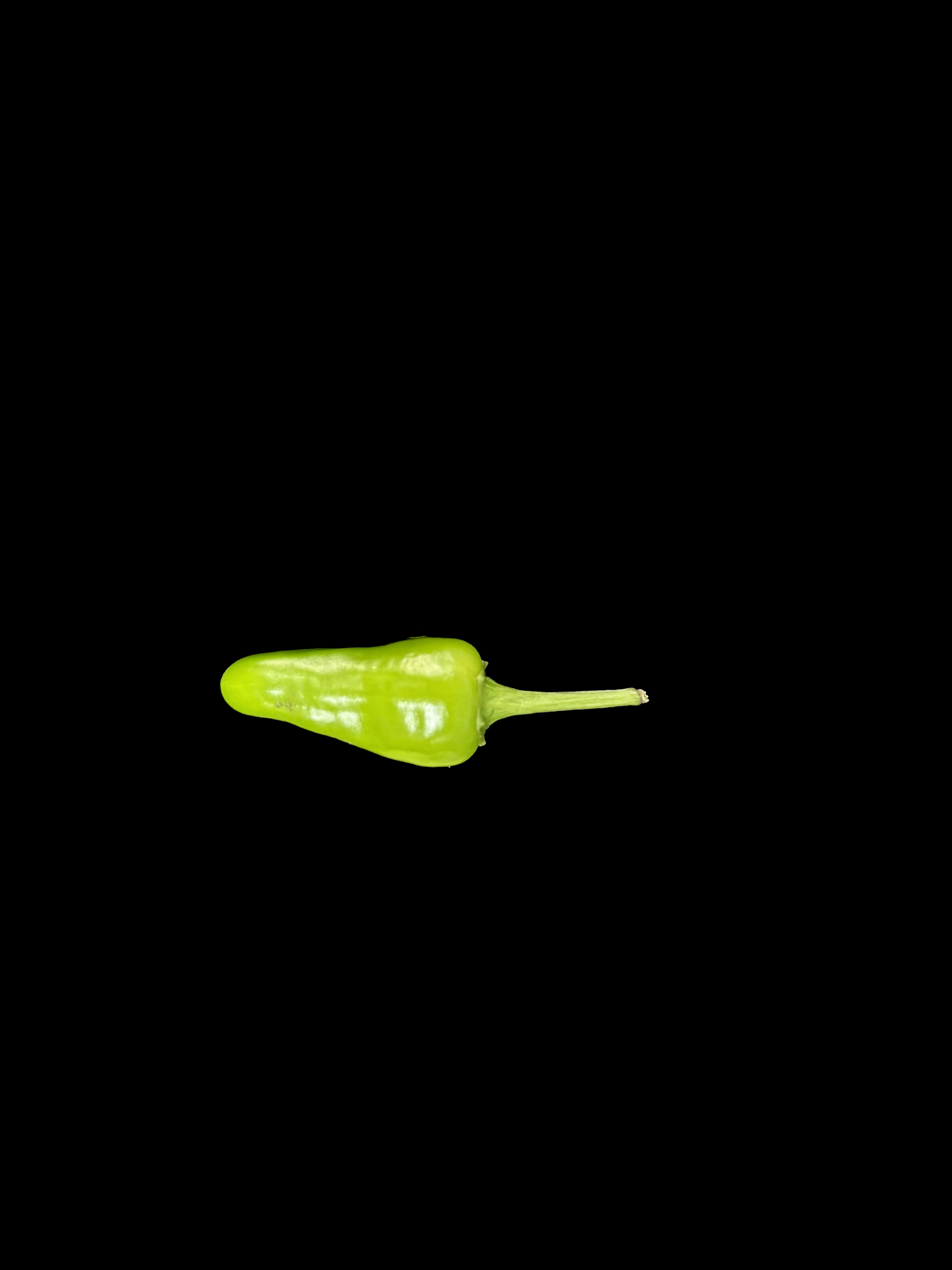

Supplement: Supplementary file 1 [file plants-15-02103-s001.zip › plants-4383327-supplementary/pepper_original_data/cone/111.2.jpg]

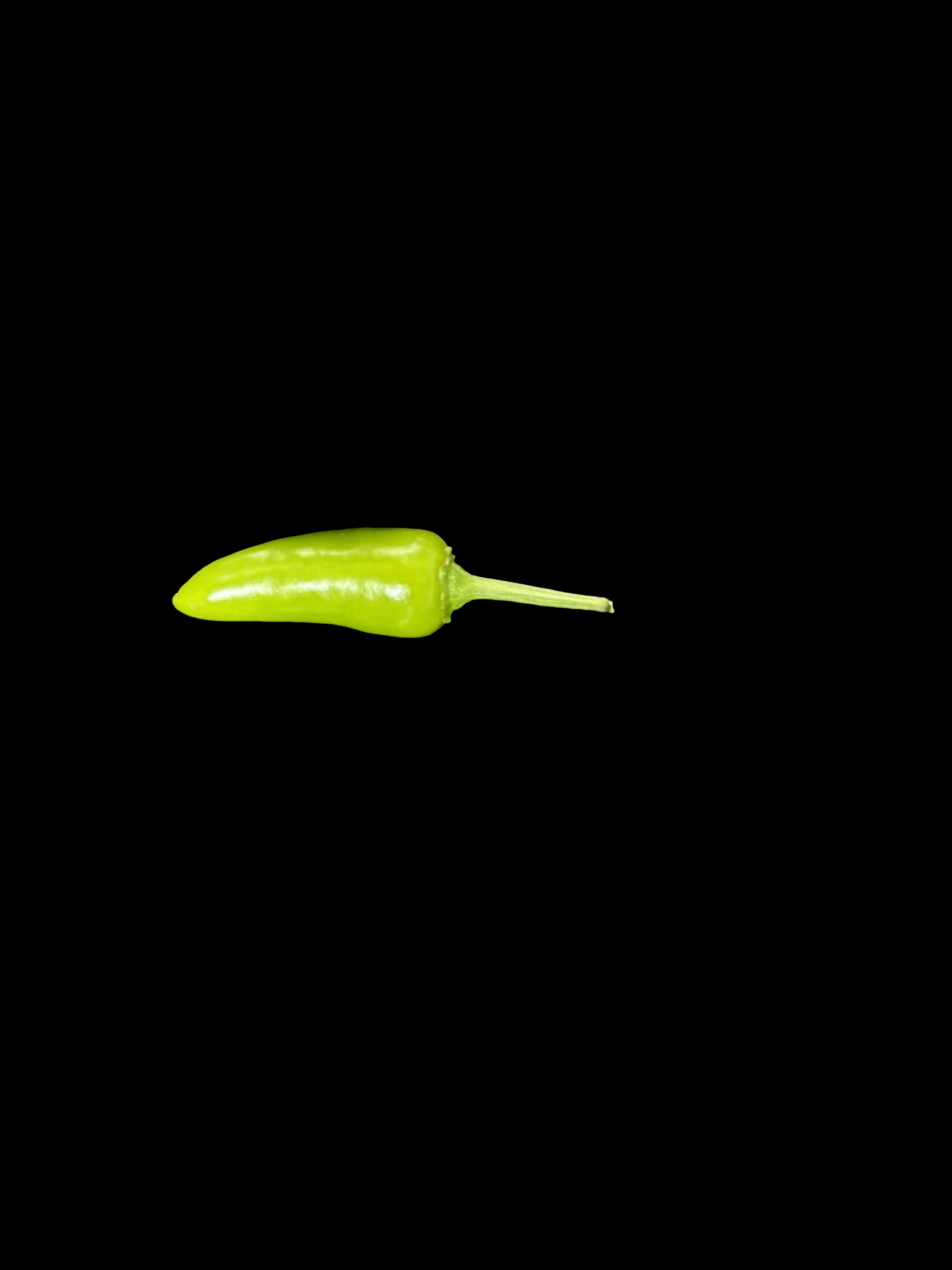

Supplement: Supplementary file 1 [file plants-15-02103-s001.zip › plants-4383327-supplementary/pepper_original_data/cone/111.jpg]

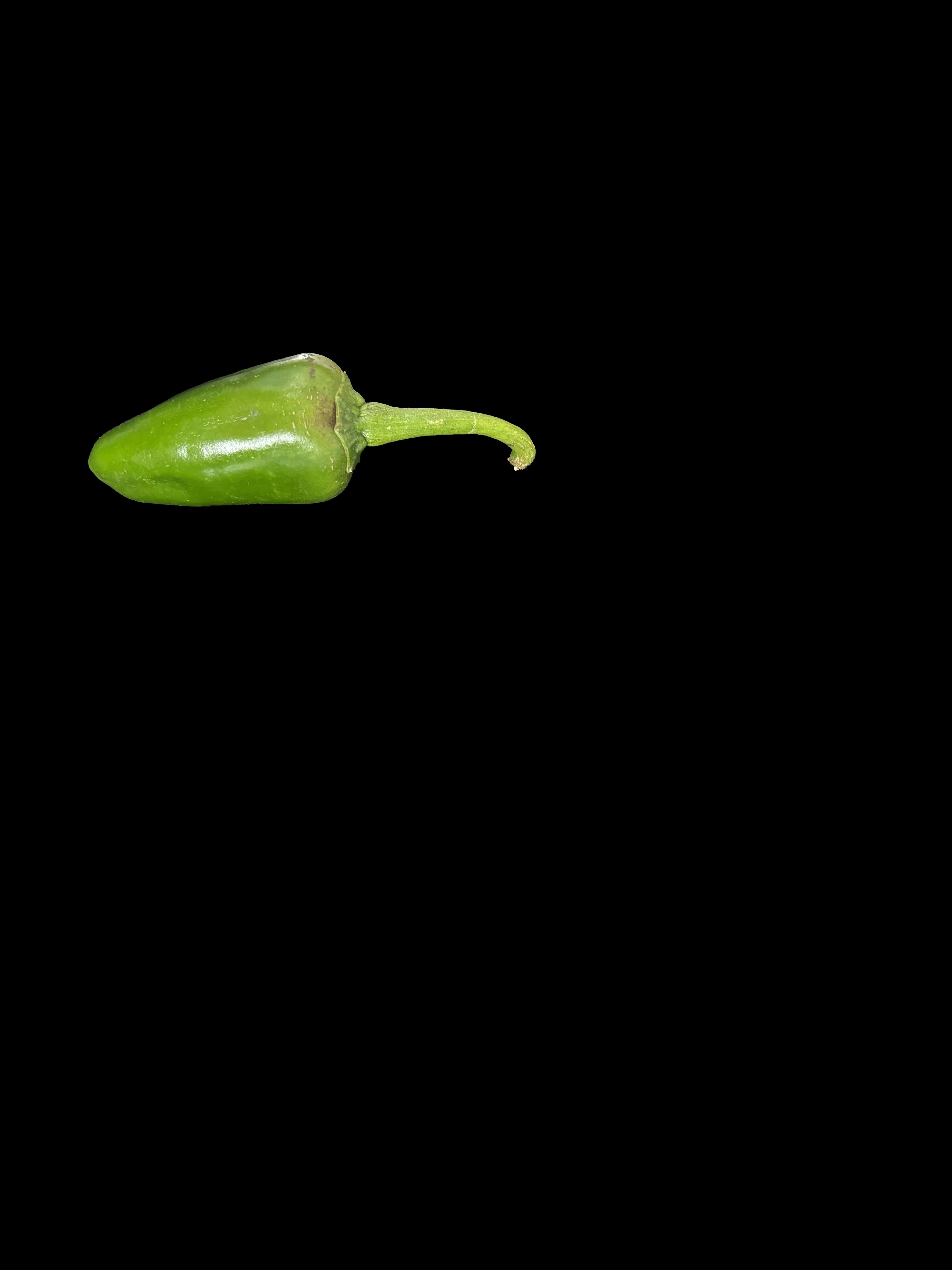

Supplement: Supplementary file 1 [file plants-15-02103-s001.zip › plants-4383327-supplementary/pepper_original_data/cone/112.jpg]

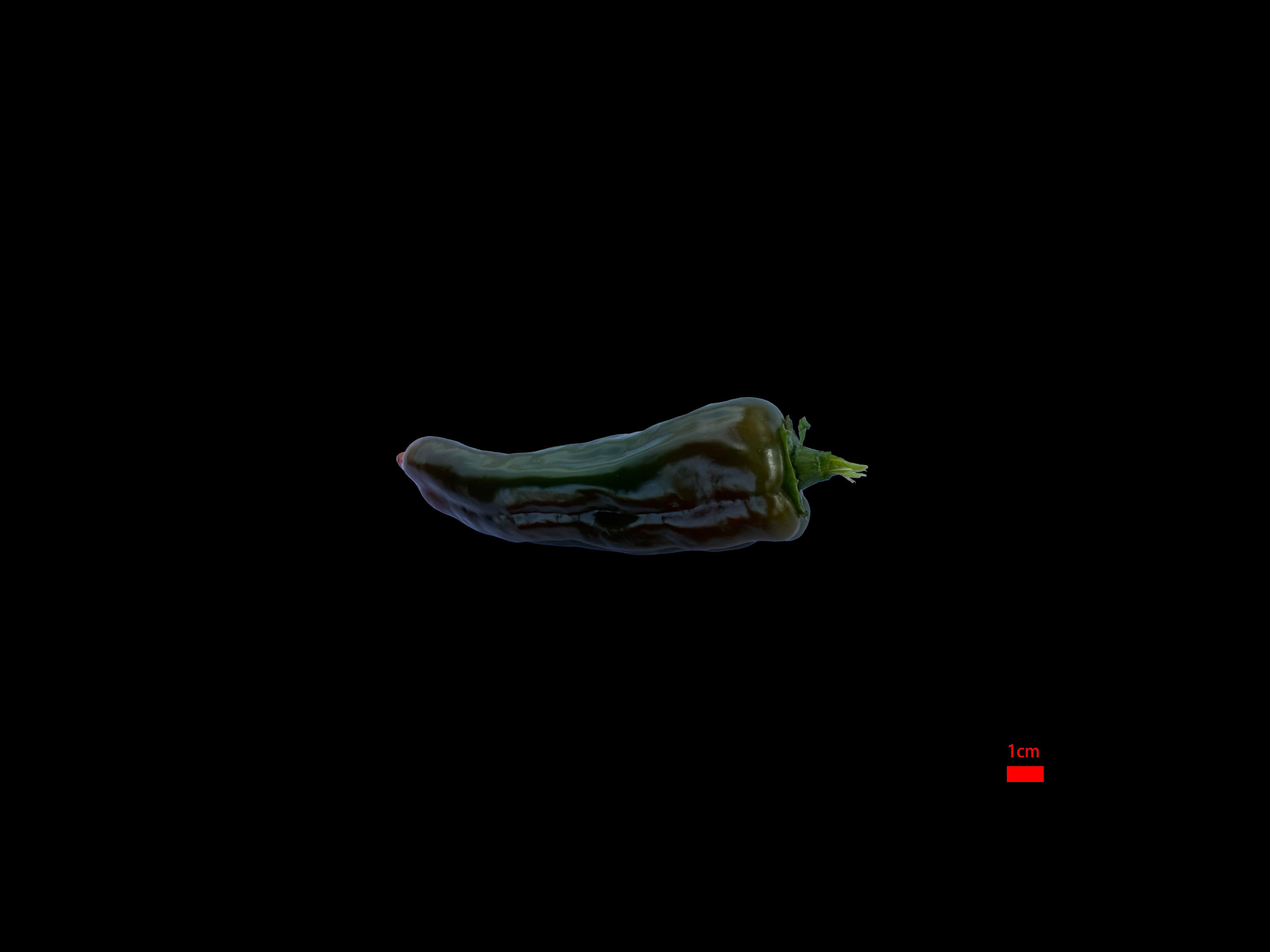

Supplement: Supplementary file 1 [file plants-15-02103-s001.zip › plants-4383327-supplementary/pepper_original_data/cone/118-5.jpg]

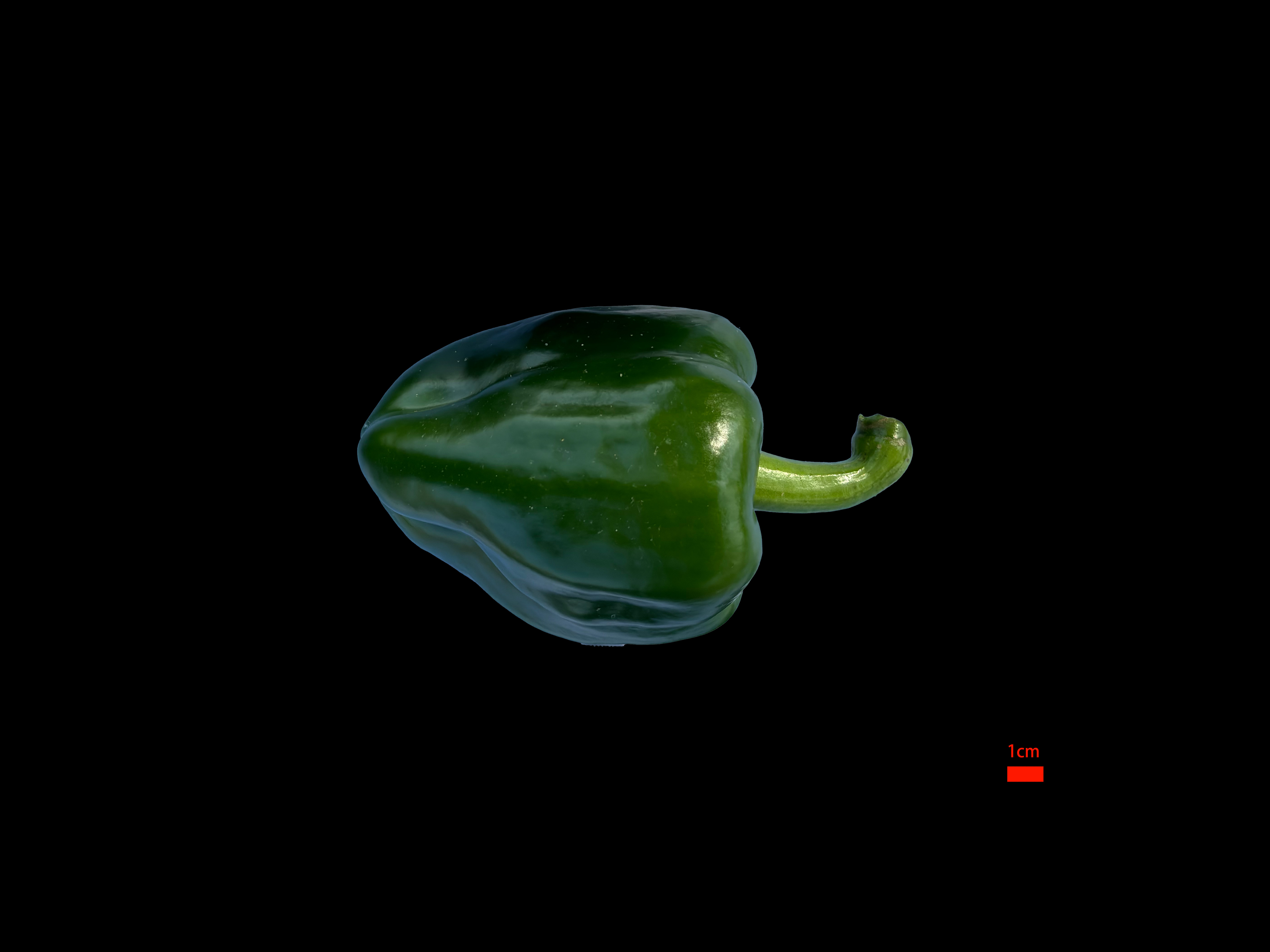

Supplement: Supplementary file 1 [file plants-15-02103-s001.zip › plants-4383327-supplementary/pepper_original_data/cone/119-3.jpg]

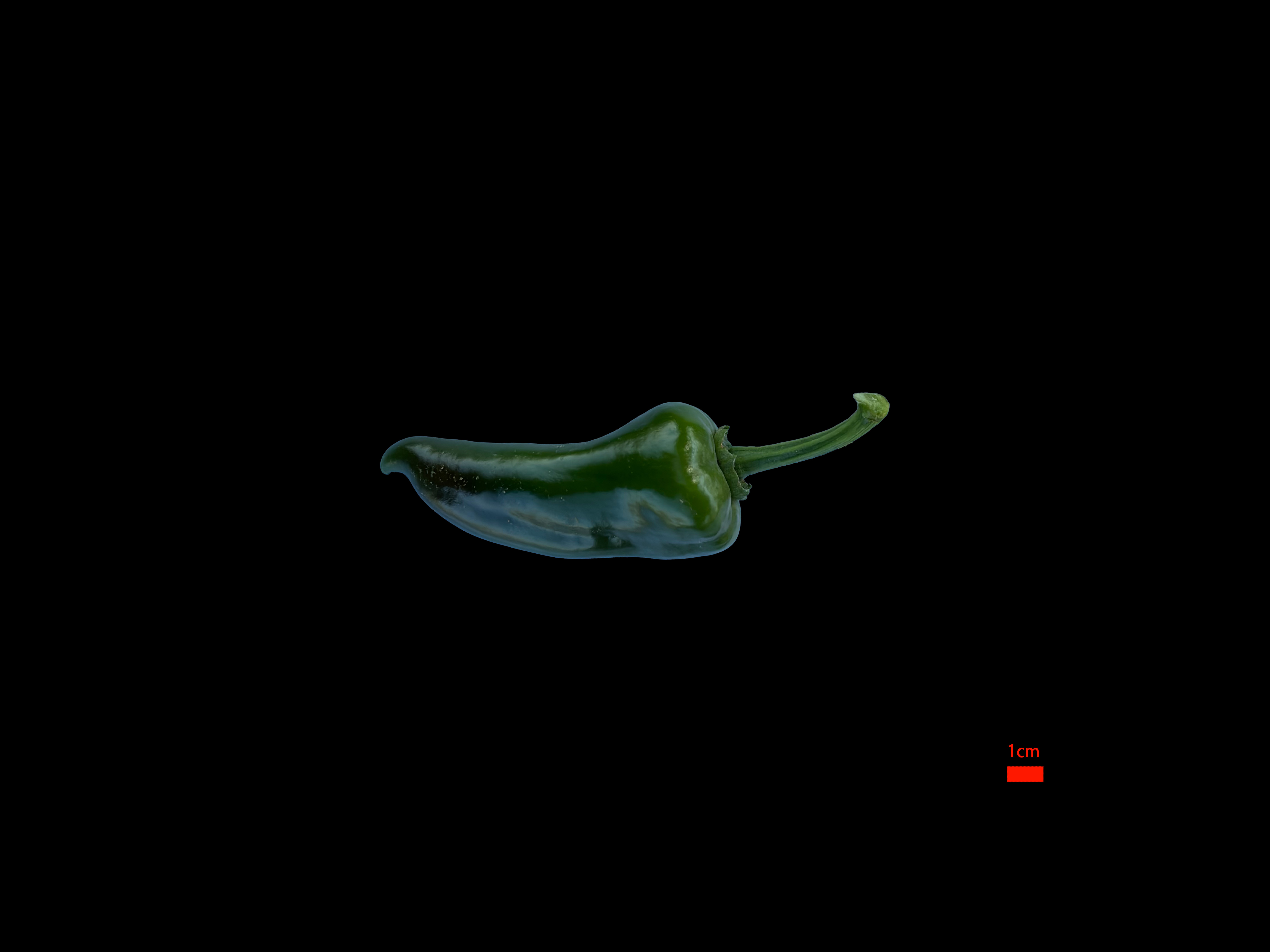

Supplement: Supplementary file 1 [file plants-15-02103-s001.zip › plants-4383327-supplementary/pepper_original_data/cone/120-10.jpg]

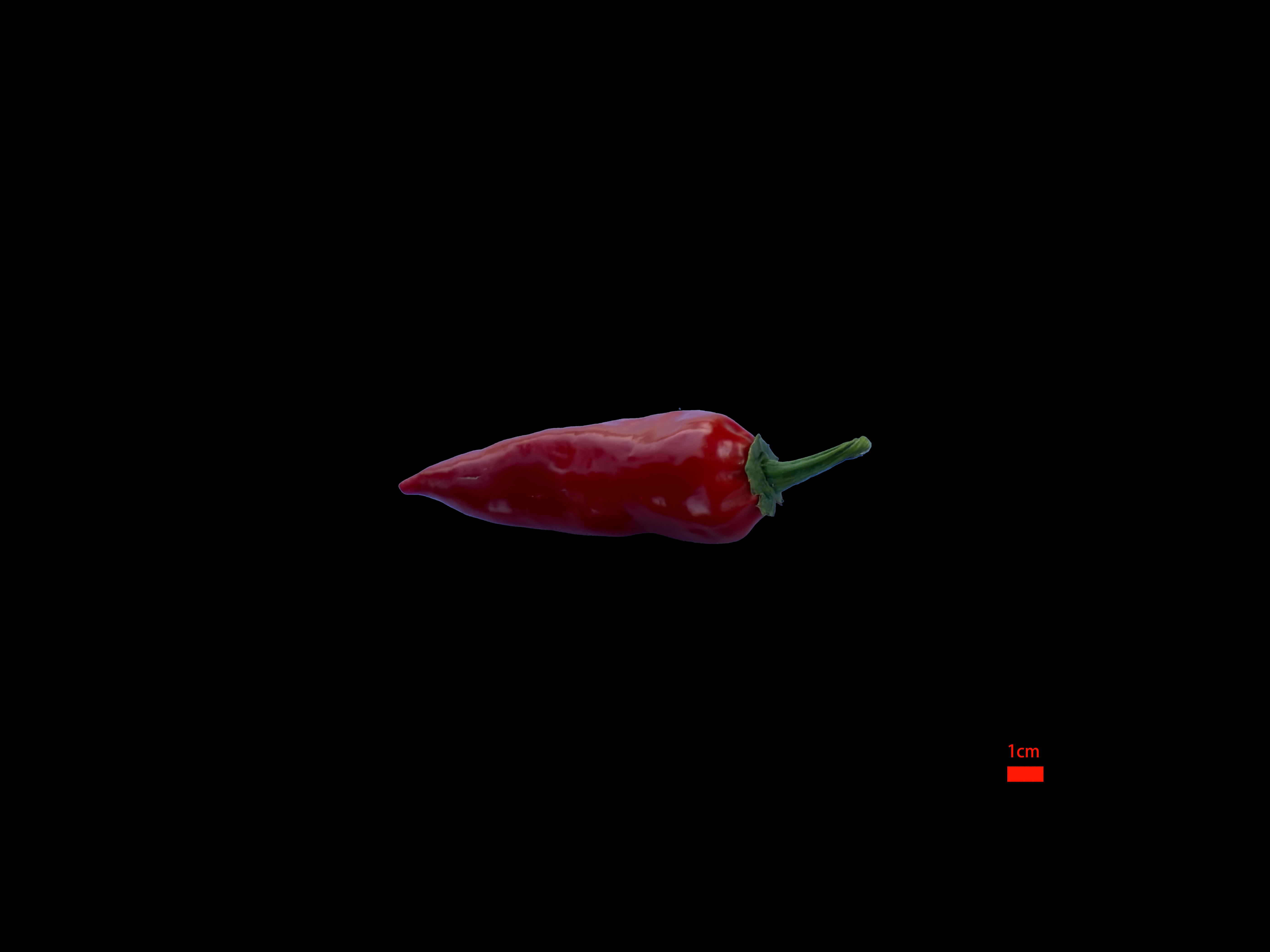

Supplement: Supplementary file 1 [file plants-15-02103-s001.zip › plants-4383327-supplementary/pepper_original_data/cone/120-11.jpg]

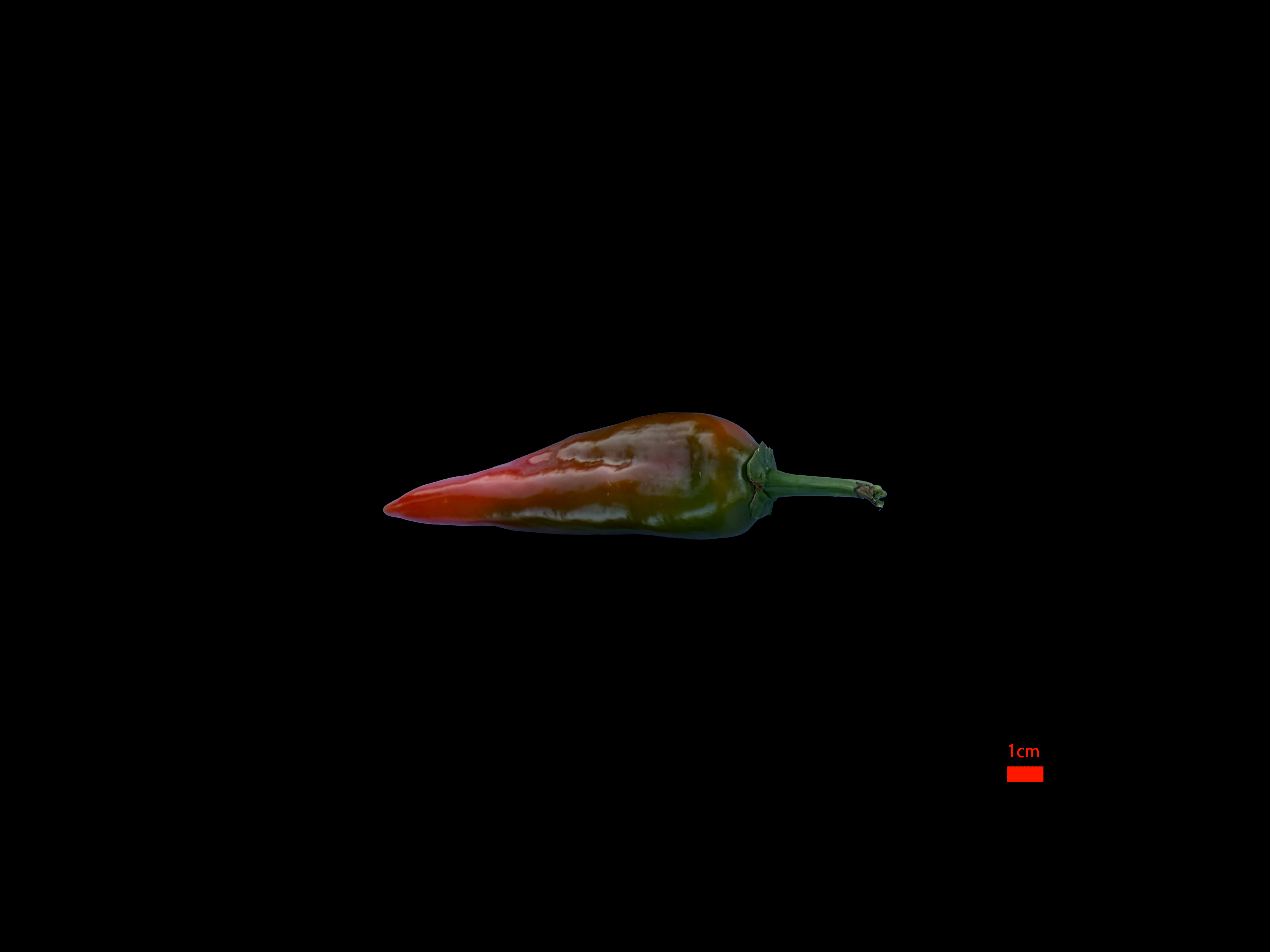

Supplement: Supplementary file 1 [file plants-15-02103-s001.zip › plants-4383327-supplementary/pepper_original_data/cone/120-12.jpg]

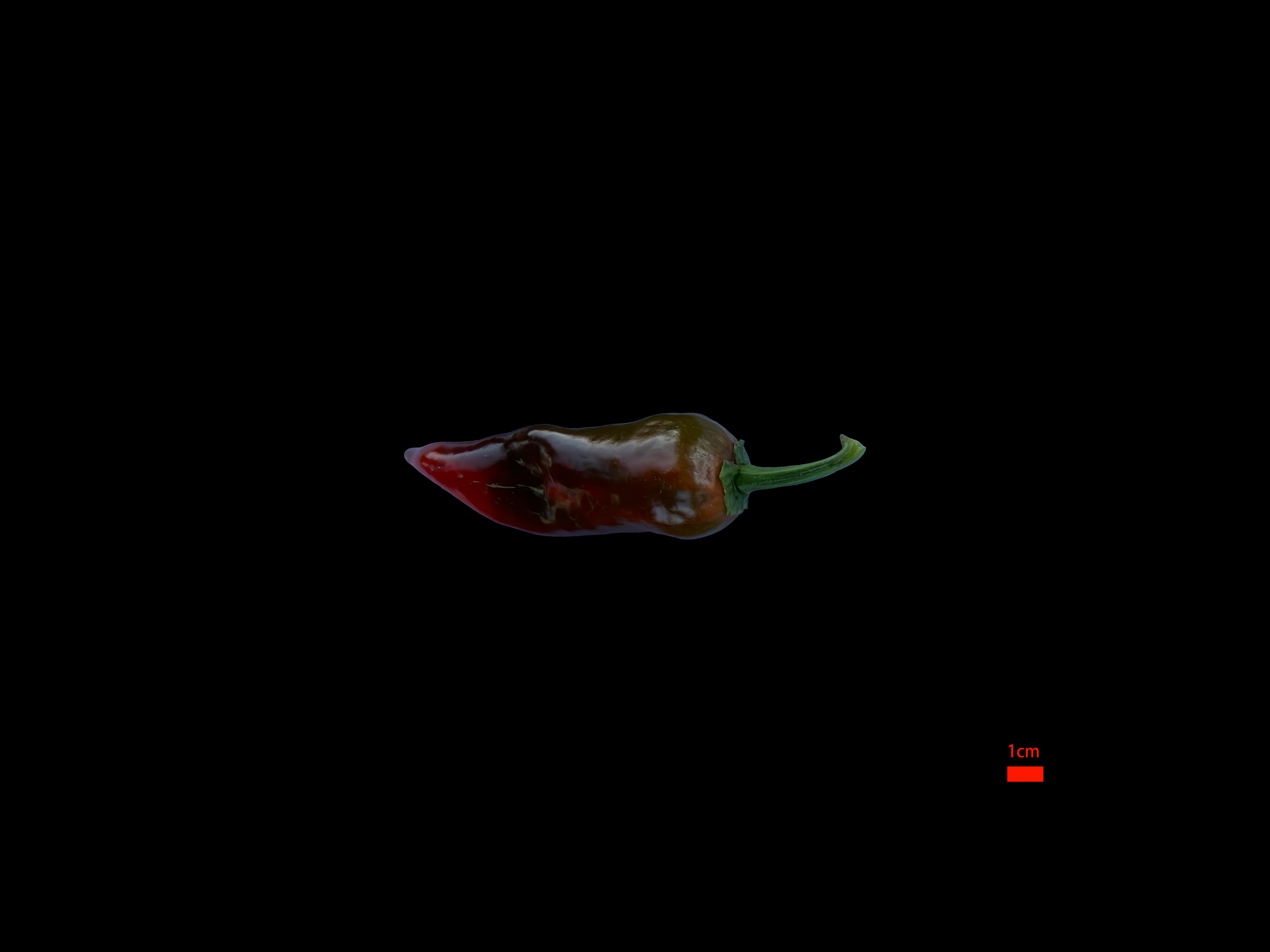

Supplement: Supplementary file 1 [file plants-15-02103-s001.zip › plants-4383327-supplementary/pepper_original_data/cone/120-13.jpg]

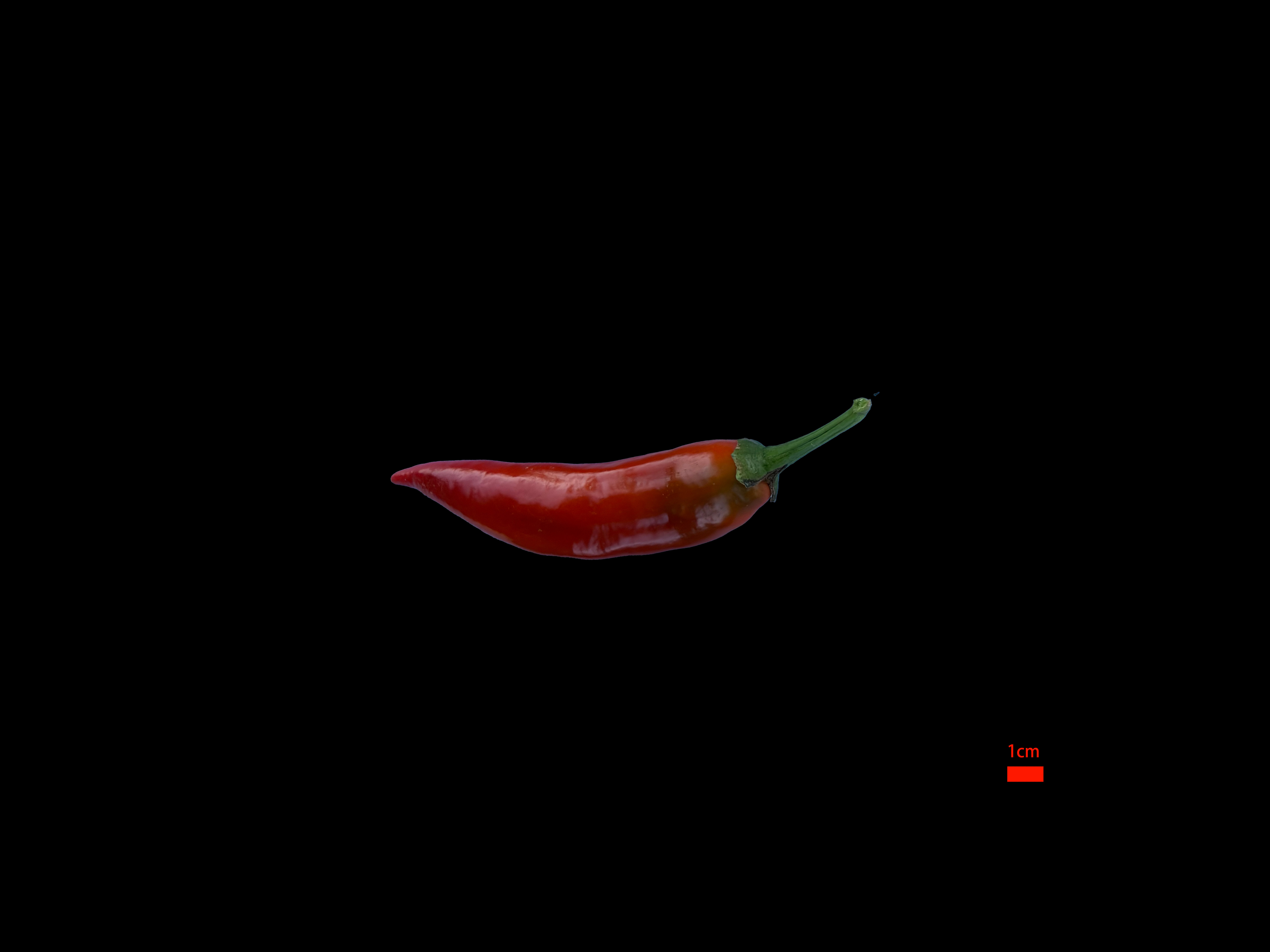

Supplement: Supplementary file 1 [file plants-15-02103-s001.zip › plants-4383327-supplementary/pepper_original_data/cone/120-14.jpg]

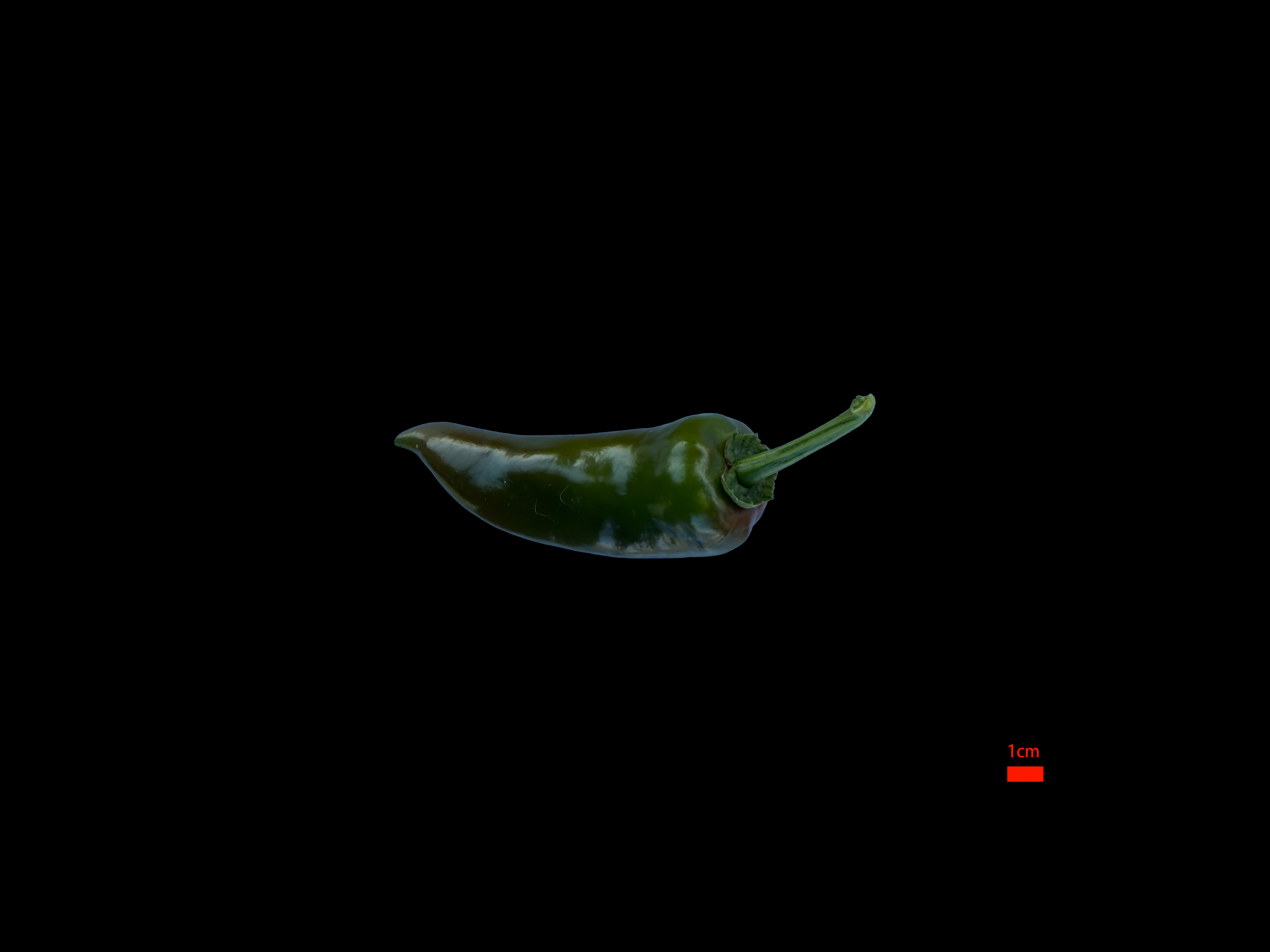

Supplement: Supplementary file 1 [file plants-15-02103-s001.zip › plants-4383327-supplementary/pepper_original_data/cone/120-15.jpg]

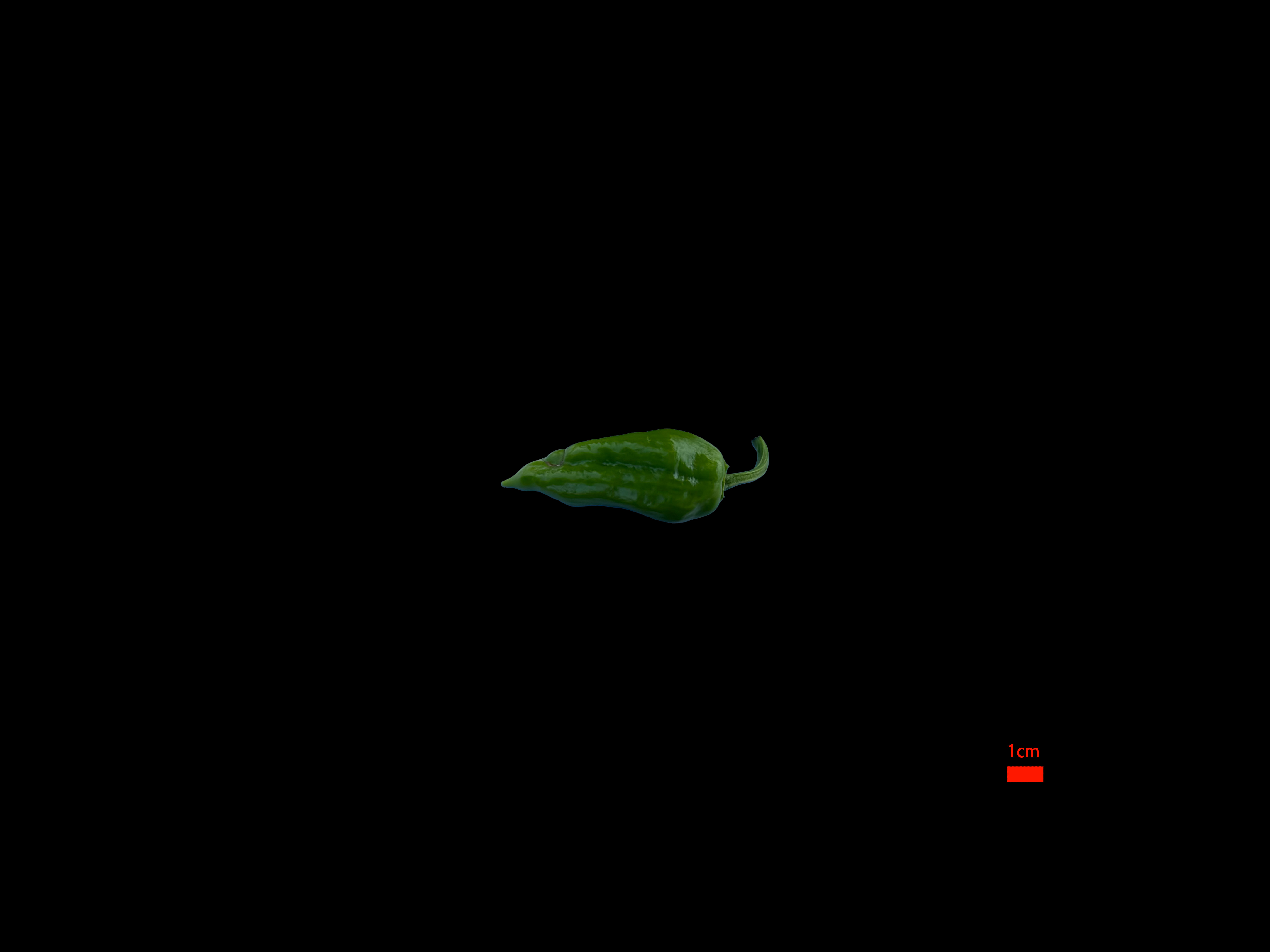

Supplement: Supplementary file 1 [file plants-15-02103-s001.zip › plants-4383327-supplementary/pepper_original_data/cone/120-2.jpg]

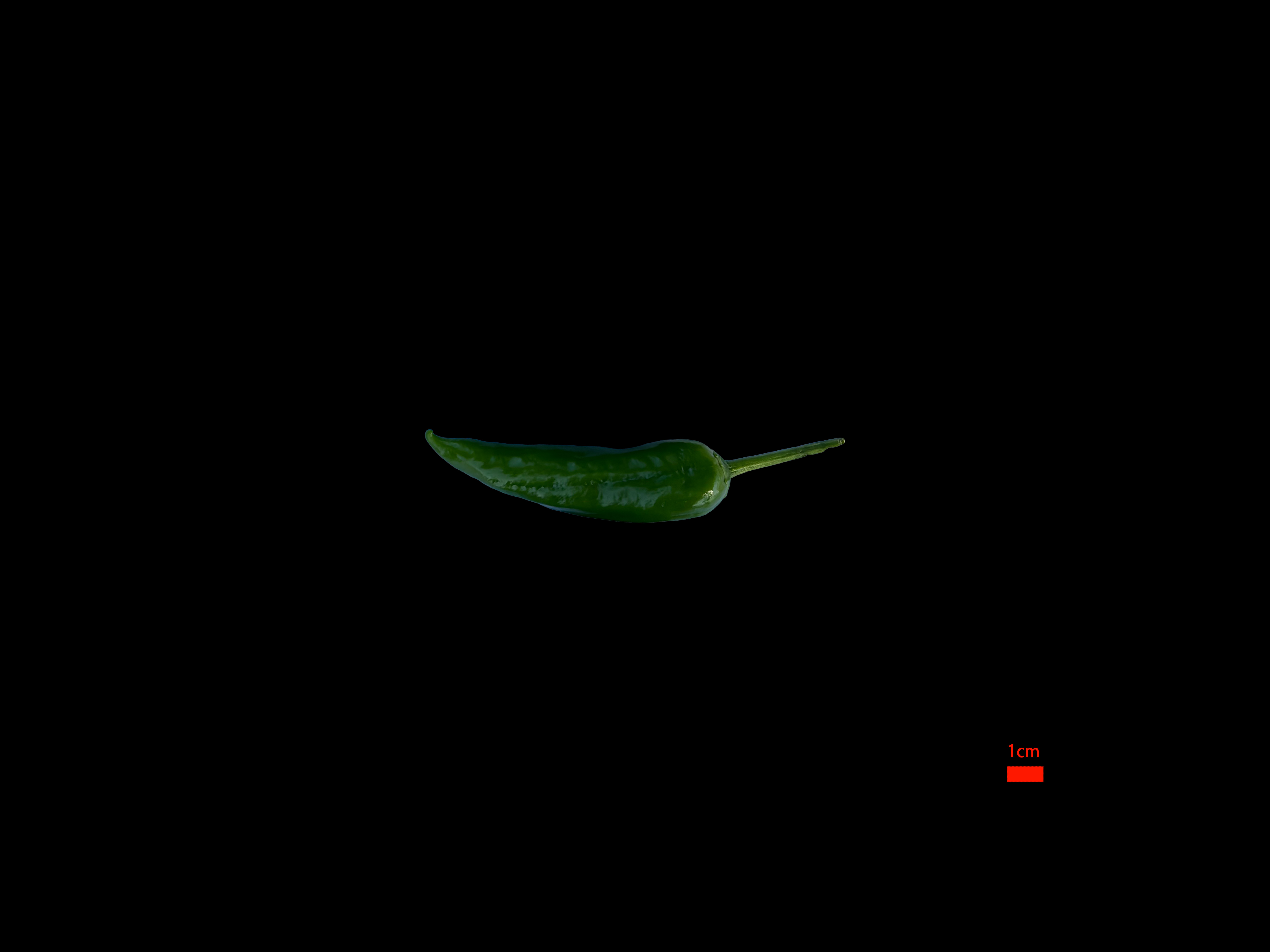

Supplement: Supplementary file 1 [file plants-15-02103-s001.zip › plants-4383327-supplementary/pepper_original_data/cone/120-3.jpg]

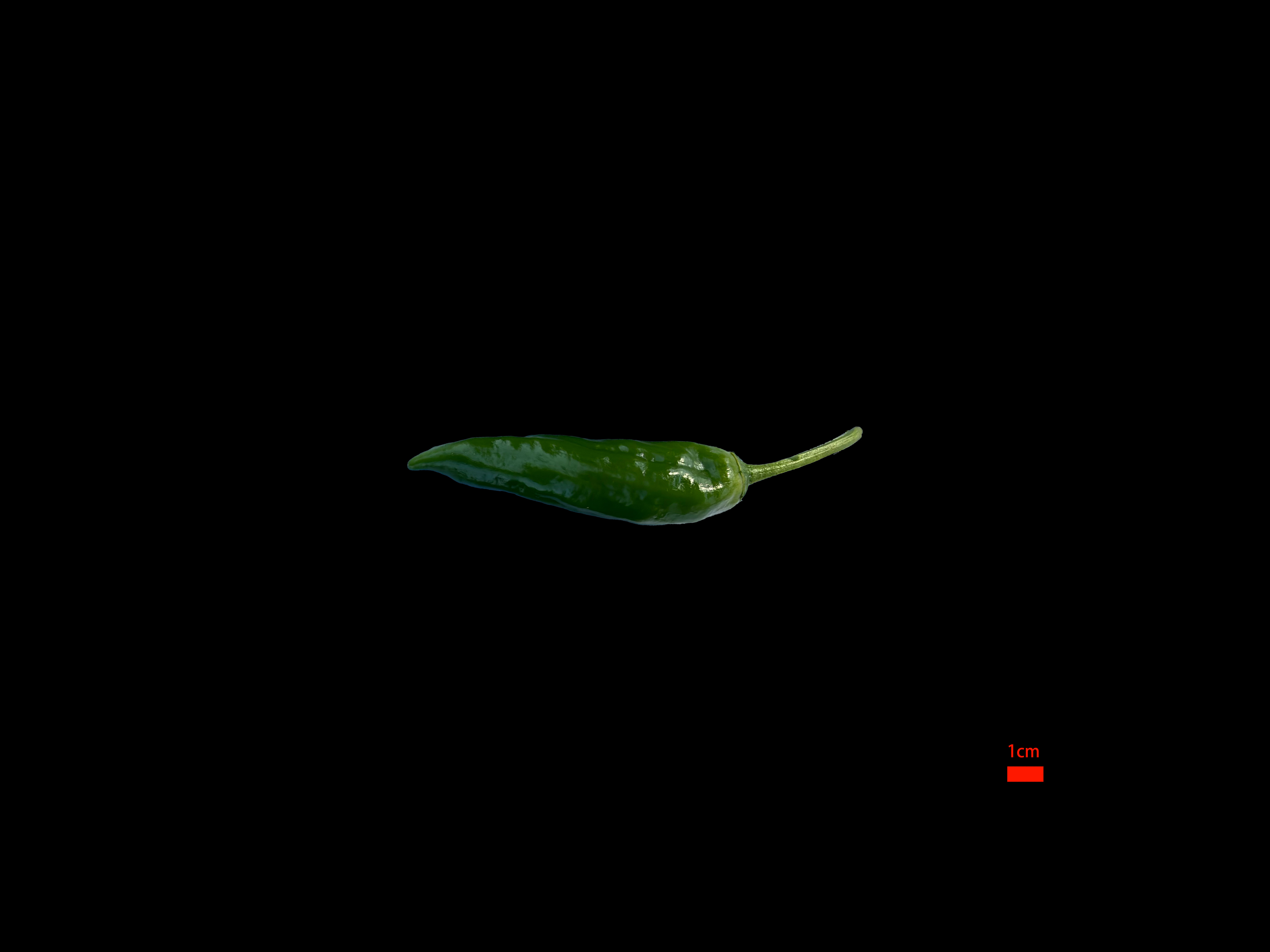

Supplement: Supplementary file 1 [file plants-15-02103-s001.zip › plants-4383327-supplementary/pepper_original_data/cone/120-4.jpg]

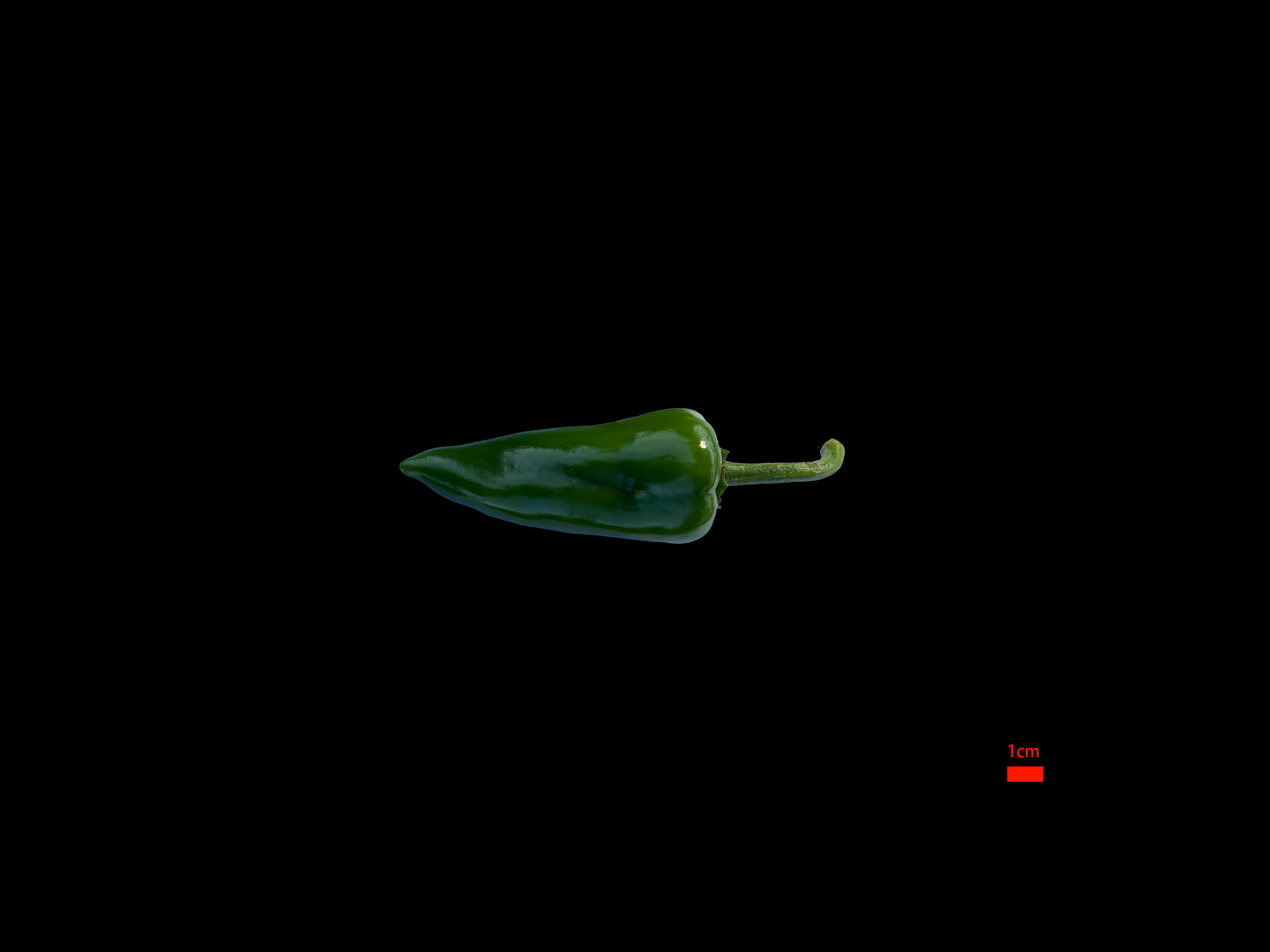

Supplement: Supplementary file 1 [file plants-15-02103-s001.zip › plants-4383327-supplementary/pepper_original_data/cone/120-5.jpg]

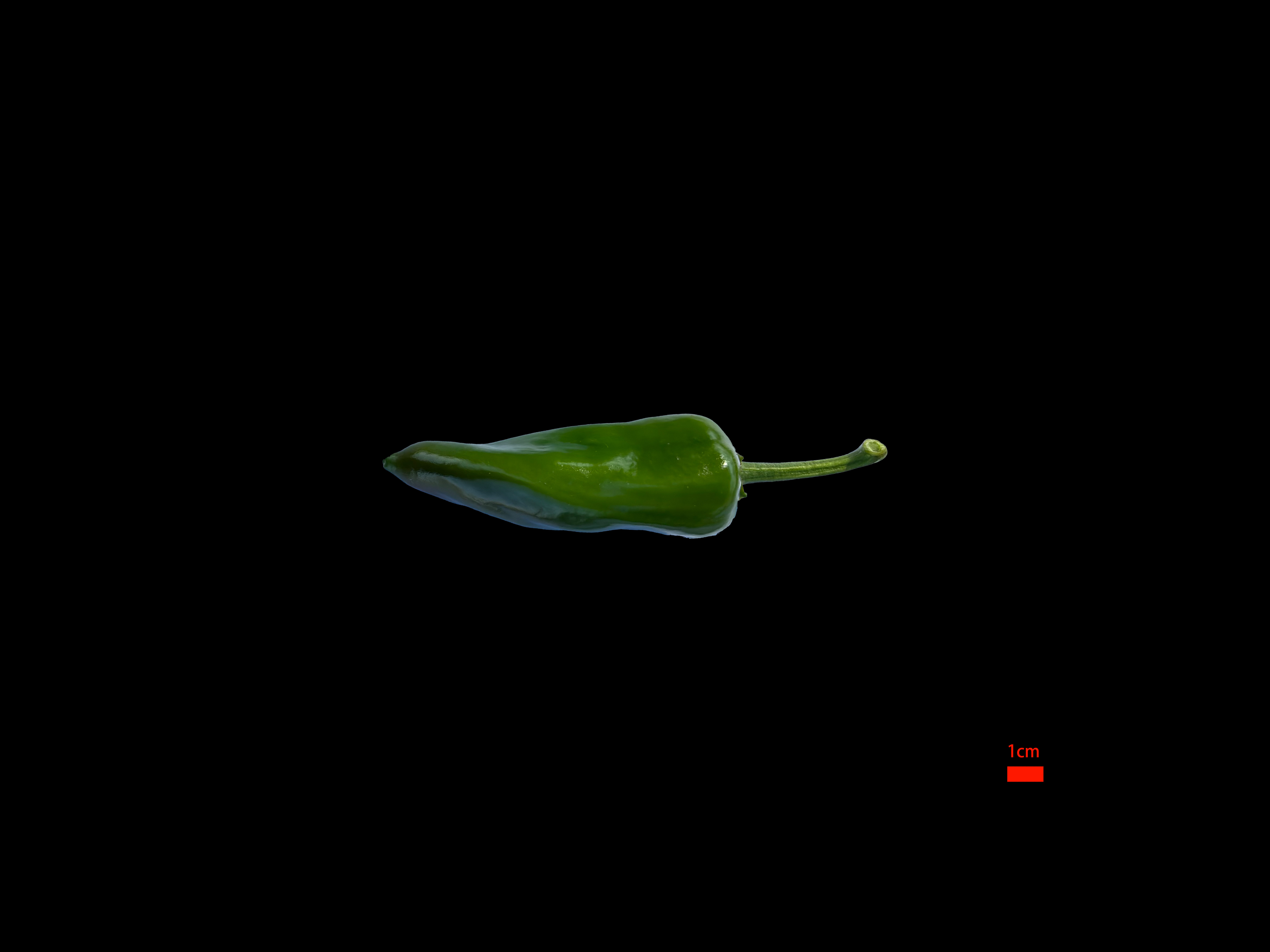

Supplement: Supplementary file 1 [file plants-15-02103-s001.zip › plants-4383327-supplementary/pepper_original_data/cone/120-6.jpg]

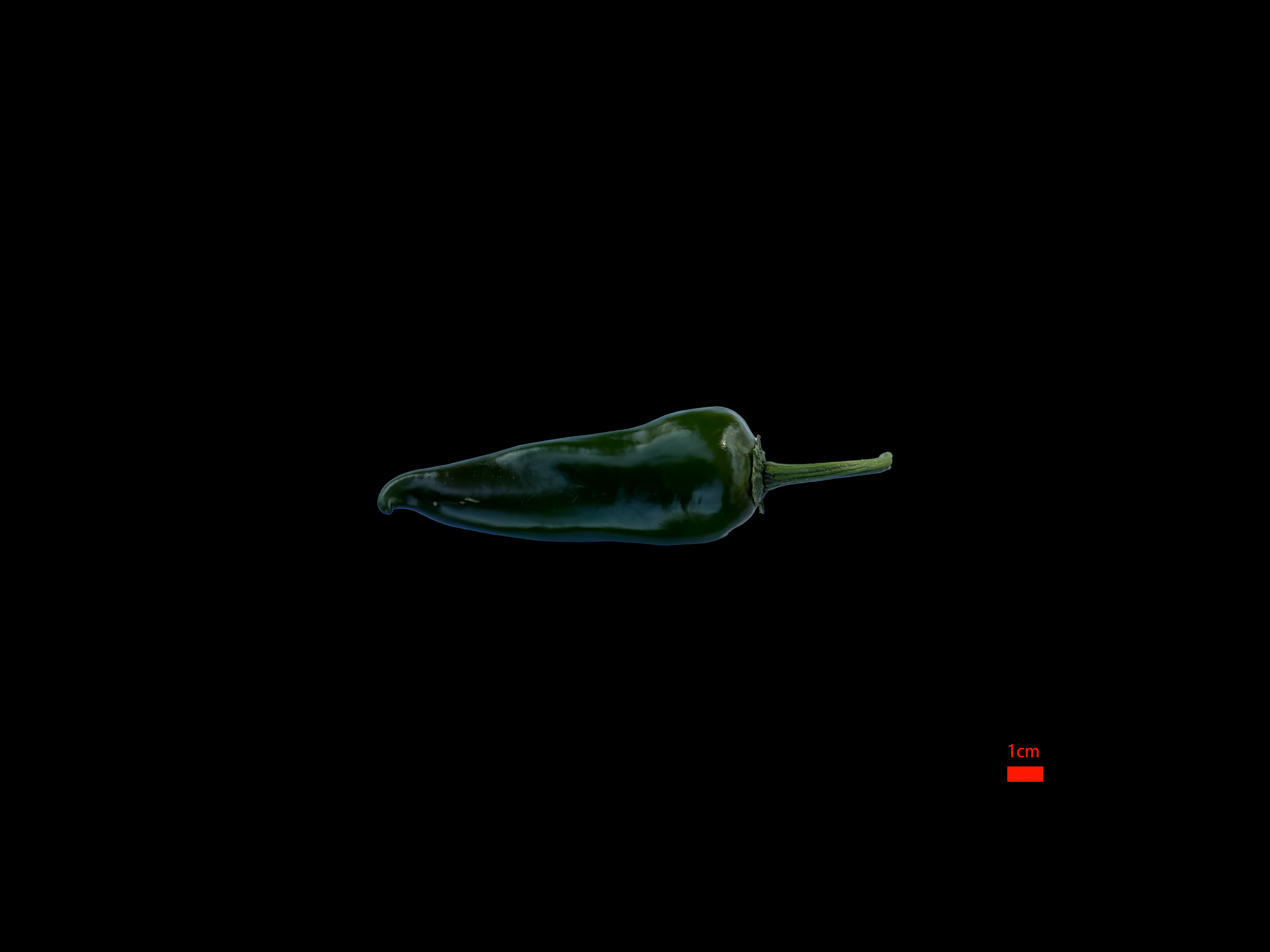

Supplement: Supplementary file 1 [file plants-15-02103-s001.zip › plants-4383327-supplementary/pepper_original_data/cone/120-7.jpg]

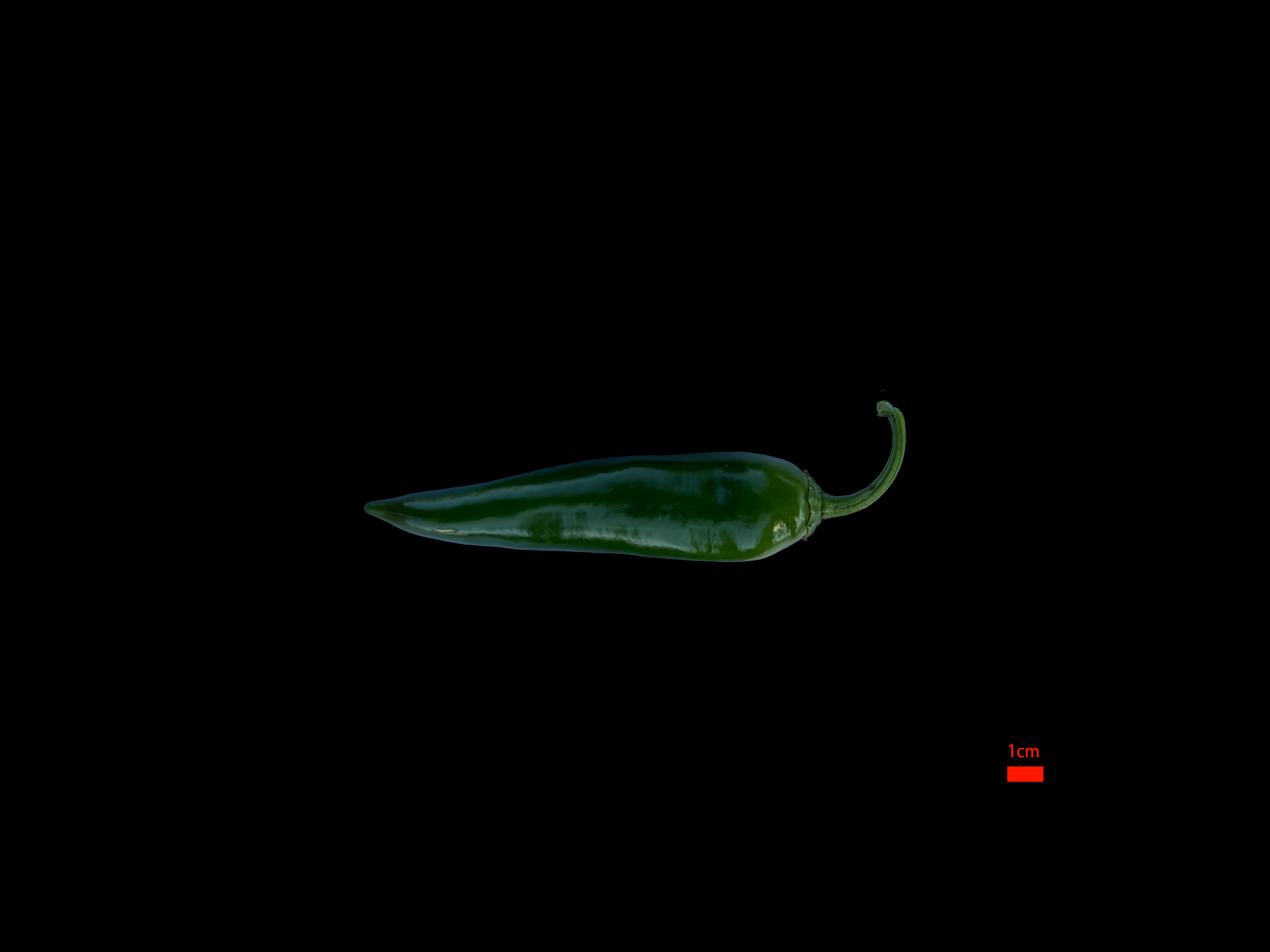

Supplement: Supplementary file 1 [file plants-15-02103-s001.zip › plants-4383327-supplementary/pepper_original_data/cone/120-8.jpg]

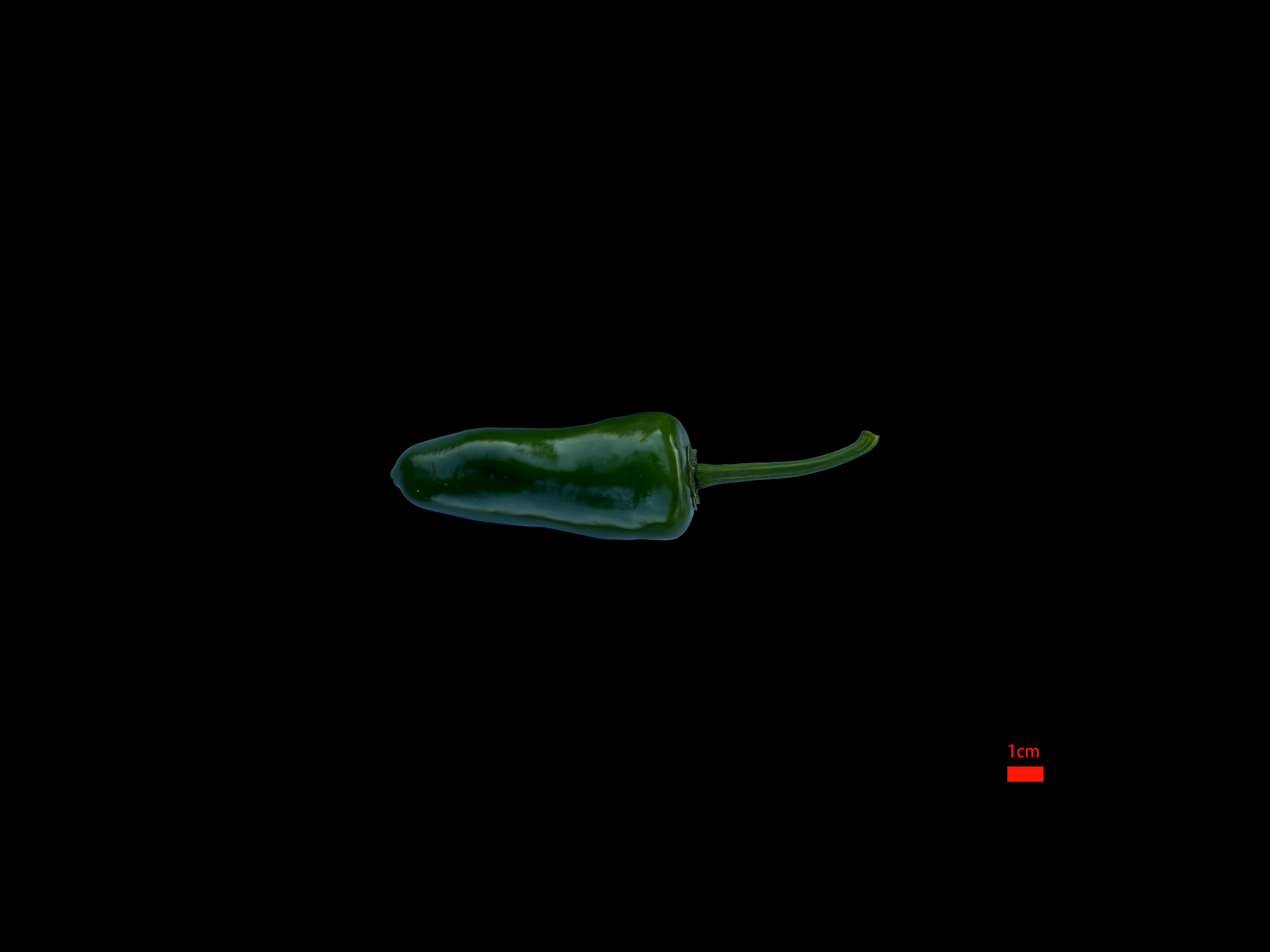

Supplement: Supplementary file 1 [file plants-15-02103-s001.zip › plants-4383327-supplementary/pepper_original_data/cone/120-9.jpg]

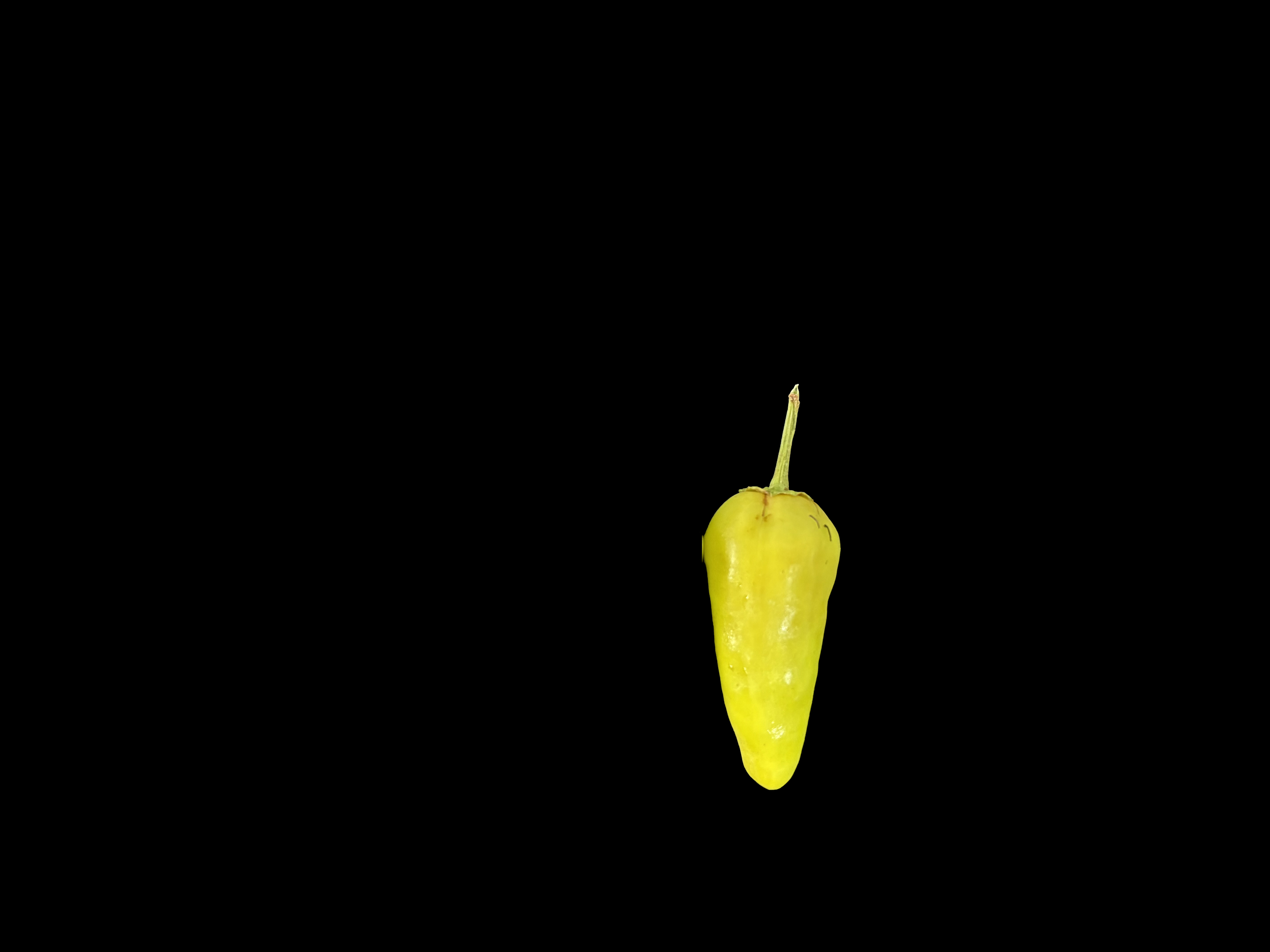

Supplement: Supplementary file 1 [file plants-15-02103-s001.zip › plants-4383327-supplementary/pepper_original_data/cone/121.1.jpg]

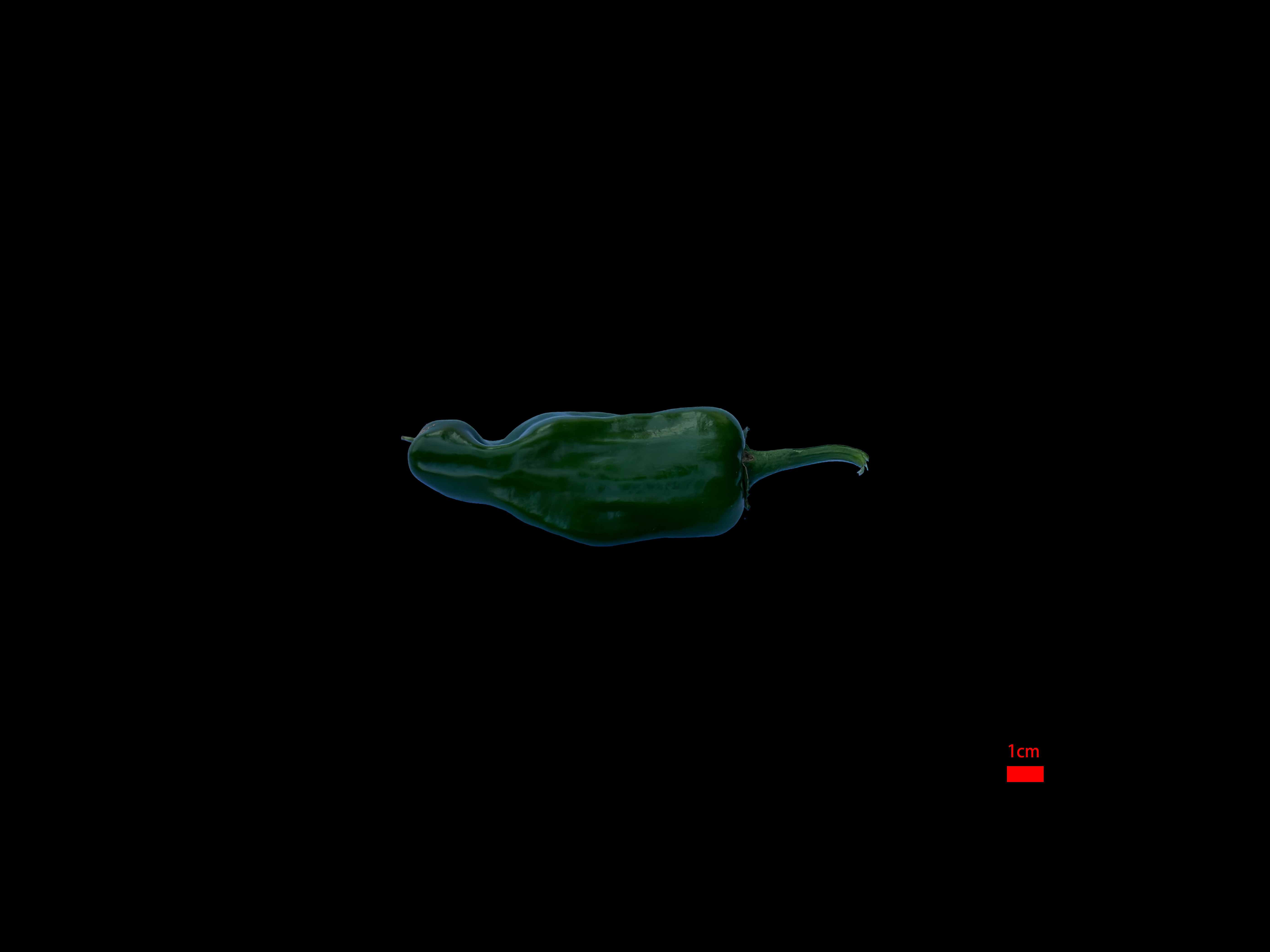

Supplement: Supplementary file 1 [file plants-15-02103-s001.zip › plants-4383327-supplementary/pepper_original_data/cone/138-1.jpg]

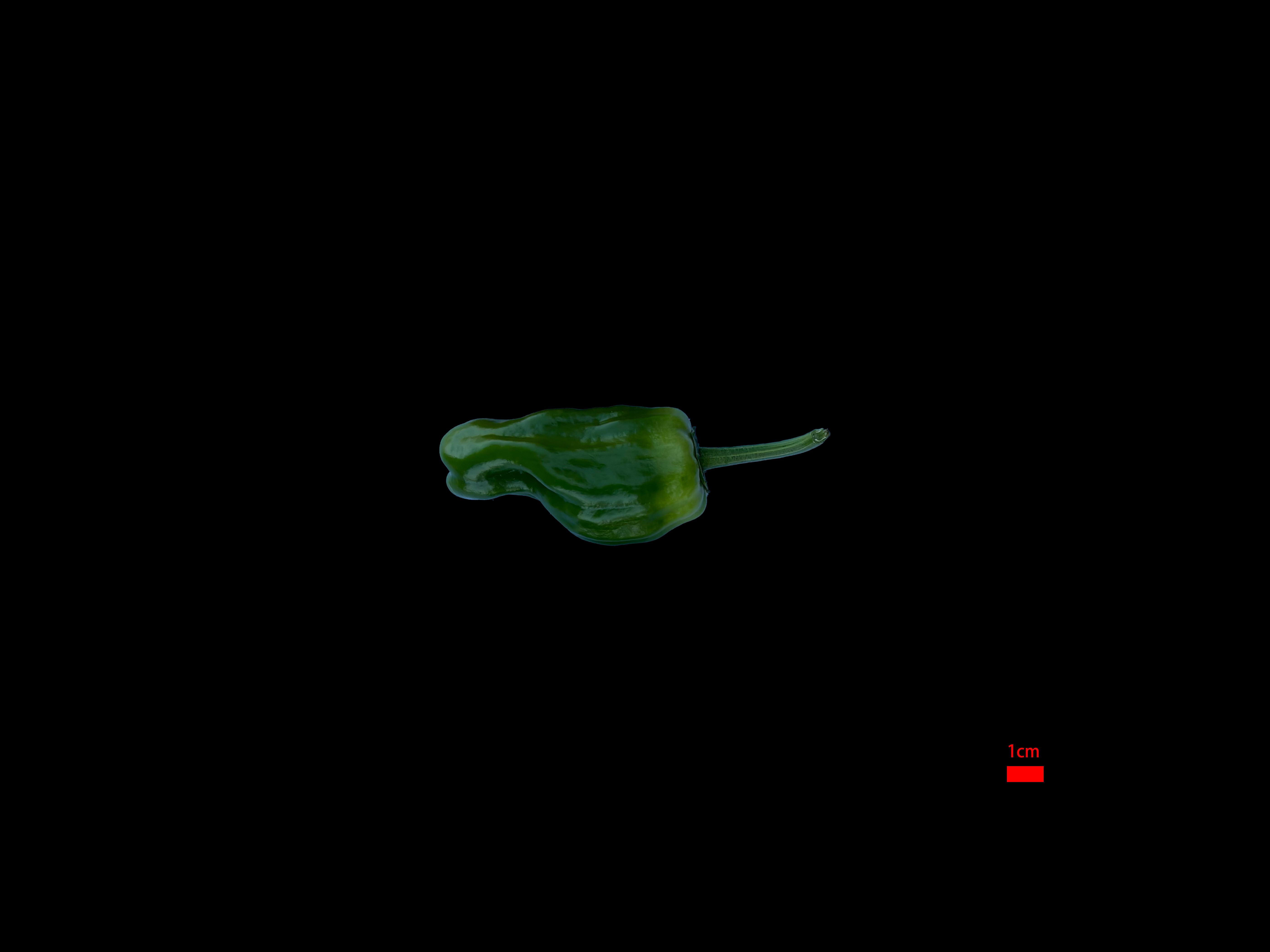

Supplement: Supplementary file 1 [file plants-15-02103-s001.zip › plants-4383327-supplementary/pepper_original_data/cone/138-10.jpg]

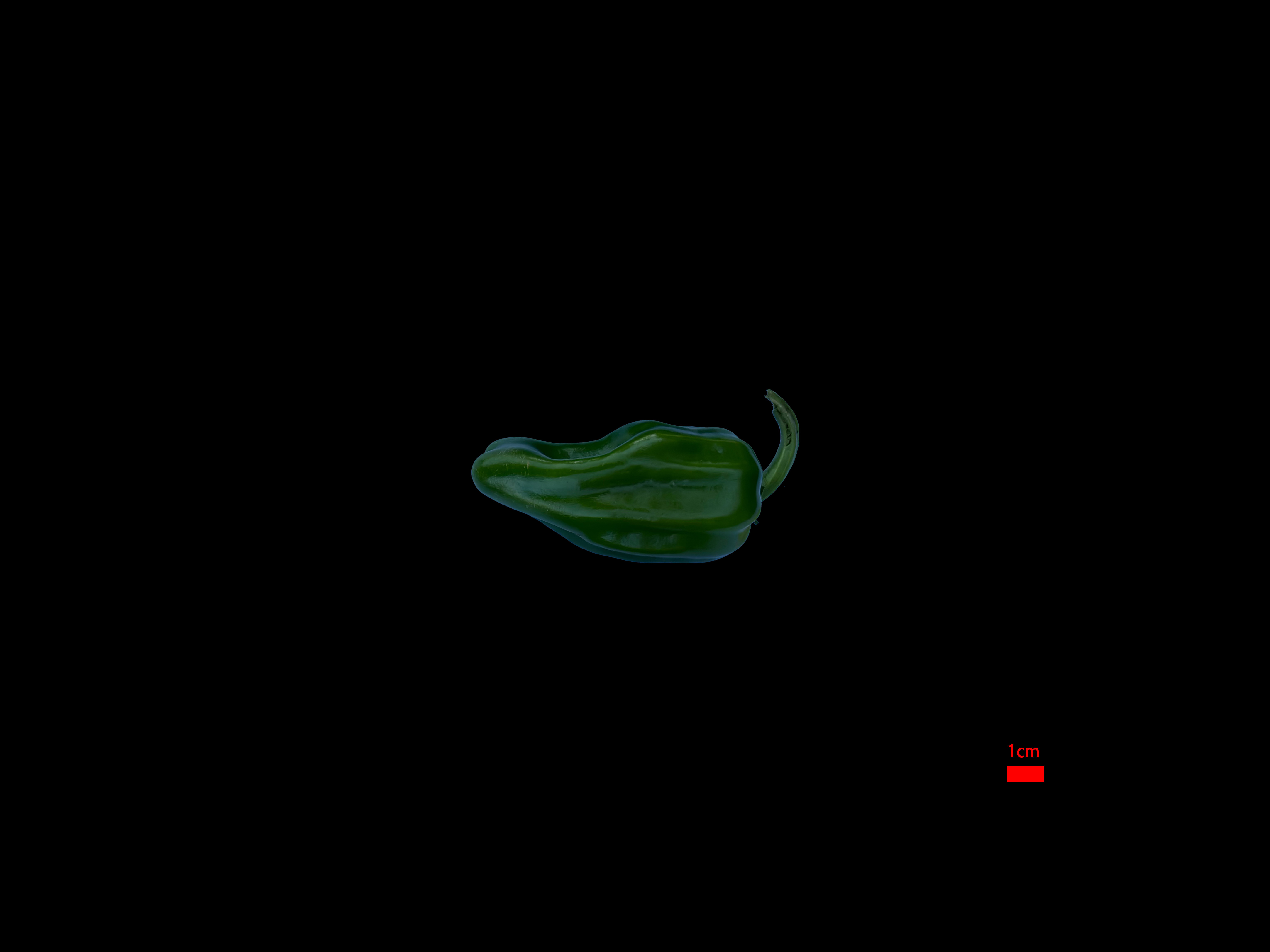

Supplement: Supplementary file 1 [file plants-15-02103-s001.zip › plants-4383327-supplementary/pepper_original_data/cone/138-2.jpg]

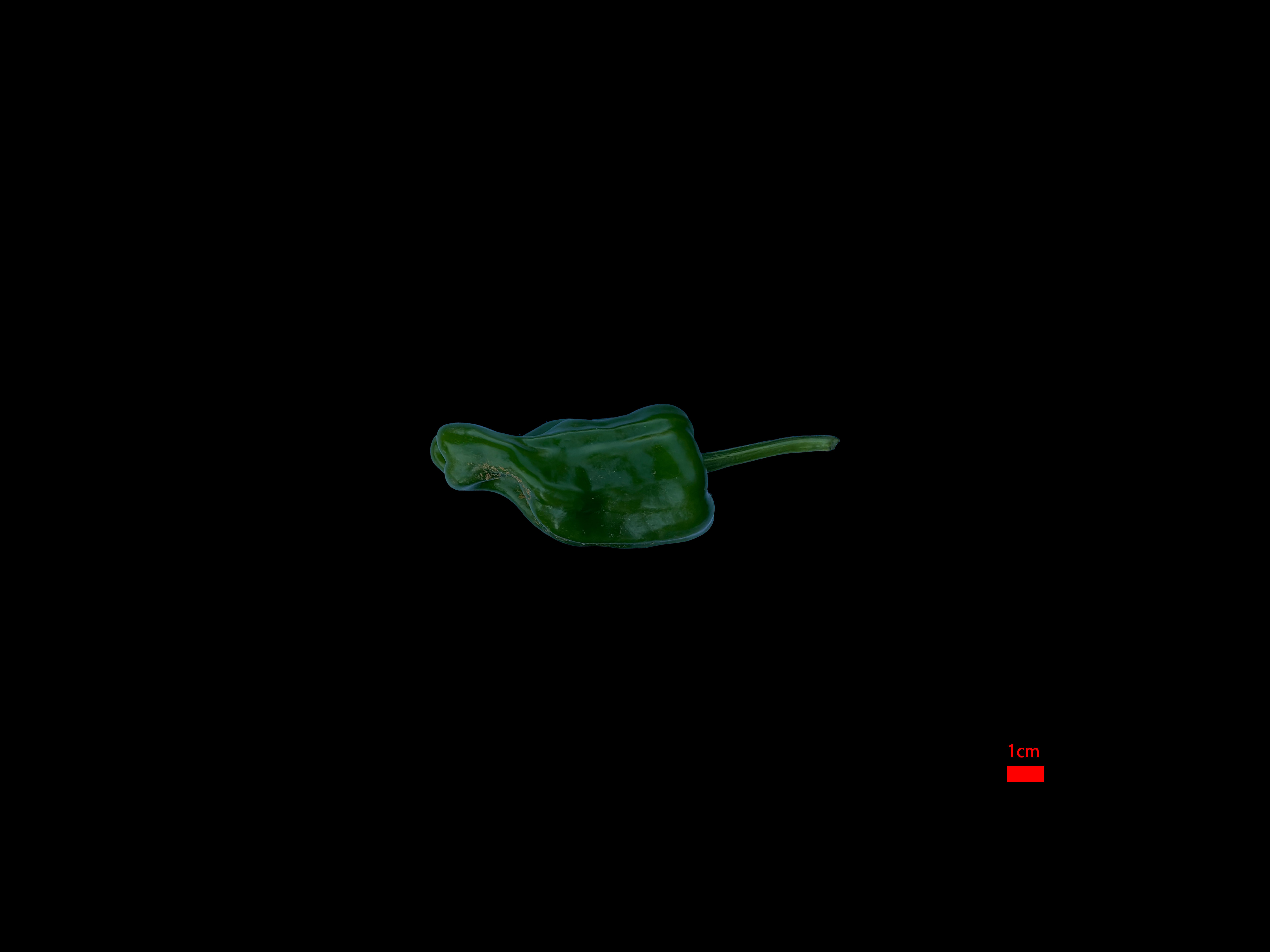

Supplement: Supplementary file 1 [file plants-15-02103-s001.zip › plants-4383327-supplementary/pepper_original_data/cone/138-3.jpg]

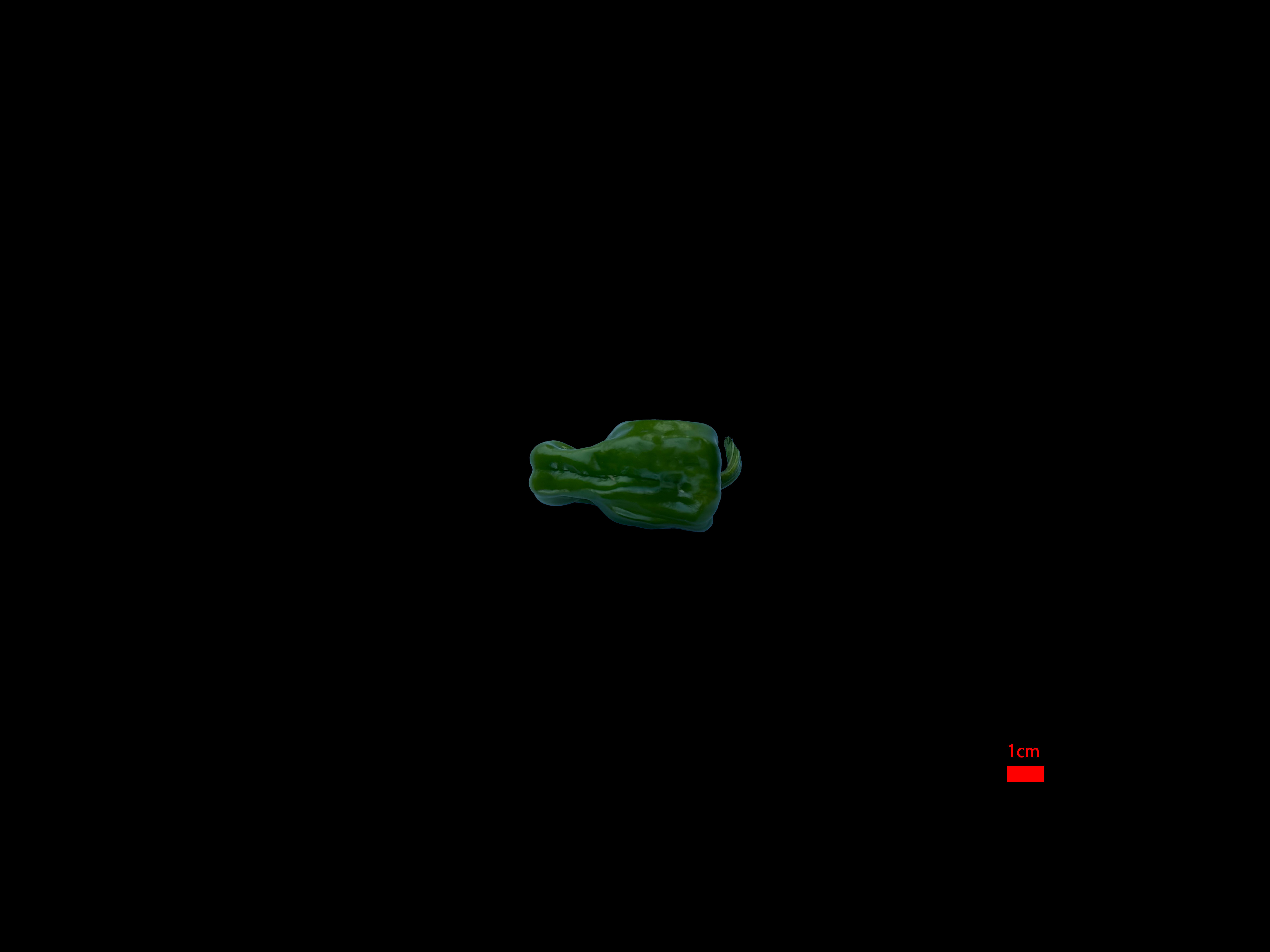

Supplement: Supplementary file 1 [file plants-15-02103-s001.zip › plants-4383327-supplementary/pepper_original_data/cone/138-7.jpg]

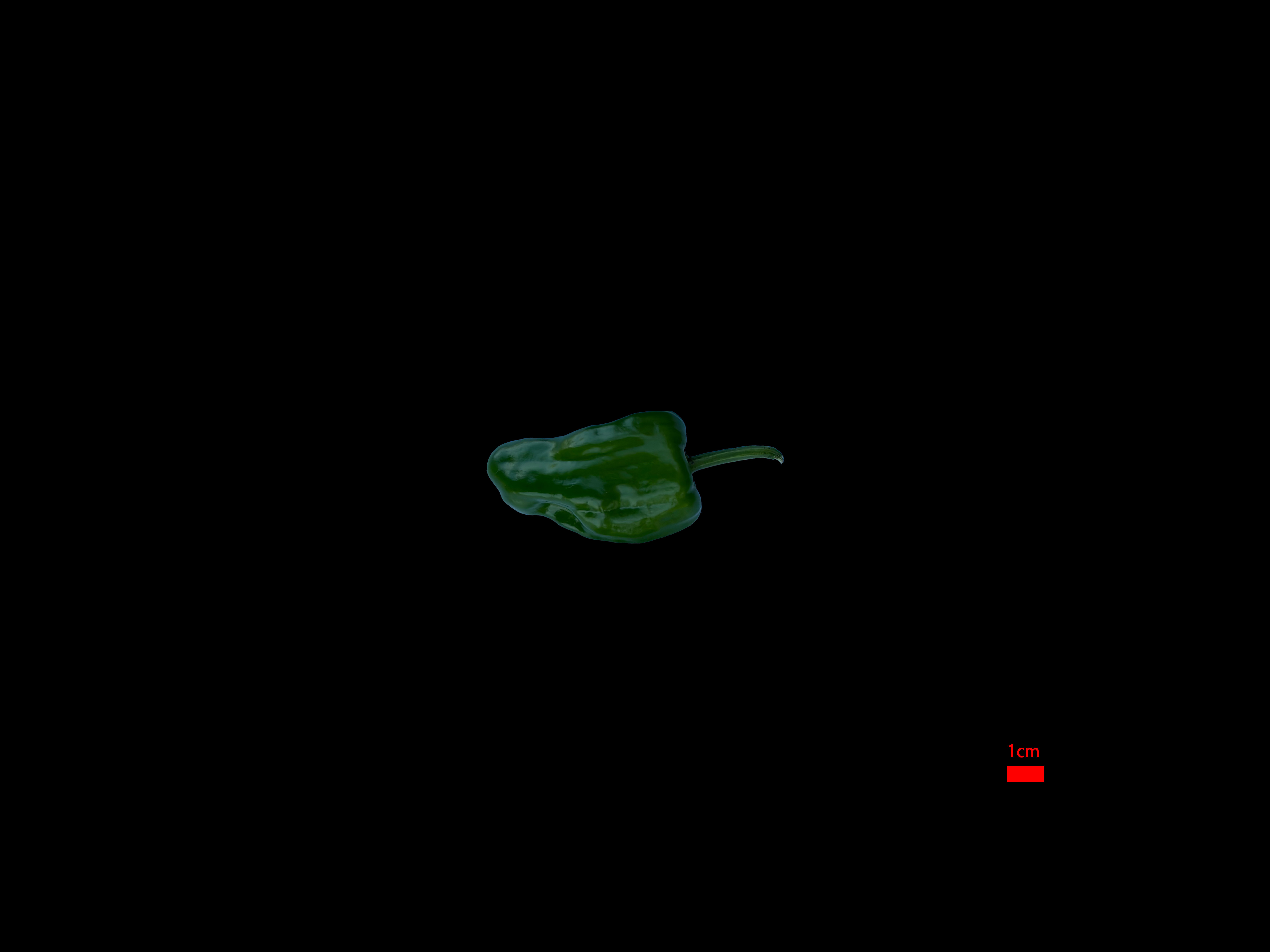

Supplement: Supplementary file 1 [file plants-15-02103-s001.zip › plants-4383327-supplementary/pepper_original_data/cone/138-9.jpg]

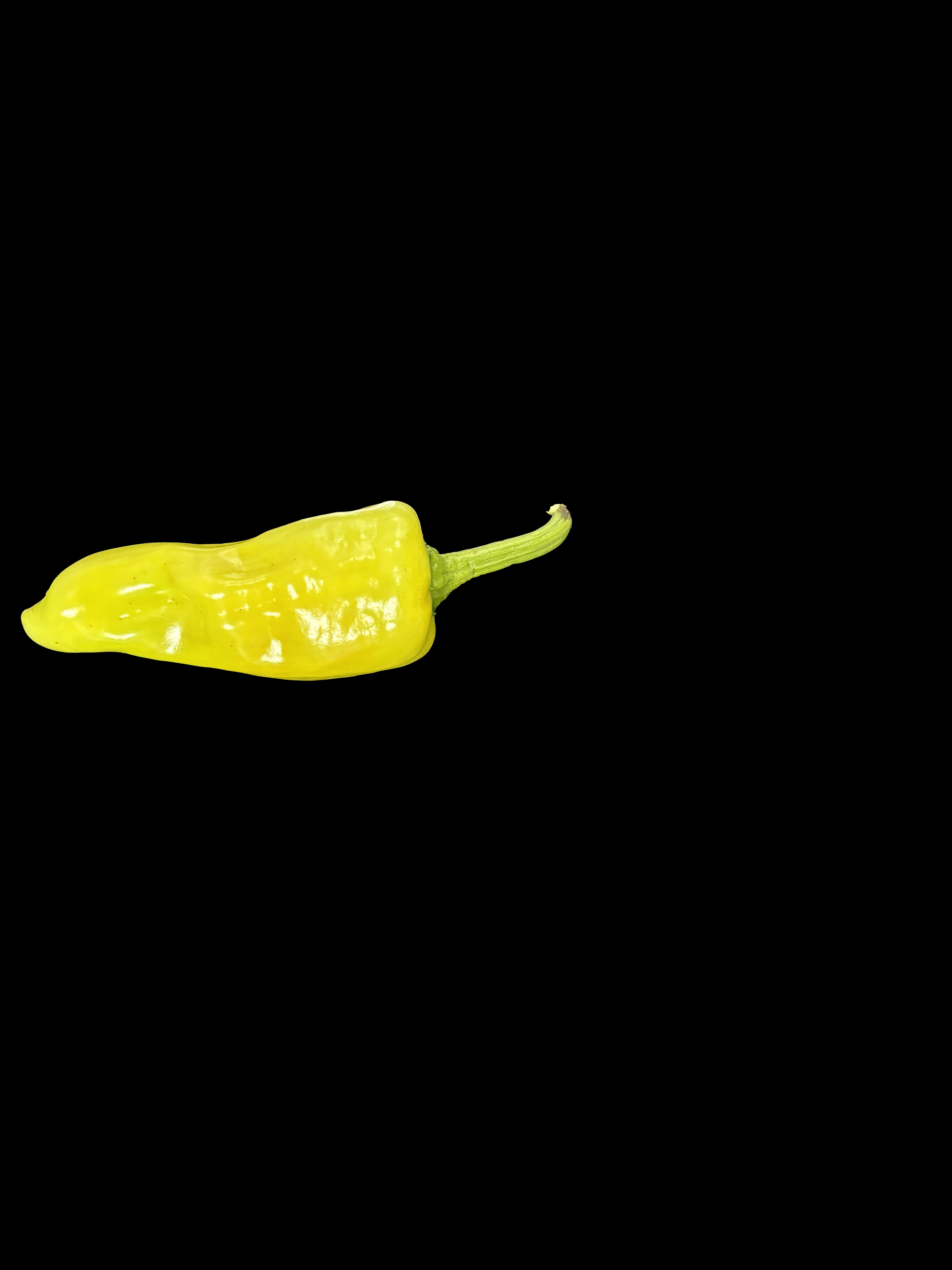

Supplement: Supplementary file 1 [file plants-15-02103-s001.zip › plants-4383327-supplementary/pepper_original_data/cone/154.jpg]

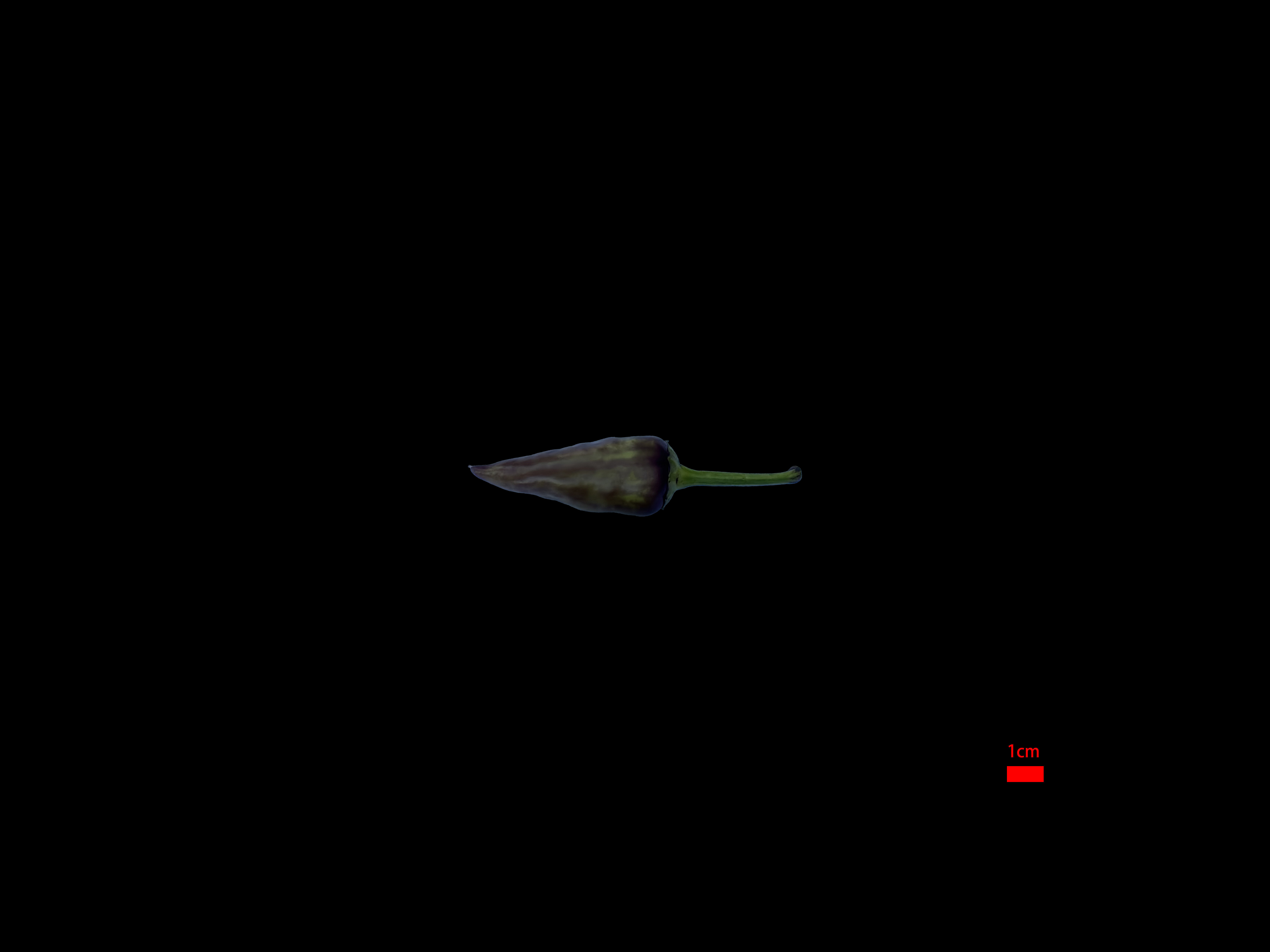

Supplement: Supplementary file 1 [file plants-15-02103-s001.zip › plants-4383327-supplementary/pepper_original_data/cone/16-1.jpg]

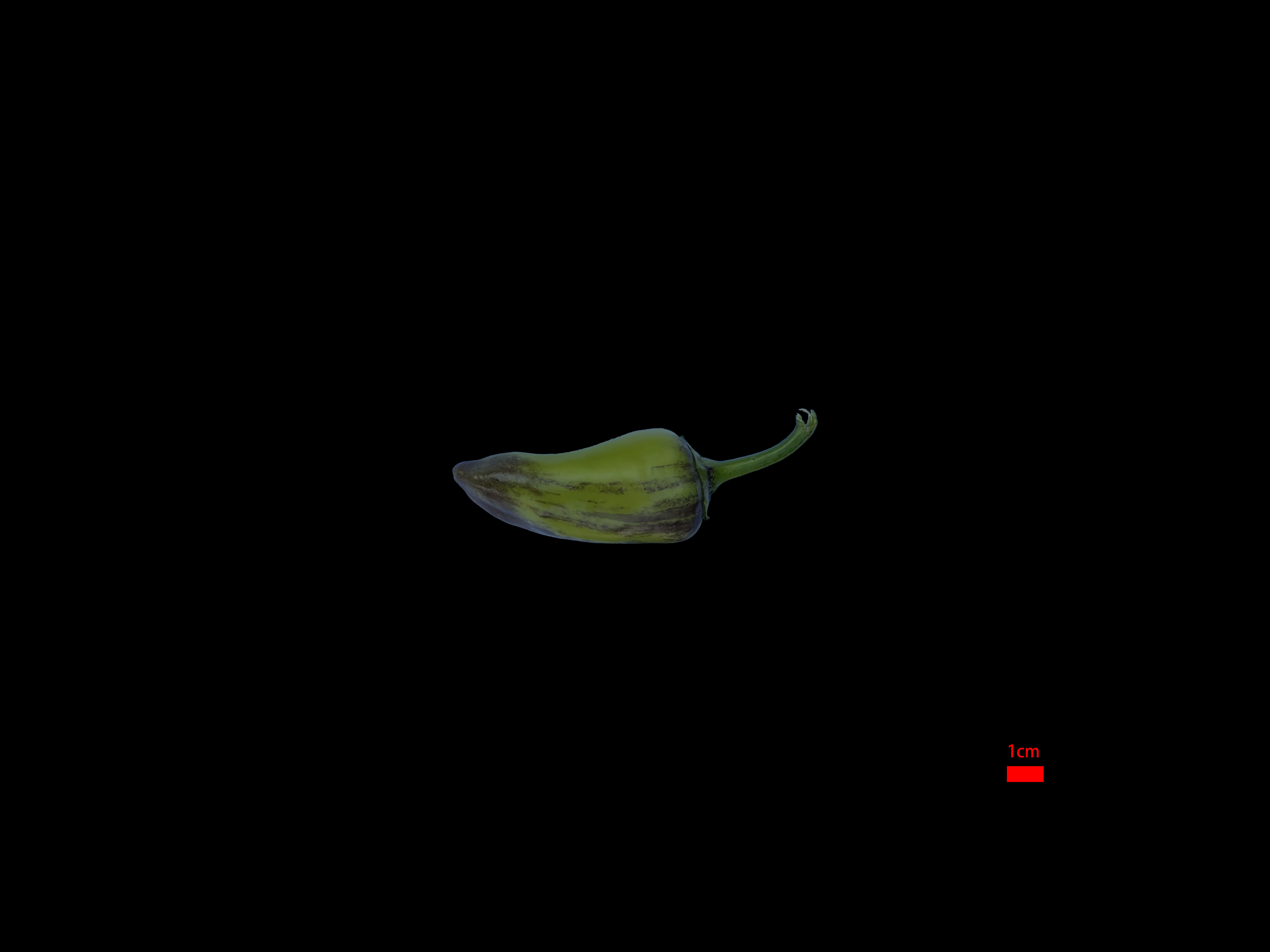

Supplement: Supplementary file 1 [file plants-15-02103-s001.zip › plants-4383327-supplementary/pepper_original_data/cone/16-2.jpg]

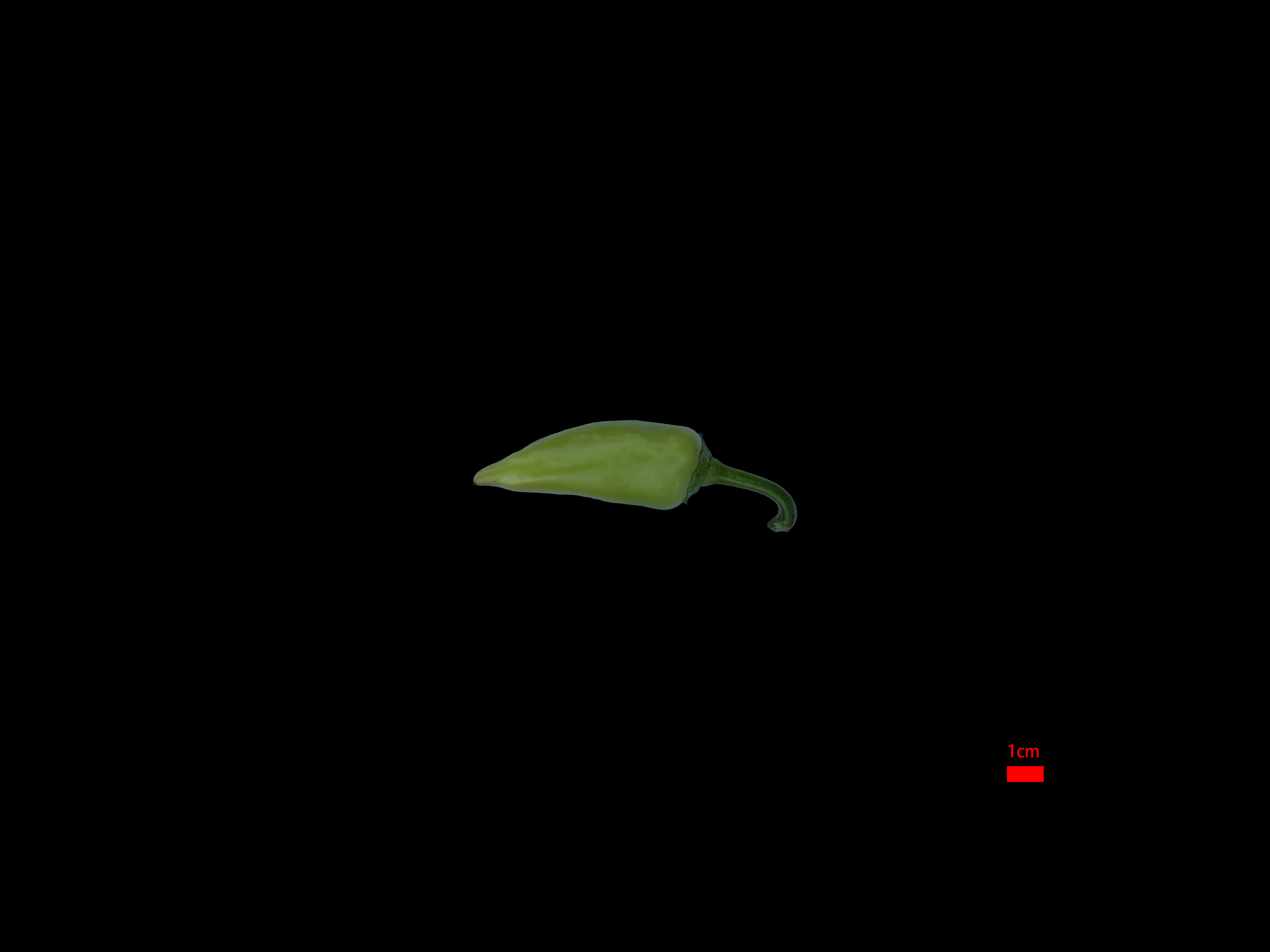

Supplement: Supplementary file 1 [file plants-15-02103-s001.zip › plants-4383327-supplementary/pepper_original_data/cone/16-3.jpg]

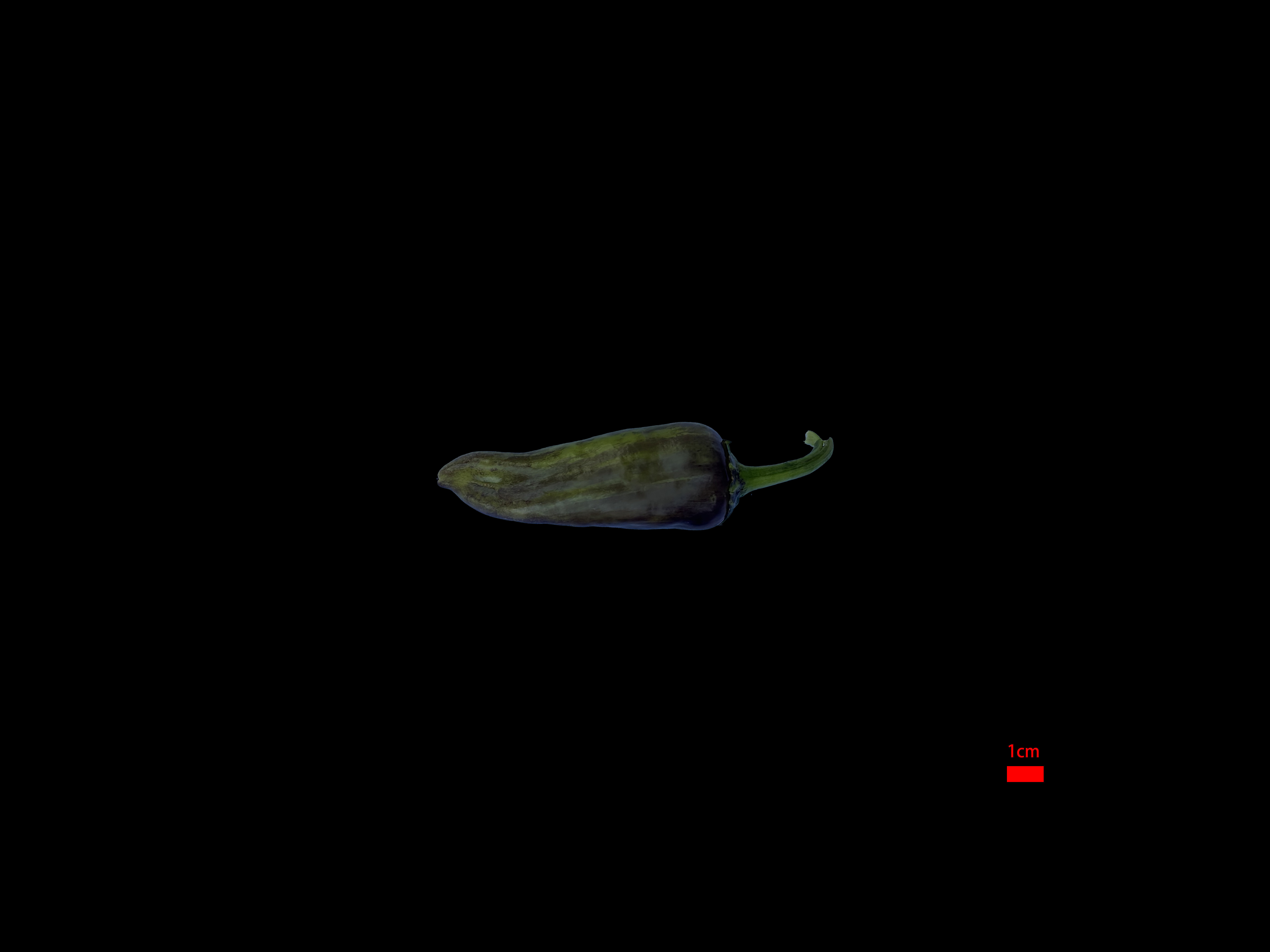

Supplement: Supplementary file 1 [file plants-15-02103-s001.zip › plants-4383327-supplementary/pepper_original_data/cone/16-4.jpg]

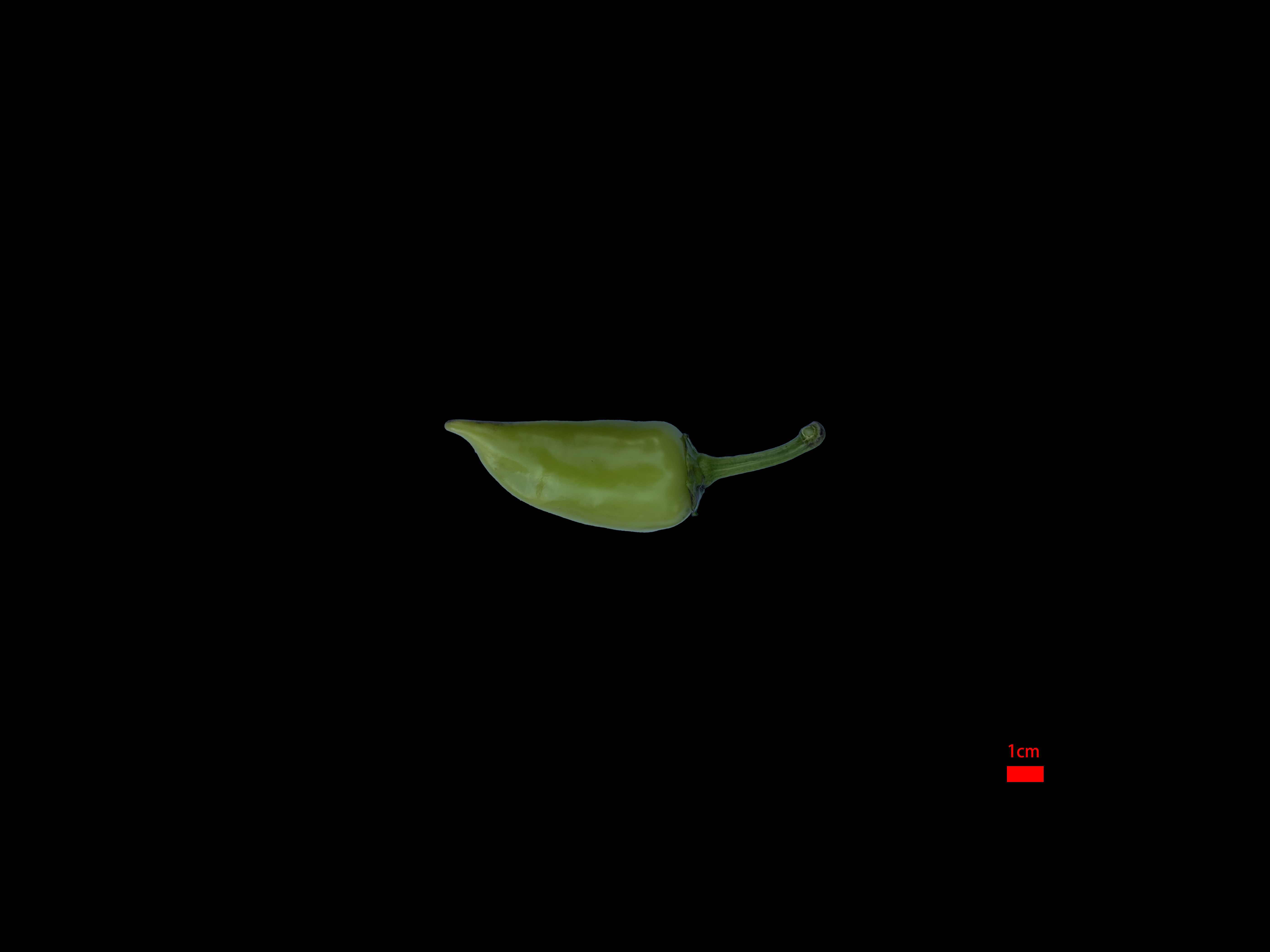

Supplement: Supplementary file 1 [file plants-15-02103-s001.zip › plants-4383327-supplementary/pepper_original_data/cone/16-5.jpg]

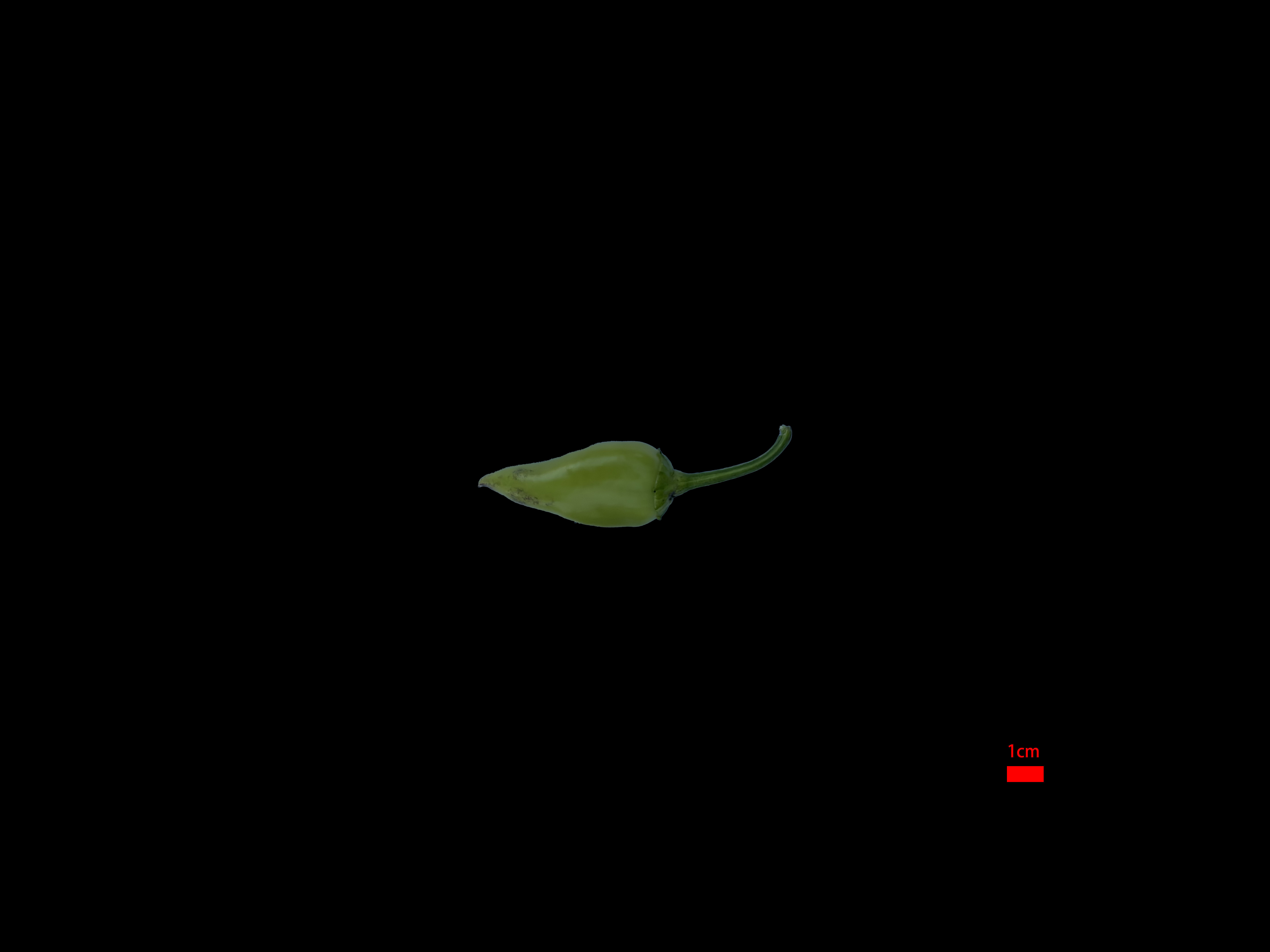

Supplement: Supplementary file 1 [file plants-15-02103-s001.zip › plants-4383327-supplementary/pepper_original_data/cone/16-7.jpg]

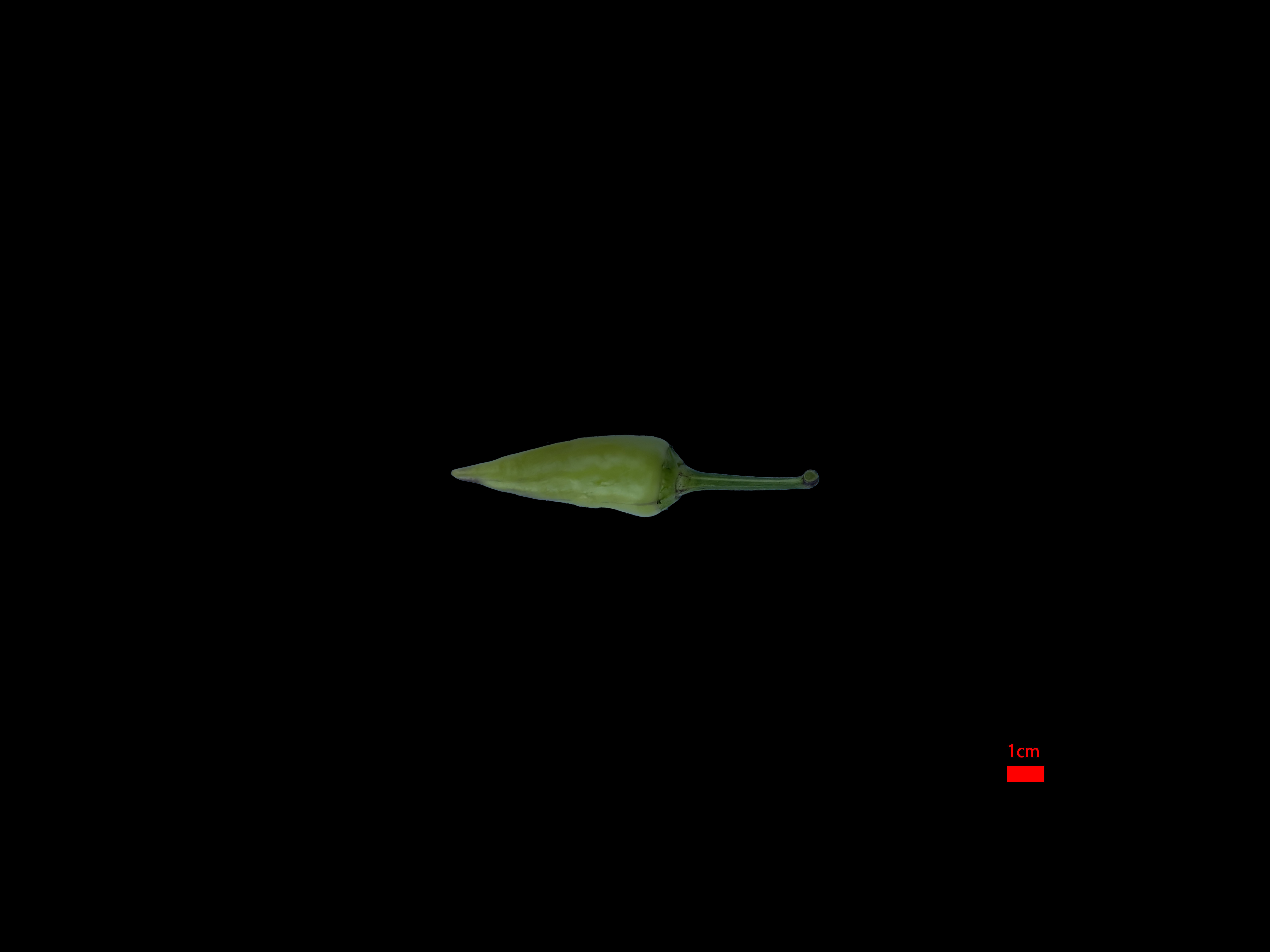

Supplement: Supplementary file 1 [file plants-15-02103-s001.zip › plants-4383327-supplementary/pepper_original_data/cone/16-9.jpg]

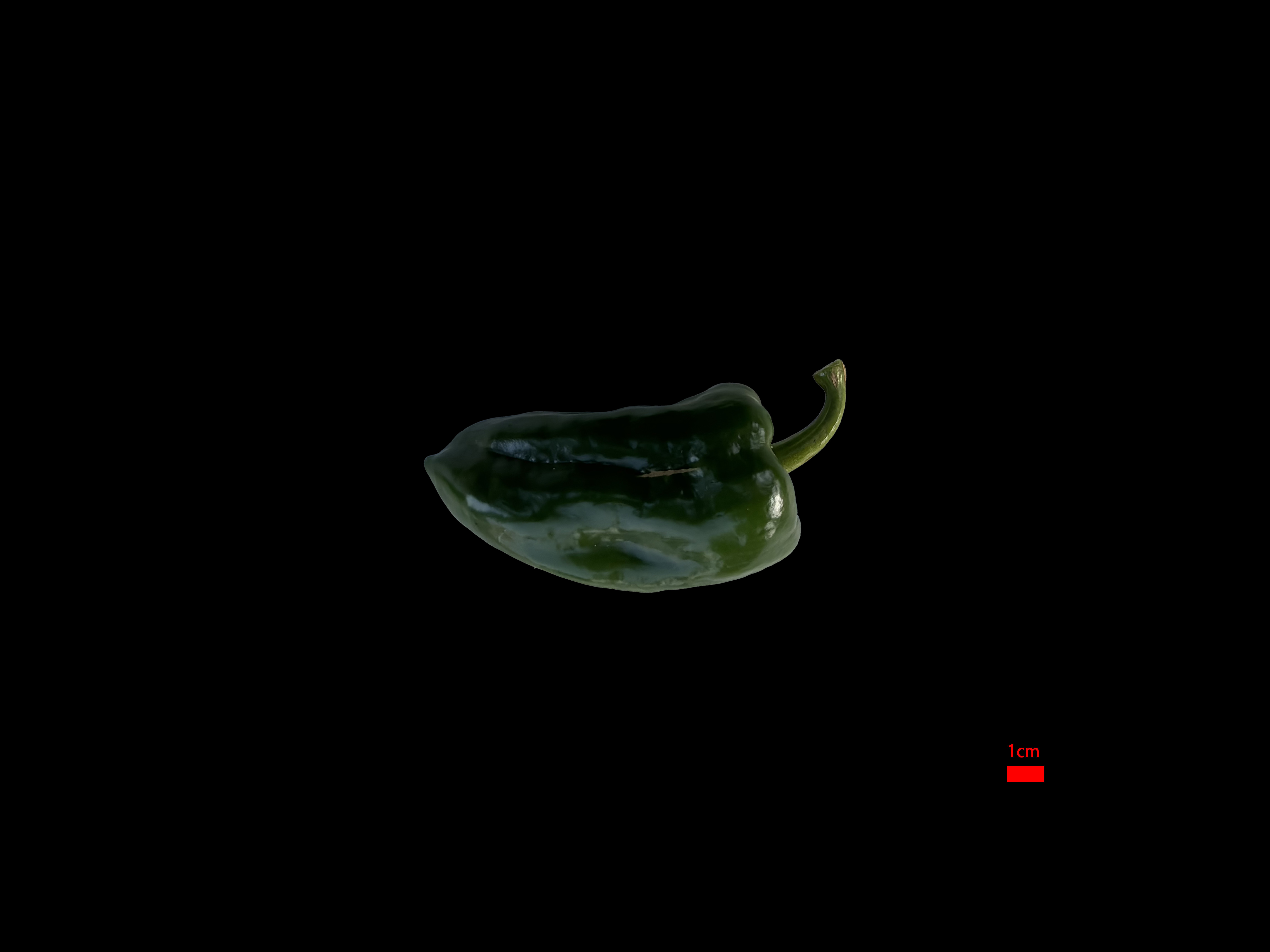

Supplement: Supplementary file 1 [file plants-15-02103-s001.zip › plants-4383327-supplementary/pepper_original_data/cone/169-1.jpg]

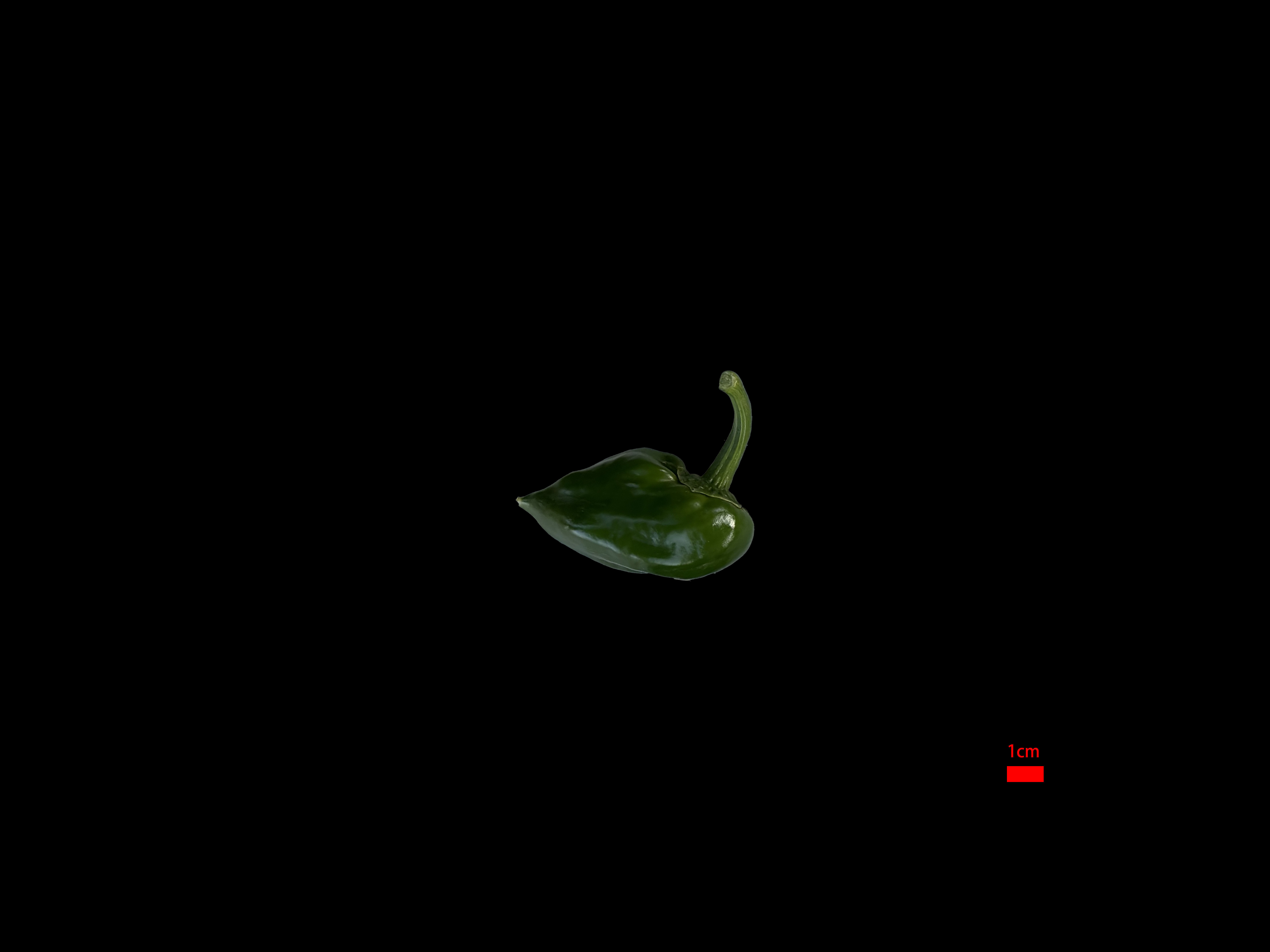

Supplement: Supplementary file 1 [file plants-15-02103-s001.zip › plants-4383327-supplementary/pepper_original_data/cone/169-10.jpg]

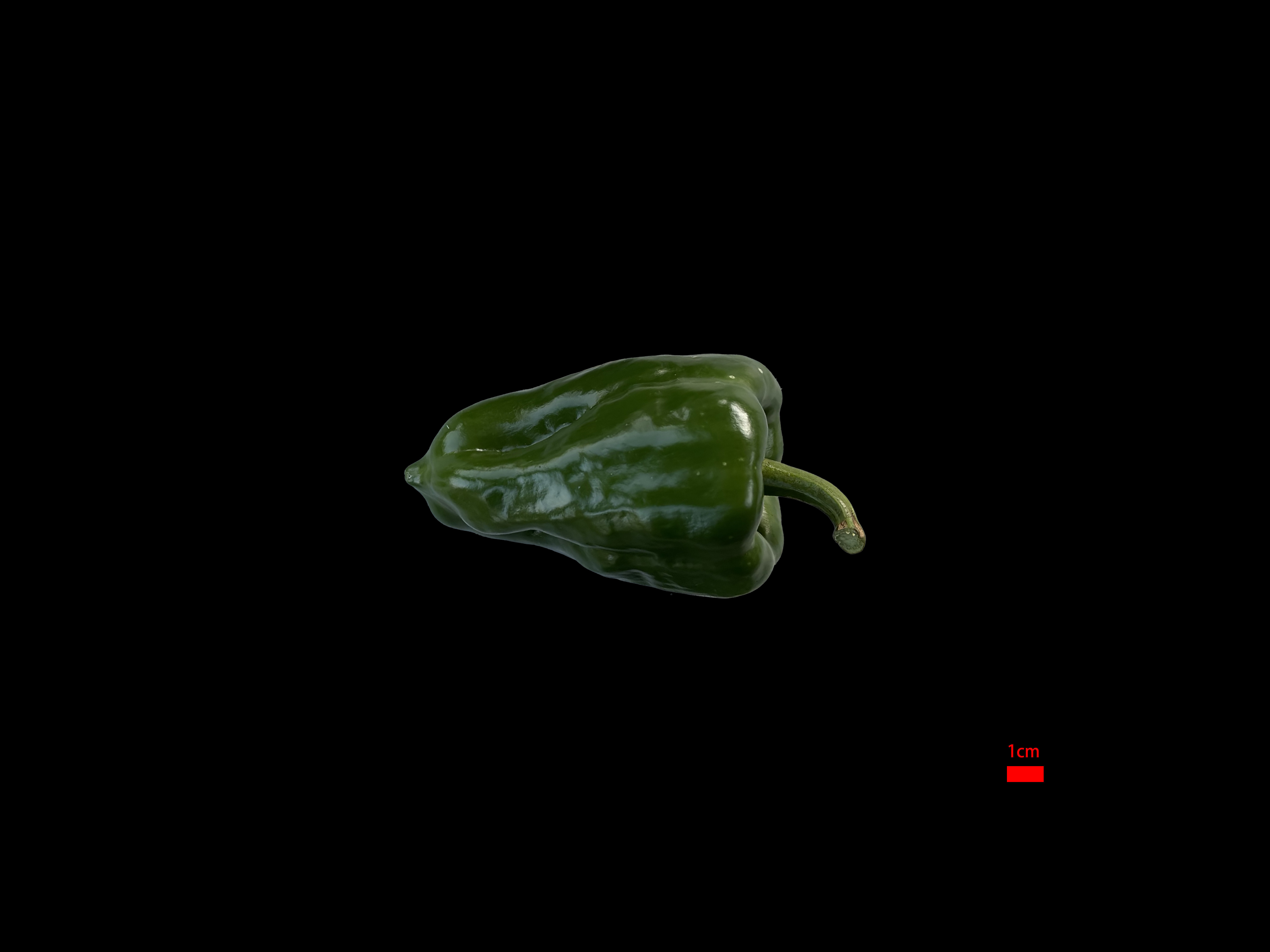

Supplement: Supplementary file 1 [file plants-15-02103-s001.zip › plants-4383327-supplementary/pepper_original_data/cone/169-4.jpg]

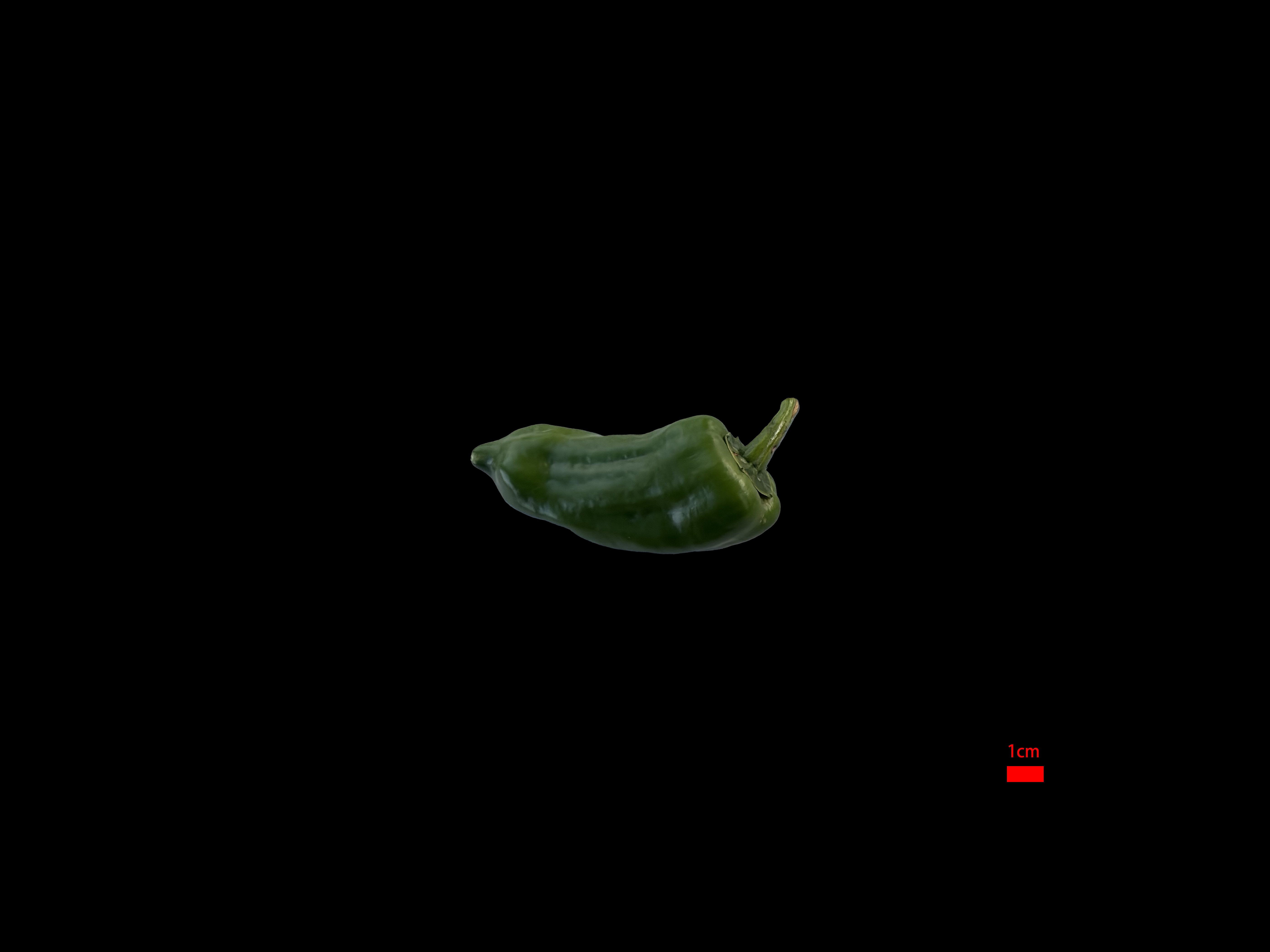

Supplement: Supplementary file 1 [file plants-15-02103-s001.zip › plants-4383327-supplementary/pepper_original_data/cone/169-6.jpg]

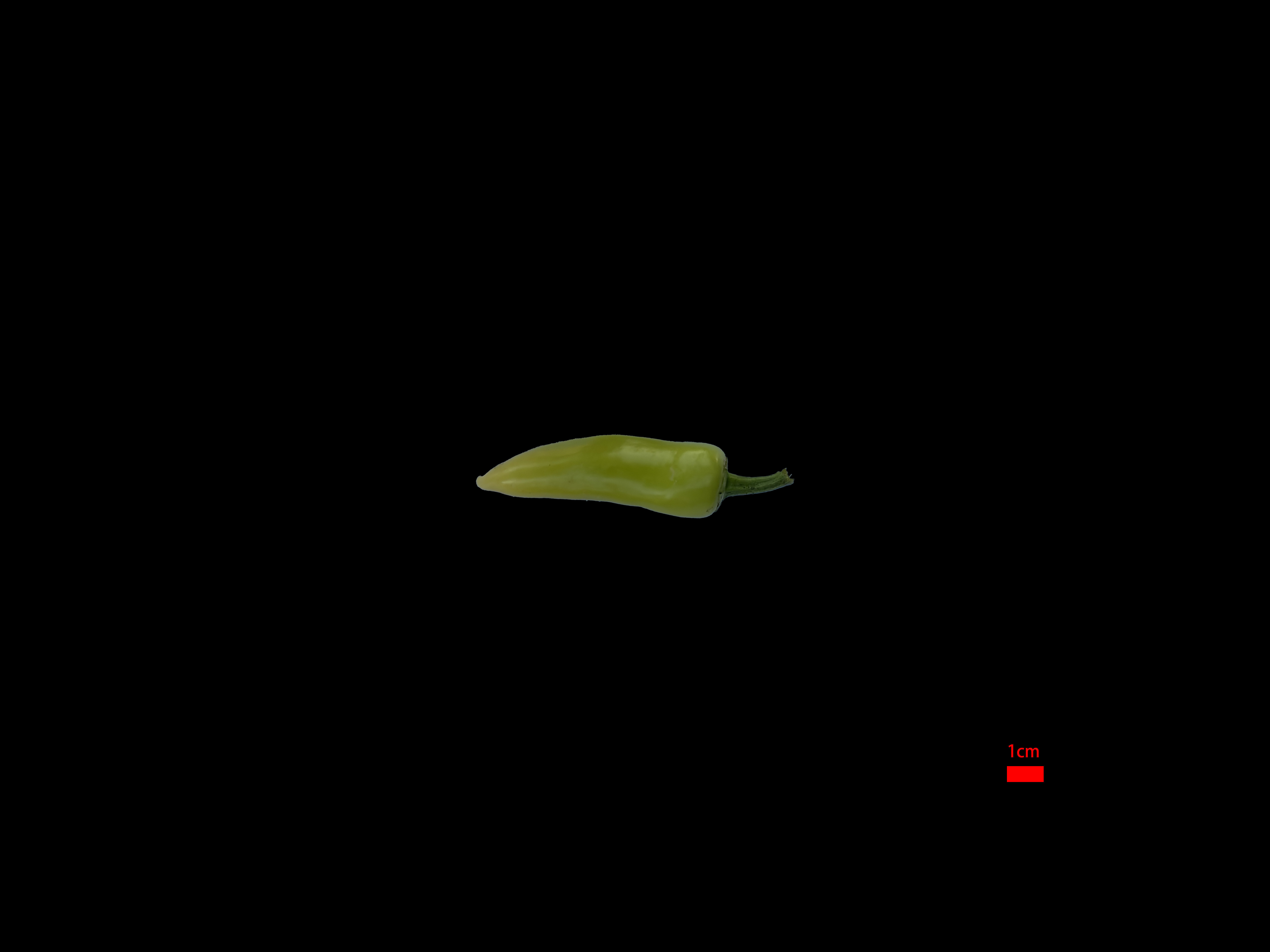

Supplement: Supplementary file 1 [file plants-15-02103-s001.zip › plants-4383327-supplementary/pepper_original_data/cone/17-2.jpg]

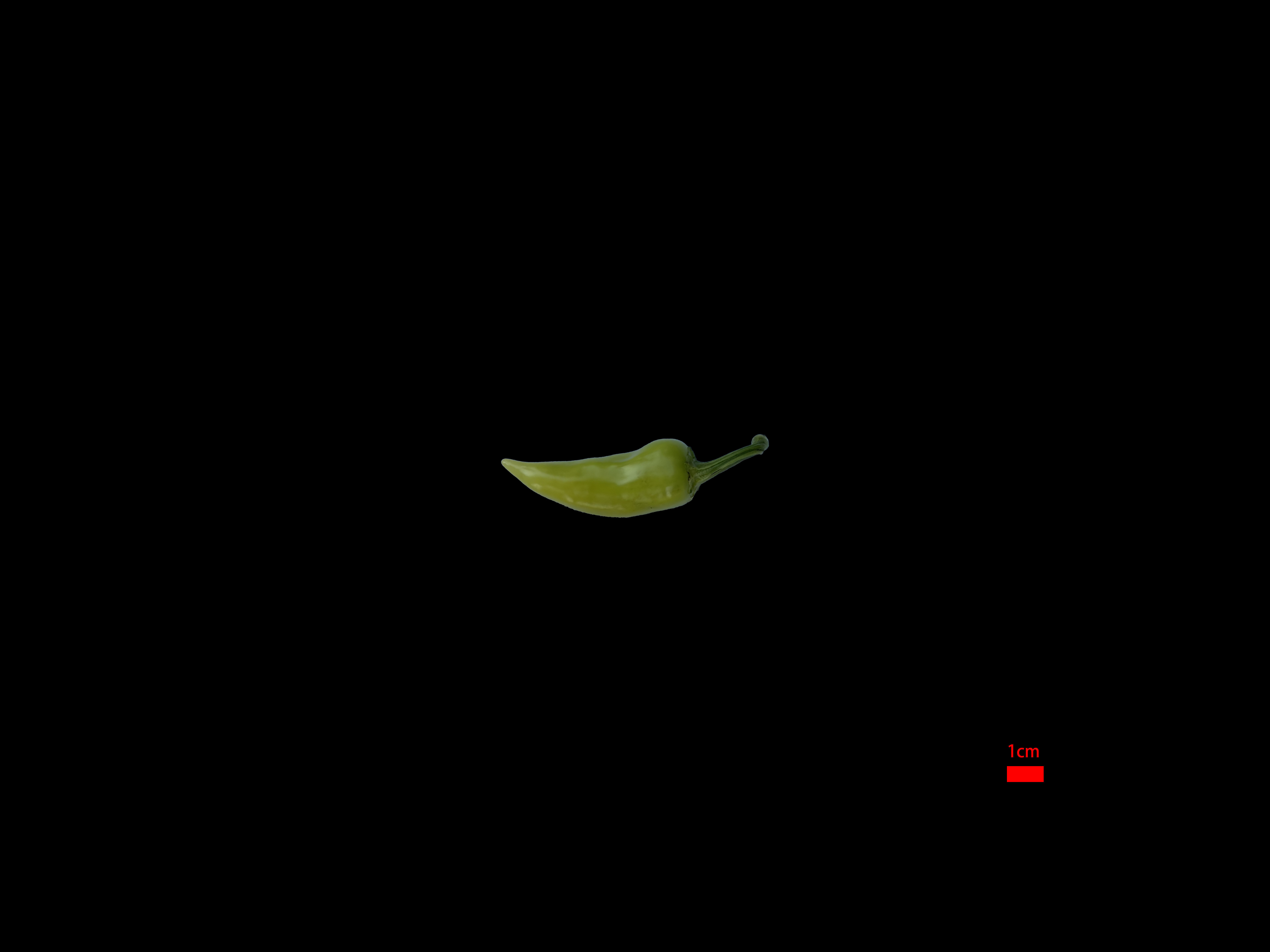

Supplement: Supplementary file 1 [file plants-15-02103-s001.zip › plants-4383327-supplementary/pepper_original_data/cone/17-4.jpg]

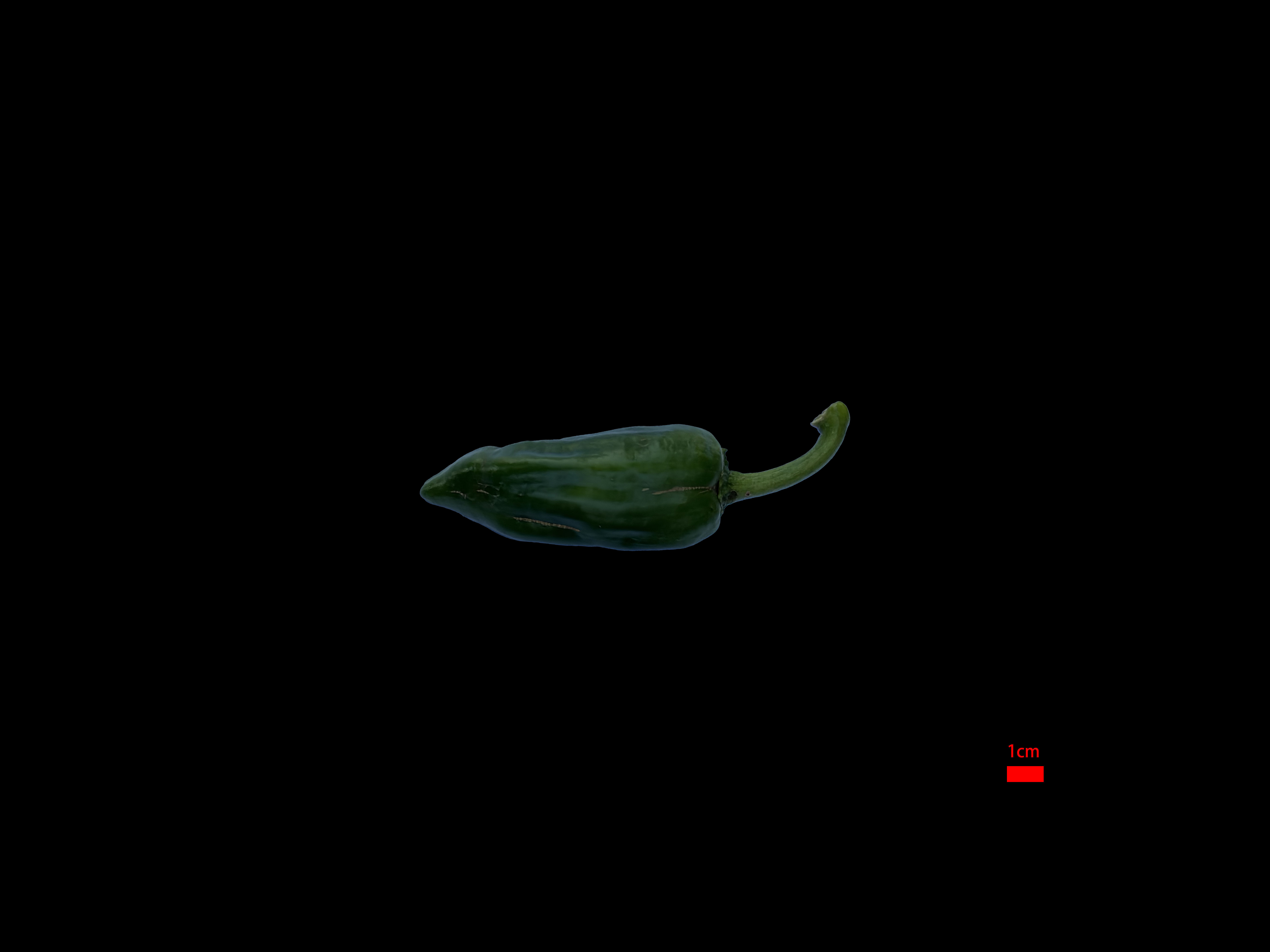

Supplement: Supplementary file 1 [file plants-15-02103-s001.zip › plants-4383327-supplementary/pepper_original_data/cone/172-1.jpg]

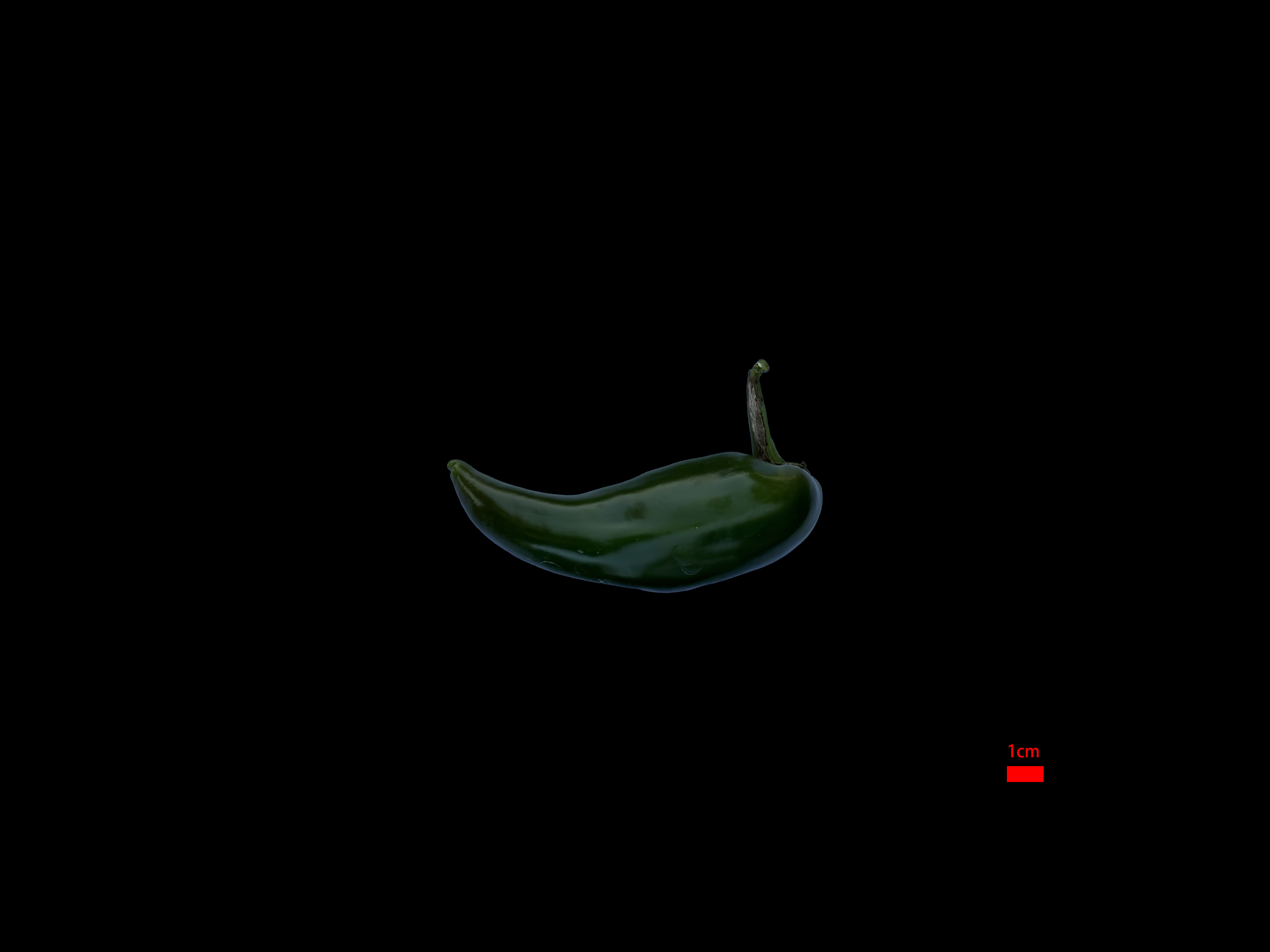

Supplement: Supplementary file 1 [file plants-15-02103-s001.zip › plants-4383327-supplementary/pepper_original_data/cone/172-2.jpg]

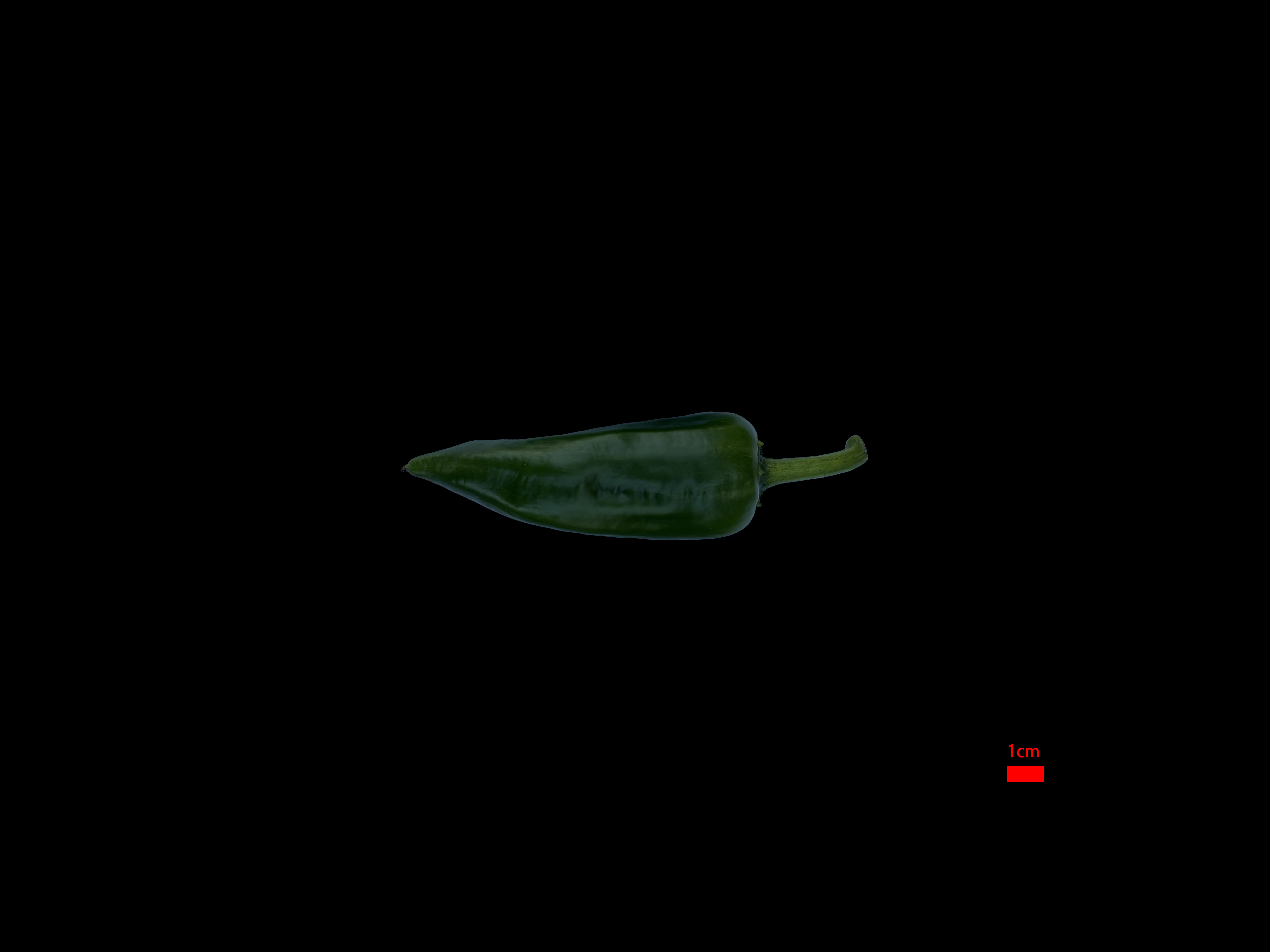

Supplement: Supplementary file 1 [file plants-15-02103-s001.zip › plants-4383327-supplementary/pepper_original_data/cone/172-3.jpg]

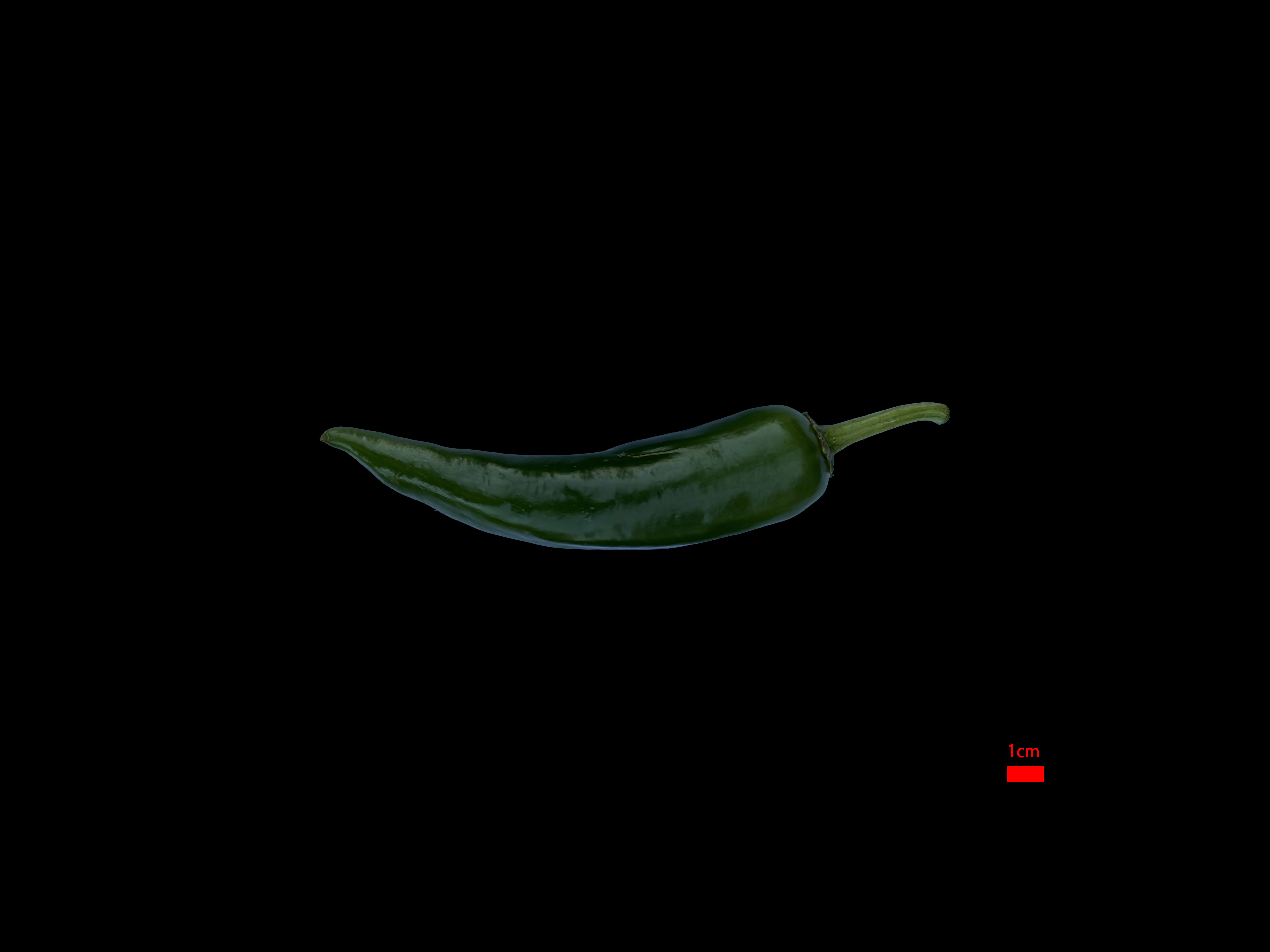

Supplement: Supplementary file 1 [file plants-15-02103-s001.zip › plants-4383327-supplementary/pepper_original_data/cone/172-4.jpg]

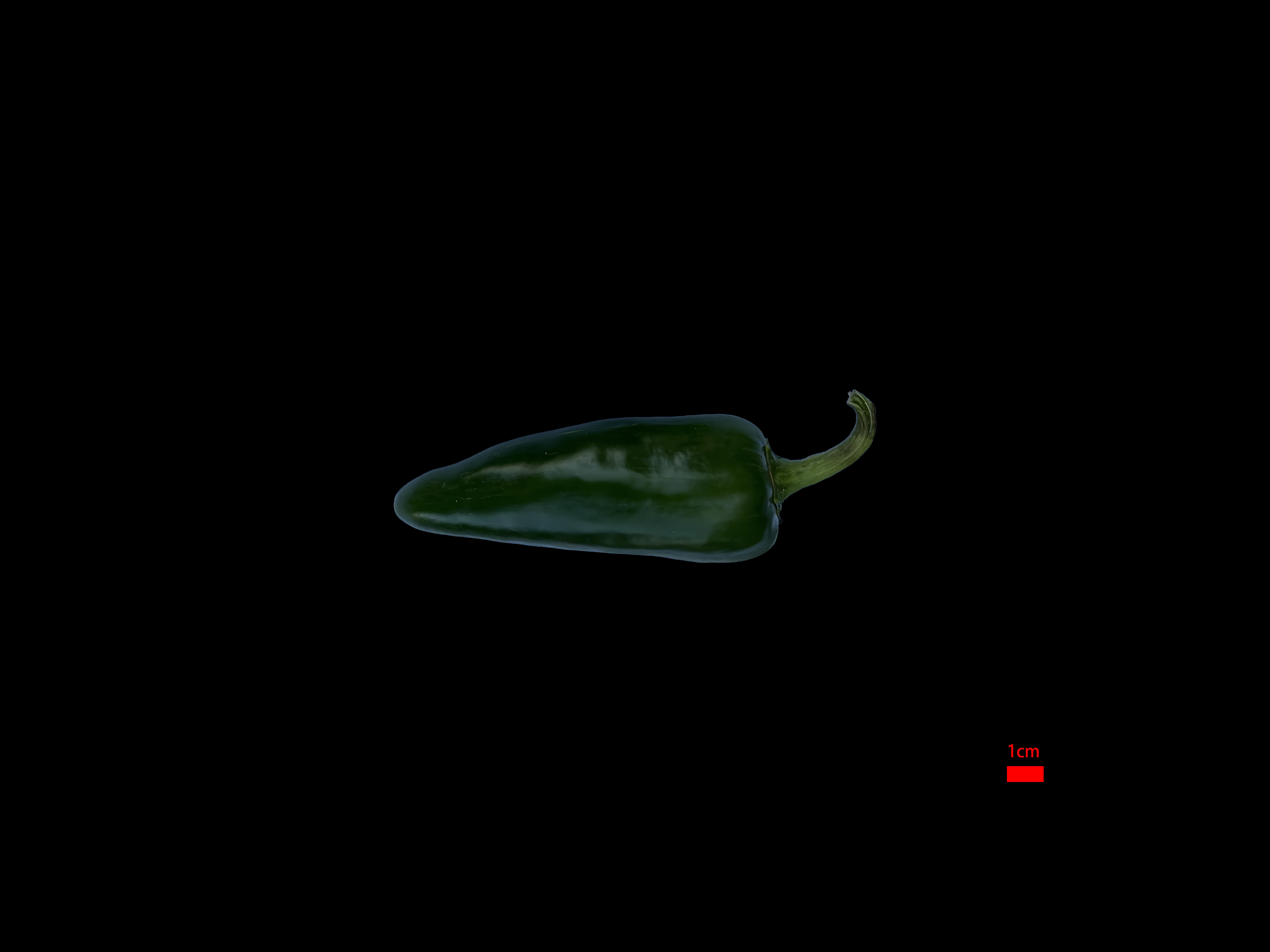

Supplement: Supplementary file 1 [file plants-15-02103-s001.zip › plants-4383327-supplementary/pepper_original_data/cone/172-5.jpg]

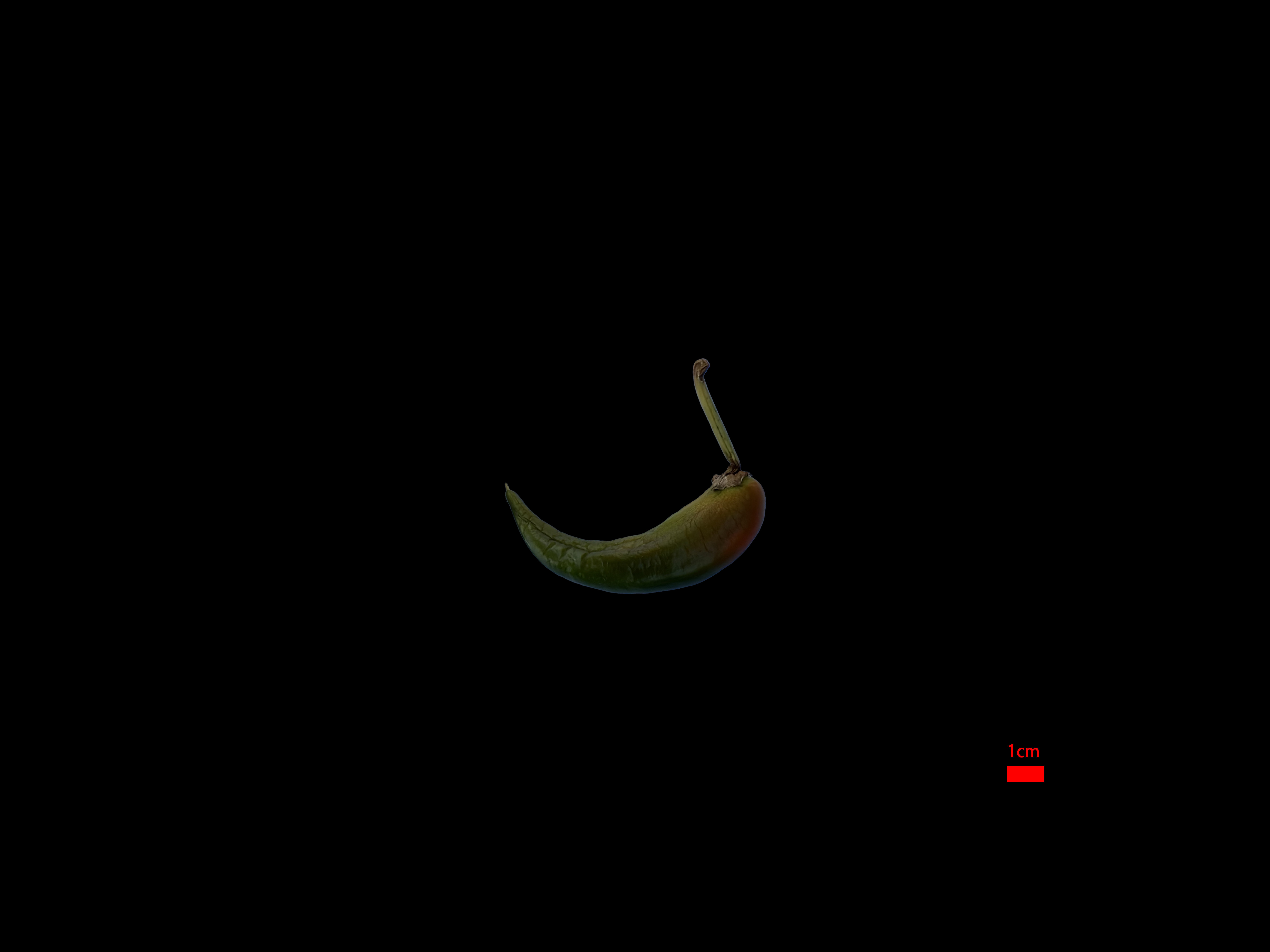

Supplement: Supplementary file 1 [file plants-15-02103-s001.zip › plants-4383327-supplementary/pepper_original_data/cone/172-6.jpg]

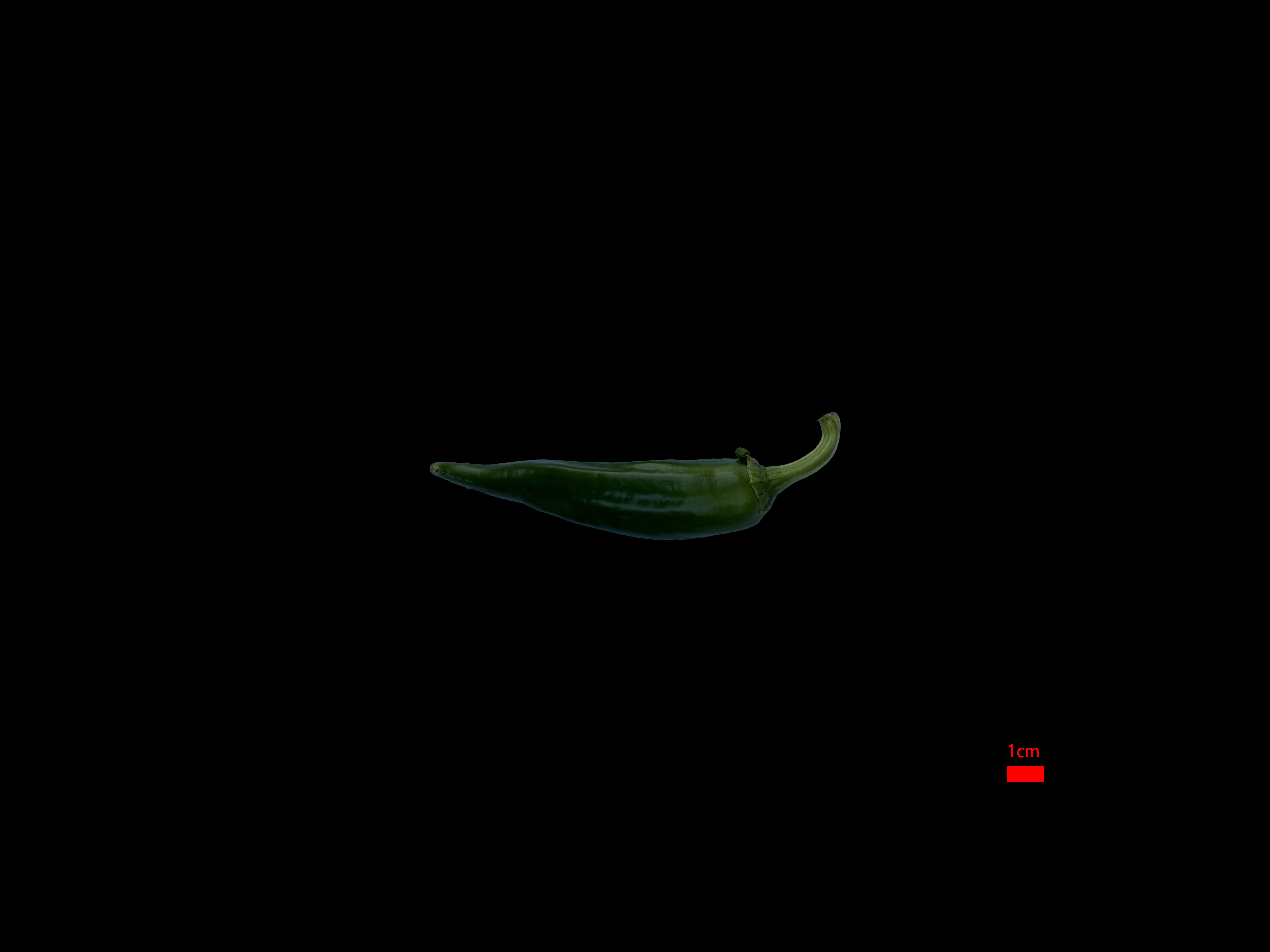

Supplement: Supplementary file 1 [file plants-15-02103-s001.zip › plants-4383327-supplementary/pepper_original_data/cone/172-7.jpg]

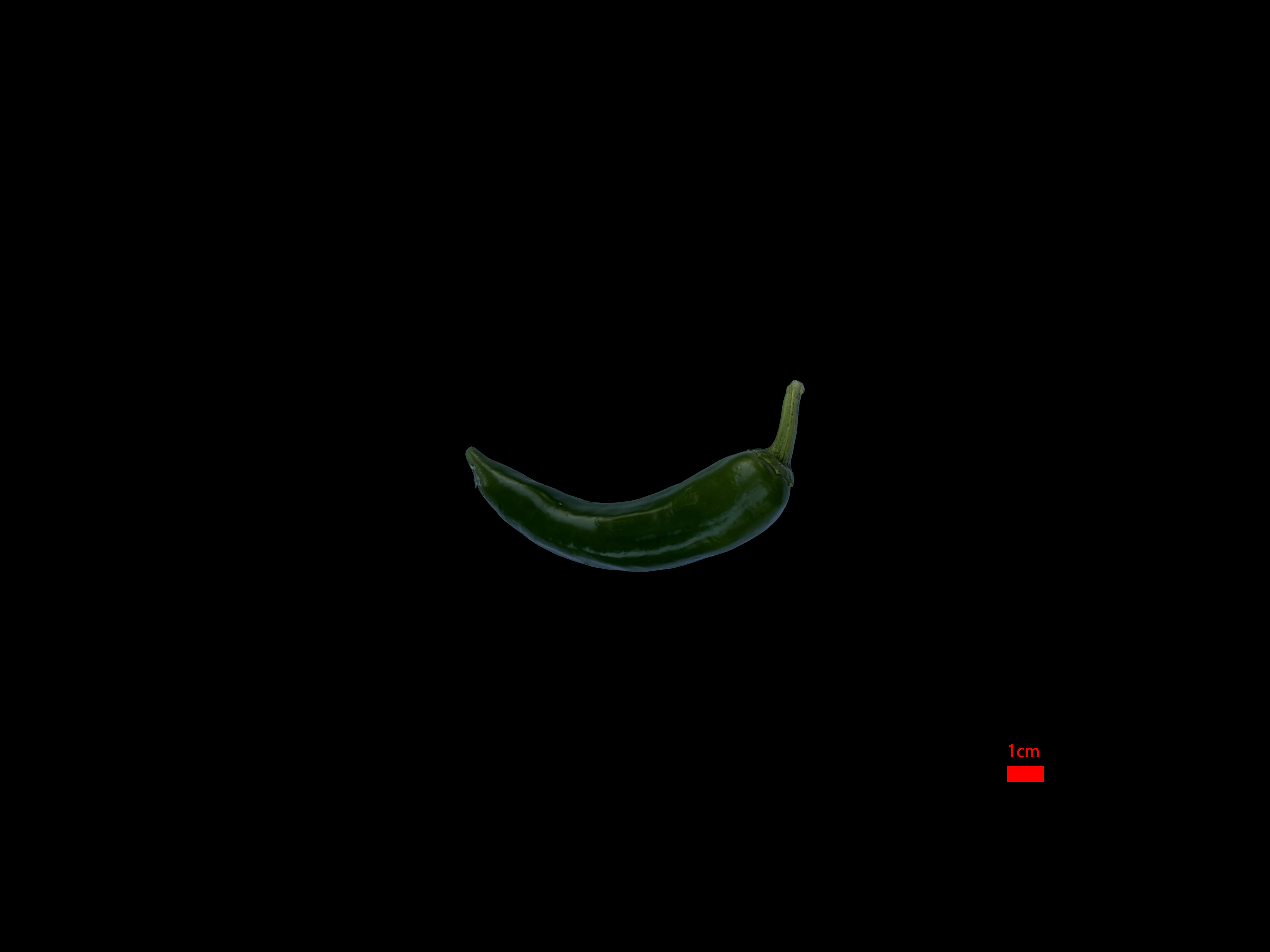

Supplement: Supplementary file 1 [file plants-15-02103-s001.zip › plants-4383327-supplementary/pepper_original_data/cone/172-8.jpg]

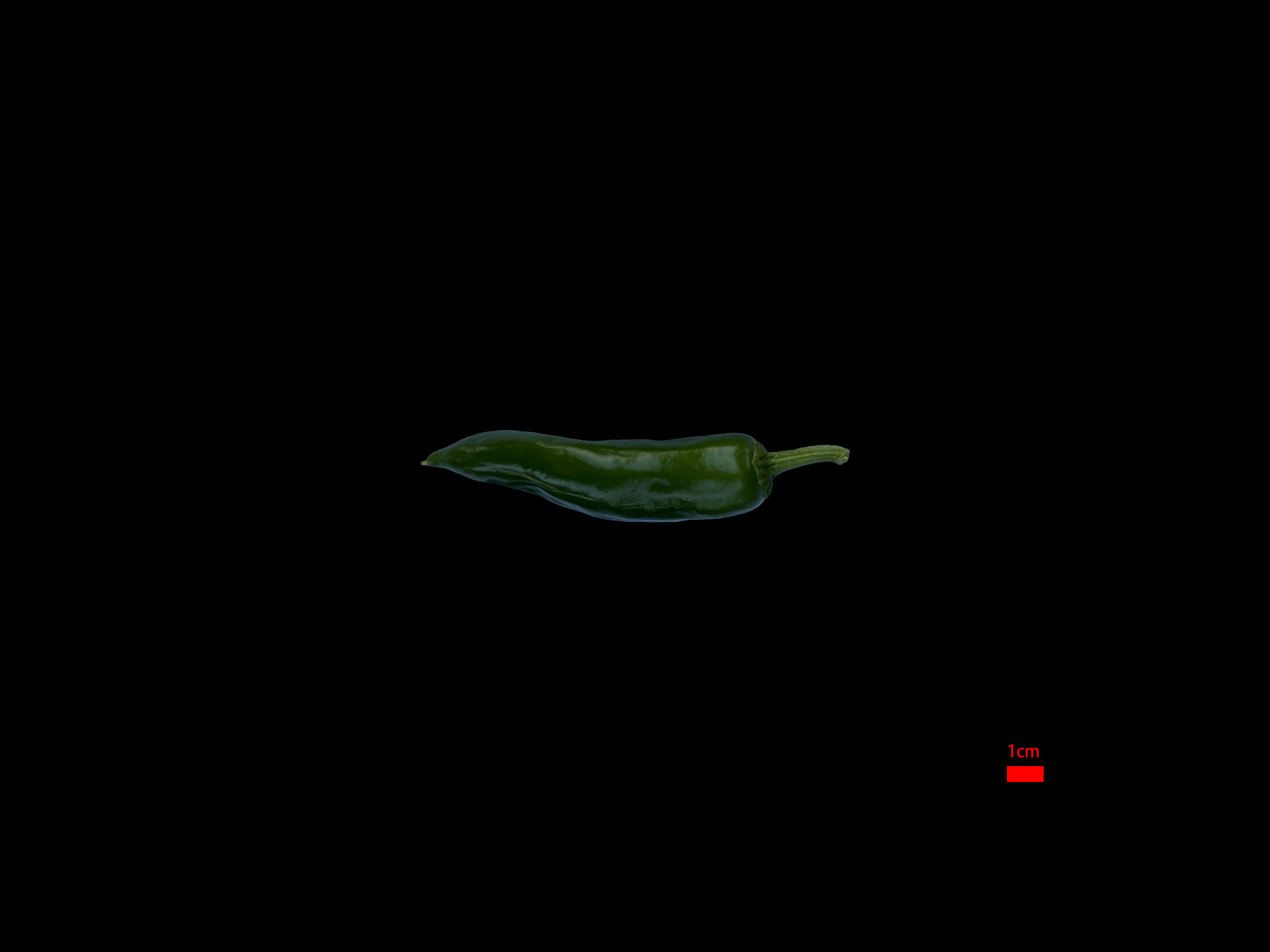

Supplement: Supplementary file 1 [file plants-15-02103-s001.zip › plants-4383327-supplementary/pepper_original_data/cone/172-9.jpg]

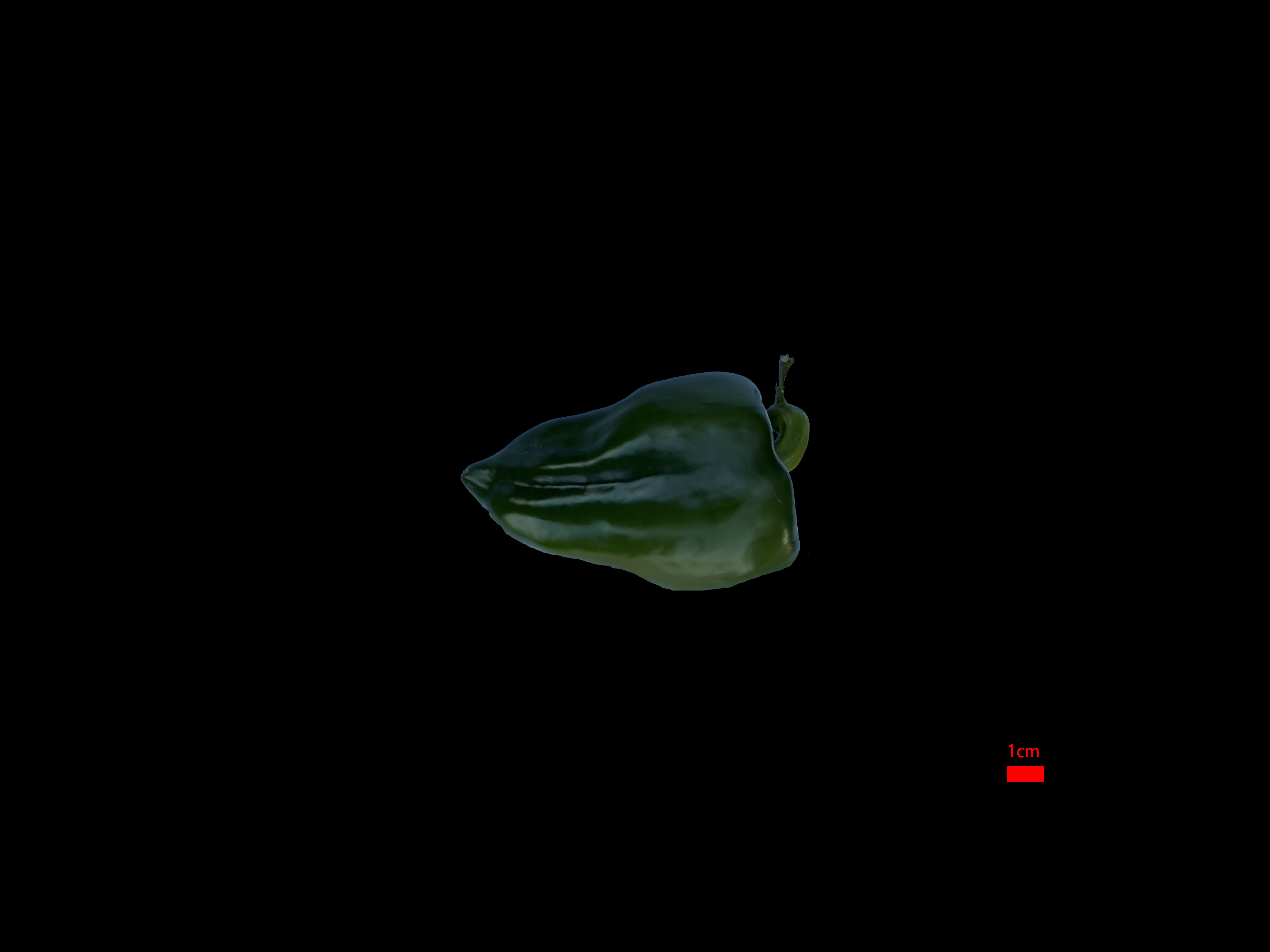

Supplement: Supplementary file 1 [file plants-15-02103-s001.zip › plants-4383327-supplementary/pepper_original_data/cone/174-1.jpg]

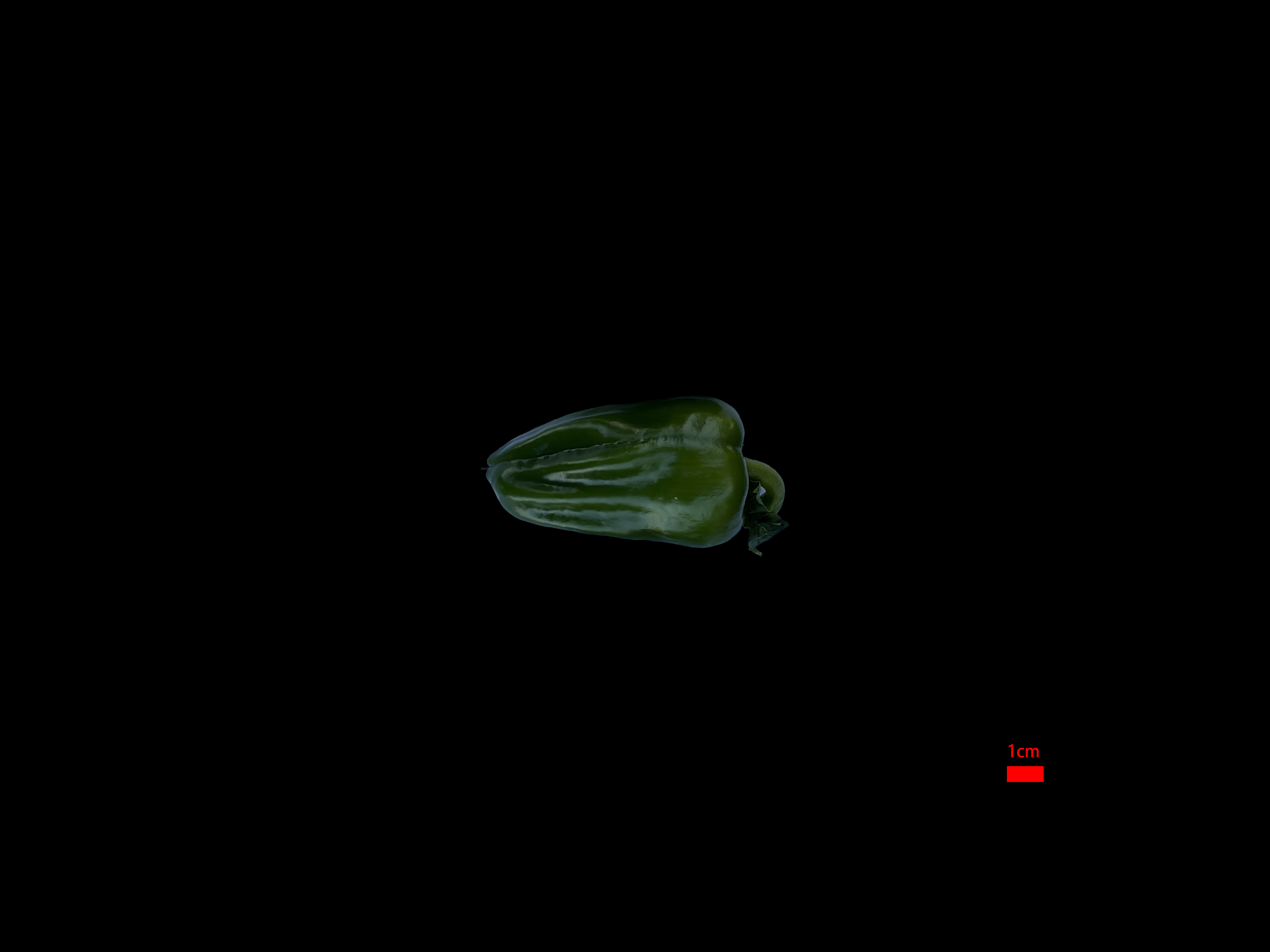

Supplement: Supplementary file 1 [file plants-15-02103-s001.zip › plants-4383327-supplementary/pepper_original_data/cone/174-10.jpg]

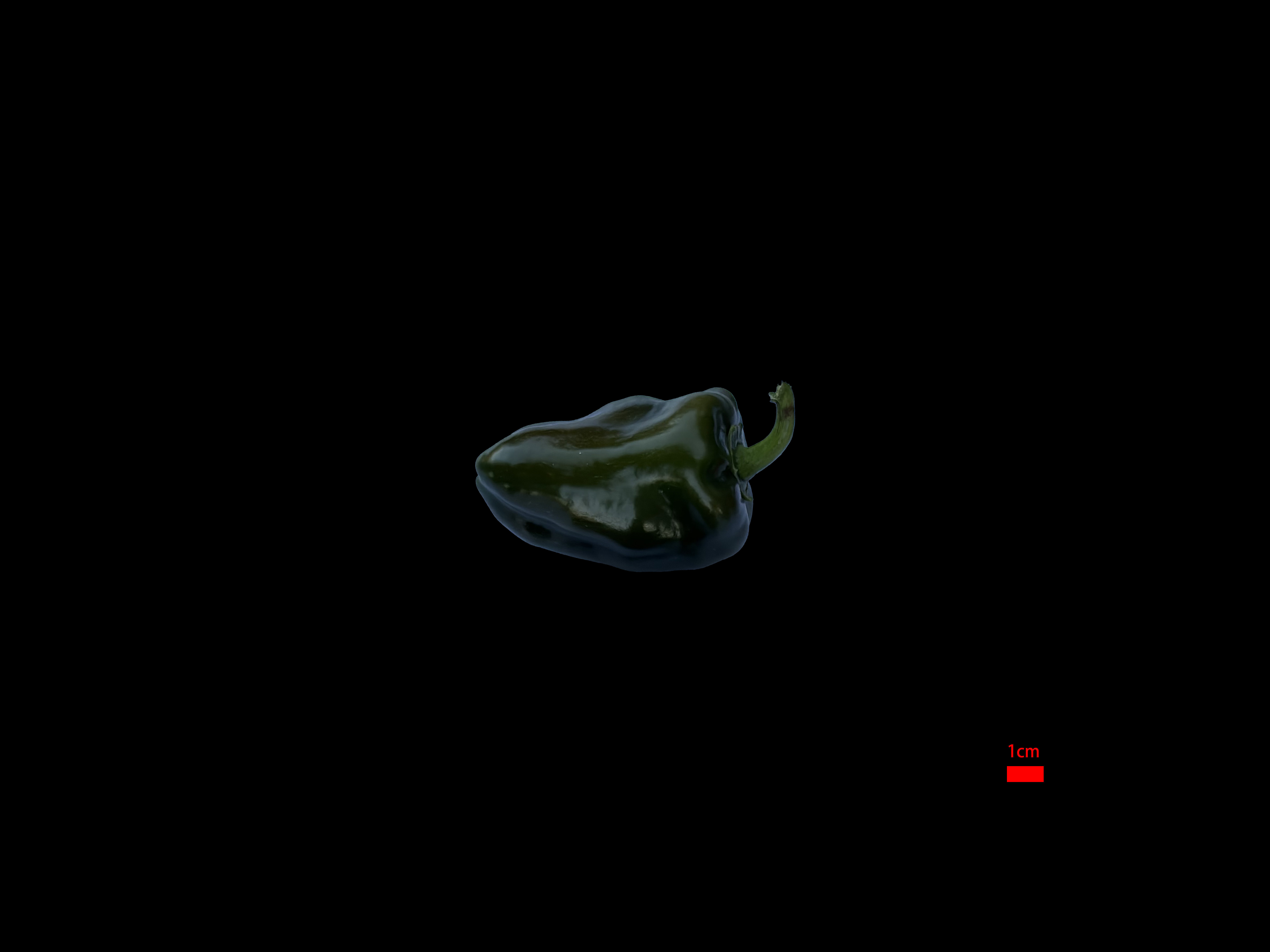

Supplement: Supplementary file 1 [file plants-15-02103-s001.zip › plants-4383327-supplementary/pepper_original_data/cone/174-12.jpg]

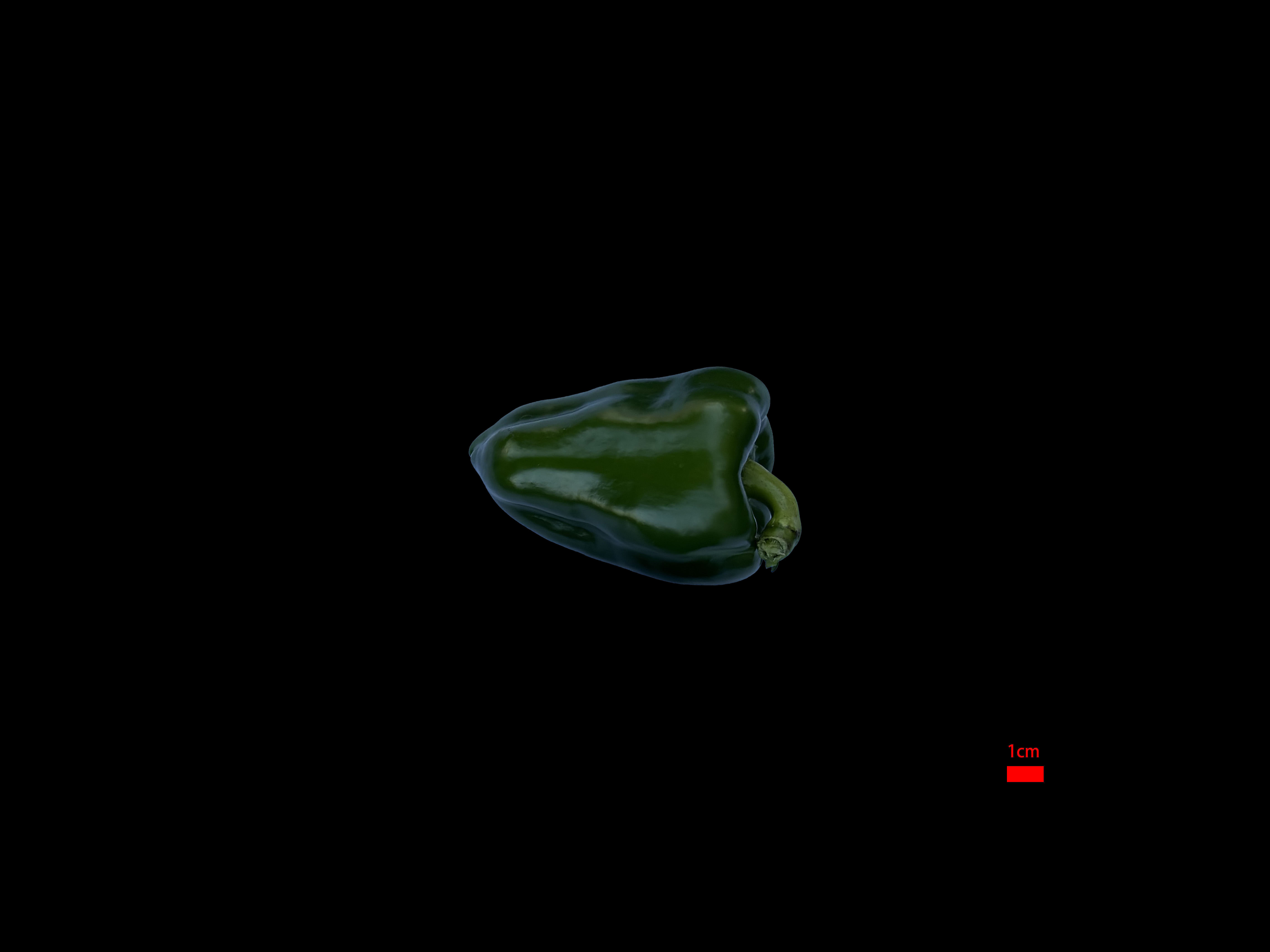

Supplement: Supplementary file 1 [file plants-15-02103-s001.zip › plants-4383327-supplementary/pepper_original_data/cone/174-2.jpg]

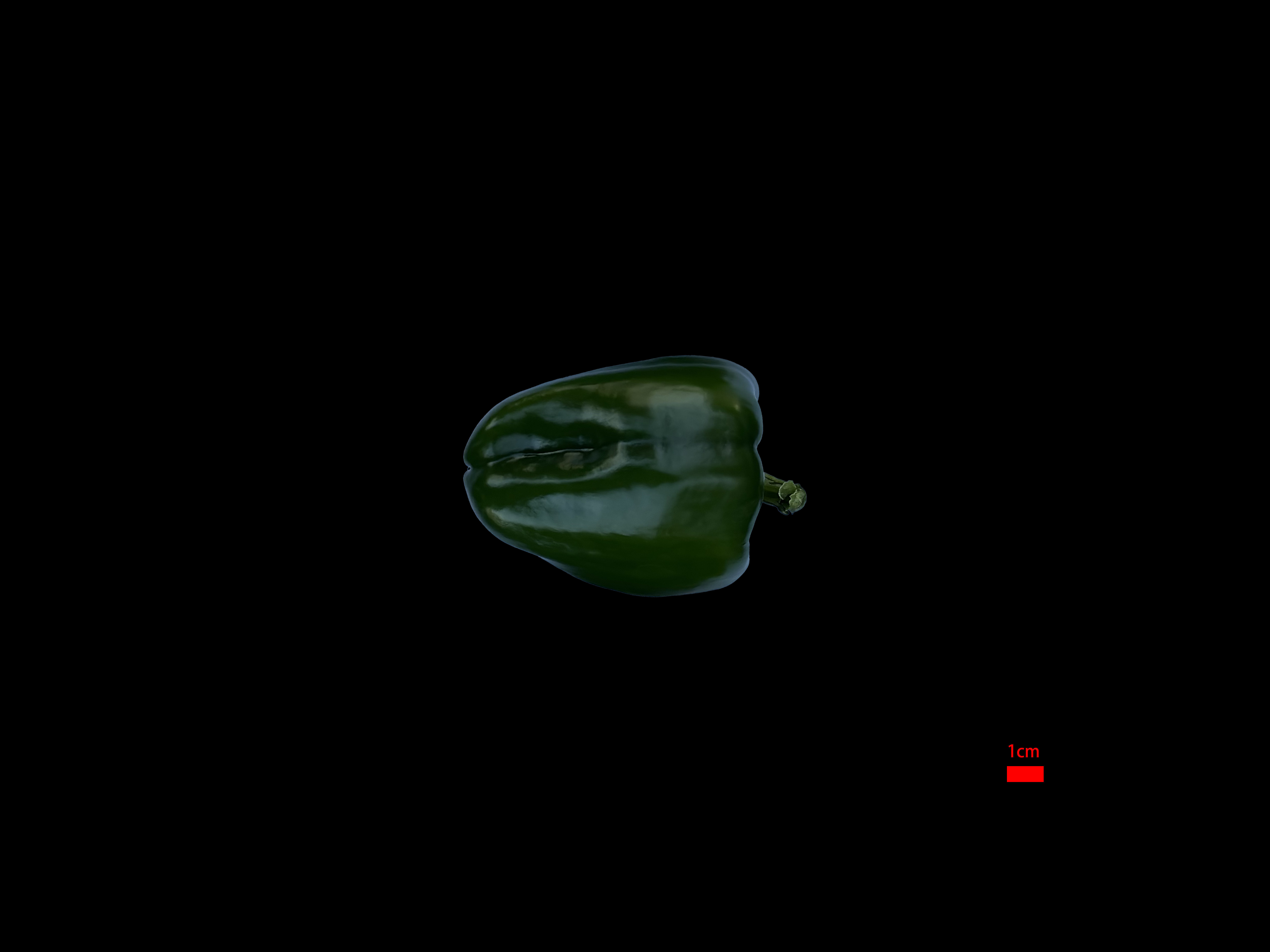

Supplement: Supplementary file 1 [file plants-15-02103-s001.zip › plants-4383327-supplementary/pepper_original_data/cone/174-3.jpg]

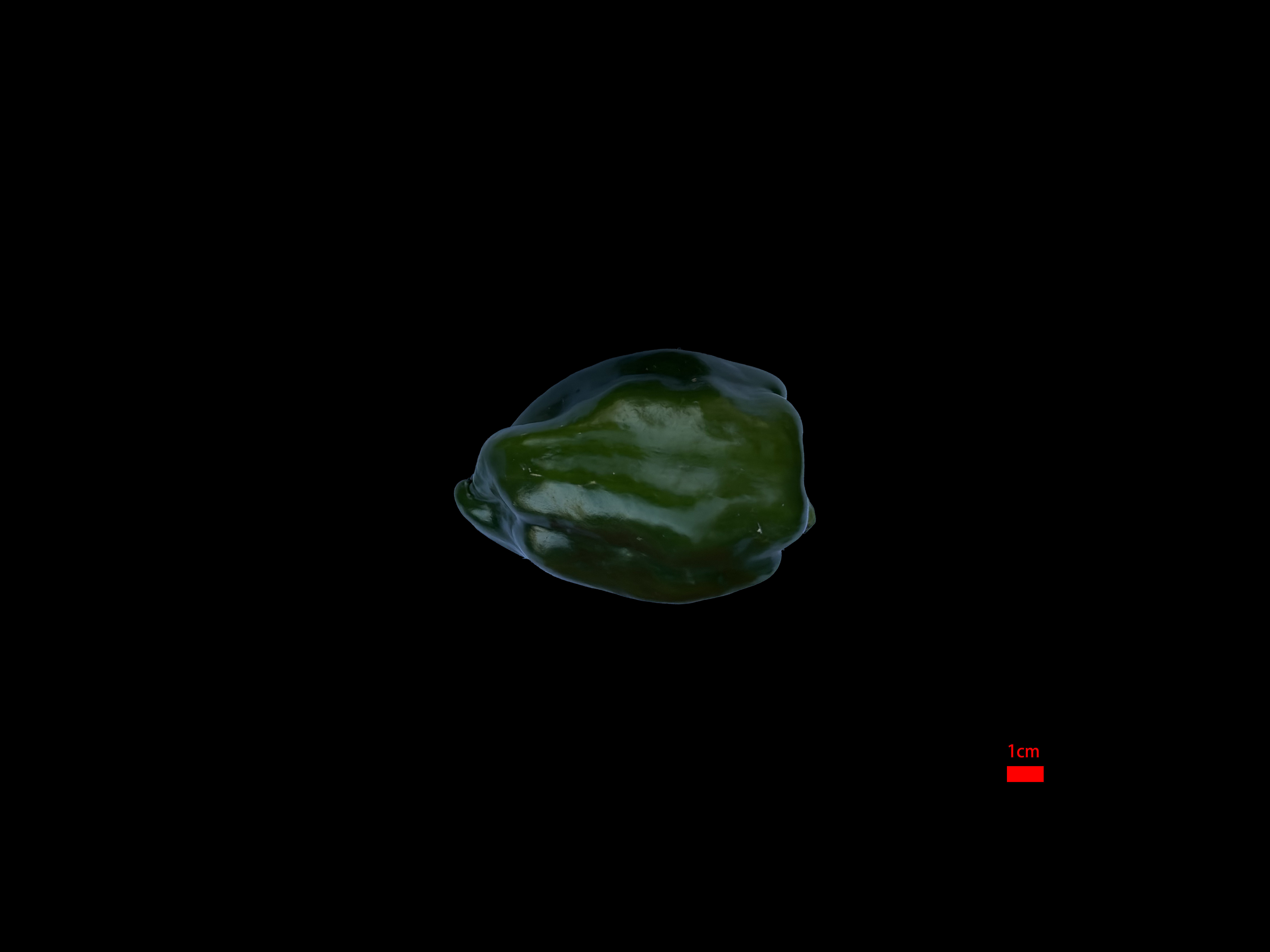

Supplement: Supplementary file 1 [file plants-15-02103-s001.zip › plants-4383327-supplementary/pepper_original_data/cone/174-4.jpg]

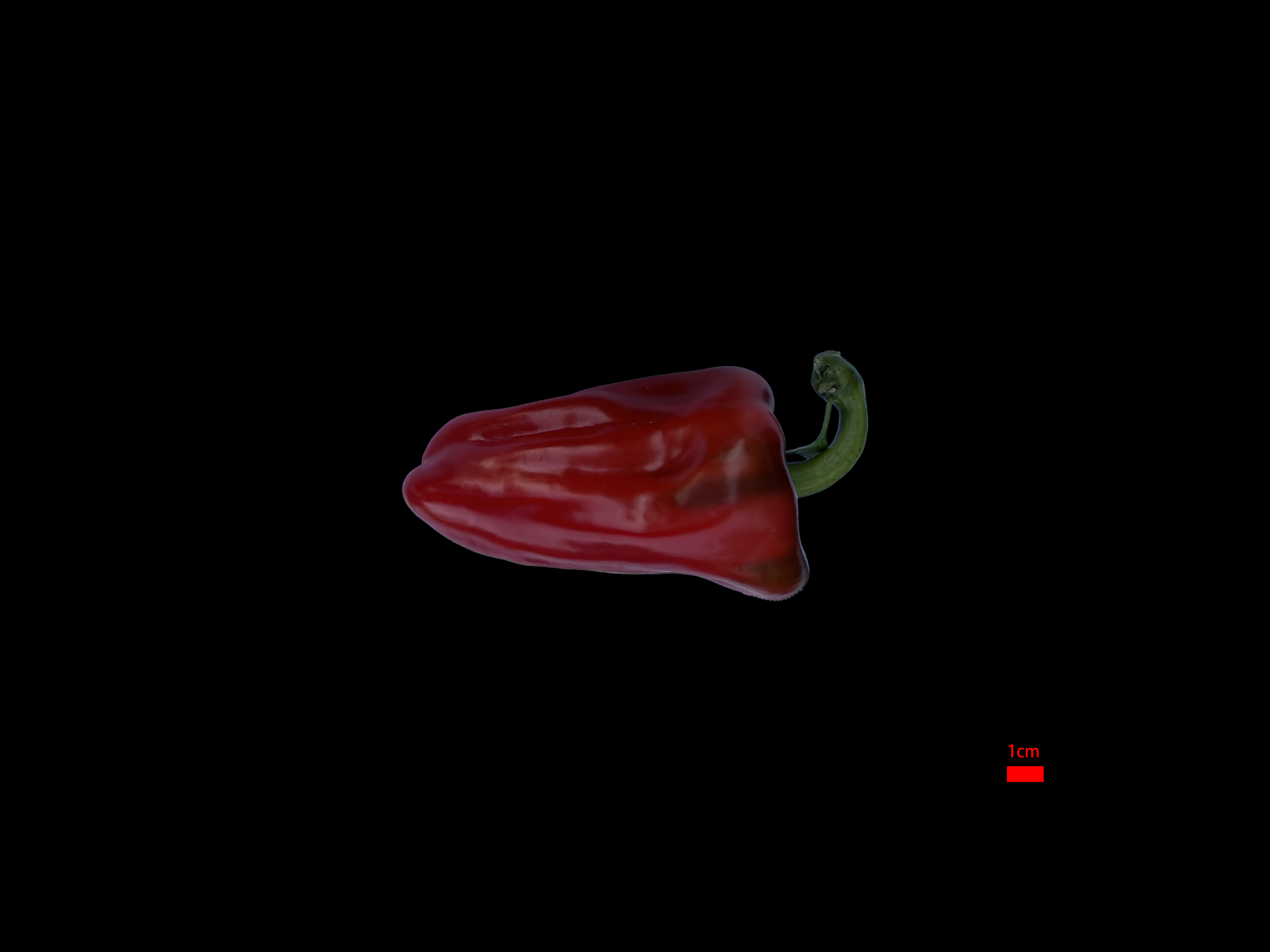

Supplement: Supplementary file 1 [file plants-15-02103-s001.zip › plants-4383327-supplementary/pepper_original_data/cone/174-5.jpg]

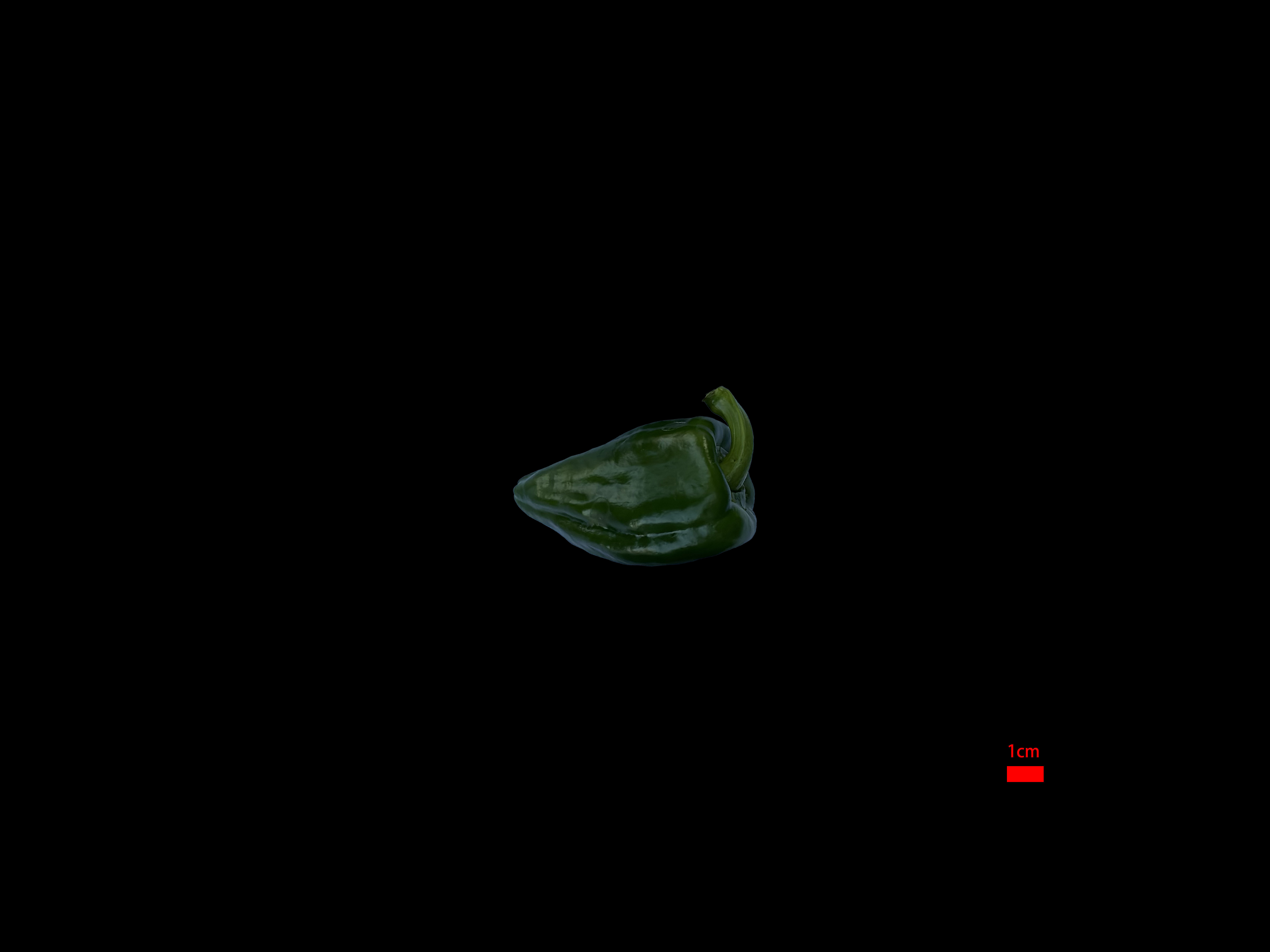

Supplement: Supplementary file 1 [file plants-15-02103-s001.zip › plants-4383327-supplementary/pepper_original_data/cone/174-9.jpg]

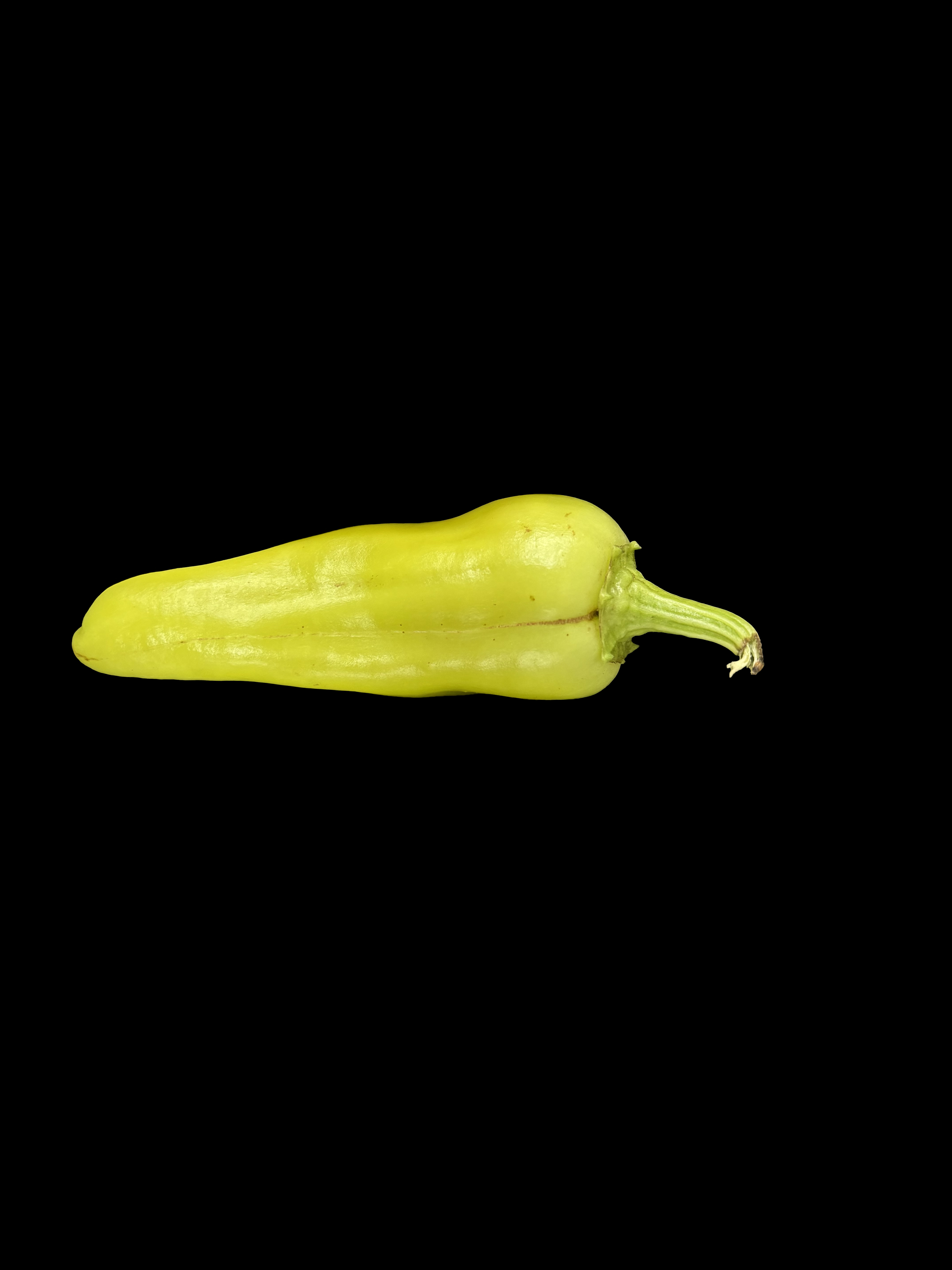

Supplement: Supplementary file 1 [file plants-15-02103-s001.zip › plants-4383327-supplementary/pepper_original_data/cone/178.1.jpg]

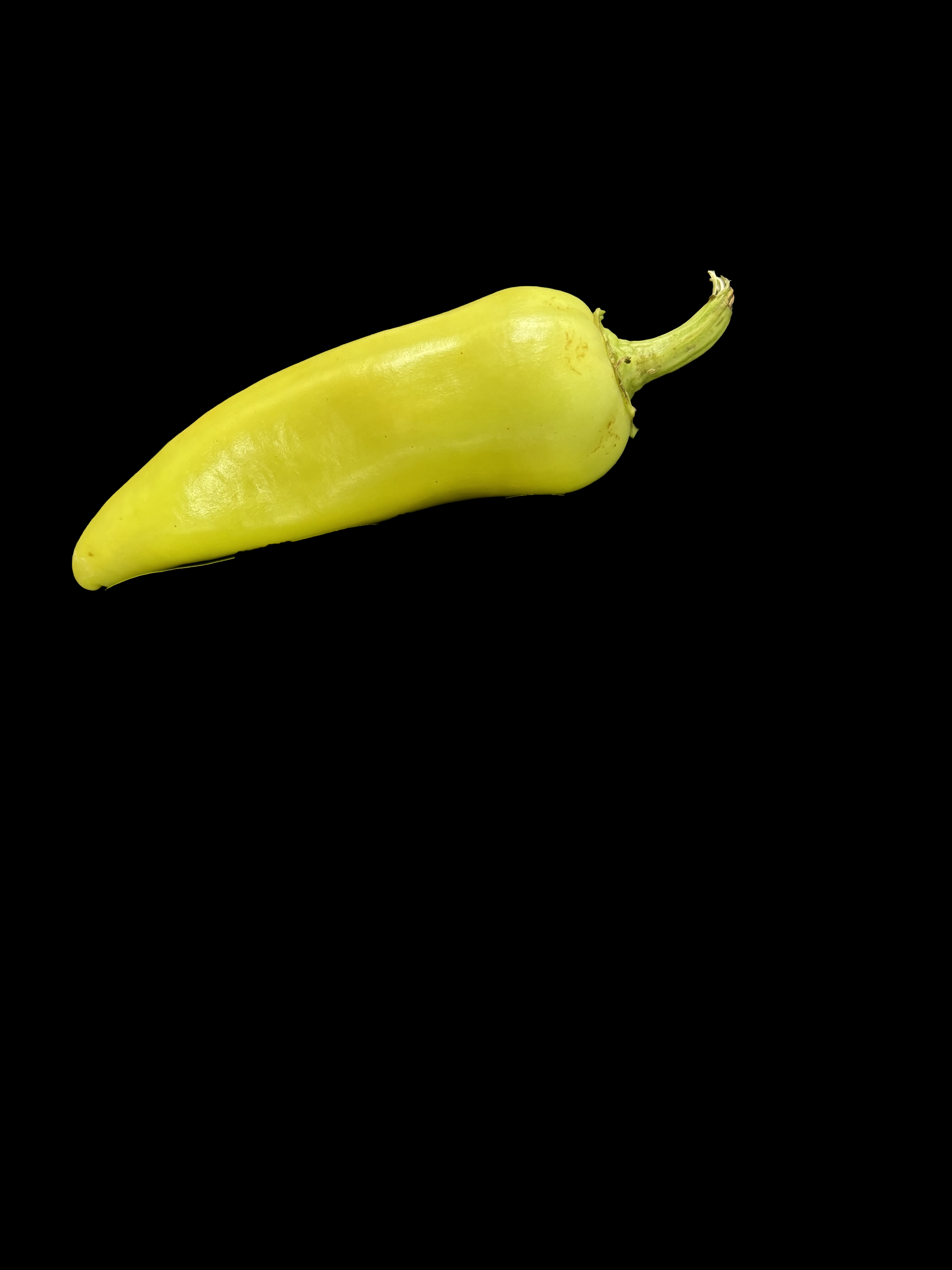

Supplement: Supplementary file 1 [file plants-15-02103-s001.zip › plants-4383327-supplementary/pepper_original_data/cone/178.jpg]

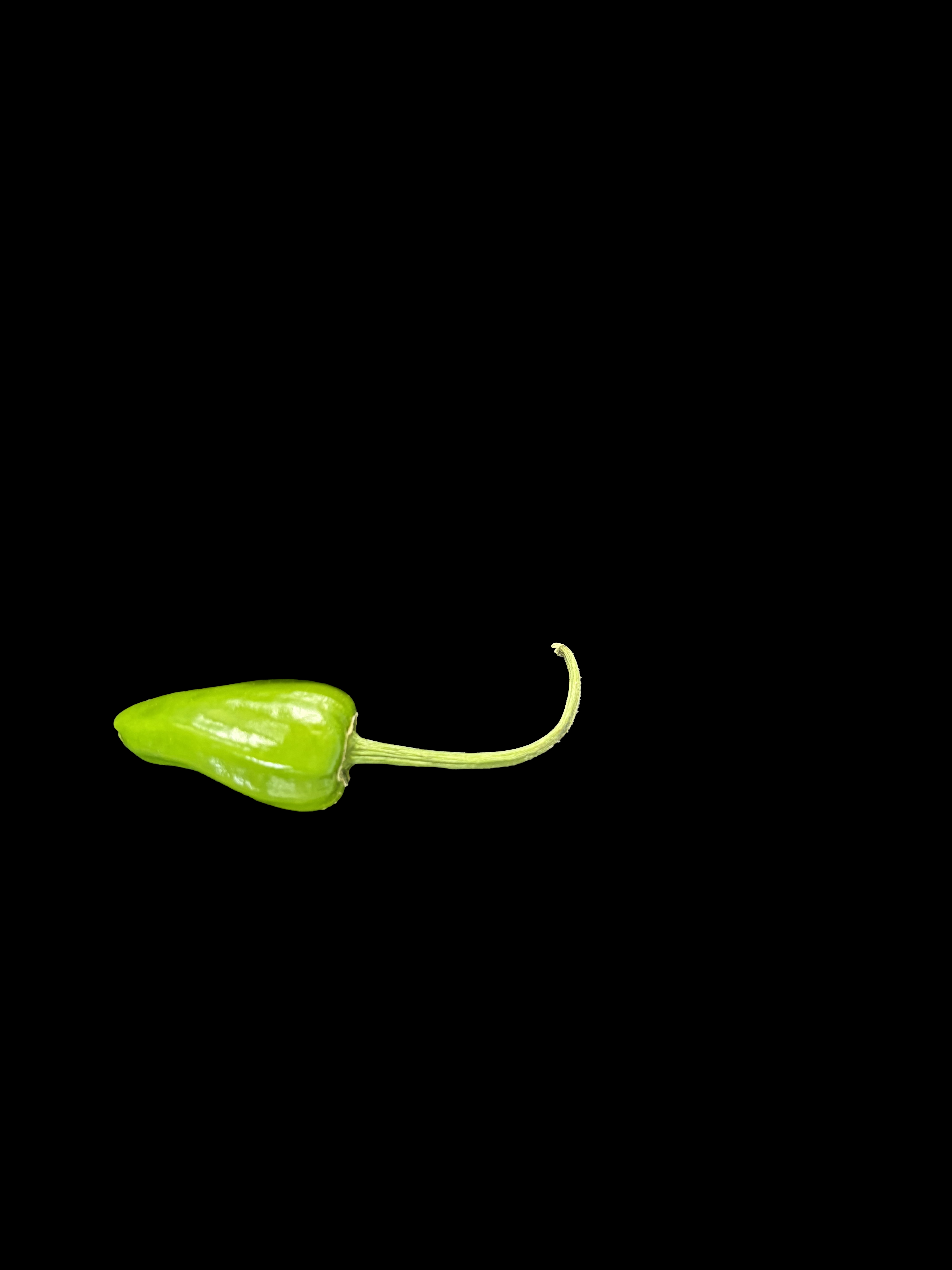

Supplement: Supplementary file 1 [file plants-15-02103-s001.zip › plants-4383327-supplementary/pepper_original_data/cone/183.1.jpg]

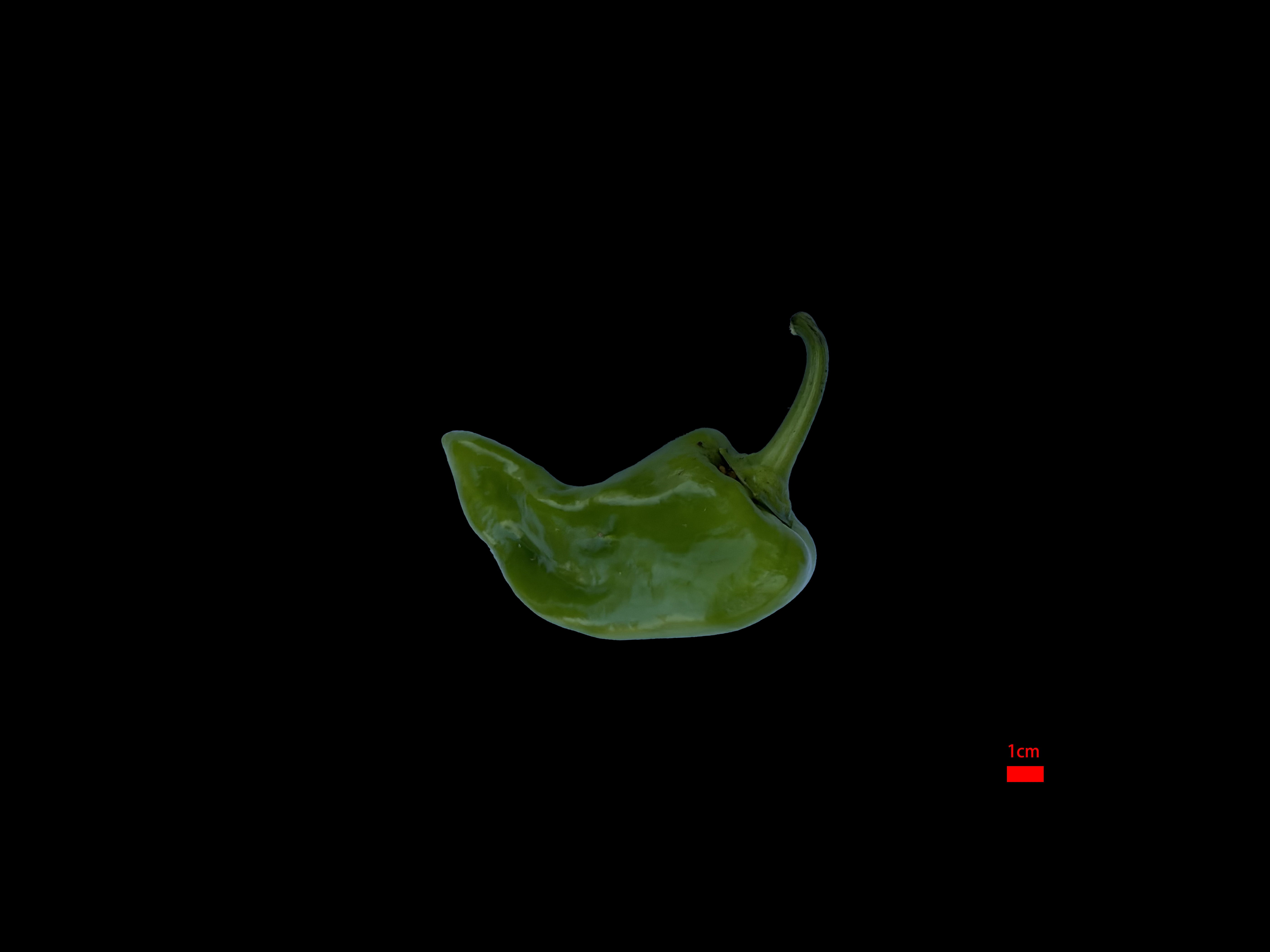

Supplement: Supplementary file 1 [file plants-15-02103-s001.zip › plants-4383327-supplementary/pepper_original_data/cone/188-12.jpg]

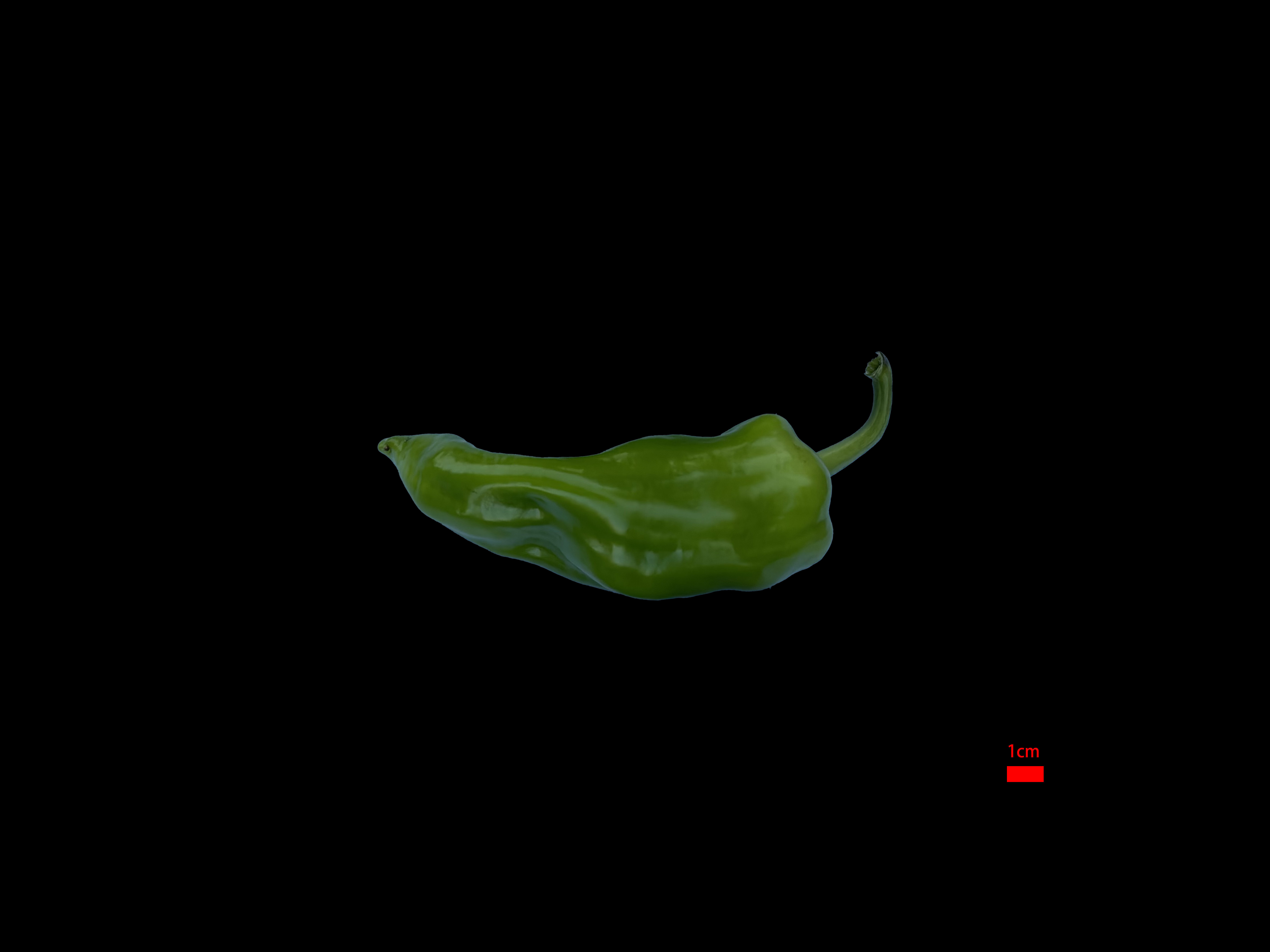

Supplement: Supplementary file 1 [file plants-15-02103-s001.zip › plants-4383327-supplementary/pepper_original_data/cone/188-3.jpg]

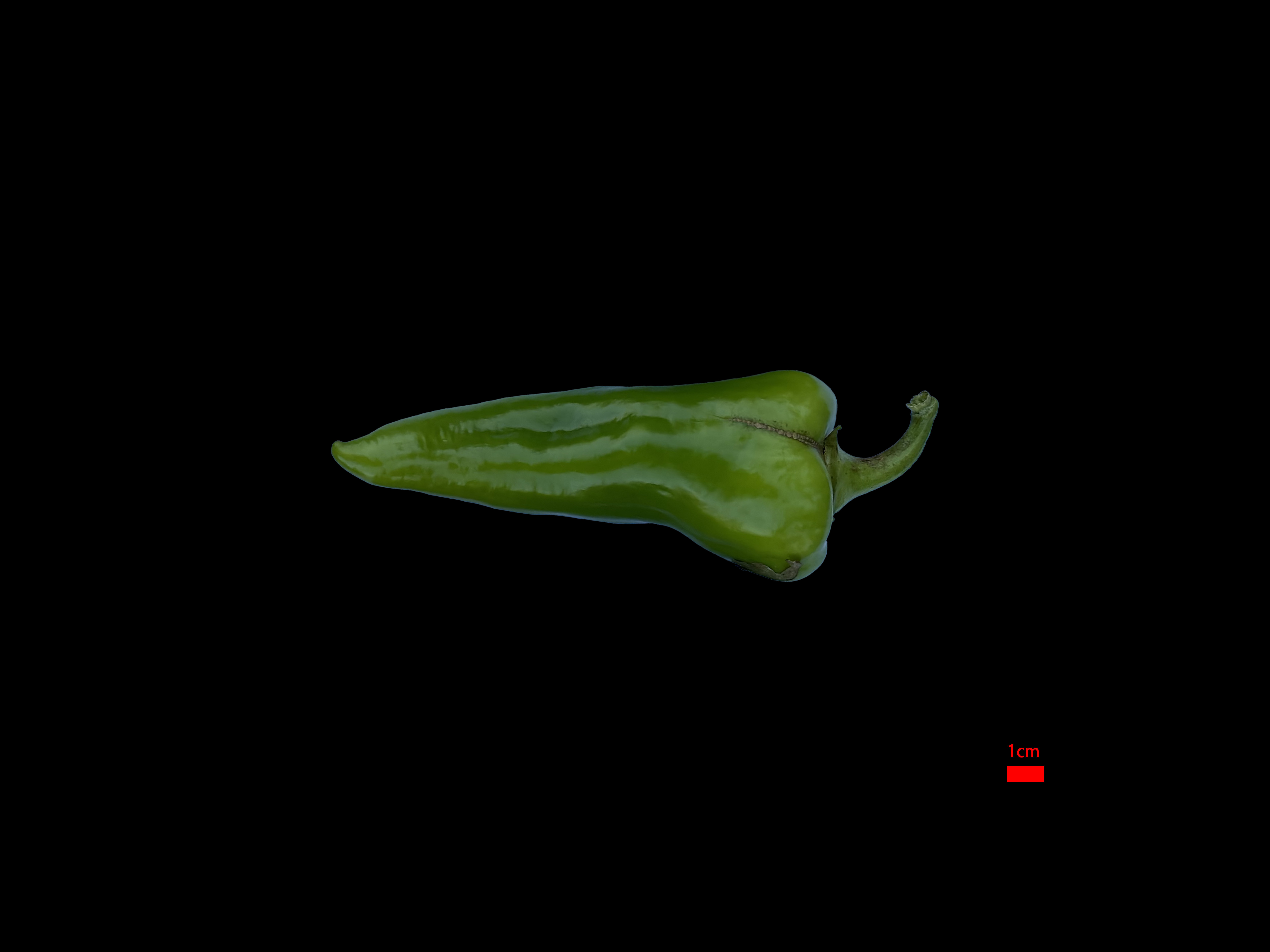

Supplement: Supplementary file 1 [file plants-15-02103-s001.zip › plants-4383327-supplementary/pepper_original_data/cone/188-4.jpg]

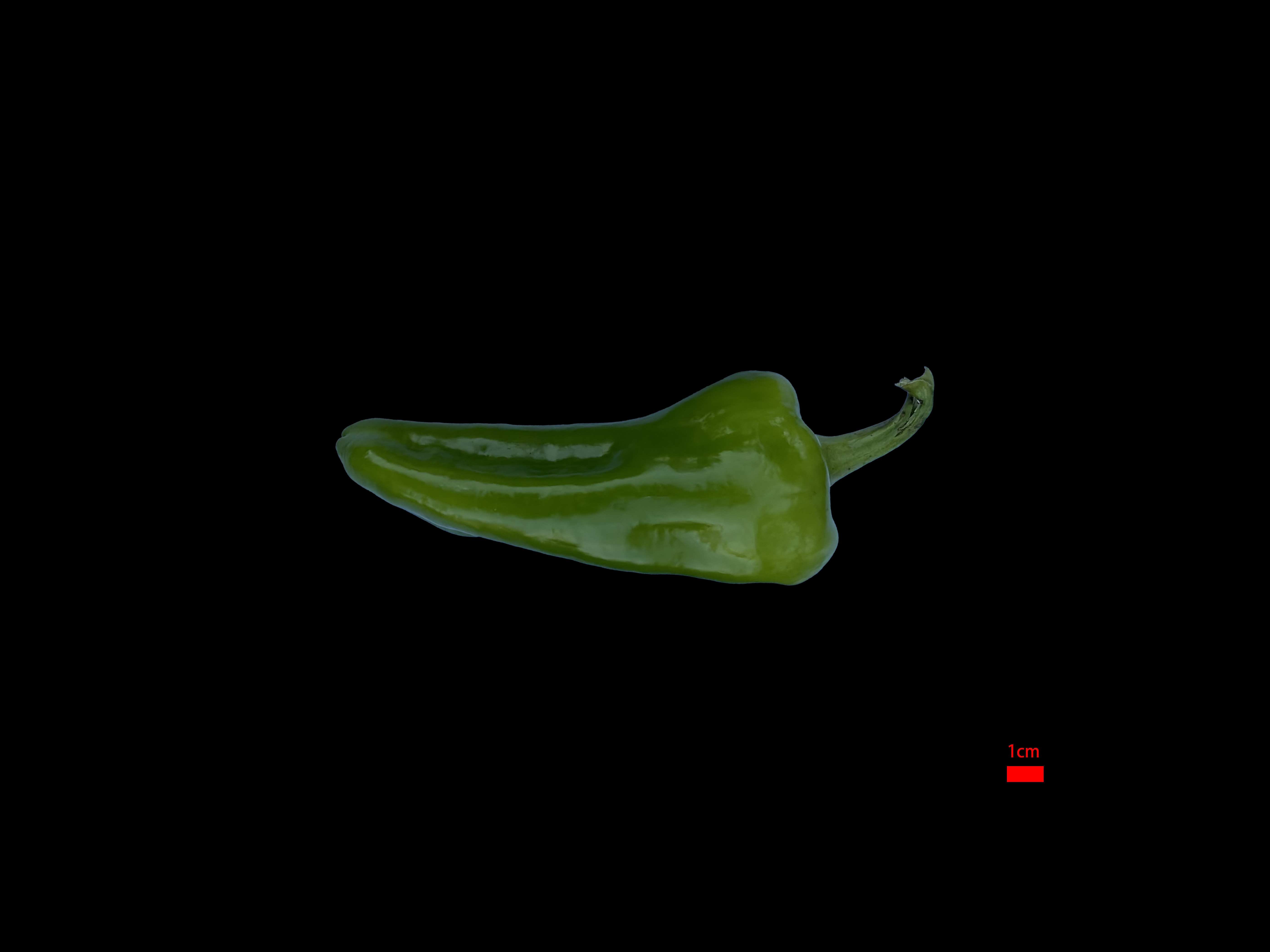

Supplement: Supplementary file 1 [file plants-15-02103-s001.zip › plants-4383327-supplementary/pepper_original_data/cone/188-5.jpg]

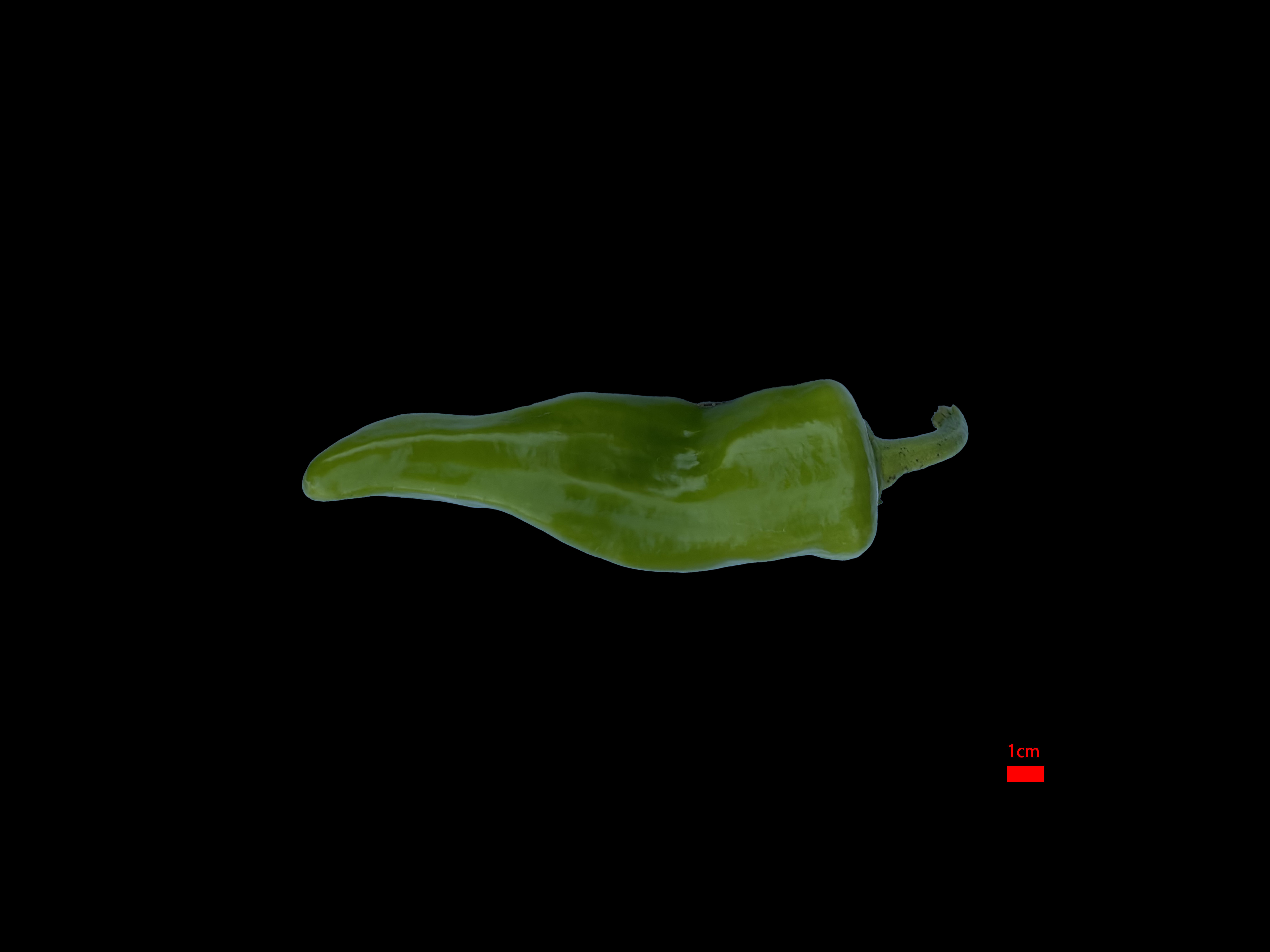

Supplement: Supplementary file 1 [file plants-15-02103-s001.zip › plants-4383327-supplementary/pepper_original_data/cone/188-6.jpg]

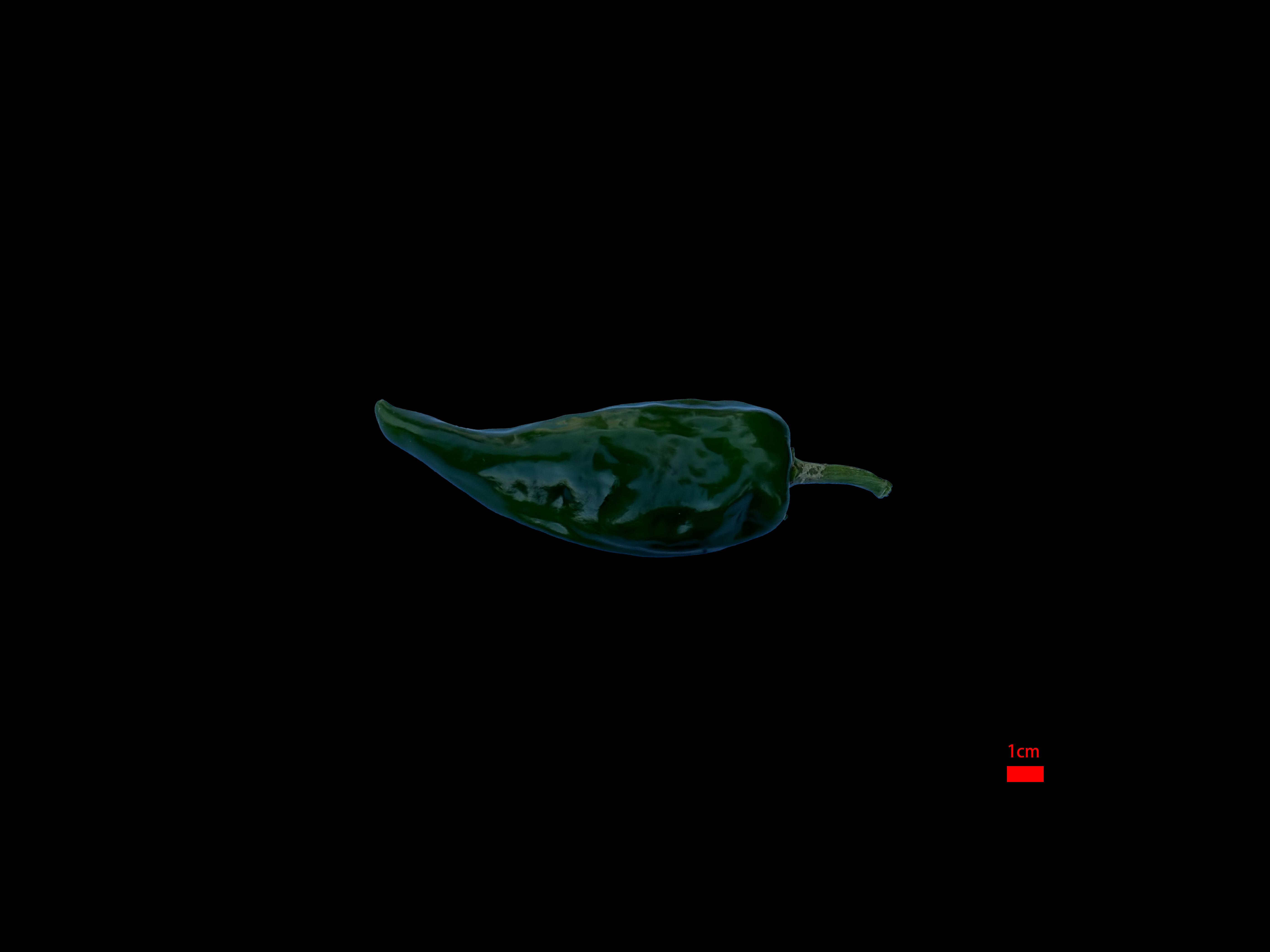

Supplement: Supplementary file 1 [file plants-15-02103-s001.zip › plants-4383327-supplementary/pepper_original_data/cone/219-1.jpg]

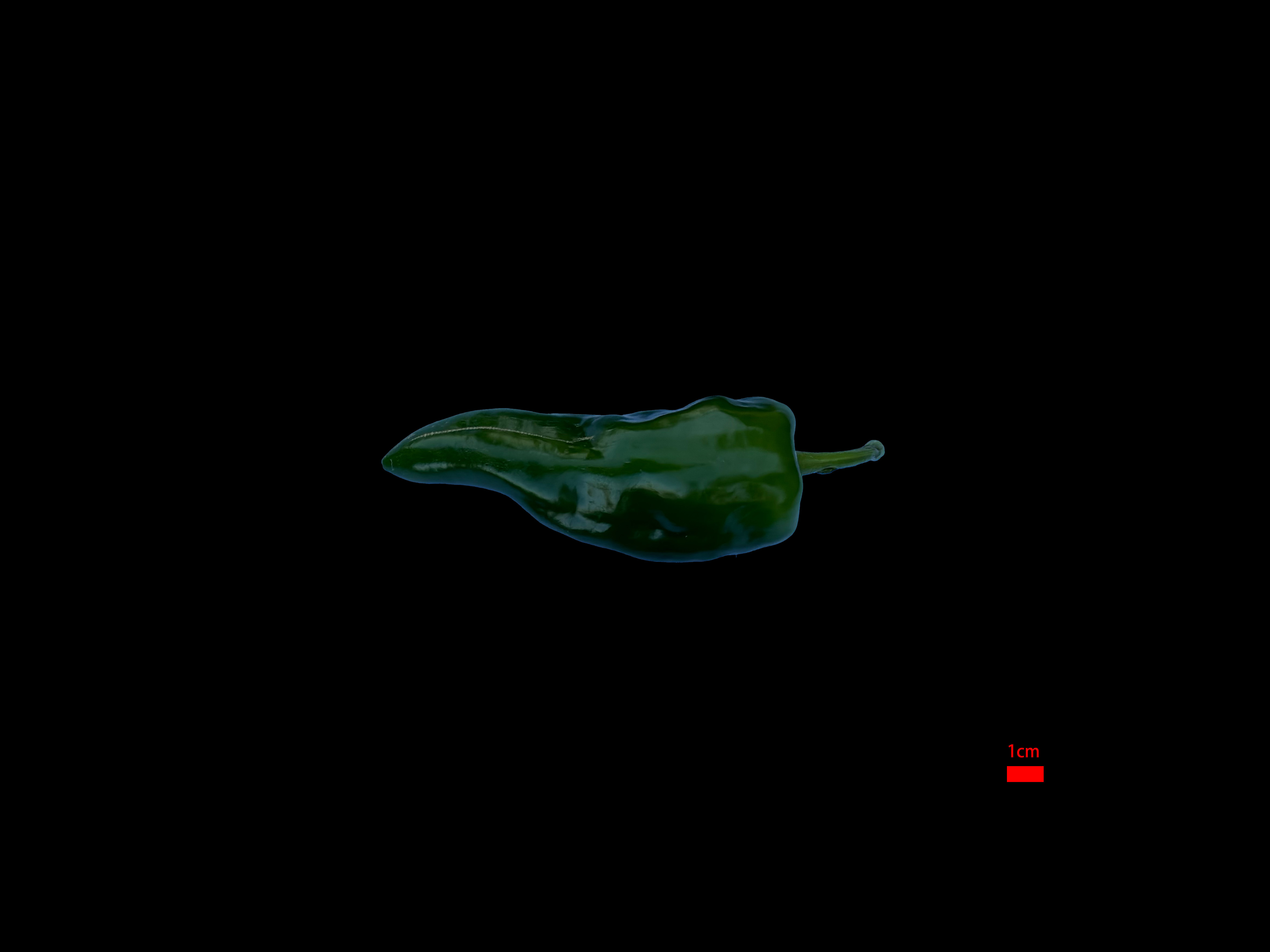

Supplement: Supplementary file 1 [file plants-15-02103-s001.zip › plants-4383327-supplementary/pepper_original_data/cone/219-2.jpg]

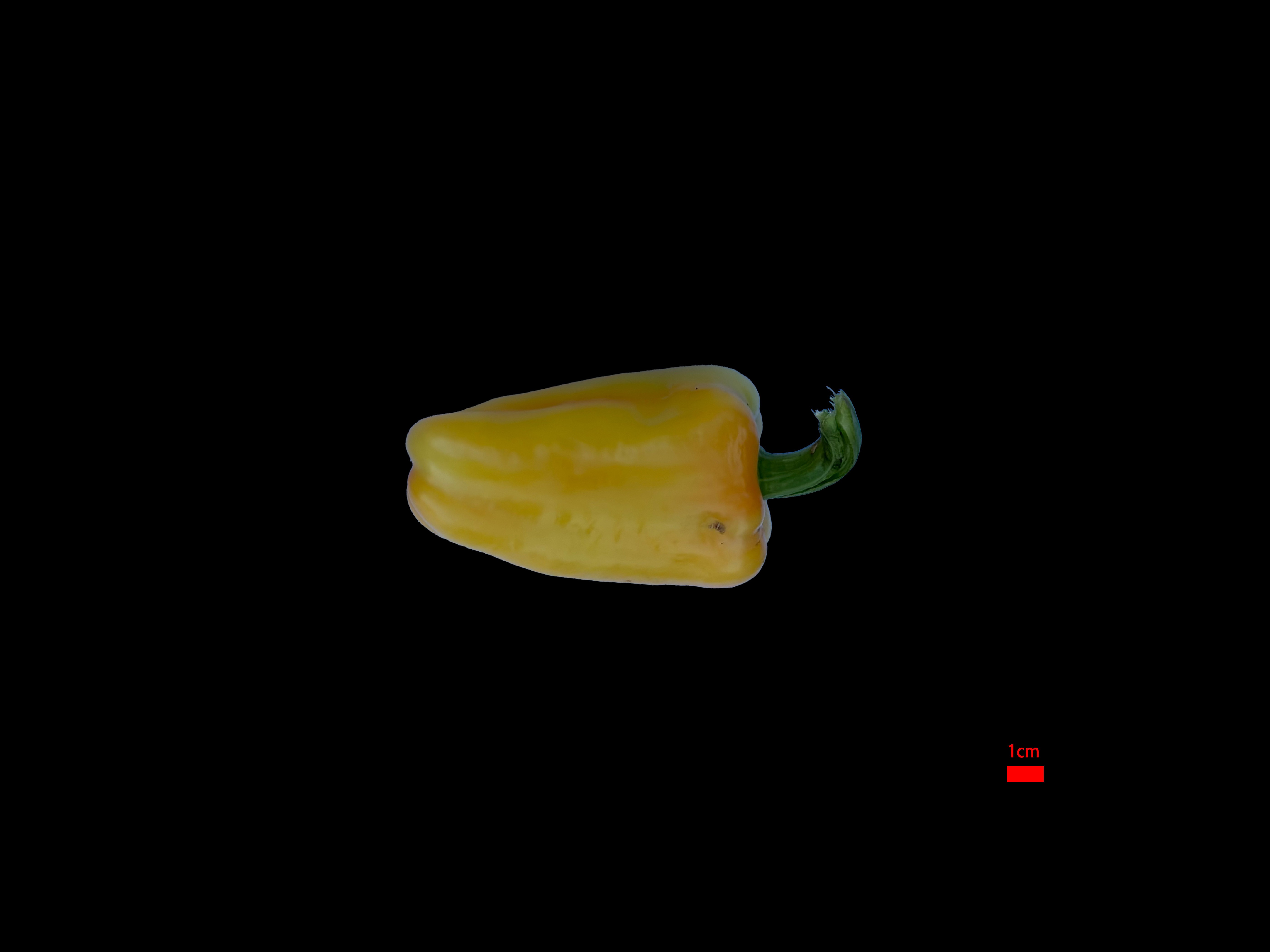

Supplement: Supplementary file 1 [file plants-15-02103-s001.zip › plants-4383327-supplementary/pepper_original_data/cone/229-1.jpg]

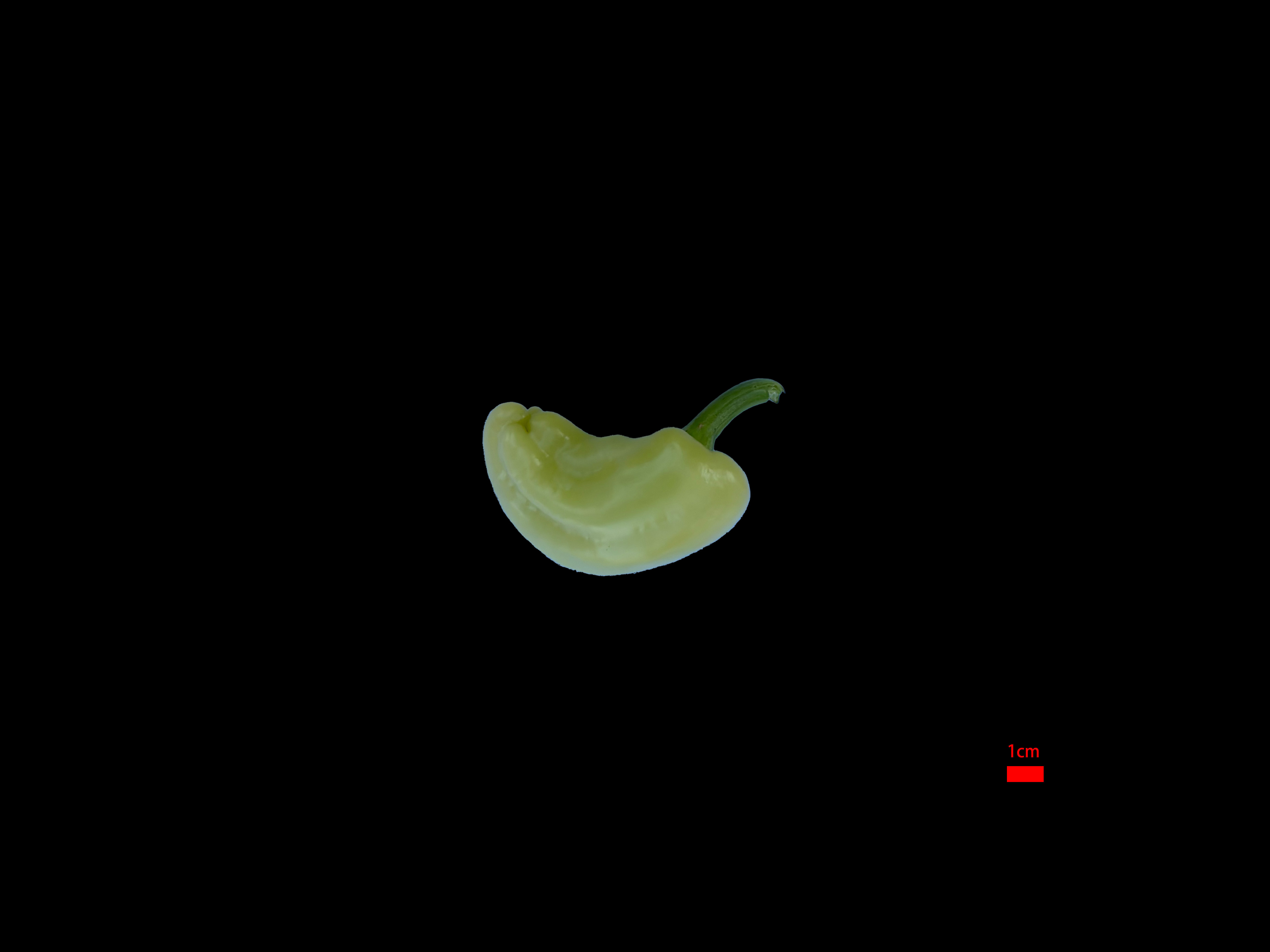

Supplement: Supplementary file 1 [file plants-15-02103-s001.zip › plants-4383327-supplementary/pepper_original_data/cone/229-10.jpg]

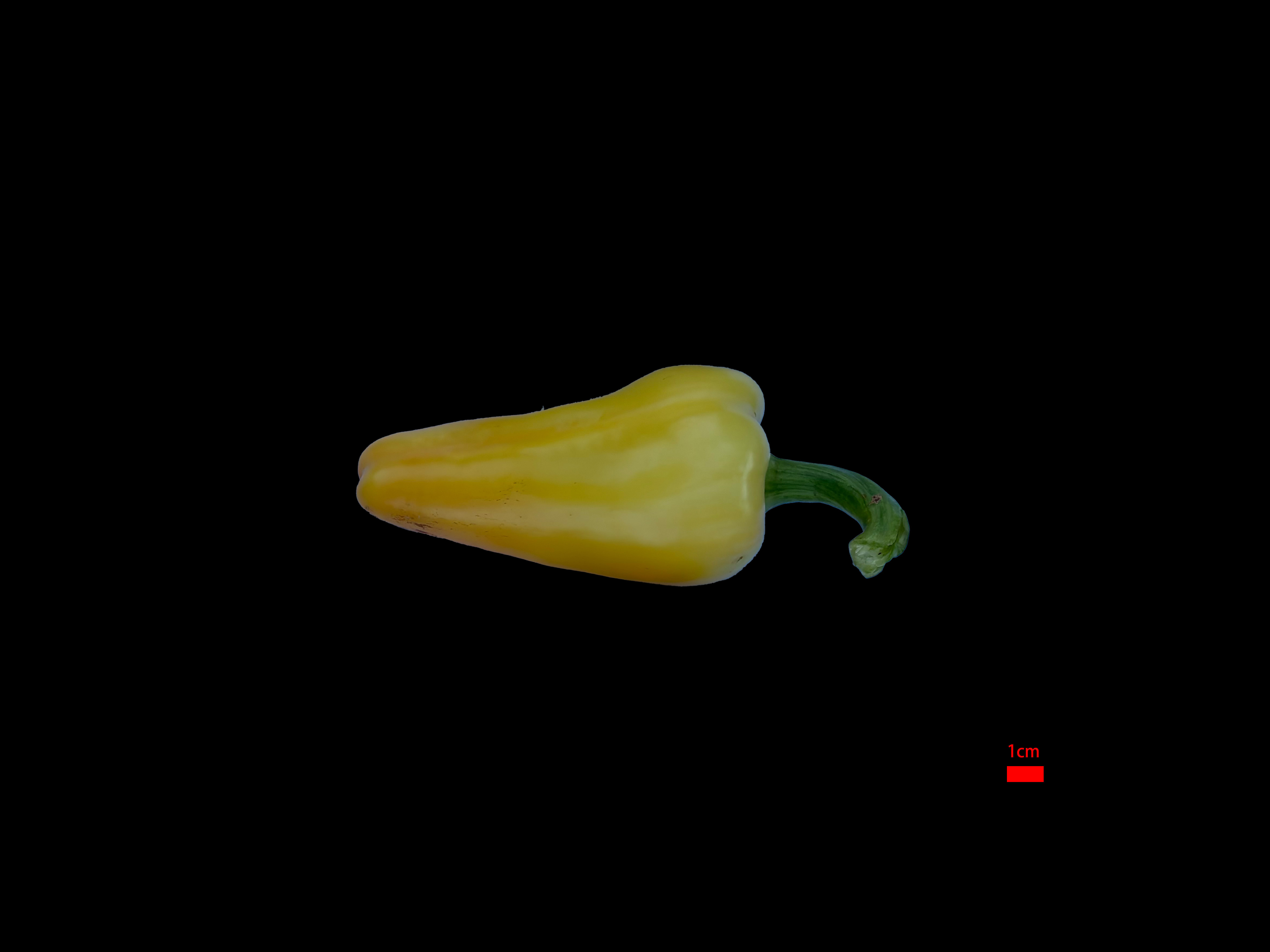

Supplement: Supplementary file 1 [file plants-15-02103-s001.zip › plants-4383327-supplementary/pepper_original_data/cone/229-2.jpg]

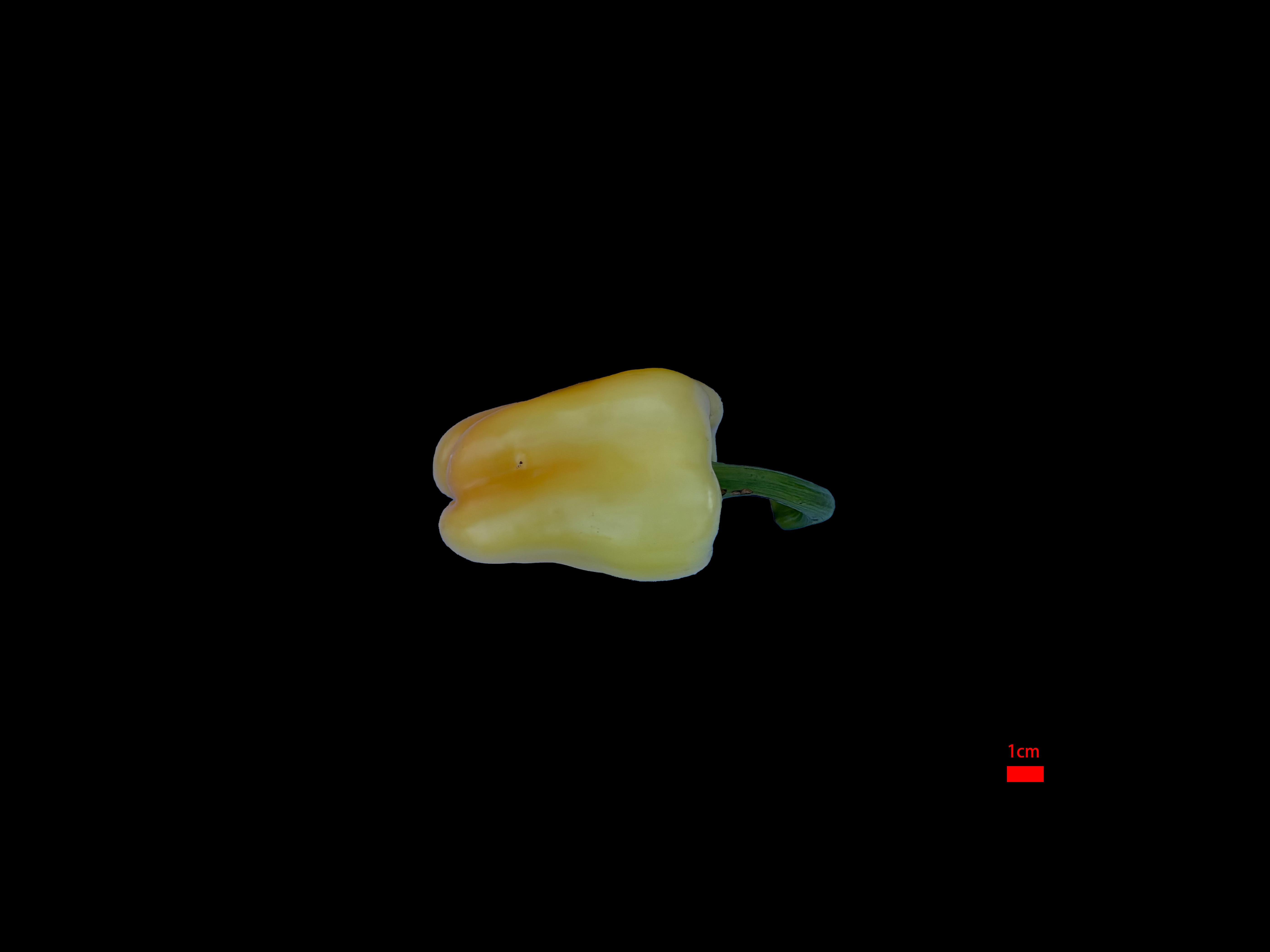

Supplement: Supplementary file 1 [file plants-15-02103-s001.zip › plants-4383327-supplementary/pepper_original_data/cone/229-3.jpg]

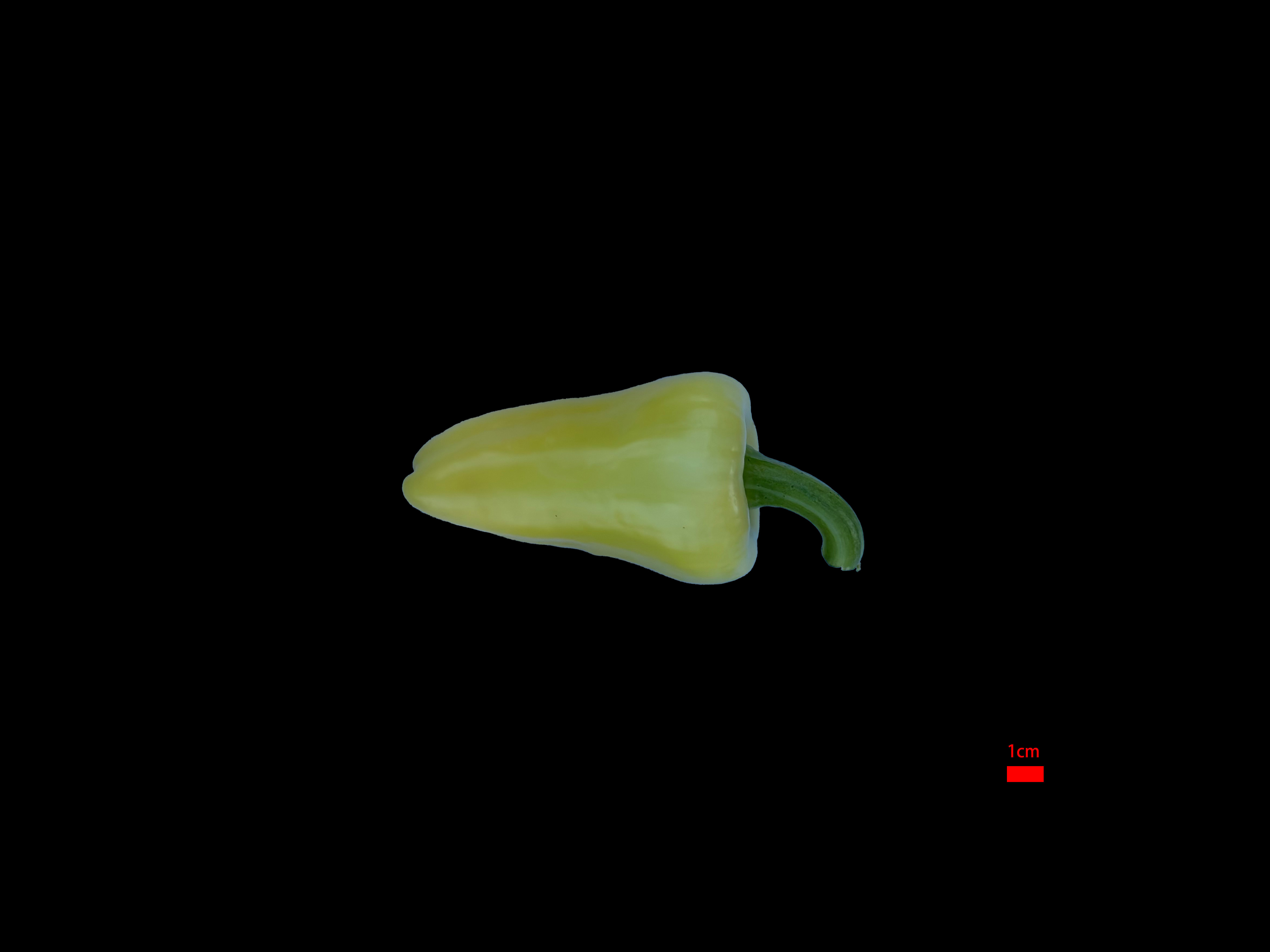

Supplement: Supplementary file 1 [file plants-15-02103-s001.zip › plants-4383327-supplementary/pepper_original_data/cone/229-4.jpg]

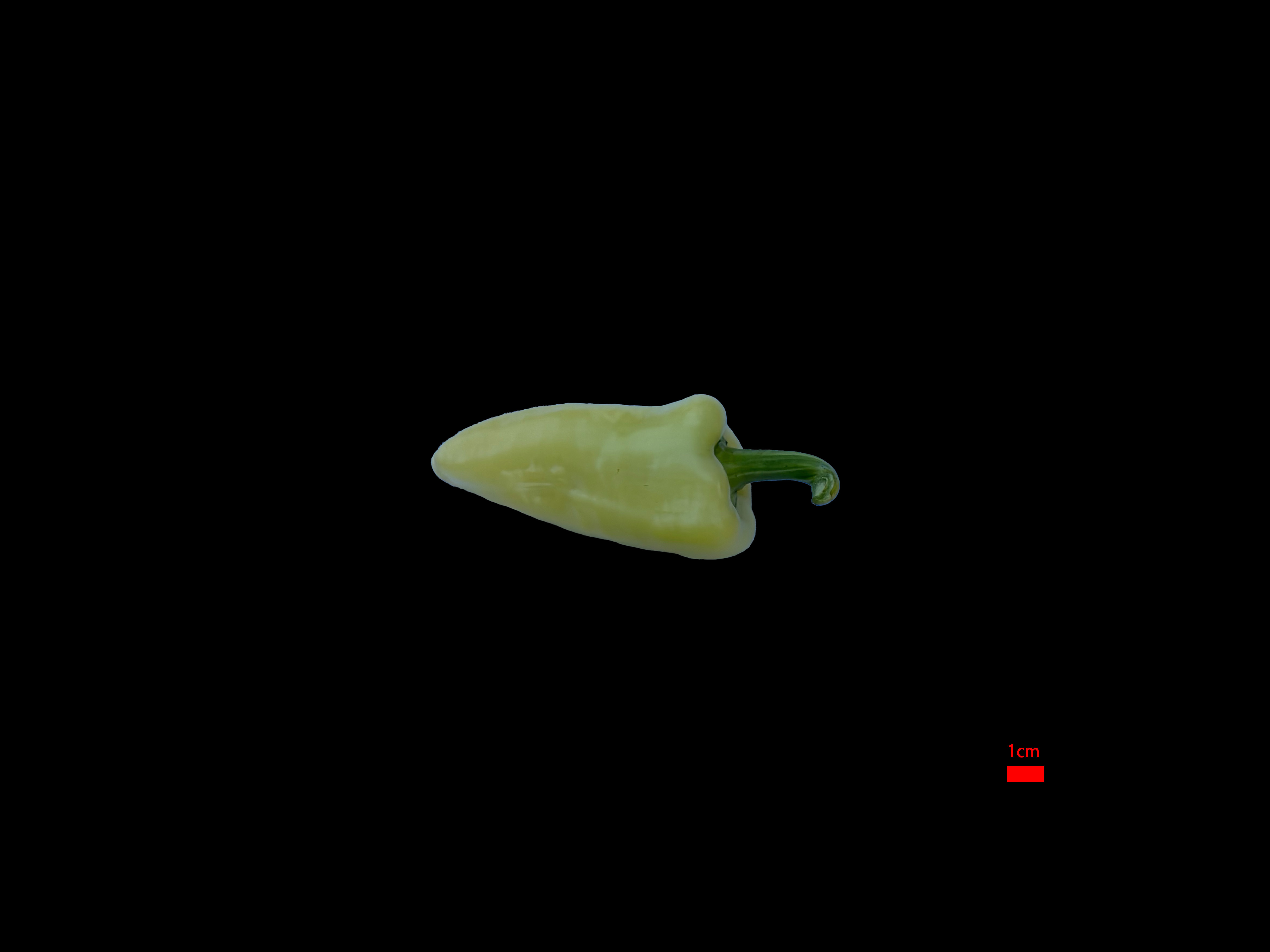

Supplement: Supplementary file 1 [file plants-15-02103-s001.zip › plants-4383327-supplementary/pepper_original_data/cone/229-6.jpg]

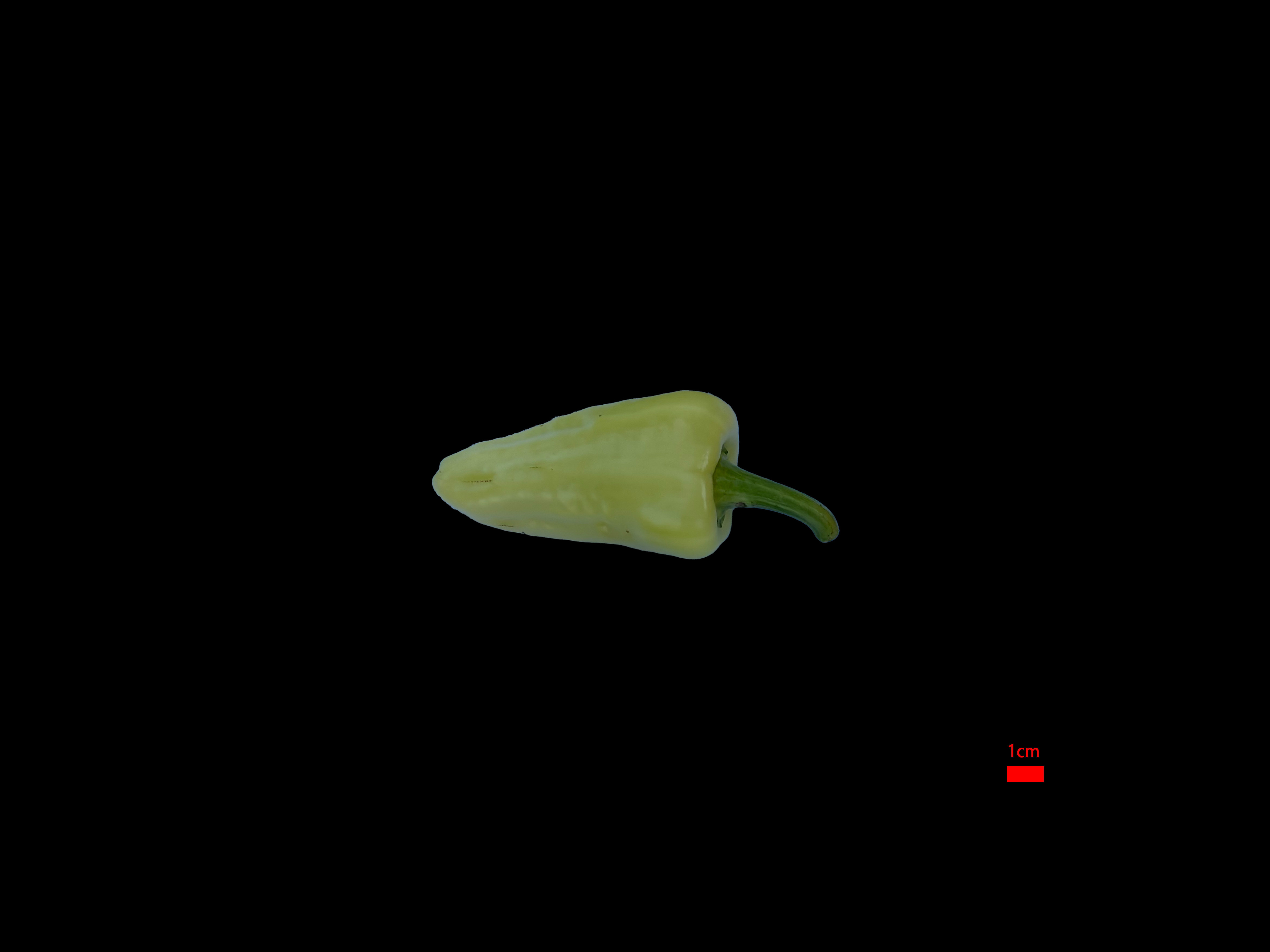

Supplement: Supplementary file 1 [file plants-15-02103-s001.zip › plants-4383327-supplementary/pepper_original_data/cone/229-7.jpg]

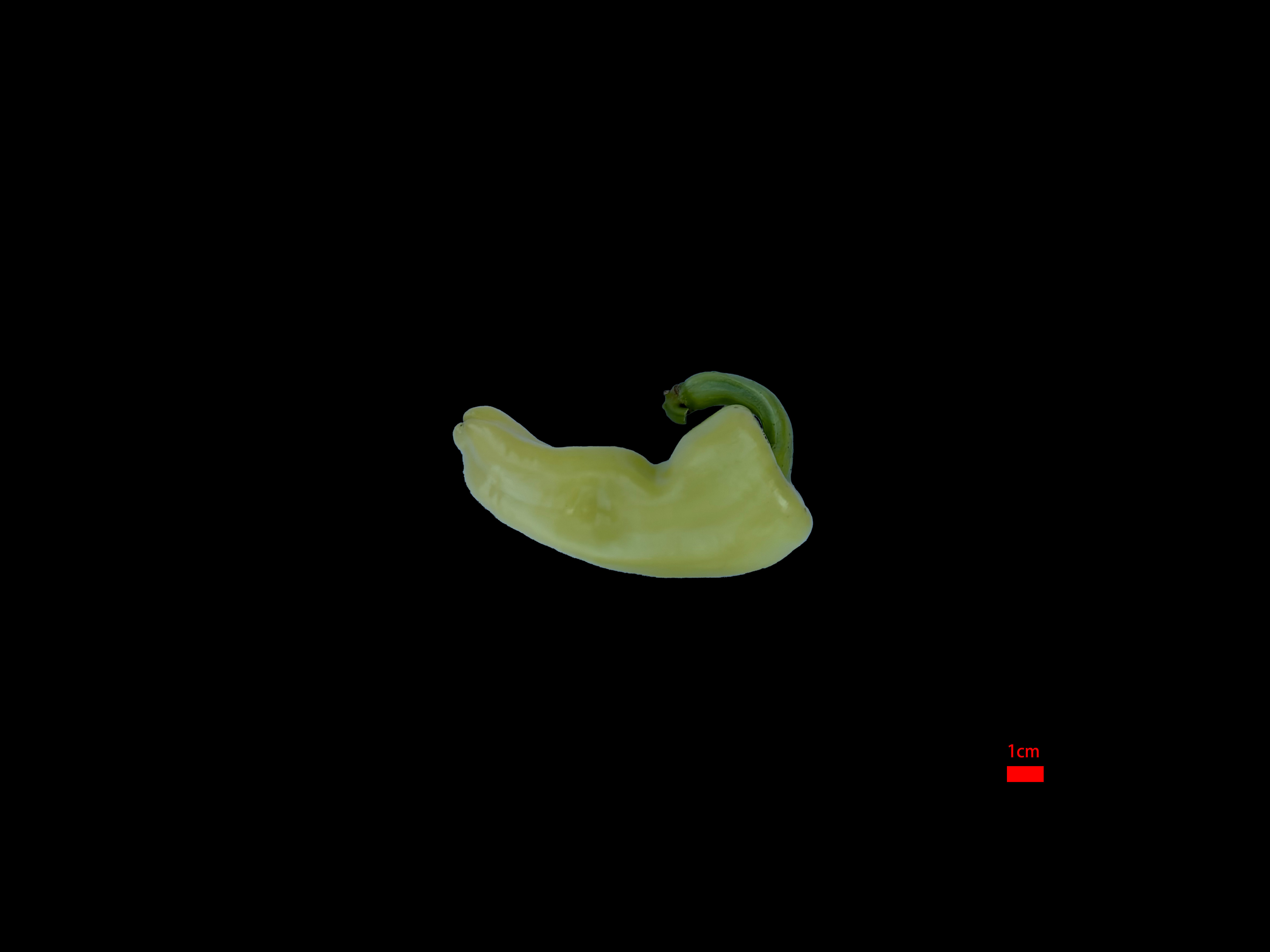

Supplement: Supplementary file 1 [file plants-15-02103-s001.zip › plants-4383327-supplementary/pepper_original_data/cone/229-9.jpg]

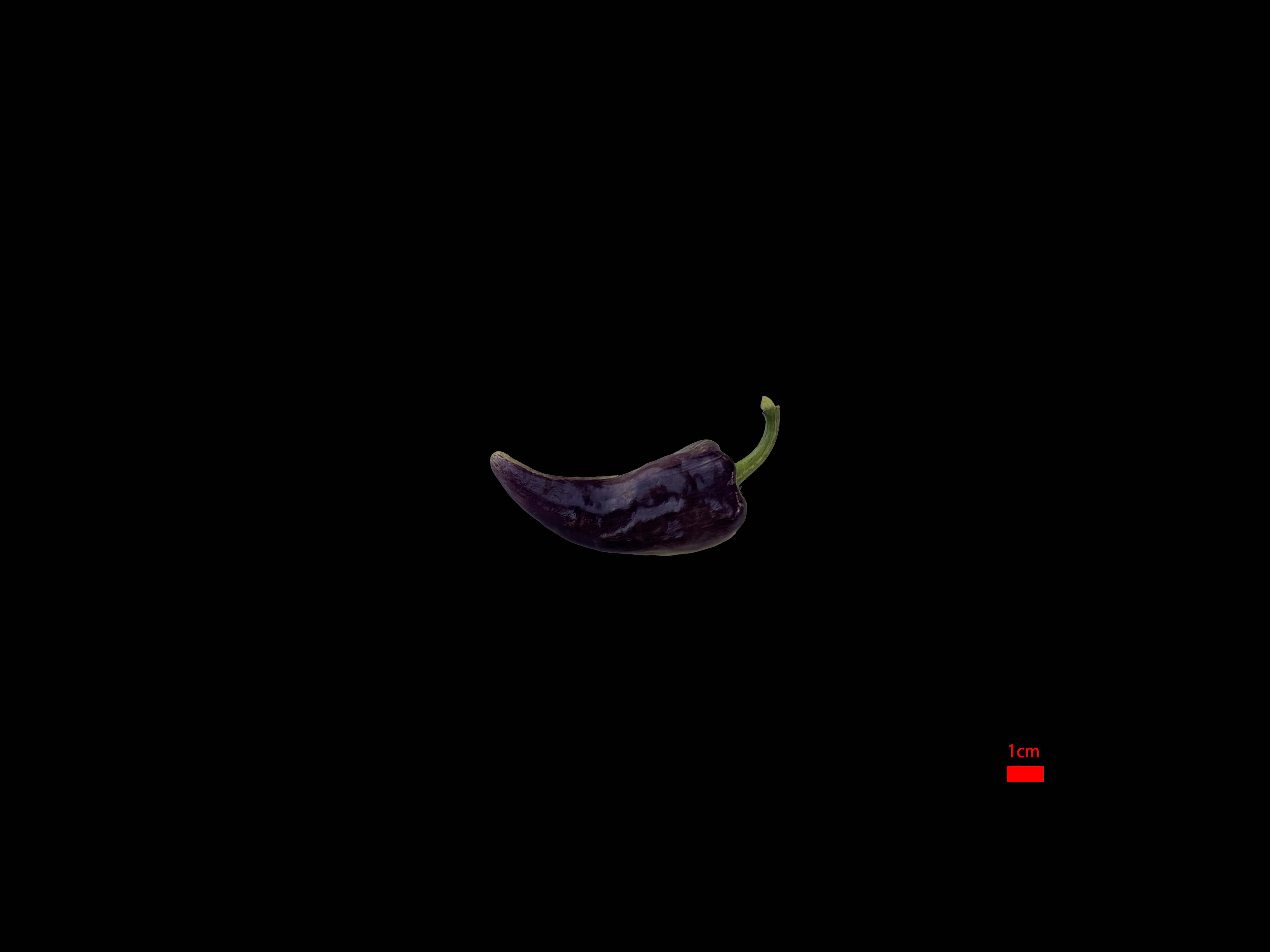

Supplement: Supplementary file 1 [file plants-15-02103-s001.zip › plants-4383327-supplementary/pepper_original_data/cone/30-1.jpg]

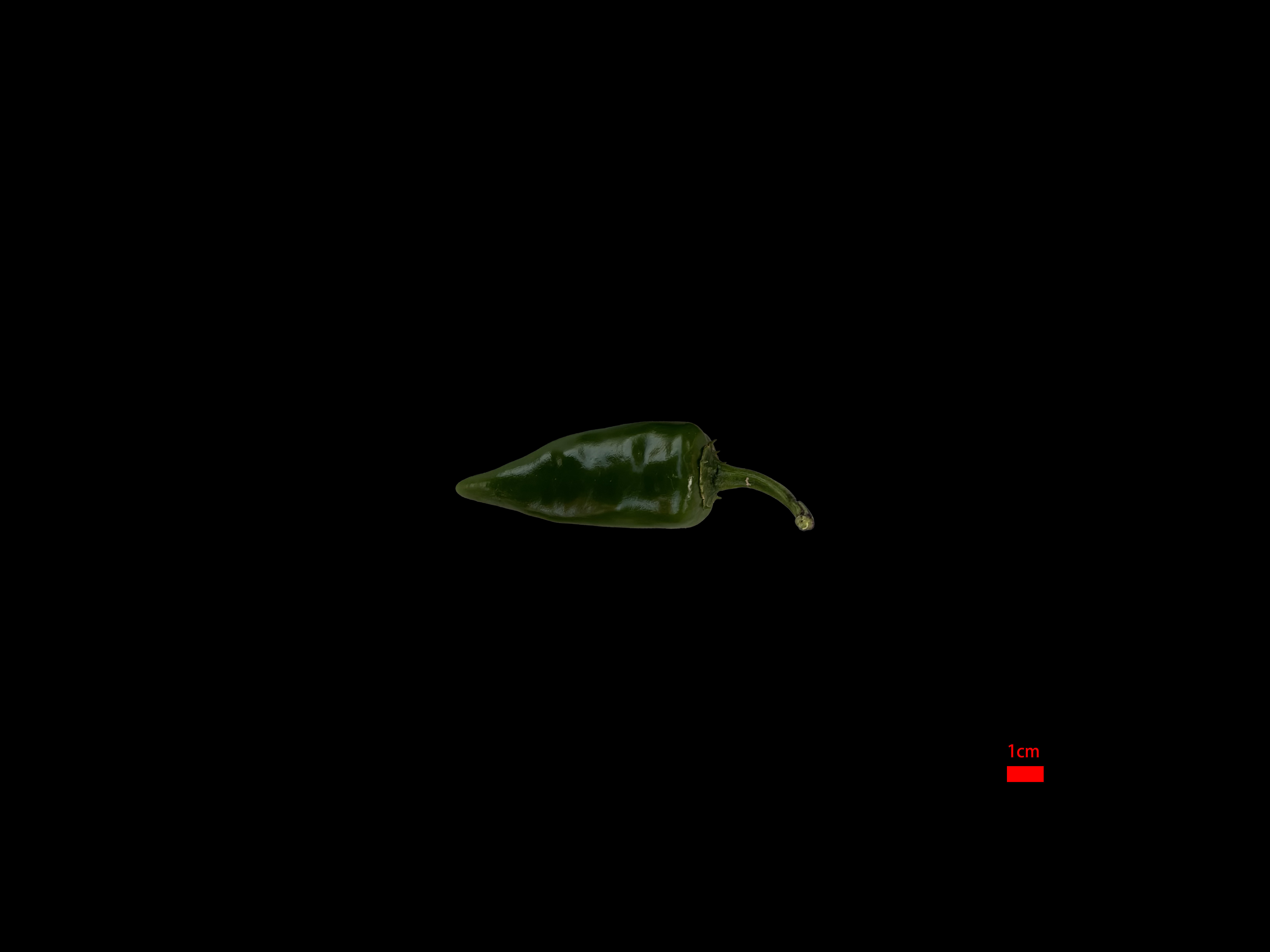

Supplement: Supplementary file 1 [file plants-15-02103-s001.zip › plants-4383327-supplementary/pepper_original_data/cone/30-10.jpg]

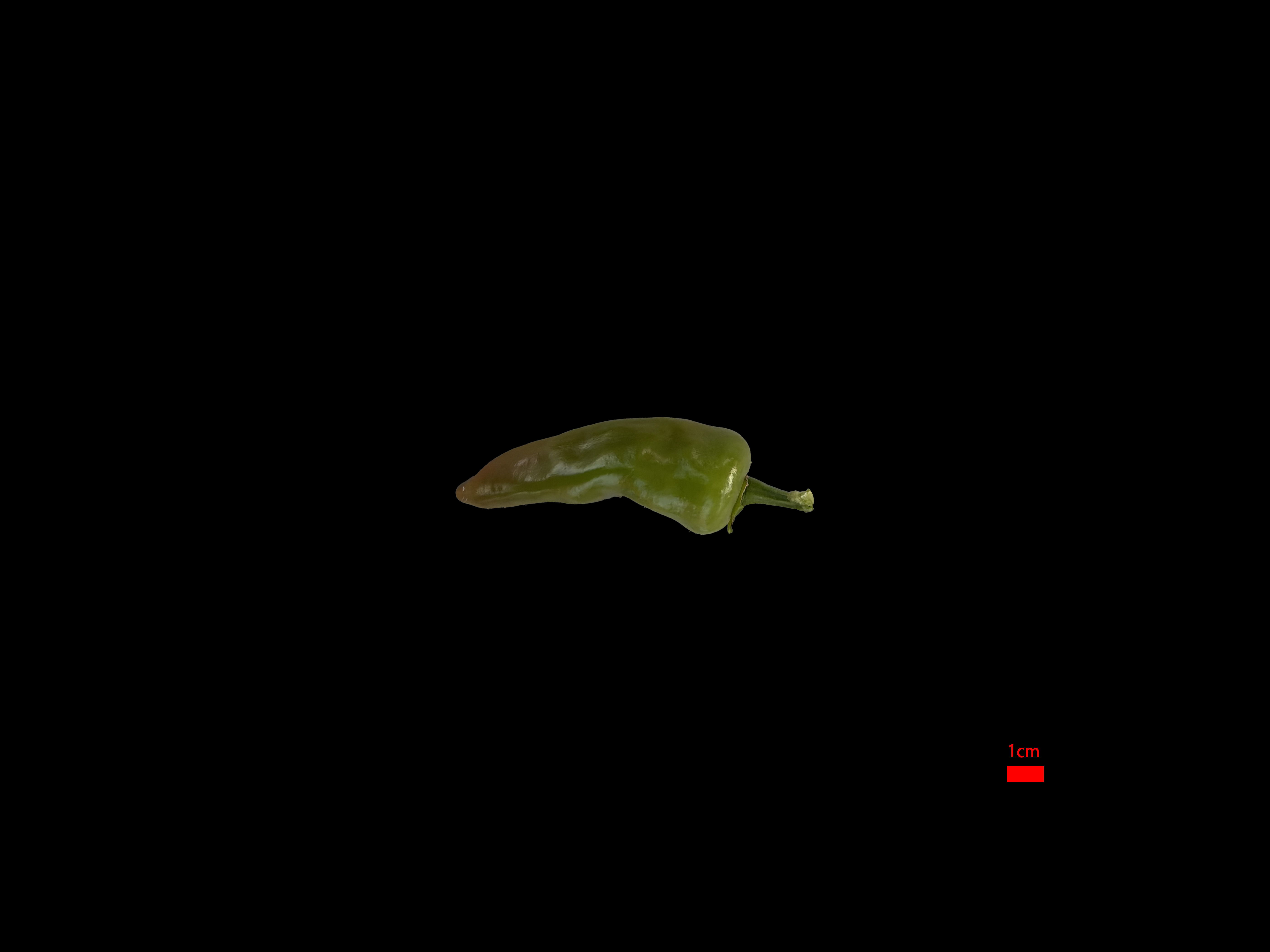

Supplement: Supplementary file 1 [file plants-15-02103-s001.zip › plants-4383327-supplementary/pepper_original_data/cone/30-11.jpg]

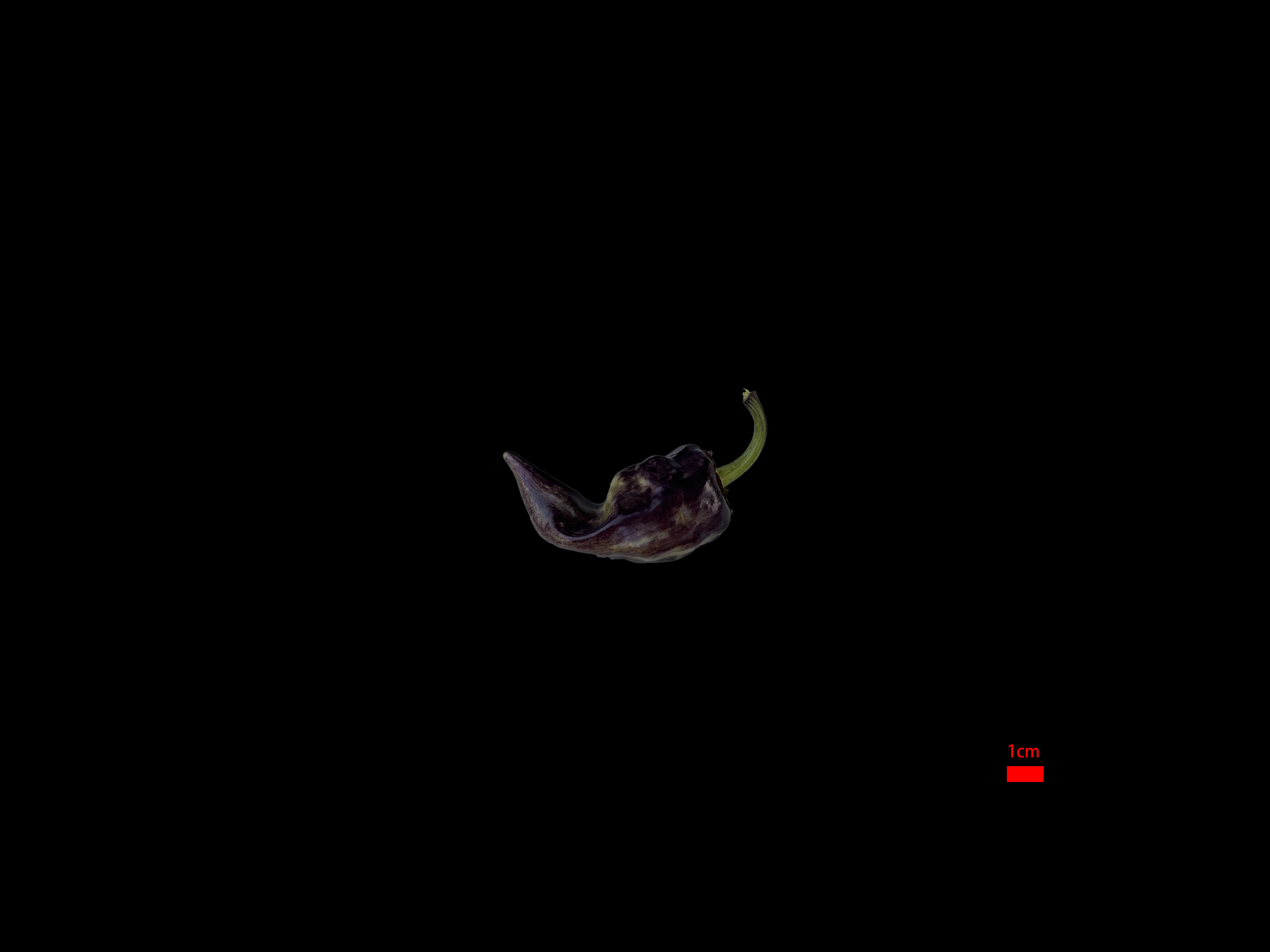

Supplement: Supplementary file 1 [file plants-15-02103-s001.zip › plants-4383327-supplementary/pepper_original_data/cone/30-2.jpg]

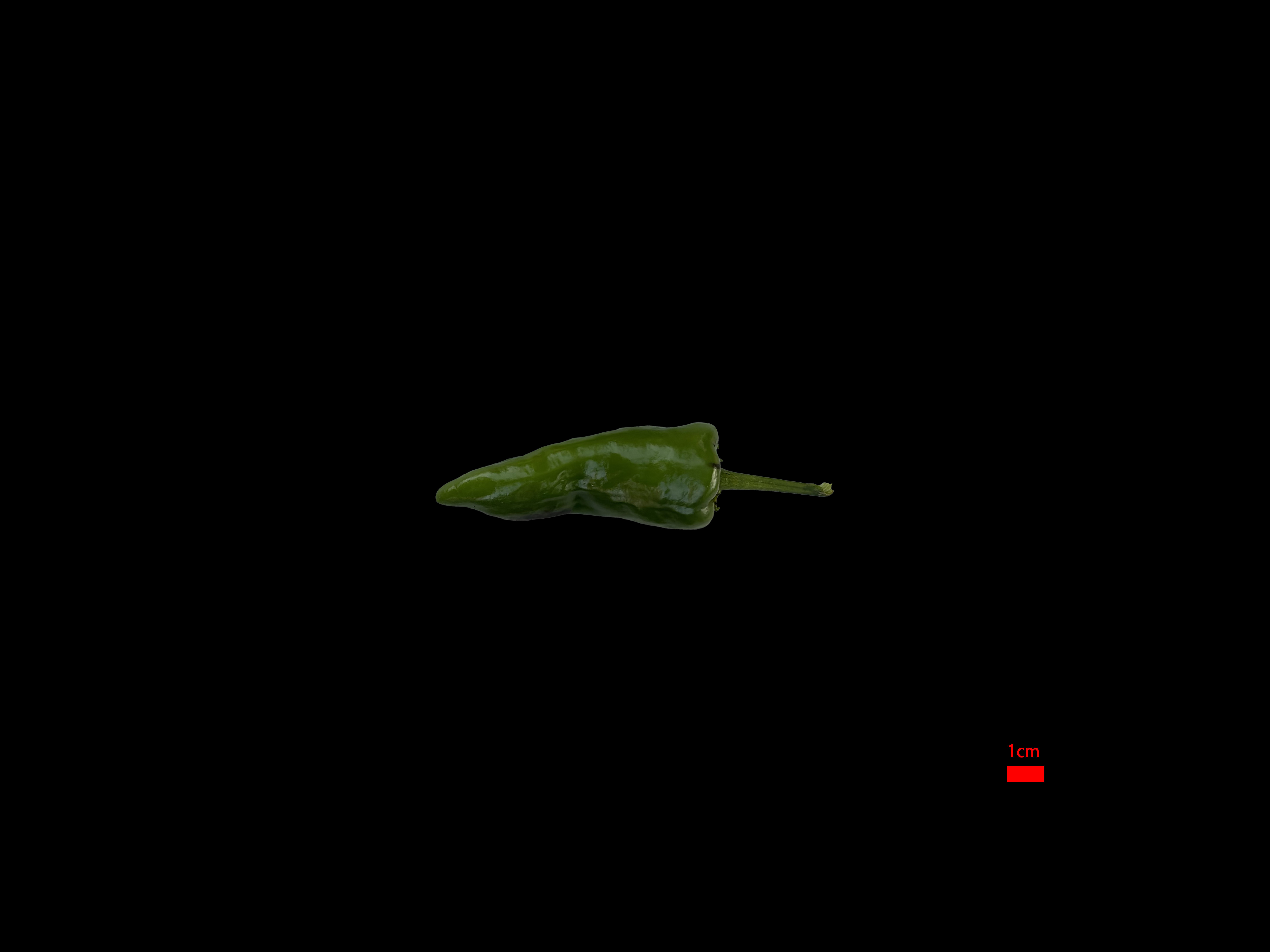

Supplement: Supplementary file 1 [file plants-15-02103-s001.zip › plants-4383327-supplementary/pepper_original_data/cone/30-3.jpg]

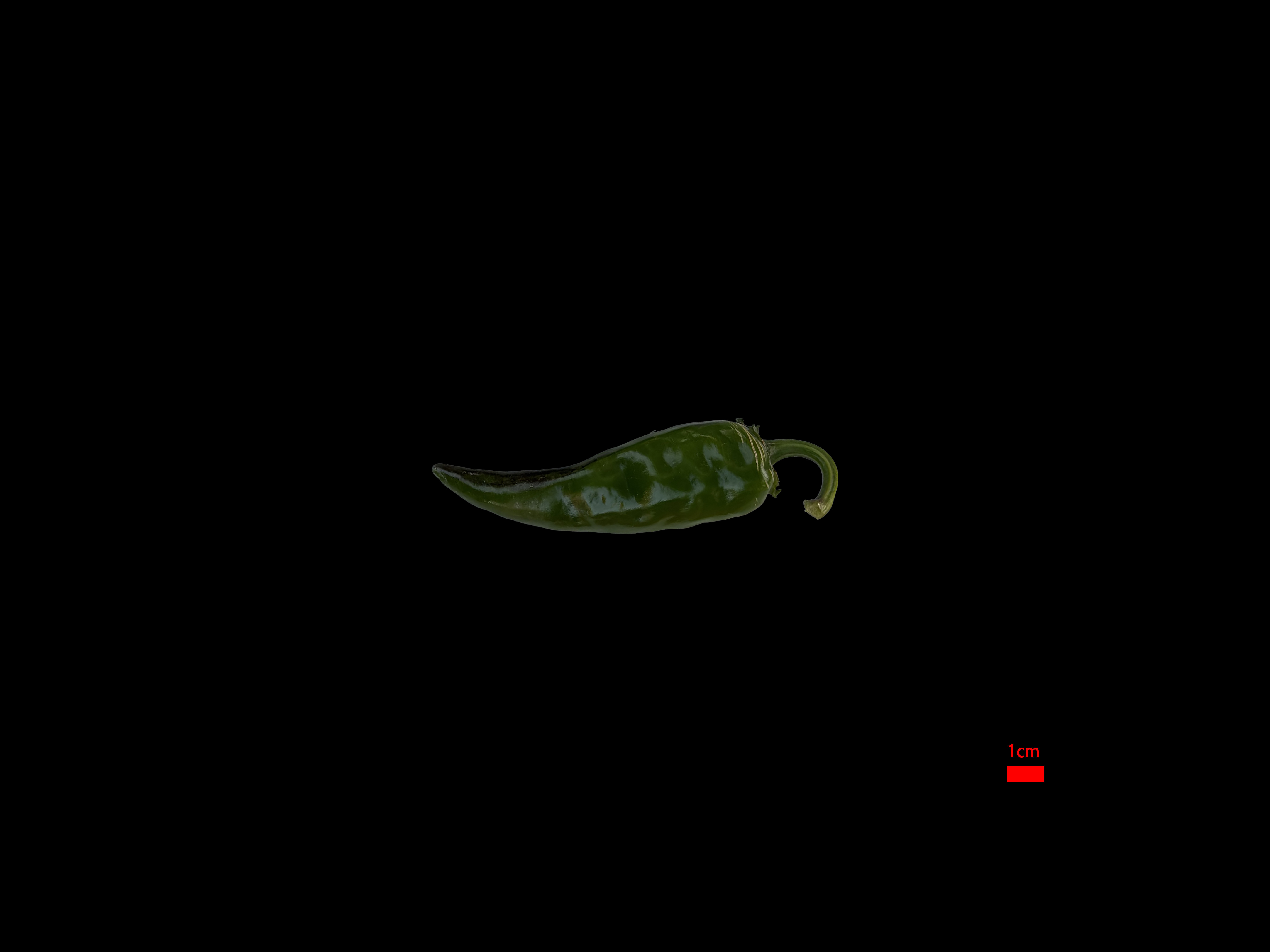

Supplement: Supplementary file 1 [file plants-15-02103-s001.zip › plants-4383327-supplementary/pepper_original_data/cone/30-4.jpg]

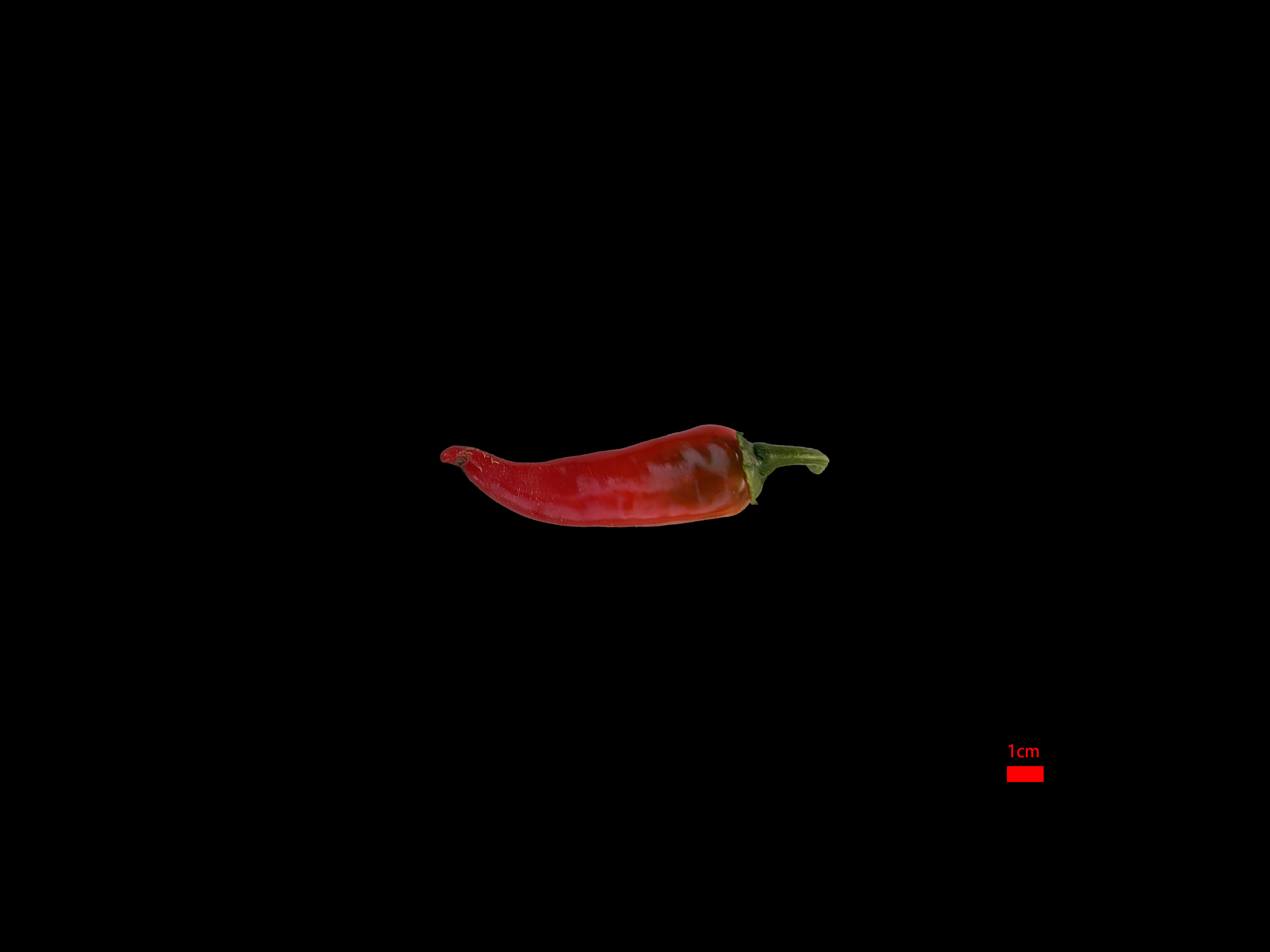

Supplement: Supplementary file 1 [file plants-15-02103-s001.zip › plants-4383327-supplementary/pepper_original_data/cone/30-5.jpg]

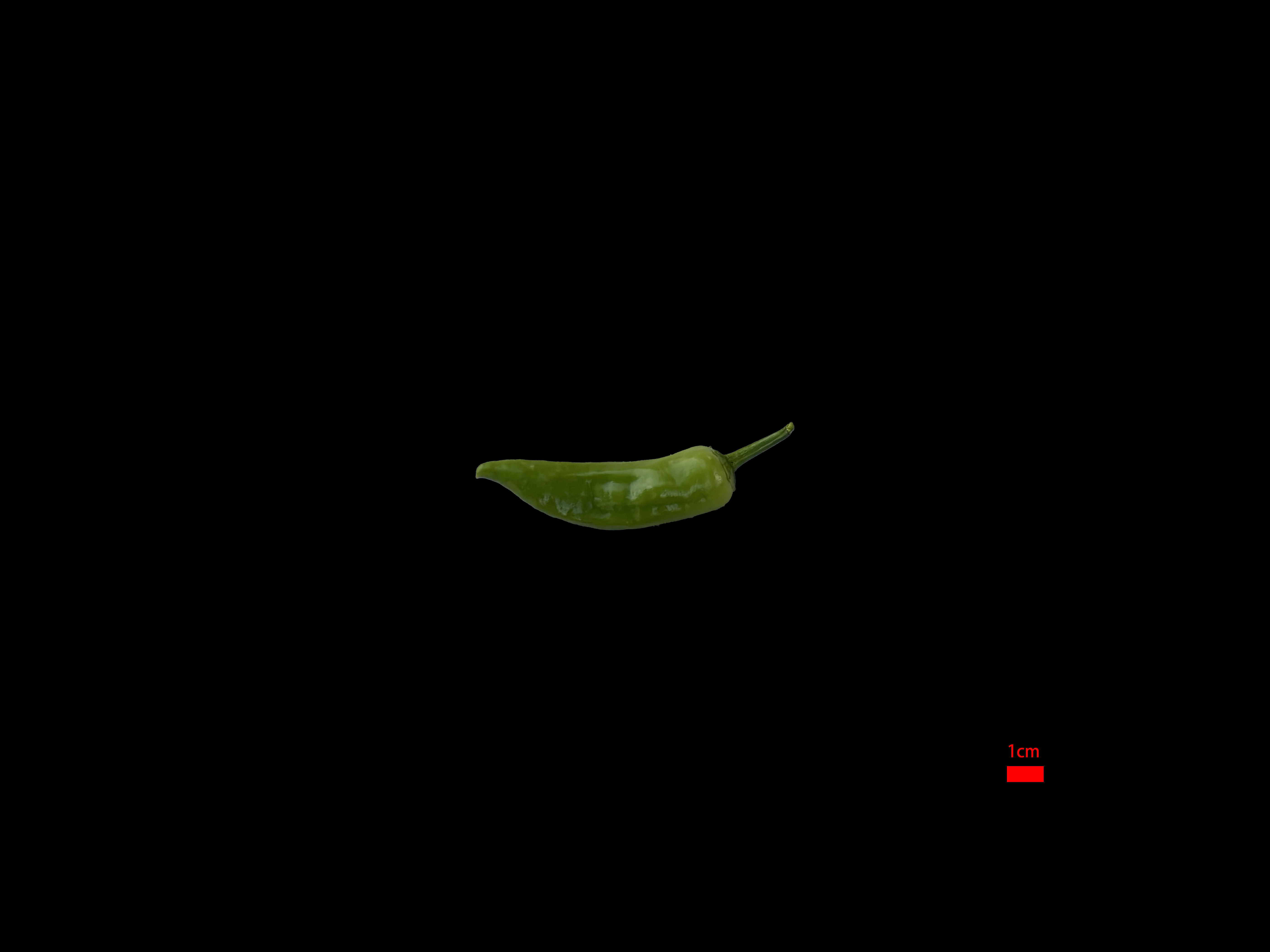

Supplement: Supplementary file 1 [file plants-15-02103-s001.zip › plants-4383327-supplementary/pepper_original_data/cone/30-6.jpg]

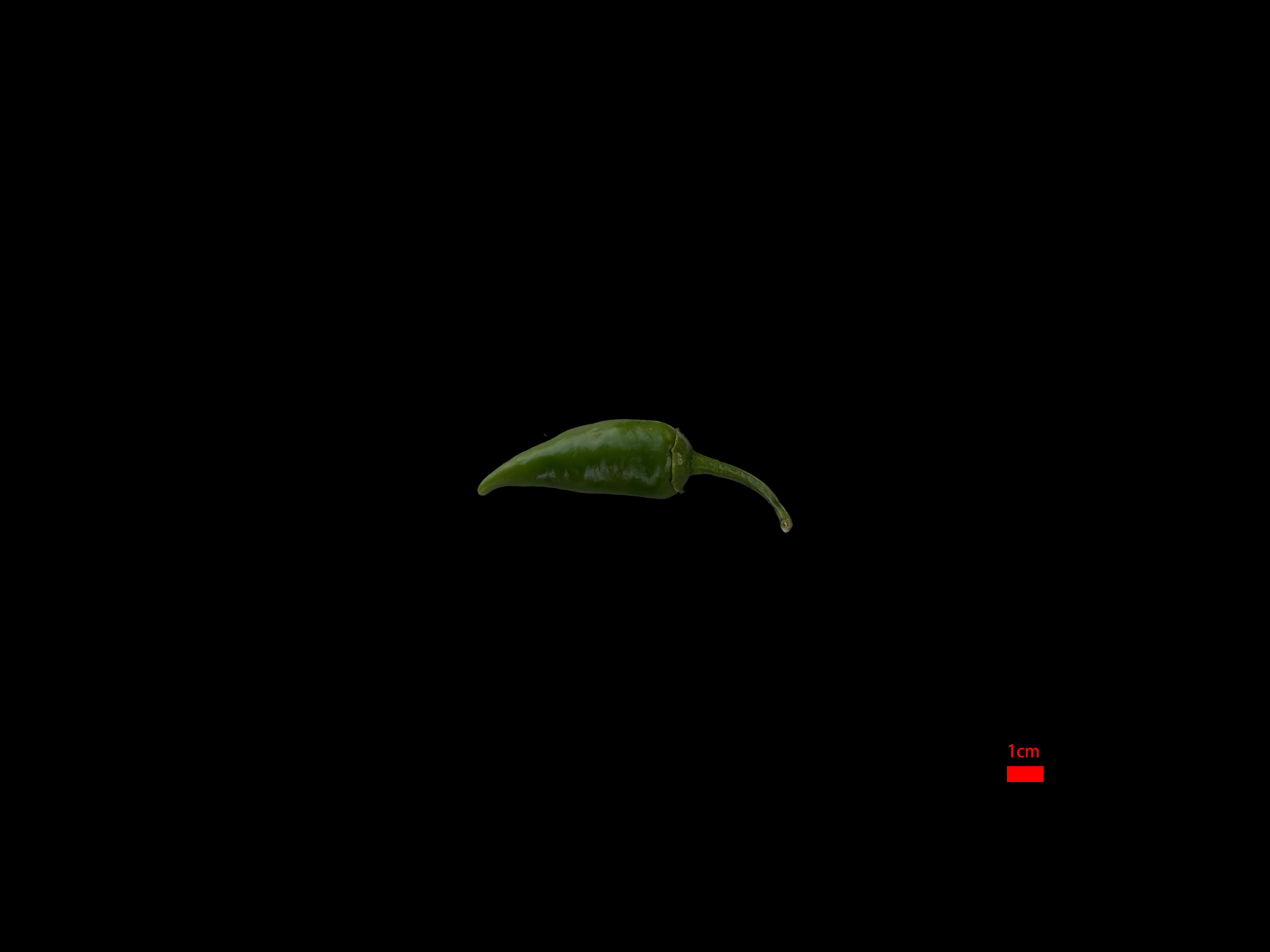

Supplement: Supplementary file 1 [file plants-15-02103-s001.zip › plants-4383327-supplementary/pepper_original_data/cone/30-7.jpg]

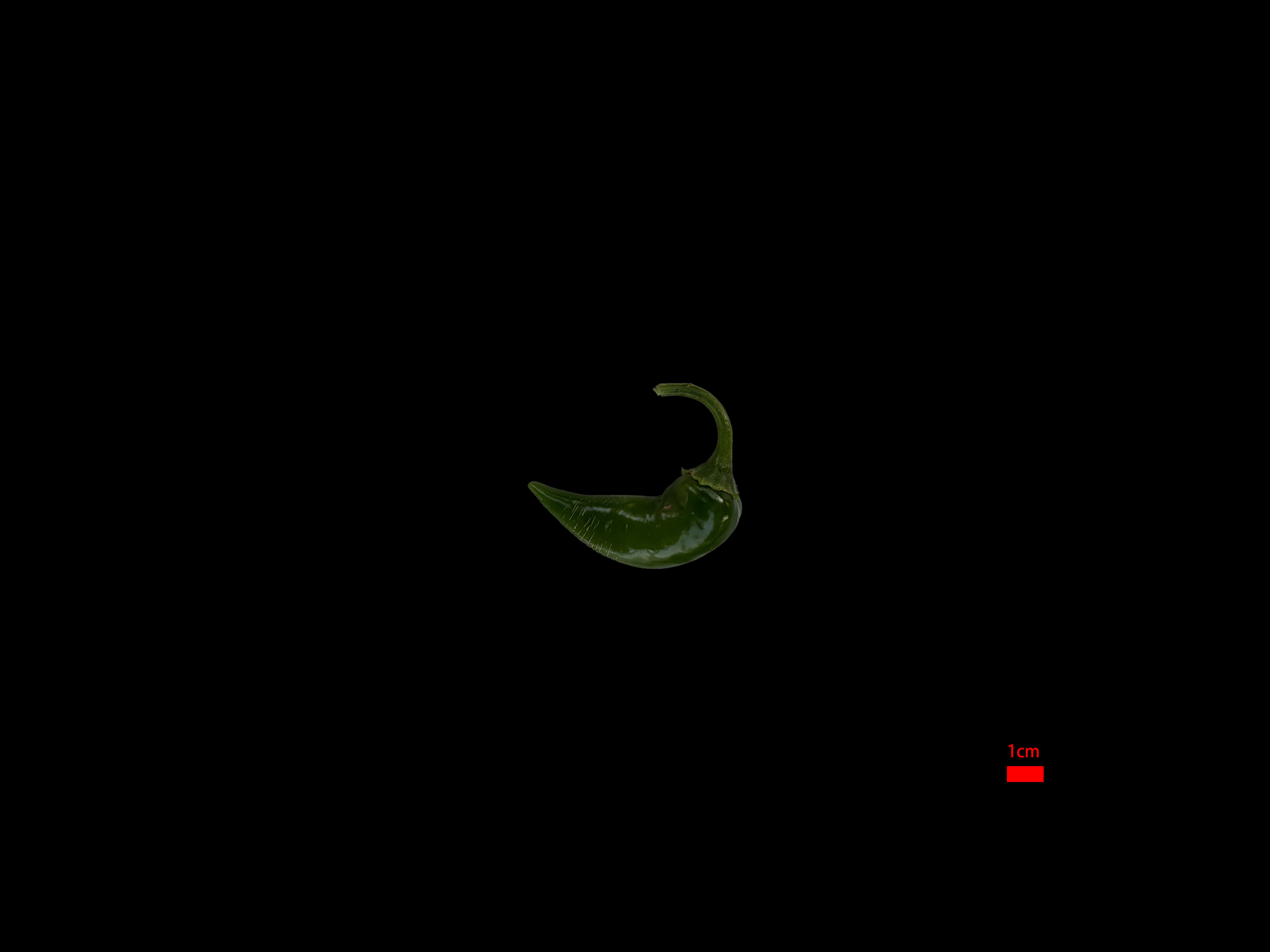

Supplement: Supplementary file 1 [file plants-15-02103-s001.zip › plants-4383327-supplementary/pepper_original_data/cone/30-8.jpg]

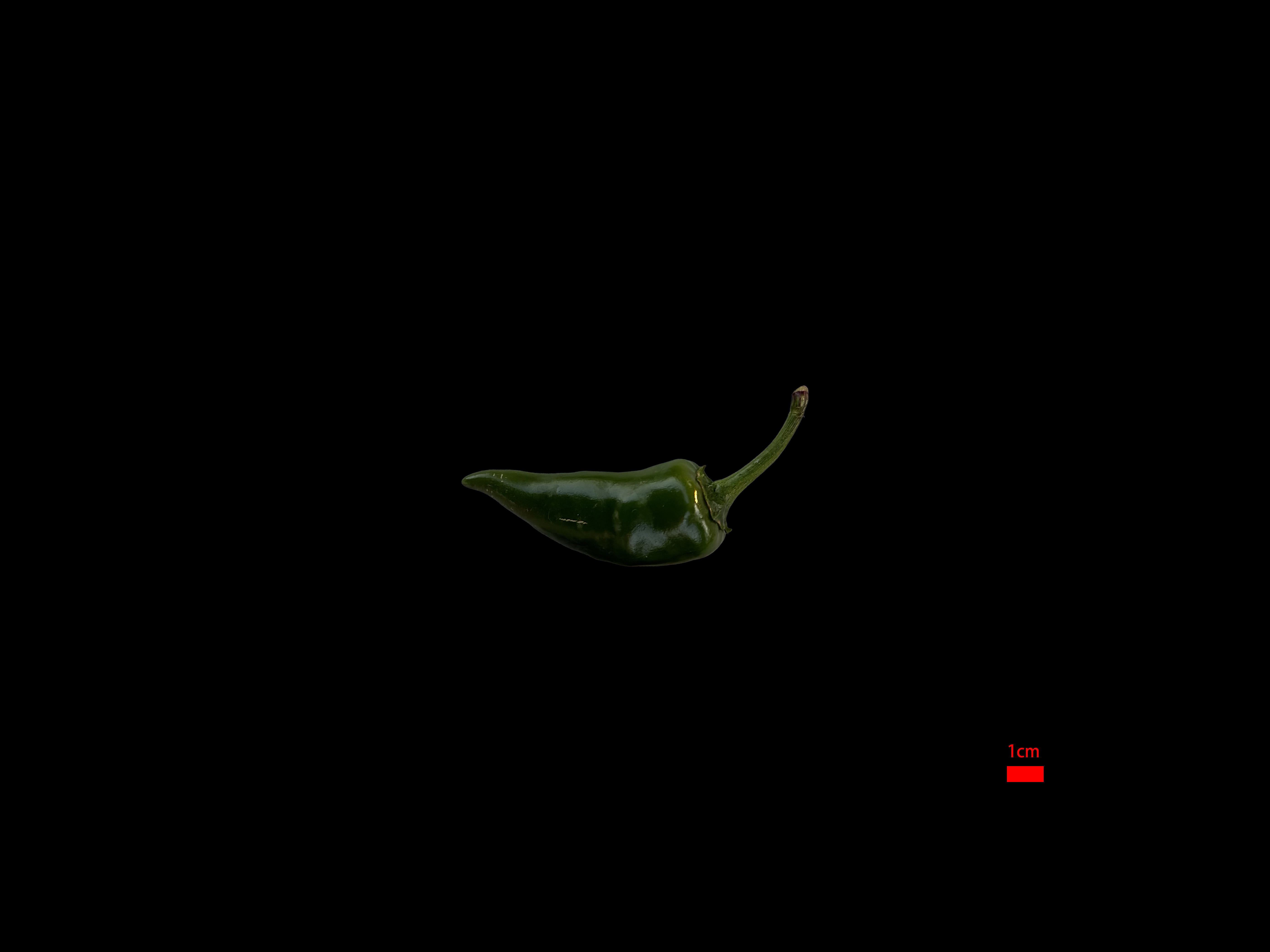

Supplement: Supplementary file 1 [file plants-15-02103-s001.zip › plants-4383327-supplementary/pepper_original_data/cone/30-9.jpg]

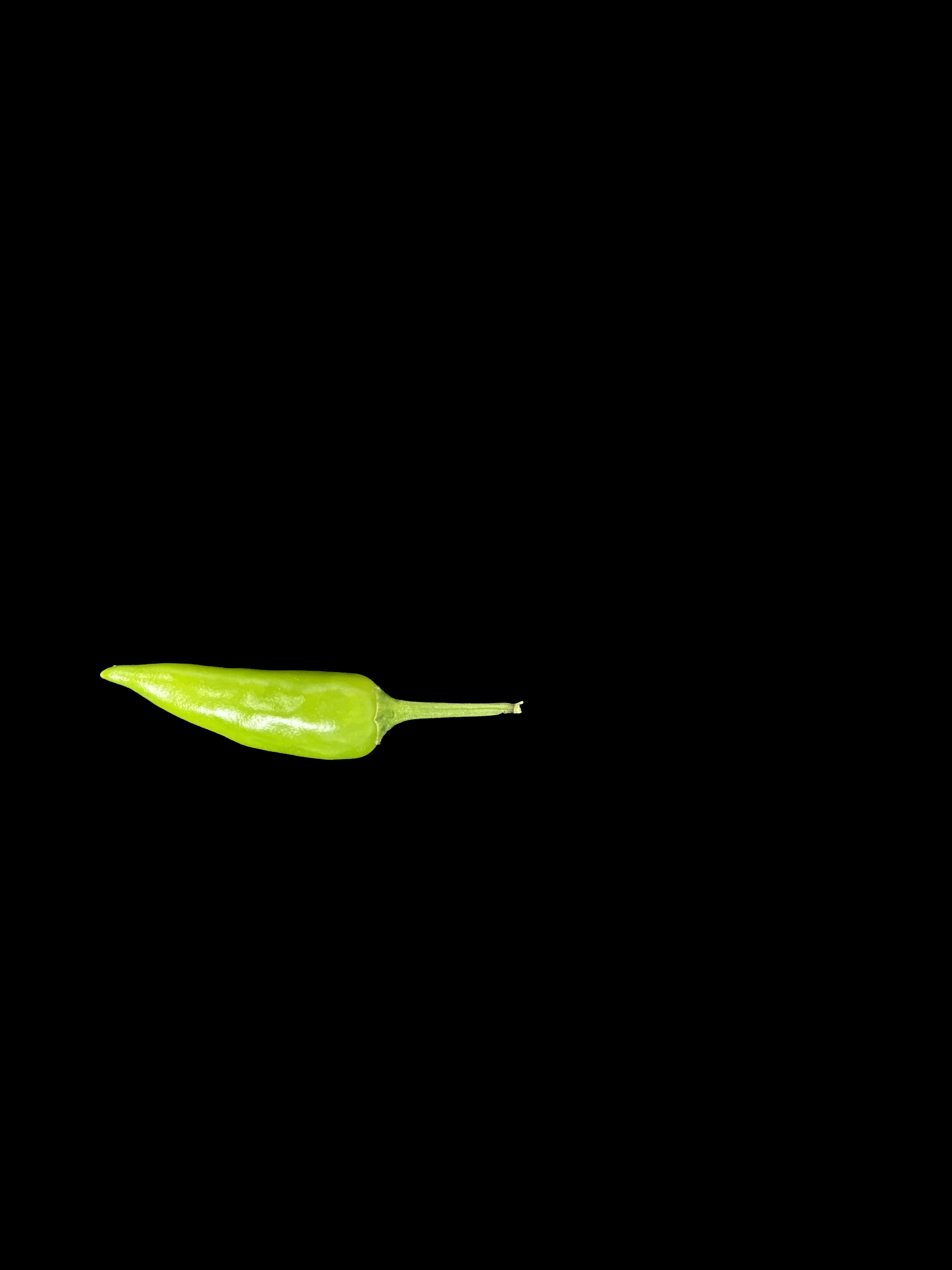

Supplement: Supplementary file 1 [file plants-15-02103-s001.zip › plants-4383327-supplementary/pepper_original_data/cone/33.1.jpg]

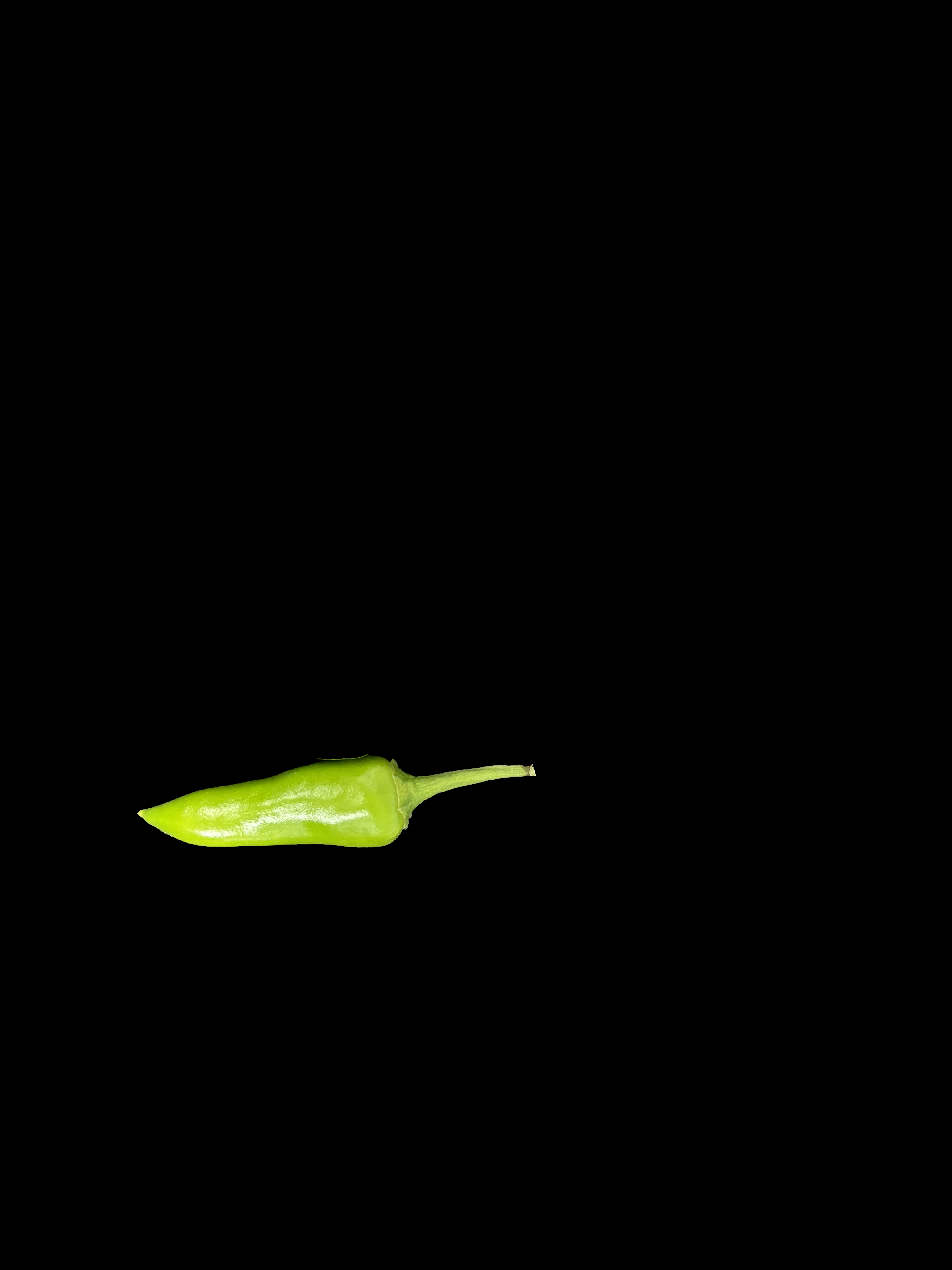

Supplement: Supplementary file 1 [file plants-15-02103-s001.zip › plants-4383327-supplementary/pepper_original_data/cone/33.2.jpg]

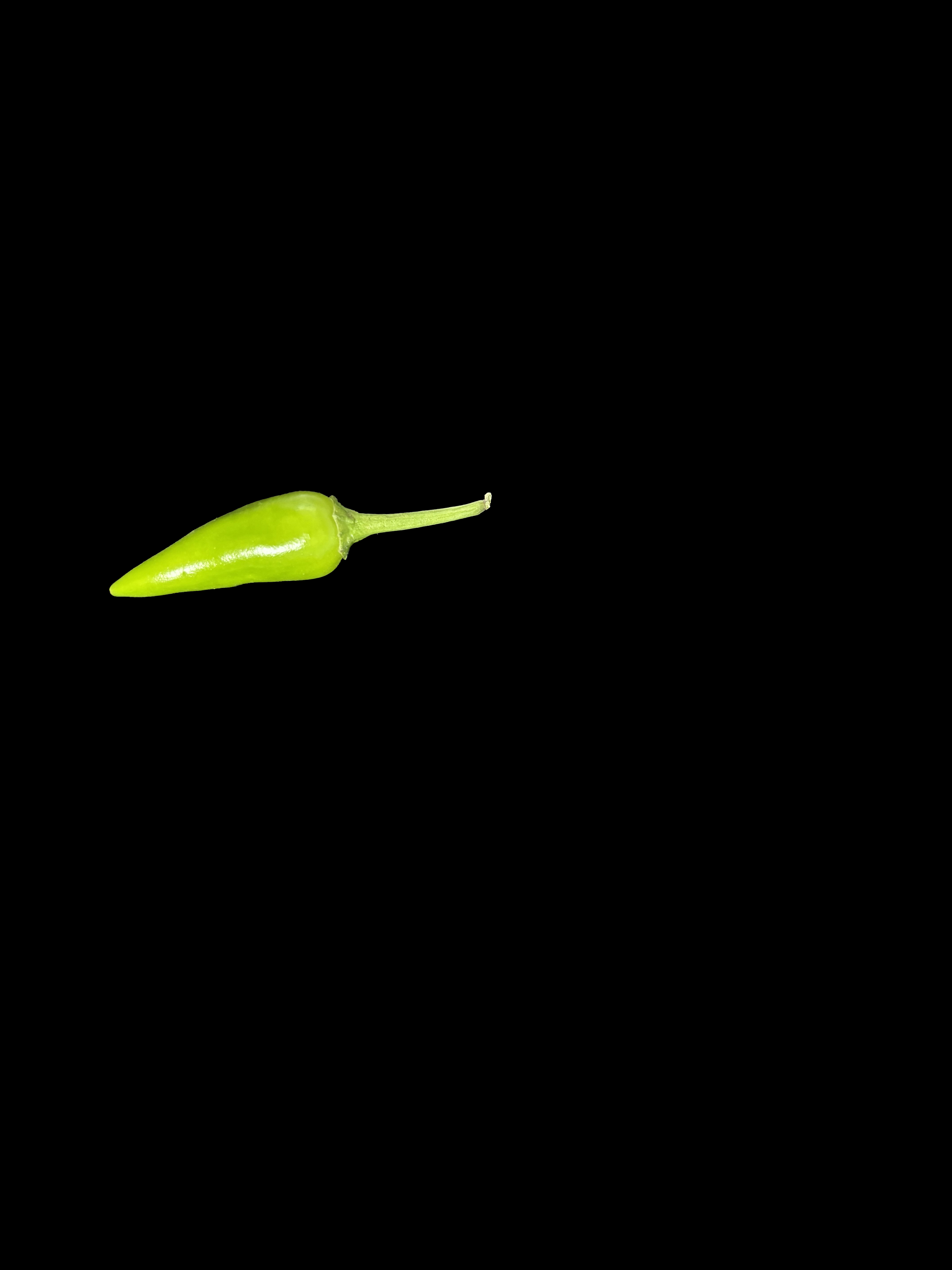

Supplement: Supplementary file 1 [file plants-15-02103-s001.zip › plants-4383327-supplementary/pepper_original_data/cone/33.jpg]
